# SCTS Annual Meeting 2022 Abstracts

**DOI:** 10.1186/s13019-023-02292-4

**Published:** 2023-07-12

## Adult Cardiac Aorta

### A1 Perfusion Strategies for Thoraco Abdominal Aortic Aneurysms—Our Institutional Experience

#### Selvaraj, Sam, Mr; PVS, Prakash, Mr; Rajamani, Selvakumar, Mr; C George, Thomson, Mr; Santhosh, Gopika, Ms; Shetty, Varun, Dr; Shahansha, S, Mr

##### Narayana Health Hospitals, Bangalore, India

*Journal of Cardiothoracic Surgery* 2023, **18(Supp 1)**:A1

**Background**

Thoraco Abdominal Aortic Aneurysm (TAAA) is rare, occurring in approximately 6–10 per every 100,000 people. But surgical correction of this pathology possesses serious complications like paraplegia and spinal cord problems. The overall 30-day mortality and paraplegia results are 8.5% and 4.2% respectively.

**Methods**

Between Jan 2019 & Sept 2021 we have performed about 12 Thoraco Abdominal Aortic Aneurysm cases and our perfusion techniques for this type of surgery provides good clinical practice and prevents the neuro, spinal cord, gut and renal related complications. All the 12 cases are retrospectively analysed in detail for perfusion techniques, neurological outcome, renal function and the post-operative outcome.

**Operative technique**

CPB established with cannulation on PA, RA and Descending Aorta or Femoral venous and Descending Aorta. The surgery was performed at 26 °C and ***Systemic Potassium was administered into the venous reservoir to arrest the heart****.* Retrograde cerebral perfusion was performed through the Long Femoral venous cannula. Once the Proximal anastomosis is done under RCP, the upper body flow is established by the sidearm of the anteflo graft. The abdominal vessels are perfused by Silicon catheter. The abdominal vessels and renal arteries are anastomosed one after another to the arms of coselli graft. We perfused the renal arteries with renoplegia every 10 min in each renal artery. Once the descending aorta is anastomosed the clamp is removed and rewarmed to 36 °C. Hemostasis secured and came off CPB uneventfully.

**Results**

There was no incidence of any neurological deficit in the post-operative period for all the 12 Patients. Two patients required tracheostomy in the post-operative phase. The Sr. Creatinine was in the desirable range and there was no gut ischemia in the post-operative period.
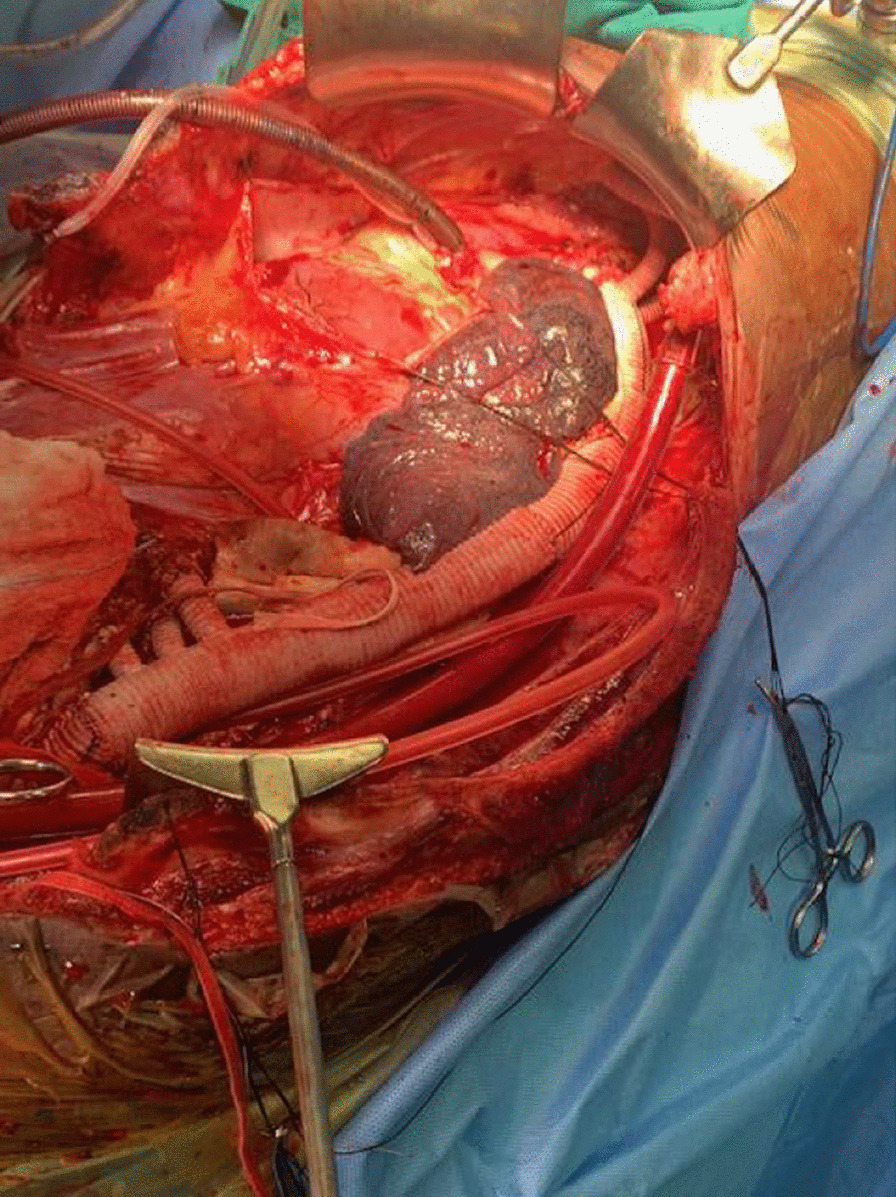


**Conclusion**

Our strategic planning of perfusion techniques for the Thoraco Abdominal Aortic Aneurysm cases resulted in yielding favorable outcome.

### A2 Acute type A Aortic Dissection—A Welsh National Audit

#### Smith, Harry^1^, Dr; Chan, Jeremy^1^, Dr; Mehta, Dheeraj^2^, Mr; Kumar, Pankaj^1^, Mr; Field, Mark^3^, Prof

##### ^1^Morriston Hospital, Swansea, UK; ^2^University Hospital of Wales, Cardiff, UK; ^3^Liverpool Heart and Chest Hospital, Liverpool, UK

*Journal of Cardiothoracic Surgery* 2023, **18(Supp 1)**:A2

**Background**

Acute type A Aortic Dissection (TAAD) is a time-critical, cardiac surgical emergency. Mortality for TAAD increases 1% per hour. Research showed that TAAD was considered in < 50% of patient presented to A&E and 33% were treated for an incorrect diagnosis. We aimed to perform a national audit to evaluate our performance in Wales.

**Methods**

All Welsh patients underwent surgery for TAAD from 2007 to 2019 were identified. A&E notes, CT report, cardiac surgical data base and survival status was evaluated in individual hospital’s data base. The A&E assessment, time of diagnosis (CT) and mortality rate were analysed.

**Results**

99 patients underwent TAAD in all 3 centres during the above period. The median time from assessment to diagnosis was 222 min (Ranged 34–14,424). No statistically significant differences were seen between time of assessment to diagnosis across the three sites (p = 0.07) and to survival status (p = 0.66).

**Conclusion**

Further work is required to raise awareness of TAAD in Wales, delay in diagnosis correlates with increased risk of mortality and this can be mitigated through increasing awareness amongst clinicians throughout Wales.

### A3 High Wall Shear Stress Can Predict Wall Degradation in Ascending Aortic Aneurysms: a Biomechanics Approach to Risk Stratify Disease

#### Salmasi, M Yousuf, Dr; Pirola, Selene, Dr; Sasidharan, Sumesh, Dr; Fisichella, Serena M, Ms; Jarral, Omar A, Dr; O'Regan, Declan, Prof; Moore Jr, James E, Prof; Xu, Xiao Yun, Prof; Athanasiou, Thanos, Prof

##### Imperial College London, UK

*Journal of Cardiothoracic Surgery* 2023, **18(Supp 1)**:A3

**Objective**

Blood flow patterns can alter material properties of ascending thoracic aortic aneurysms (ATAA) via vascular wall remodelling. This study examines the relationship between wall shear stress (WSS) obtained from image-based computational modelling with tissue-derived material properties of the ATAA wall using segmental analysis.

**Methods**

Ten patients undergoing surgery for root or ascending ATAA were recruited. Exclusions: bicuspid aortopathy, connective tissue disease. All patients had pre-operative 4-dimensional flow magnetic resonance imaging (4D-MRI), allowing for patient-specific computational fluid dynamics (CFD) analysis and anatomically precise time-averaged WSS mapping of ATAA regions (12 segments per patient). Aneurysmal aortic samples were obtained from surgery and subjected to region specific tensile failure and peel testing (matched to WSS segments). Computational pathology was used to characterise elastin/collagen abundance and smooth muscle cell (SMC) count. Multilevel hierarchical regression modelling was conducted to analyse the influence of aortic flow on material properties of the aortic wall.

**Results**

Elevated values of time-averaged WSS (TAWSS) were predictive of: reduced wall thickness (coef − 0.0489, 95% CI [− 0.0905, − 0.00727], p = 0.022) and dissection energy function (longitudinal) (− 15,0, 95% CI [− 33.00, − 2.98], p = 0.048). High TAWSS values also predicted higher ultimate tensile strength (coef 0.136, 95% CI, [0 0.001, 0.270], p = 0.048) i.e. increased wall stiffness. Additionally, elevated TAWSS predicted a reduction in elastin levels (coef − 0.276, 95% [CI − 0.531, − 0.020], p = 0.035) and lower SMC count (coef − 6.19, 95% CI [− 11.41, − 0.98], p = 0.021). TAWSS was found to have no effect on collagen abundance or circumferential mechanical properties.

**Conclusions**

Our study identifies a strong association between WSS and aortic wall degradation in ATAA disease. Further studies will help identify its utility in predicting acute aortic events.

### A4 Endovascular Treatment of Ascending Aortic Pathology- A Meta-Analysis

#### Alwis, Shehani^1^, Dr; Mozalbat, David^2^, Mr; Cyclewala, Shabnam^1^, Dr; Metwalli, Amr^3^, Ms; Athansiou, Thanos^3^, Mr; Salmasi, M Yousuf^3^, Mr; Nienabar, Christoph A.^3^, Prof

##### ^1^Barts Health NHS Trust, London, UK; ^2^St George's Hospital, London, UK; ^3^Royal Brompton and Harefield NHS Trust, London, UK

*Journal of Cardiothoracic Surgery* 2023, **18(Supp 1)**:A4

**Objectives**

Open surgical repair is the established gold standard treatment for pathology of the ascending aorta (AA). In recent years, endovascular stenting (TEVAR) of the AA has been attempted, but only in expert centres with a limited understanding of outcomes. This study aimed to systematically review the literature to determine the safety and outcomes of stenting in the ascending aorta.

**Methods**

A systematic literature search was conducted in 5 online databases, incorporating cohort studies and case series of patients undergoing TEVAR for pathology in the AA region. Case reports were excluded Qualitative analysis of patient covariates and outcomes were measured using pooled meta-analysis. Meta-regression was used to assess the influence of covariates on complication rates.

**Results**

Overall, 25 full-text titles were included, encompassing a total of 572 endovascular procedures, the majority of which were elective aneurysm repairs (89%), and 11% acute aortic dissection. Pooled analysis revealed a procedural mortality as 4.19%. The incidence of endoleaks was17.6% and at long-term follow-up 17.1% (98 cases) required reintervention. Neurological complications occurred at a rate of 6.8% which was a combination of major strokes, minor strokes and spinal cord ischaemia.

A meta-regression analysis revealed congestive heart failure as a predictor post-operative endoleak (coef 27.47, 95% CI [6.53, 48.42], p = 0.017). No other variables (age, gender diabetes, PVD, COPD) were shown to be predictive of endoleaks post-operatively. The presence of diabetes as a covariate was found to be a predictor of lower rates of re-intervention (coef − 17.66, 95% CI [− 28.2, − 7.14], p = 0.004).

**Conclusions**

Endovascular repair of ascending aortic aneurysms is a safe alternative to surgery in high-risk patients, although the risks of endoleaks and re-intervention are not negligible. This analysis indicates careful patient selection is needed, especially to ensure a good short-term outcome.

### A5 Impact of Perioperative Sarcopenia on In-hospital Mortality and Spinal Cord Ischaemia Following Open Thoracoabdominal Aortic Aneurysm Repair (TAAA)

#### Simoniuk, Urszula^1^, Ms; Christodoulidou, Michelle^2^, Miss; Ntouskou, Marousa^3^, Miss; Shaw, Mathew^3^, Mr; Richards, Toby^2^, Mr; Muneer, Asif^4^, Mr; Kuduvalli, Manoj^3^, Mr; Field, Mark^3^, Prof; Theologu, Thomas^3^, Mr; Y Oo, Aung^1^, Prof

##### ^1^St Bartholomew's Hospital, London, UK; ^2^University College London, Division of Surgery and Interventional Sciences, London, UK; ^3^Liverpool Heart and Chest Hospital, Liverpool, UK; ^4^NIHR Biomedical Research Centre UCLH and Division of Surgery and Interventional Science UCL, London, UK

*Journal of Cardiothoracic Surgery* 2023, **18(Supp 1)**:A5

**Table:** 

Table 1. Cohort Mortality	Total number of the patients (n-88)	Male (n-47) p value	Female (n-41) p value
Age		0.035 (U = 81.500)	0.054
Hypertension	0.81	0.026 OR = 0.590 CI 95% [0.45–0.766]	0.46
Sarcopenia	48(54.5%)	0.54	0.016 OR = 10.9 CI 95% [1.135–104.807]
TAAA Extent V	1 (6.7%)	0.02 OR = 1.14 CI 95% [0.88–1.48]	0.54
Stroke	4 (26.7%)	0.049 OR = 5.25 CI 95% [0.89–30.7]	0.34
Re-operation	4 (26.7%)	0.23	< 0.0001 OR = 33 CI 95% [3.59–303]
Haemofiltration	10(66.7%)	0.29	0.035 OR = 8.46 CI 95% [0.88–80.5]
Spinal Cord Ischaemia	5 (33%)	0.85	< 0.001 OR = 21.3 CI 95% [2.69–168.9]
Paraplegia	4 (26.7%)	0.51	< 0.0001 OR = 68 CI 95% [4.97–928.89]

**Objective**

Sarcopenia is defined as loss of skeletal mass making it a quantifiable marker for frailty. We aim to evaluate whether sarcopenia may predict postoperative outcomes like spinal cord ischaemia and mortality following open TAAA.

**Methods**

Between 2008–2017, we identified 88 out of 232 patients who underwent open TAAA repair with available preoperative imaging for sarcopenia evaluation. Sarcopenia was defined by the skeletal muscle index using specialised computer software and CT imaging. To reduce reporting bias, we analysed male and female subgroups, as gender is known to cause differences in body composition.

**Results**

**Female**

In-hospital mortality in sarcopenic patients was significantly higher compared to non-sarcopenic group following the surgery (p = 0.013). Additionally, univariate analysis revealed that sarcopenia (p = 0.016), spinal cord ischaemia (p < 0.001), paraplegia (p < 0.0001), redo-surgery (p < 0.0001), and post-op haemofiltration (p = 0.035) influenced hospital mortality. Following multivariate analysis, haemofiltration and sarcopenia remained independently predictors of mortality (95%CI;p < 0.05).

**Male**

Univariate analysis identified age, hypertension, and postoperative stroke as statistically significant with in-hospital mortality. In-hospital mortality in the sarcopenic vs non-sarcopenic group was higher but not significant (p = 0.55).

**Conclusions**

Sarcopenia correlates with poor survival outcomes and higher in-hospital mortality in female patients compared to non-sarcopenic group. It has the potential of becoming a valuable tool in assessing preoperative frailty to assist in perioperative risk stratification of aortic patients. This is the first study evaluating sarcopenia in extensive TAAA repair, and further studies in a larger group are required to assess the full impact of this frailty marker on patient outcomes.

### A6 Identification of High-risk Cases Through Micromechanical Characterisation of Aneurysmal Aortic Tissues

#### Hossack, Martin^1^, Mr; Fisher, Robert^1^, Prof; Torella, Francesco^1^, Prof; Field, Mark^2^, Mr; Madine, Jillian^3^, Dr; Akhtar, Riaz^4^, Dr

##### ^1^Liverpool University Hospitals NHS Foundation Trust, Liverpool, UK; ^2^Liverpool Heart and Chest Hospital NHS Foundation Trust, Liverpool, UK; ^3^Institute of Systems, Molecular and Integrative Biology, University of Liverpool, Liverpool, UK; ^4^Mechanical, Materials and Aerospace Engineering, University of Liverpool, Liverpool, UK

*Journal of Cardiothoracic Surgery* 2023, **18(Supp 1)**:A6

**Objectives**

Use of a maximum diameter threshold as the sole indicator for aneurysm repair risks rupture during surveillance in higher-risk cases, and unnecessary repair in others. Here, we utilise nanoindentation, a high-resolution technique capable of measuring the material properties of vascular tissue non-destructively at an appropriate length-scale. This study aims to characterise the micromechanical properties of aneurysmal aortic tissue to personalise rupture risk and direct specific management.

**Methods**

Full thickness aortic wall tissue samples were harvested from 8 patients undergoing repair of thoracoabdominal aneurysm (n = 1), aneurysmal dilatation of chronic aortic dissection (n = 1), asymptomatic (n = 3) and symptomatic (n = 2) abdominal aortic aneurysm, and stent explantation after failed endovascular repair (n = 1). We probed the micromechanical properties using nanoindentation with a 100 mm flat punch tip, determining the shear storage modulus (G′). We performed 4–5 indentations in axial orientation on cross-sectional wall samples in 3 layers (inner, middle, outer) where possible. 9–10 samples were tested from each patient. In total, there were 89 samples (962 indentations).

**Results**

All tissues demonstrated a pattern of reducing stiffness from the luminal to abluminal edge (median 30.6 kPa vs 12.9 kPa, P < 0.05), likely a consequence of atherosclerosis affecting the intima. Symptomatic aneurysms were stiffer than asymptomatic (median 20.9 kPa vs 16.1 kPa, P < 0.05), whilst tissue from the explantation subgroup demonstrated significantly higher stiffness (median 41.6 kPa) than all others, which were not significantly different (Fig. 1).

**Conclusions**

This micromechanical approach may distinguish between higher risk (symptomatic) and lower risk (asymptomatic) aneurysms. Aortic wall micro-stiffness may be an indicator of high-risk aneurysm. Further studies are needed to confirm the findings and correlate aortic stiffness with clinical and radiological presentation.
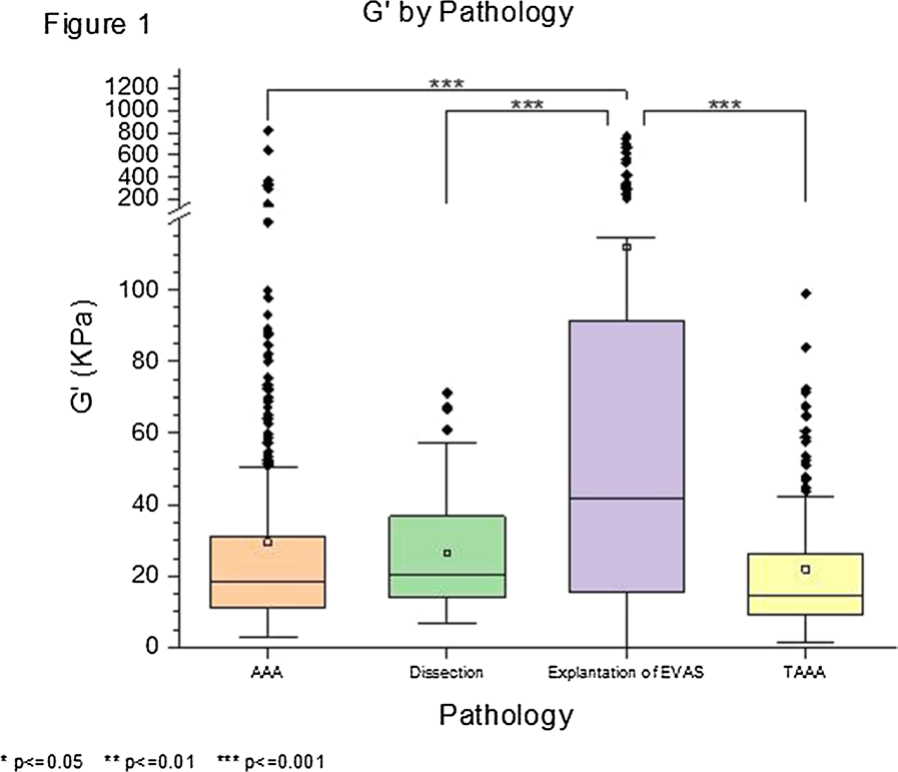


### A7 The Elephant in the Room, Does Frozen Elephant Trunk Offer Better Outcomes in Type A Aortic Dissection? A Comparative Study in a Single Centre

#### Moawad, Nader, Mr; Sinha, Shubhra, Miss; Harfield, Jack, Mr; Villaquiran, Jaime, Mr; Villaquiran, Christopher, Mr; Wali, Anuj, Mr; Unsworth-White, Jonathan, Mr; Kuo, James, Mr

##### Derriford Hospital, Plymouth, UK

*Journal of Cardiothoracic Surgery* 2023, **18(Supp 1)**:A7

**Objectives**

To compare the outcomes of hybrid stented graft (Frozen elephant trunk) versus other operative strategies in surgical repair of acute aortic dissection.

**Methods**

Single centre retrospective analysis of prospectively collected data for patients undergoing repair of acute type A Aortic dissection repair between April 2012 and October 2021 (N: 181). They were divided into two groups; Group A had isolated Ascending Aorta replacement +/− extension to Aortic arch (N: 152), Group B had FET (N: 29).

**Results**

Pre-operative characteristics were comparable between the two groups. Cardiopulmonary bypass times and cross-clamp times were shorter in Group A. However, when comparing Arch replacement case cases from Group A with Group B, the times were comparable. There was no marked difference in reoperation for bleeding, dialysis nor sepsis between both groups. The incidence of temporary neurologic dysfunction was slightly higher in group B (24% vs 17%). The 30-day mortality was lower in Group B (13.8% Vs 20.4%) but this did not reach statistical significance due to the small sample size. At median follow-up of 3 years there was a trend towards improved survival in Group B.

**Conclusion**

Frozen Elephant trunk offers good early results and comparable operative times to other surgical techniques. We believe that it should be considered as first-line therapy in experienced centres.
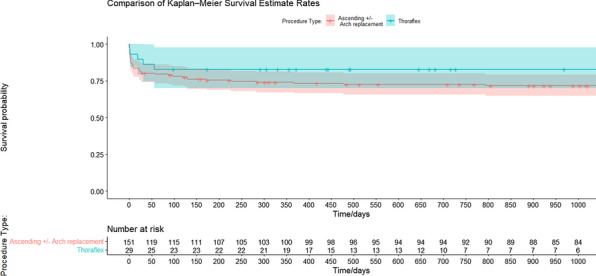


### A8 Subacute Dissection—Saturday Night TEVAR

#### Muston, Benjamin^1^, Mr; Guo, Allen^1^, Mr; Sahai, Prachi^2^, Ms; Wilson-Smith, Ashley^3^, Dr

##### ^1^University of New South Wales, Sydney, Australia; ^2^University of Newcastle, New South Wales, Australia; ^3^Chris O’Brien Lifehouse Center, Sydney, Australia

*Journal of Cardiothoracic Surgery* 2023, **18(Supp 1)**:A8

**Objectives**

While there has been discussion in the field for some time regarding the preference of medical management, open surgery or minimally invasive intervention for the treatment of aortic dissection, the subacute population is an upcoming and relatively unknown cohort. This systematic review and meta-analysis provides a complete aggregation of reported long-term survival and freedom from reintervention of subacute complicated Type B aortic dissection patients based on the existing literature.

**Methods**

Three online databases (Embase, Medline, Scopus) were searched from date of inception until June 2021, accruing a total of 2580 references which were reviewed by three independent authors. The primary endpoints were survival and freedom from reintervention, whilst secondary endpoints were post-operative outcomes, such as technical success and endoleak. Kaplan–Meier curves were digitized and aggregated to graph estimated survival data.

**Results**

Sixteen studies were selected according to our criteria, yielding 365 patients with a mean age of 59.1 ± 6.0 years. The ‘subacute’ cohort had a mean time from symptom onset to diagnosis of 21.0 ± 2.6 days, differentiating it from both acute and chronic dissection definitions. Overall survival at 1, 3, and 5 years was 85.7%, 73.9% and 71.2%, respectively. Freedom from reintervention at 1, 3 and 5 years was 88.0%, 78.0% and 73.1%, respectively.

**Conclusions**

This systematic review and meta-analysis described the first aggregated survival data for a subacute dissection cohort receiving thoracic endovascular aortic repair to date. TEVAR is associated with promising long-term outcomes to 5-years, with high rates of technical success, though more data is needed to make true comparisons to acute/chronic cohorts. Randomized controlled trials involving TEVAR for aortic dissection with varying chronicity is required for progression in this field.
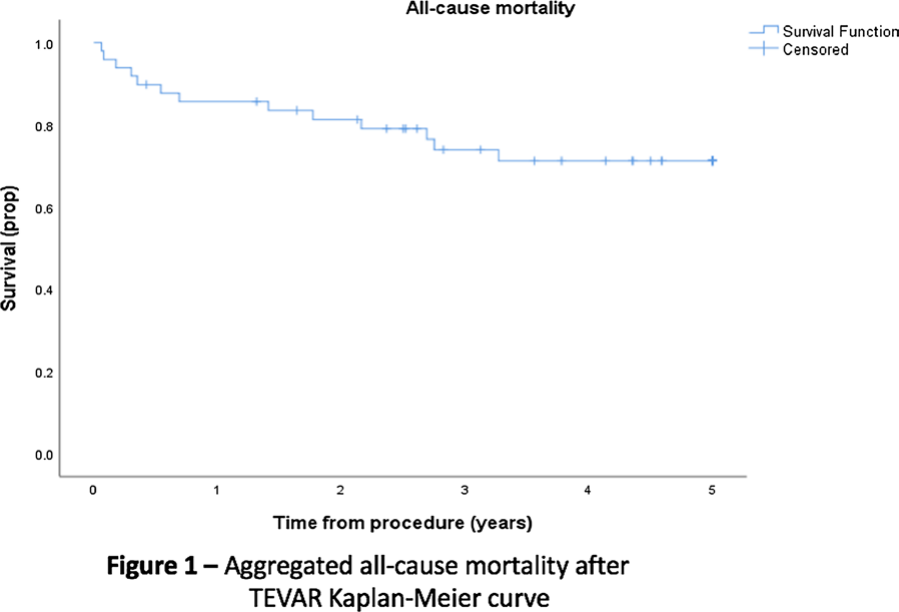


### A9 Type-I Aortic Dissection: The Fate of Distal Aorta Following Limited to the Ascending Aorta Repair

#### Verdichizzo, Danilo, Mr; Braithwaite, Simon, Dr; Kemp, Ben, Dr; Kearns, Daniel, Dr; D'Alessio, Andrea, Mr; Ttofi, Iakovos, Mr; Power, Harvinder, Dr; Keiralla, Amar, Dr; Uberoi, Raman, Dr; Krasopoulos, George, Mr

##### John Radcliffe Hospital, Oxford, UK

*Journal of Cardiothoracic Surgery* 2023, **18(Supp 1)**:A9

**Objectives**

The aim of this study was to assess the remodelling of distal aorta after emergency limited to ascending aorta repair of acute De Bakey type I aortic dissection.

**Methods**

55 surviving patients (2011–2019) were enrolled. All patients had pre-operative computed tomography (CT) scan and at least one follow-up CT. Intramural haematomas and retrograde dissections were excluded. The repair was limited to the ascending aorta (± aortic root), with open distal anastomosis at Zone-0. Longitudinal study analysis was applied for unbalanced data. Mixed effect linear regression model with random intercept and random slopes was used.

**Results**

Median age 59 (IQR:52–66), 39 (71%) male; Log-EuroSCORE 21.10(12.35–21.90); cardiopulmonary bypass 175 min (146–197), cross clamp 84 min (59–120), circulatory arrest 23 min (20–31), length of stay 11 days(IQR 7–18), follow-up 39 months (29–73).

Presence of residual patent false lumen (pFL) has a significant adverse impact on the remodelling to the aortic arch with fixed effect related growth of 4.42 mm/year (inter-individual variance 3.87 mm/year, p = 0.05) and 4.64 mm/year for descending thoracic aorta (individual variance 4.32 mm/year, p = 0.03).

The aortic dilatation adverse remodelling was not associated with increased mortality.

**Conclusions**

Limited to the ascending aorta surgical repair of a Type-I aortic dissection with pFL is a life-saving procedure that leads into a time related negative remodelling and possible need for further interventions.
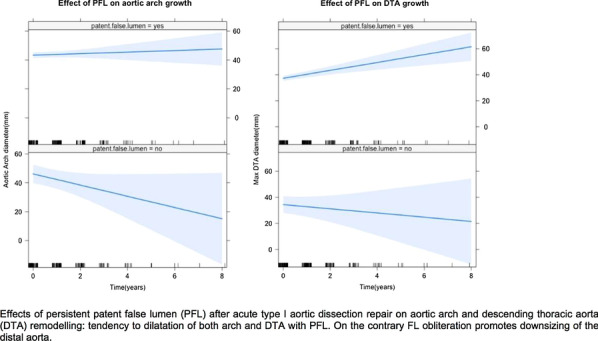


### A10 Is Rural Status Associated with Adverse Outcomes in Patients Undergoing Surgery for an Acute Type A Aortic Dissection: A Western Australian Study

#### Eranki, Aditya, Dr

##### John Hunter Hospital, Newcastle, Australia

*Journal of Cardiothoracic Surgery* 2023, **18(Supp 1)**:A10

**Introduction**

Acute Type A Aortic Dissection (ATAAD) represents a cardiothoracic emergency and should be managed without delay. Western Australia is an expansive state of 2.5 million square kilometeres with a network of regional centres and cardiothoracic centres located in the capital Perth. The aim of this study is to assess the differences in presentation and outcomes between rural and urban patients undergoing emergent repair for an ATAAD.

**Methods**

We performed a retrospective analysis of all patients undergoing emergent repair of an ATAAD at Fiona Stanley Hospital between 2015 to 2020. A number of variables associated with the patient’s geography, physiology, and postoperative outcome were assessed. Univariate and multivariate logistical regression analysis was performed to evaluate significant differences between the cohorts.

**Results**

A total of 64 patients underwent emergent repair of an ATAAD between 2015–2020. The overall 30-day mortality rate was 20%. The 30-day mortality rate in the rural cohort was higher than the urban cohort (30% vs 16%) however this was not statistically significant. The rural cohort faced a higher median time to surgery (12 vs 6 h, P = 0.002) and are likely to require transfer between more than two centres (7 vs 1, P = 0.001). Rural patients were also more likely to require resuscitation perioperatively (OR 4.33, P = 0.013) and more likely to require return to theatre postoperatively (OR 7.60, P = 0.002). Both return to theatre and multi-hospital transfer were associated with rural status in multivariate analysis.

**Conclusion**

Rural patients wait twice as long for definitive surgery as their urban counterparts and require multi-hospital transfer. Rural patients are more likely to present in a critical peri-operative state requiring resuscitation, and have a significantly higher risk of returning to theatre post operatively.

### A11 Risk Factors for Haemofiltration Following Surgery on the Descending Thoracic and Thoracoabdominal Aorta

#### Bennett, James, Mr; Field, Mark, Prof; Kuduvalli, Manoj, Mr; Doonan, Robert, Mr; Shaw, Matthew, Mr

##### Liverpool Heart and Chest Hospital, Liverpool, UK

*Journal of Cardiothoracic Surgery* 2023, **18(Supp 1)**:A11

**Objectives**

Open surgical repair of the thoracoabdominal aorta (TAA) and descending thoracic aorta (DTA) carries significant morbidity risk. We sought to identify risk factors for haemofiltration (HF) requirement after TAA and DTA surgery.

**Methods**

Retrospective analysis was conducted using characteristics of 491 patients undergoing TAA or DTA repair from October 1998 to October 2021 at a single institution. HF by perfusion modality was assessed in 346 patients. Univariate and multivariate analyses were performed to identify predictors of post-operative HF.

**Results**

New post-operative HF was required in 66 (13.4%) patients. HF was associated with increased age (median age = 63 vs 66; p = 0.03) and lower pre-operative estimated glomerular filtration rate (eGFR) (median 76 vs 68; p = 0.01). Patients who required TAA Extent II (p < 0.001) or III (p = 0.01) repair were more likely to require HF.

For TAA repair, HF was required in 35 (21.6%) patients when left heart bypass (LHB) was used and 6 (16.2%) patients when deep hypothermic circulatory arrest (DHCA) was used. For DTA repair, HF was required in 2 (4.8%) patients when LHB was used and 2 (4.2%) of patients when DHCA was used.

Need for HF was associated with in-hospital mortality (28.8% vs. 9.9%, p < 0.001), stroke, paraplegia, and need for reoperation. HF was associated with longer ICU stay (p < 0.001).

Multivariable analysis revealed increasing age (OR (95% CI) = 1.03 (1.01, 1.06); p = 0.005), body mass index ≥ 25 (1.86 (1.03, 3.36); p = 0.039 and Extent II or Extent III surgery (4.83 (2.71, 8.63); p < 0.001) were associated with post-operative HF.

**Conclusions**

Age, baseline renal function, BMI, and extent II/III operations were associated with post-operative HF in TAA and DTA surgery. Need for post-operative HF was also associated with complications such as death, stroke, paraplegia, need for reoperation and extended length of stay.

**Table:** 

Aortic Presentation	No Post-op HF (n = 425)	Post-op HF (n = 66)	P value
DTA Type A	41 (9.7)	2 (3.0)	0.08
DTA Type B	16 (3.8)	2 (3.0)	> 0.99
DTA Type C	91 (21.4)	4 (6.1)	0.003
TAA Extent I	62 (14.6)	7 (10.6)	0.39
TAA Extent II	122 (28.7)	35 (53.0)	< 0.001
TAA Extent III	31 (7.3)	11 (16.7)	0.01
TAA Extent IV	23 (5.4)	3 (4.6)	> 0.99
TAA Extent V	12 (2.8)	2 (3.0)	> 0.99
Data N/A	27 (6.4)	0 (0)	0.04

### A12 Patient Reported Outcome Measures in Patients Undergoing Proximal Aortic Surgery for Aneurysmal Disease – A Pilot Study

#### Shaw, Matthew^1^, Dr; Salem, Agni^2^, Miss; Day, Jennie^3^, Dr; Oo, Aung^4^, Prof; Field, Mark^2^, Prof; Haycox, Alan^3^, Dr; Rowe, Michael^3^, Dr

##### ^1^No affiliation; ^2^Liverpool Heart and Chest Hospital, Liverpool, UK; ^3^University of Liverpool, Liverpool, UK; ^4^Barts Health NHS Trust, London, UK

*Journal of Cardiothoracic Surgery* 2023, **18(Supp 1)**:A12

**Objectives**

Surgical interventions to treat aortic aneurysms are relatively invasive and can impact greatly on well-being, particularly as patients are often asymptomatic. Tracking post-operative health-related quality of life (HRQOL) is problematic as patient reported outcome measures (PROMs) have not yet been specifically designed for this disease. This study aims to assess the feasibility of PROM delivery and HRQOL reporting in this patient cohort.

**Methods**

Suitable patients scheduled for elective aortic surgery at Liverpool Heart and Chest Hospital between October 2017 and March 2019 were identified and invited to participate in the pilot study. Patients were asked to complete the PROM prior to surgery and then at 6 weeks and 3 months after their operation. The PROM items were arranged into four domains: symptoms, physical, psychosocial and cognitive. The newly developed instrument underwent preliminary testing for its appropriateness, acceptability, feasibility, interpretability, precision, reliability and responsiveness.

**Results**

In total, 30 patients completed all 3 questionnaires. Initial testing showed that the newly developed instrument performed to acceptable standards. It showed good internal consistency (Cronbach’s alpha results for all domains > 0.85), and test retest reliability (intraclass correlation coefficient for all domains > 0.85). In paired sample tests, the values in each domain led to statistically significant differences from baseline at either 6 weeks or 3 months (p < 0.05), supporting the construct validity and responsiveness of the instrument.

**Conclusions**

The PROM pilot questionnaire demonstrated satisfactory validity as well as good internal reliability and test–retest reliability for each item across all four domains. The PROM identified a negative impact of a diagnosis of aneurysm on all domains and a positive influence of surgery.

### A13 The Risk of Right Ventricular Outflow Tract Breach or Rupture in Patients Undergoing Aortic Valve Sparing Procedures

#### Abdul Hakeem, Muhammad, Mr; Shaw, Matthew, Mr; Kenawy, Ayman, Mr; Othman, Ahmed, Mr; Harrington, Deborah, Ms; Kuduvalli, Manoj, Mr; Field, Mark, Prof; Nawaytou, Omar, Mr

##### Liverpool Heart and Chest Hospital, Liverpool, UK

*Journal of Cardiothoracic Surgery* 2023, **18(Supp 1)**:A13

**Objective**

Deep anterior dissection (DAD) separating the RVOT from the interventricular septum may be necessary in some cases of aortic valve and root repair to reach below the level of the basal ring anteriorly.

This may help support the aortic annulus, and prevent late incompetence. However, this technique may breach the RVOT. This study aims to identify patients at higher risk of having this complication.

**Methods**

We included all patients having valve preserving aortic root surgery with either David's procedure, or complete external ring annuloplasty, from November 2017 to September 2021, in our centre.

We identified patients in which DAD was needed to reach below the basal ring. We also identified patients in which the RVOT was breached. We used logistic regression to identify predictors for these outcomes.

**Results**

147 patients were included. Mean age was 51 years. DAD was required in 93 patients (63%).

Mean pre-operative annular diameter was 26.5 mm, mean diameter at the sinuses of Valsalva was 48 mm. 41 patients (28%) had bicuspid aortic valves (BAV). 11 patients were re-explored for bleeding, 8 of which had had DAD, one patient was to repair an RVOT breach. Predictors for DAD were age (OR 0.94), connective tissue disorders (OR 15.9), BAV (OR 8.9) and preoperative annulus diameter (OR 1.2), on multivariable logistic regression. Anterior leaflet prolapse was the only predictor of RVOT breach (OR 3). All patients left the operating theatre with aortic incompetence of 1/4 or less.

**Conclusion**

DAD is safe when used to augment annular support. Younger age, connective tissue disorders, wider annuli and BAV required DAD to reach below the basal anterior ring, as these patients have a more extensive anterior annular dilation into the septum. Caution is needed in patients with anterior leaflet prolapse, as they show extreme anterior annular degeneration, and are prone to RVOT breach. Further follow-up is needed to assess the role of DAD in maintaining aortic valve competence.

### A14 Predictors of Permanent Pacemaker Implantation Following Valve Preserving Aortic Root Procedures

#### Abdul Hakeem, Muhammad, Mr; Rao, Archana, Dr; Kenawy, Ayman, Mr; Othman, Ahmed, Mr; Harrington, Deborah, Ms; Kuduvalli, Manoj, Mr; Field, Mark, Prof; Nawaytou, Omar, Mr

##### Liverpool Heart and Chest Hospital, Liverpool, UK

*Journal of Cardiothoracic Surgery* 2023, **18(Supp 1)**:A14

**Objective**

Unlike valve replacements, data regarding the need for permanent pacemaker (PPM) following valve preserving root surgery has not been adequately investigated. This study aims at detecting predictors of PPM implantation following these procedures.

**Methods**

We included patients who had valve preserving root surgery, with either David's Procedure, or a complete external annuloplasty, at our centre, between November 2017 and April 2021. We excluded intraoperative conversions to valve replacement, and those who had only subcommisural annuloplasty or lone leaflet repair.

Decision for PPM was based on significant conduction abnormality lasting at least 4 days postoperatively, after consulting electrophysiology team.

**Results**

120 patients were included. Mean age was 52 years.

David's procedure was done in 114 patients (95%), 6 patients had external annuloplasty.

9 patients (7.5%) had a PPM, of which 3 had preoperative first-degree AV block.

33 patients had bicuspid aortic valves (28%).

65% of patients had significant preoperative aortic incompetence, and 46% had preoperative annular dilatation (> 26 mm).

Significant predictors of PPM implantation were mild or moderate LV systolic dysfunction (OR 7.2 and 8.6, p-value 0.04 and 0.02, respectively), preoperative aortic annular diameter (OR 1.19, p-value 0.04) and preoperative LVESD (OR 1.09%, p-value 0.02).

PPM was inserted between post-operative day 4 and 14 (median day 9). At 3 months after insertion, 7 out of 9 patients had > 90% pacing. One patient had significant LV dysfunction with RV pacing.

**Conclusion**

PPMs remain a problem in valve preserving aortic root surgery.

We employ deep anterior dissection, separating RVOT from the muscular interventricular septum, this may explain our higher rate of PPMs, in addition to the fact that 18% of our patients had preoperative conduction abnormalities.

Caution has to be employed in patients with a dilated annulus, as they are at risk for conduction disorders.

### A15 Organ Protection Strategies in Thoracoabdominal Aortic Aneurysm (TAAA) Repair – A Single Centre Experience

#### Simoniuk, Urszula, Ms; Naruka, Vinci, Mr; Mangel, Tobin, Miss; Lopez-Marco, Ana, Miss; Adams, Benjamin, Mr; Mastracci, Tara, Miss; Oo, Aung, Prof

##### St Bartholomew's Hospital, London, UK

*Journal of Cardiothoracic Surgery* 2023, **18(Supp 1)**:A15

**Objective**

Organ ischaemia following TAAA repair is associated with significant comorbidities. The incidence varies depending on the aortic centre practice: spinal cord ischaemia (15–20%), stroke (8.1–10%) or renal impairment (20%). We aim to determine the outcomes and effectiveness of organ protection techniques during TAAA repair.

**Methods**

Retrospective analysis of 88 patients who underwent open (75) and endovascular (13) TAAA repair between 2017–2021. The cohort contained: Crawford extent I (9), II (55), III (12), IV (12) TAAA repair, of which 23.9% of procedures were non-elective. We evaluated intraoperative methods of spinal cord protection based on local protocol. The renal protection strategies included blood or cold crystalloid perfusion, and cerebral protection was monitored using cerebral near-infrared spectroscopy (NIRS).

**Results**

Overall mortality in the analysed cohort was 21.6%, including 17% in the Crawford extent II subgroup.12.5% of patients developed spinal cord ischaemia post-operation. 14,8% of patients sustained renal impairment. Spinal cord ischaemia (p = 0.039) and haemofiltration (p = 0.0062) significantly influenced cohort mortality. 8% of patients developed stroke. The spinal cord protection protocol incorporated: cerebrospinal fluid drainage 81(92%), left heart bypass 66 (75%), intercostal arteries reimplantation 49 (55.7%), motor evoked potential monitoring 61 (69.3%), controlled hypertension, Hb > 100 and paraspinal NIRS. The renal perfusion was protected using cold crystalloid 34(38.6%) or blood 6(29.5%). Cerebral perfusion was monitored in all patients.

**Conclusion**

The organ protection programme's introduction helps decrease significant comorbidities like spinal cord ischaemia, renal impairment or stroke associated with TAAA repair. It is an essential component of the strategy for preventing ischaemia complications and improving postoperative complications.

### A16 The Acute Aortic Dissection Pathway—Reviewing the Referral Process to a Tertiary Centre and Impact on Operative Mortality and Complications

#### Ashraf, Muhammad Arsalan^1^, Mr; Mcgurk, Catherine^2^, Miss; Salmasi, Mohammad Yousuf^3^, Mr; Zargaran, David^1^, Mr; El-Hilly, Abdulrahman^1^, Mr; Jarral, Omar^1^, Mr; Baig, Kamran^1^, Mr; Sabetai, Michael^1^, Mr

##### ^1^Guy's and St Thomas' NHS Foundation Trust, London, UK; ^2^King's College London, London, UK; ^3^Department of Surgery, Imperial College London, London, UK

*Journal of Cardiothoracic Surgery* 2023, **18(Supp 1)**:A16

**Objectives**

Acute Type A Aortic Dissections (AAD) often require transfer to a tertiary centre for surgical management. There are no universal transfer guidelines for these patients and little is known regarding impact on patient outcome. We aim to determine transfer timings from onset of symptoms to knife to skin, the extent of medical optimisation during transfer, and the impact on operative mortality.

**Methods**

A retrospective analysis of 118 patients referred to St. Thomas’ Hospital from Southeast England for emergency surgical repair of AAD between 2014–2020. Notes were evaluated to identify timings of each step of the referral process and optimisation of patients. Regression analysis was performed to determine impact of these on outcome.

**Results**

Mean time from onset of symptoms to incision was 22.1 h. Mean time to diagnosis following admission was 8.65 h, from referral to incision 7.6 h and in transit was 1.02 h. During transit, 49% had blood pressure control with an infusion, 45% had an arterial line, 31% catheterized and 35% were accompanied by a medical escort. 51% were misdiagnosed on initial presentation. Linear regression analysis found misdiagnosis to be a strong predictor of patient delay to the operating theatre (coef 841, 95% CI [36—1646], p-0.041). However, neither misdiagnosis nor patient delay were related to operative mortality, stroke or renal failure (p > 0.05). Logistic regression found malperfusion pre-op to be a strong predictor of patient death (OR 6.7, 95% CI [1.7—26.0, p = 0.006).

**Conclusion**

This cohort is the first evaluation of the timings and variability in medical optimisation during transfer of patients with AAD. Our results show that misdiagnosis is a significant cause for delay in in overall transfer time. Malperfusion was the only statistically significant predictor of mortality immediately post-op. Future work will evaluate survival outcomes related to the transfer timings and formulate a transfer optimisation guideline.

### A17 In-hospital and Long-term Outcomes of Surgery in Patients with Acute Type A Aortic Dissection: A 15-year Experience

#### Jos, Helena, Ms; Wicks, William, Mr; Hamid, Umar, Mr; Awad, Wael IMr

##### Barts Health NHS Trust, London, UK

*Journal of Cardiothoracic Surgery* 2023, **18(Supp 1)**:A17

**Objectives**

To evaluate long-term outcomes of patients with acute type A aortic dissection (ATAAD) undergoing surgical repair at our centre over a 15-year period.

**Methods**

Patient demographics, operative details and post-operative outcomes were analysed for the period of January 2005 to September 2020. This period was divided into First Half (2005–2012) and Second Half (2012–2020) to assess trends. Kaplan–Meier curves were constructed to establish long-term survival.

**Results**

A total of 323 patients underwent ATAAD repair during this period. The mean age of the cohort was 59.2 ± 15.3 years, 222 patients out of 323 (68.7%) were male; 24 out of 323 (7.4%) had previous cardiac surgery. There were significant changes between First Half and Second Half periods including number of cases performed (88/323 versus 235/323, a 167% increase); number of patients undergoing surgery within 24 h of presentation (22/70 (31.4%) versus 126/227 (55.5%), *P* = 0.0004); in-hospital mortality (16/88 (18.2%) versus 60/235 (25.5%), *P* = 0.1656); ICU stay > 30 days (1/60 (1.7%) versus 23/212 (10.8%), *P* = 0.0268); post-operative haemofiltration requirement (2/72 (2.8%) versus 25/222 (11.3%), *P* = 0.0303) and any post-operative cerebrovascular event (15/72 (20.8%) versus 85/222 (38.3%, *P* = 0.0066). The overall 5-year survival was 67%, 10-year survival was 55% and 15-year survival was 38% (Fig. 1), with no significant difference in survival between the two groups.

**Conclusions**

The number of ATAAD repair procedures performed increased significantly throughout our study period. Patients have a more complicated post-operative recovery and both early and late outcomes remain high.

Figure 1. Kaplan–Meier curve illustrating long-term survival of patients.
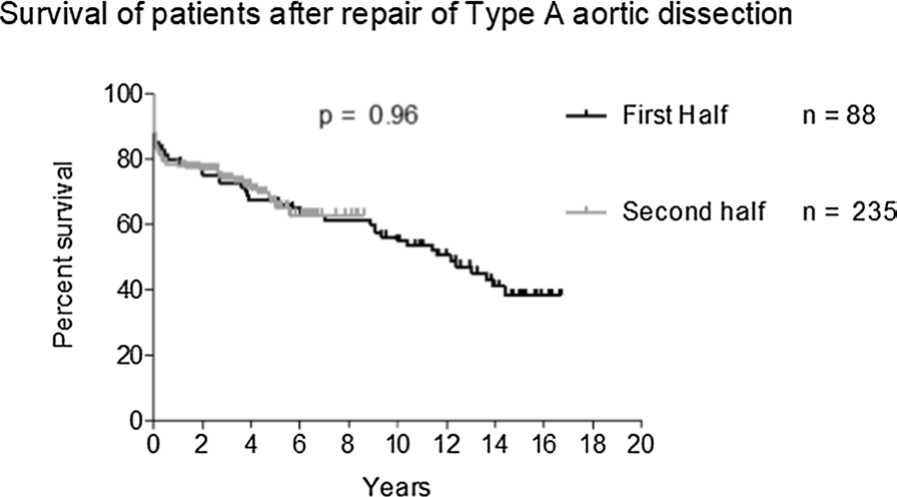


## Adult Cardiac Aortic Valve

### A18 Infected TEVAR Explantation, Descending Thoracic Aorta Repair & Repair of Aorto-Oesophageal Fistula

#### Shehata, Monicka, Dr; Rizzo, Victoria, Miss; Price, Nicholas, Dr; Chawla, Amit, Dr; Sallam, Morad, Mr; Sabetai, Michael, Mr

##### Guy's & St Thomas' NHS Foundation Trust, London, UK

*Journal of Cardiothoracic Surgery* 2023, **18(Supp 1)**:A18

**Large**

**Objectives**

This study sought to compare the morbidity and mortality of redo Aortic Valve Replacement (redo-AVR) versus valve-in-valve Transcatheter Aortic Valve Implantation (valve-in-valve TAVI) for patients with a failing bioprosthetic valve.

**Methods**

A multicentre UK retrospective study of redo-AVR or valve-in-valve TAVI for patients referred for redo aortic valve intervention due to a degenerated aortic bioprosthesis. Logistic regression coefficients were used for propensity score matching with a tolerance of 0.01.

**Results**

From July 2005 to April 2021, 911 patients underwent redo-AVR and 411 patients valve-in-valve TAVI. There were 125 pairs for analysis after propensity score matching. In-hospital mortality was 7.2% (n = 9) for redo-AVR vs 0 for valve-in-valve TAVI, p = 0.002. Long-term mortality was 30.4% (n = 38) vs 15.2% (n = 19), p = 0.004, at 3.30 ± 3.29 years follow-up. Redo-AVR had worse survival at every moment in time (Kaplan–Meier, p = 0.02). Surgical patients suffered more post-operative complications, including IABP support (p = 0.02), early re-operation (p < 0.001), arrhythmias (p < 0.001), respiratory and neurological complications (p = 0.02 and p = 0.03) and multiple organ failure (p = 0.01). The valve-in-valve TAVI group reported a shorter intensive care unit and hospital stay (p < 0.001 for both). Finally, the degree of aortic regurgitation at discharge was significantly higher in the percutaneous approach (p < 0.001).

**Conclusion**

Valve-in-valve transcatheter aortic valve implantation, as opposed to redo surgical aortic valve replacement with a biological prosthesis, appears to be the best treatment option for elderly patients with a degenerated bioprosthetic valve.
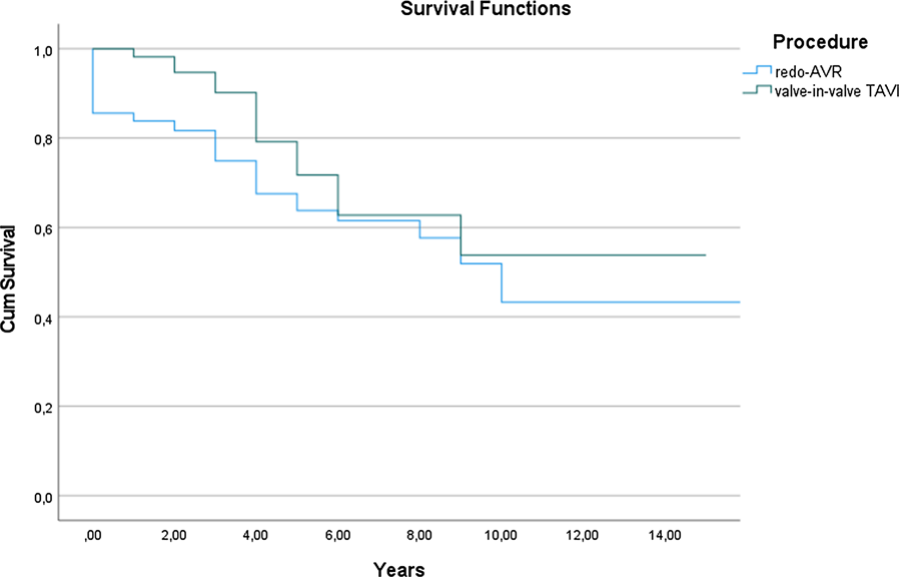


### A19 Perioperative Outcomes and Long Term Survival of Octogenarian Patients Undergoing Re-sternotomy for Aortic Valve Replacement

#### Masraf, Hannah^1^, Miss; G Malvindi, Pietro^2^, Mr; Luthra, Suvitesh^2^, Mr; Ohri, Sunil K^2^, Prof

##### ^1^University of Southampton, Southampton, UK; ^2^University Hospital Southampton, Southampton, UK

*Journal of Cardiothoracic Surgery* 2023, **18(Supp 1)**:A19

**Objectives**

Little data on the safety of re-sternotomy for surgical aortic valve replacement (SAVR) in octogenarians with prior surgery exists. Our study aims to analyse the perioperative results and long-term survival outcomes of re-sternotomy for SAVR in octogenarians.

**Methods**

This is a retrospective, single-centre study (Apr 2000–Dec 2019). Perioperative data were compared for re-sternotomy with isolated SAVR and re-sternotomy with SAVR and associated cardiac procedure(s). Uni- and multivariable logistic regression analyses were performed to identify predictors of inpatient mortality. Cox regression was used to calculate hazard ratios (HRs) for covariates of long-term survival and Kaplan Meier survival curves were compared for groups.

**Results**

There were 163 patients (Isolated redoSAVR; 69, Associated redoSAVR; 94). The median age was 83 (81–85) years and the median logistic EuroSCORE was 19.2% (13.0–26.7%). The mean follow-up period was 4.2 ± 3.5 years. Inpatient mortality was 4.9% (1.4% versus 7.4% for Isolated redoSAVR and Associated redoSAVR respectively, p = 0.08). Demographics, operative data and postoperative results were broadly comparable between both groups. Multivariable logistic regression identified COPD as a significant predictor of inpatient mortality (OR 8.86 95%CI: 1.19, 66.11, p = 0.03). Overall survival was 88.7% at 1 year, 86.4% at 2 years, 70.1% at 5 years, 49.5% at 7 years and 26.3% at 10 years. There was no survival difference between Isolated redoSAVR and Associated redoSAVR (logrank p = 0.36, Wilcoxon p = 0.84). Significant predictors of adverse long-term survival were COPD, postoperative TIA/stroke and length of stay. Survival is comparable but lower than age- and sex-matched first-time SAVR and England's general population.
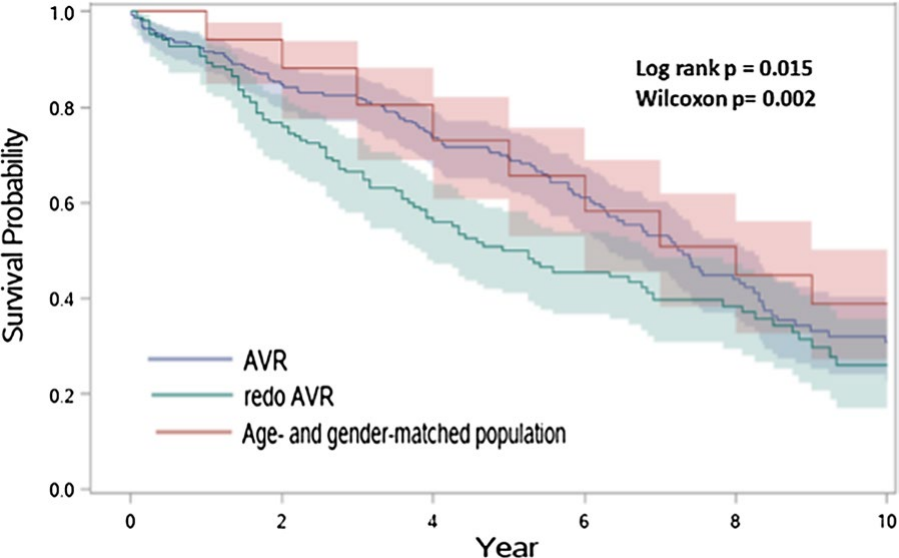


**Conclusions**

RedoSAVR in octogenarians is associated with significant morbidity and mortality although results are acceptable in carefully selected patients.

### A20 Aortic Valve Replacement and Coronary Artery Bypass Grafts. Do Numbers Matter?

#### Layton, Georgia R., Miss; Marsico, Roberto, Mr; Hadjinikolaou, Leon, Mr; Mariscalco, Giovanni, Mr; Murphy, Gavin, Prof; Zakkar, Mustafa, Mr

##### Department of Cardiac Surgery, Glenfield Hospital, University Hospitals of Leicester NHS Trust, Leicester, UK

*Journal of Cardiothoracic Surgery* 2023, **18(Supp 1)**:A20

**Objectives**

Concomitant aortic valve replacement (AVR) and coronary artery bypass grafting (CABG) is associated with a mean mortality of 5.4% in the UK. It has been debated whether number of grafts adversely impacts patient outcomes. We aim to investigate the influence of number of bypass grafts on in-hospital and long-term survival.

**Methods**

Retrospective analysis of prospectively collected data of consecutive patients undergoing AVR + CABG in a single unit between January 2016 and January 2021.

**Results**

406 patients (mean age 72.5 ± 7.5 years, mean EuroSCORE II 4.7% ± 6.4) were included: 65 had more than 2 grafts in addition to AVR.

There were no significant differences in mean age or surgical risk between groups. There was a preponderance of male patients with increasing graft number (p = 0.0027).

Overall, in-hospital mortality was 5.7% but this increased significantly with graft number (p = 0.01). Similarly, there was increased use of IABP by graft number (p = 0.002). Overall, 0.5% of patient had CVA and 2.2% had TIA which did not differ between groups (p = 0.7, 0.9 respectively). 22 patients (5.4%) required CVVHF with no difference between groups (p = 0.06). Logistic regression identified the performance of more than 2 grafts (OR 3.1, CI 1.2–7.5, P = 0.02), bypass time (OR 1.02, CI 1.02–1.04, p = 0.001) and BMI (OR 1.01, CI 0.38–8.8, p = 0.04) as independent predictors of in hospital mortality. Multivariate Cox model identified bypass time but not the number of grafts as independent predictor of long-term survival. Similarly, 5 years survival was not impacted by the number of grafts (log rank p = 0.099).

**Conclusions**

More than 2 bypass grafts in addition to AVR is associated with increased in-hospital mortality and use of IABP. This highlights the need for consideration of alternative intervention or hybrid approach for such high-risk patients.

### A21 Redo Intervention on the Aortic Valve: Indications, Outcomes and Factors Predicting Mortality

#### Oyebanji, Tunde, Dr; Aljanadi, Firas, Mr; Jones, Mark, Mr

##### Royal Victoria Hospital, Belfast, Northern Ireland

*Journal of Cardiothoracic Surgery* 2023, **18(Supp 1)**:A21

**Objectives**

To evaluate the indications and early and long-term outcomes of redo surgical aortic valve replacement (rSAVR).

**Methods**

The study was carried out retrospectively over ten years (2010–2020). We included patients requiring isolated redo-AVR, redo-AVR plus CABG, complex aortic surgery involving AVR, and concurrent mitral valve procedures. Primary outcome was mortality at 30-days, 1, 5 and 10 years. Secondary outcomes were 30-day incidences of stroke, AKI, myocardial infarction (MI), permanent pacemaker (PPM) requirement, and hospital stay (LOHS).

**Results**

83 patients had rSAVR during the period. 30 (36.6%) patients required rSAVR because of endocarditis (mechanical – 11 [13.2%], biological – 19 [23.4%]; p = 0.92) and 31 (37.8%) for bioprosthetic valve degeneration. 30 (36.6%) patients required concomitant procedures including CABG, aortic root enlargement, aortic root replacement and mitral valve surgeries. Mortality at 30-days, 1, 5 and 10 years was 14 (16.87%), 16 (19.28%), 19 (22.89%) and 22 (26.51%) respectively. Cox regression showed the following predictors of mortality: increasing age (HR 1.08, 95% CI 1.03 to 1.14, p = 0.001), long cardiopulmonary bypass time (HR 1.02, 95% CI 1.01 to 1.02, p < 0.001), endocarditis (HR 8.93, 95% CI 2.56 to 31.2, p < 0.001), peripheral vascular disease (HR 4.44, 95% CI 1.35 to 14.61, p = 0.01), valve and CABG (HR 21.34, 95% CI 1.55 to 294.62, p = 0.02), and moderate ejection fraction (HR 9.73, 95% CI 2.87 to 32.98, p < 0.001). Of patients that died within 30-days of surgery, 9 (64.2%) had endocarditis. The incidence of stroke, MI, AKI, and PPM were 2.44%, 3.87%, 15.86% and 15.86%, respectively. The mean LOHS was 26.9 ± 22.3 days. Mean survival time was 99.8 months. 1, 5, and 10-year survival were 80%, 74% and 64%, respectively.

**Conclusion**rSAVR achieves good mid and long-term outcomes, but there is significant postoperative morbidity and mortality. Endocarditis is a common indication and strongly affects outcomes.

**Table:** 

OUTCOME	TOTAL	MECHANICAL	BIOLOGICAL	P-VALUE
AGE (YEARS)	62.14 ± 15.82	56.5 ± 15.44	70 ± 13.11	< 0.05*
GENDER MALE FEMALE	83 50(60.24%) 33(39.76%)	48(57.83) 29(34.94%) 19(22.89%)	35 (42.17%) 21(25.3%) 14(16.87%)	0.97
LOGISTIC EUROSCORE	26.22 ± 21.9	21.77 ± 19.7	32.33 ± 23.55	0.03
MORTALITY 30-DAY 1-YEAR 5-YEARS	14(16.87%) 16(19.28%) 19(22.89%) 22(26.51%)	8(9.64%) 9(10.84%) 11(13.25%) 13(15.66%)	6(7.23%) 7(8.43%) 8(9.64%) 9(10.84%)	0.95
MEAN SURVIVAL (MONTHS)	99.8	98.6	93.4	0.79
SURGERY TYPE VALVE ONLY VALVE + CABG COMPLEX AORTIC	62(74.7%) 9(10.84%) 12(14.46%)	36(43.37%) 4(4.82%) 8(9.64%)	26(31.33%) 5(6.02%) 4(4.82%)	0.59
MYOCARDIAL INFARCTION	3 (3.85%)	0	3 (3.85%)	0.04
STROKE	2 (2.44%)	0	2 (2.44%)	0.097
ACUTE KIDNEY INJURY	13 (15.86%)	6 (7.32%)	7 (8.54%)	0.37

### A22 Analysis of Target INRs in Patients with On-X Mechanical Aortic Valve Replacement – The Gap Between Evidence and Real World Practice

#### Mangel, Tobin, Dr; Rai, Karan, Dr; Yates, Martin, Mr; Balmforth, Damian, Mr; Lopez-Marco, Ana, Ms; Shipolini, Alex, Mr; Uppal, Rakesh, Prof; Oo, Aung, Prof

##### St Bartholomew's Hospital, London, UK

*Journal of Cardiothoracic Surgery* 2023, **18(Supp 1)**:A22

**Objective**

The PROACT trial showed the safety of a lower target INR (1.5–2) + Asprin in patients with an On-X mechanical valve in the aortic position. This is thought to reduce the risk of anticoagulation-associated bleeding however some surgeons may be reluctant to reduce target INR. We aim to determine if clinical practice reflects the evidence for On-X valve INR target values.

**Methods**

All patients undergoing On-X mechanical aortic valve replacement from June 2017 to March 2021 were included. Those being anticoagulated for other reasons were excluded. Electronic patient records were reviewed. Primary outcome was anticoagulation regime three months following surgery. Secondary outcomes were presence or absence of discussion of valve choice and anticoagulation plans in the pre or post-operative period.

**Results**

On-X valves were implanted in 156 patients. Mean age 48 years, 119(76%) were male, 121(78%) elective and 35(22%) urgent. Seventy-five (48%) of patients had a documented discussion regarding valve choice preoperatively however only eleven (7%) mentioned specific type of mechanical valve. Seventy-nine (51%) discharge letters had a post-operative anticoagulation plan for low INR + Aspirin. Of these, 8(10%) were from doctors and 71(90%) were from pharmacy. Follow up clinic letters mentioned low INR + aspirin 28 (18%) patients. Only 34(22%) patients met primary outcome of low INR + Aspirin at three months.

**Conclusion**

Despite evidence to run a lower INR, the majority of patients are not advised of this. This may be due to lack of communication with patients or surgeons/anticoagulation clinic staffs reluctance to lower INR targets.

### A23 Trifecta Aortic Valve Bioprosthesis—Excellent Early and Long Term Outcomes

#### Karuppannan, Mukesh, Mr; Rose, David, Mr; Walker, Antony, Mr; Bose, Amal, Mr

##### Blackpool Victoria Hospital, Blackpool, UK

*Journal of Cardiothoracic Surgery* 2023, **18(Supp 1)**:A23

**Objectives**

The Trifecta bio-prosthesis is a bovine pericardial valve externally mounted on a titanium stent. Reports of early valve degeneration led to an evaluation of our experience with this valve and its long-term outcomes.

**Methods**

Patients undergoing aortic valve replacement (AVR) with the Trifecta valve between May 2011 and December 2019 at a single centre were included. The primary outcome was overall survival. Secondary outcomes included operative mortality and morbidity, aortic valve re-operations, and re-operation for structural valve deterioration. Echocardiographic outcomes were evaluated.

**Results**

The study included 419 Trifecta valve implants (2 03—first generation, 216—GT series). Operations included isolated AVR in 211 (50.35%), AVR plus coronary artery bypass grafting in 165 (39.37%), and AVR plus mitral valve operation in 43 (10.26%). AVR by minimal access technique was used in 53 patients (12.64%). Early mortality rate was 3.81% (n = 16). Overall survival at 1 year, 5 years and 10 years were 89.03%, 77.08% and 73.51% respectively (Fig. 1). Overall freedom from aortic valve re-operation was 98.33% and 98.1% at 5 years and 10 years respectively. There were a total of 8 re-operations (median 3.4 years, IQR 3.46) with 2 re-operations in < 1 year and 4 late re-operations, giving a total 10-year re-operation rate of 1.90%. Of these, 4 were for infective endocarditis, 1 was for paravavular leak and 3 were for structural valve degeneration (mean 4.52 years). Overall mean gradients were 6.9 ± 5.2 mm Hg postoperatively and remained low at 10.5 ± 6.4 mm Hg at 1 year.

**Conclusions**

Our results demonstrate that this valve can be safely implanted in the aortic position with excellent long-term durability and haemodynamics.
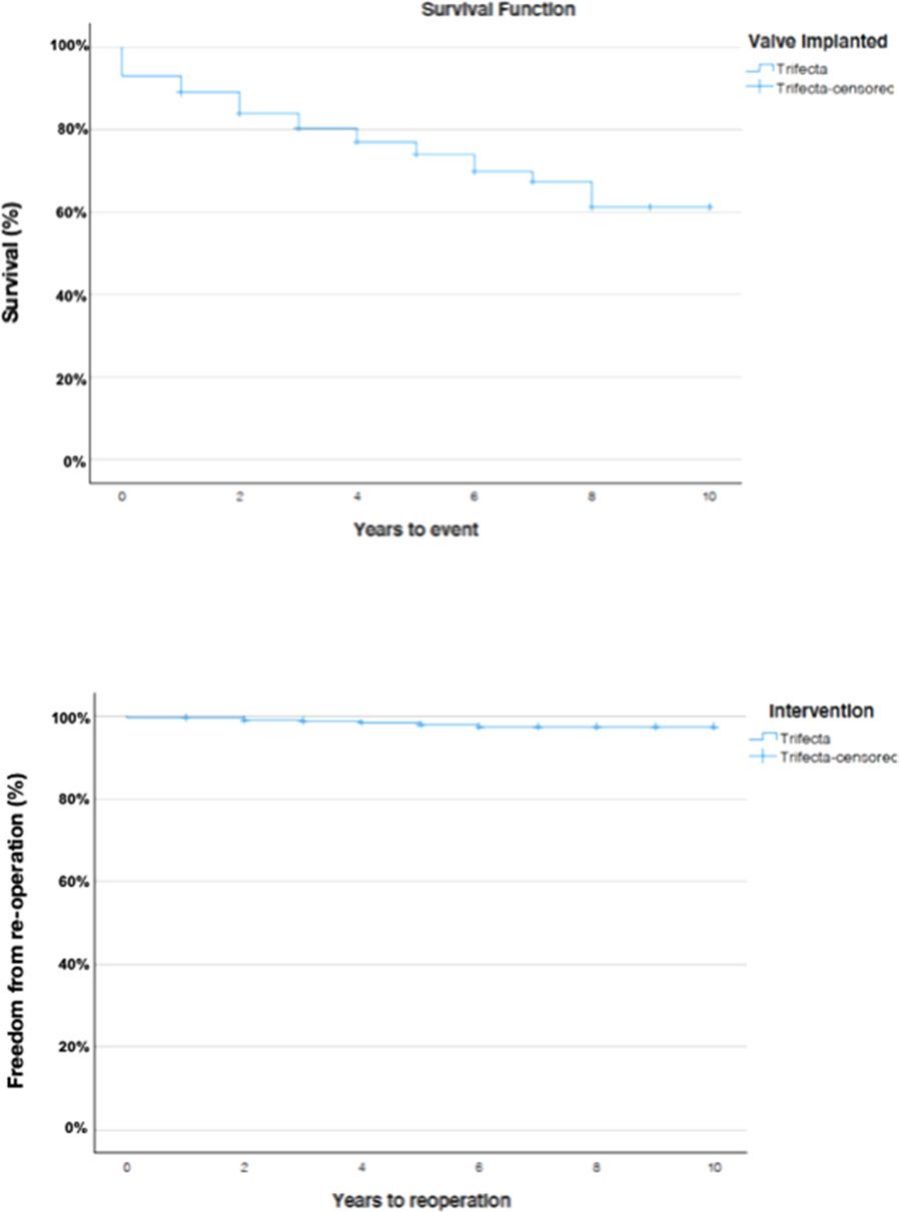


### A24 Surgical Aortic Valve Replacement Outcomes in Young Patients Under the Age of 60

#### Meuris, Bart^1^, Professor; Senage, Thomas^2^, Prof; Borger, Michael^3^, Prof; Siepe, Matthias^4^, Prof; Stefano, Pierluigi^5^, Prof; Laufer, Guenther^6^, Prof; Langanay, Thierry^7^, Prof; De Paulis, Ruggero^8^, Prof

##### ^1^University Hospitals Leuven, Leuven, Belgium; ^2^Centre Hospitalier Universitaire de Nantes, Nantes, France; ^3^Leipzig Heart Center, Leipzig, Germany; ^4^Freiburg Heart Center, Baden-Wurttemberg, Germany; ^5^Careggi University Hospital, Firenze, Italy; ^6^Heart Center Wien, Wien, Austria; ^7^CHU Rennes, Rennes, France; ^8^European Hospital Rome, Rome, Italy

*Journal of Cardiothoracic Surgery* 2023, **18(Supp 1)**:A24

**Background**

Prospective outcomes of bioprosthetic surgical aortic valve replacement (SAVR) in young patients are scarce, but critical for decision-making. We assessed VARC-2 time-related events and freedom from stage-3 SVD in patients below 60y.

**Methods**

INDURE is a prospective, multicenter registry with a 5-year core-lab adjudicated follow-up, to assess the clinical outcomes of sAVR in patients younger than 60 years who received a novel bioprosthetic valve *(NCT03666741)*.

**Results**

435 patients ≤ 60y were enrolled at 21 sites in Europe and Canada: mean age 53 years, female gender 22.5%, EuroSCORE II 1.6 ± 1.9% and 73.9% of bicuspid valve morphology (BAV). Comparison of 103 patients ≤ 50y versus 332 patients 51-60y showed significant differences: BAV 82.5% vs. 70.5% (p = 0.016), AR dominance 33.3% vs. 20.8% (p = 0.009), severe AR w/o significant valve stenosis 19.6% vs. 11.4% (p = 0.034), diabetes 6.8% vs. 15.4% (p = 0.025), hypertension 31.1 vs. 55.7% (p < 0.001). Isolated AVR was performed in 59% of cases. Valve size 23-25 mm were implanted in 59% of the patients. Median (IQR) hospital stay was 7d (6–10), ICU stay 29.5 h (22–56) with a 30-day mortality of 0.7% for the entire cohort. At 1 year follow-up (195/435), preliminary safety outcomes resulted in 3.6% (7/195) of all-cause mortality (42.9% not valve-related), 1.1% (2/189) endocarditis, and no stage-3 SVD; valve hemodynamics were stable with MPG 12.7 ± 5.6, EOA 1.8 ± 0.5 cm^2^.

**Conclusions**

Patients ≤ 50 years old undergoing SAVR were more likely to have a bicuspid aortic valve or aortic valve regurgitation at baseline, and less likely to have aortic stenosis, hypertension, or diabetes. INDURE registry data indicate excellent 30-day outcomes of SAVR with a new tissue valve in young patients, including valve performance being comparable across age subgroups. Preliminary 1 year follow-up outcomes confirm satisfactory safety and valve performance with no stage-3 SVD. Further follow-up is ongoing with echo corelab reviewed data.

### A25 Avoiding Prosthesis-Patient Mismatch: The Role of Valve MDT and Root Enlargement

#### Sherif, Mohamed^1^, Mr; Khan, Tanveer^1^, Mr; Capoccia, Massimo^2^, Mr; Elmahdy, Walid^1^, Mr

##### ^1^Leeds General Infirmary, Leeds, UK; ^2^Bristol Heart Institute, Bristol, UK

*Journal of Cardiothoracic Surgery* 2023, **18(Supp 1)**:A25

**Objective**

To report early results of a case series of patients with high preoperative risk of PPM based on aortic root assessment. These patients were discussed pre-operatively in valve MDT and had a planned aortic root enlargement (ARE).

**Patients and Methods**

We present the outcomes of 13 patients who had a preoperative predicated PPM and underwent planned root enlargement post Valve MDT discussion. PPM risk was evaluated and predicated by an effective orifice area index EOAI less than 0.85 cm2 m^−2^.

Pre and post-operative patients’ data collected from our local patients’ data software. There were 2 (15.4%) male patients and 11 (84.6%) female patients who underwent electively planned ARE and AVR ( ±) CABG. The mean age was 68.7 ± 5.3 years (61-77 years), body mass index (BMI) 30.2 ± 5.7 kg m^−2^ and body surface area (BSA) 1.77 ± 0.30 m2. Patients had mean Euroscore II of 11.2 ± 9.3.

Preoperative mean aortic valve area of 0.59 ± 0.20 cm2, mean gradient of 59 ± 21 mmHg and peak gradient of 97 ± 32 mmHg. The mean aortic annulus size was 17 ± 1.5 mm.

**Results**

The mean implanted valve size was 22.4 ± 1.2 (19–23). The mean increase in valve size post enlargement was 5.3 mm (SD 1.9 mm), and that was statically significant (p < 0.001). The mean bypass time and aortic clamping times were 144 ± 30.9 min and 99 ± 18 min, respectively. All patients had significant reduction in peak and mean pressure gradients, with improvement in LV function.

There was no in-hospital mortality and all patients still alive during follow-up. There is 0% paravalvular leak. Only one patient had permanent pacemaker for complete heart block and one patient required surgical drainage of pericardial effusion. None of the patients had TIA / stroke, renal or respiratory failure.

**Conclusion**

PPM can be predicated per-operatively and should be discussed in valve MDT for individualised planning. Elective root enlargement in trained hands is a safe solution for preven PPM.

### A26 New Innovations, New Operations & Difficult Decisions—Movie

#### Deglurkar, Indu, Miss; Syed Nong Chek, Syed Aidil Hizman, Mr; John, Anish, Mr; Karthikeyan, Sivagnanam, Dr

##### University Hospital of Wales, Cardiff, UK

*Journal of Cardiothoracic Surgery* 2023, **18(Supp 1)**:A26

https://www.youtube.com/embed/tFF8xQRSZe8

### A27 Patient Prosthesis Mismatch in Aortic Valve Replacement: Are all Bioprosthetic Valves the Same?

#### Haqzad, Yama, Mr; Chrysikopoulou, Megan, Dr; Ripoll, Brenda, Ms; Jarvis, Martin, Mr; Chaudhry, Mubarak, Mr; Loubani, Mahmoud, Prof

##### Castle Hill Hospital, Hull, UK

*Journal of Cardiothoracic Surgery* 2023, **18(Supp 1)**:A27

**Introduction**

Patient Prosthesis Mismatch (PPM) is associated with increased morbidity and mortality. Standard cut-offs for adjudicating PPM in a patient are effective orifice area index (EOAI) 0.85 to 0.65 cm2/m2 for moderate PPM and < 0.65 cm2/m2 for severe PPM.

**Objectives**

Retrospective analysis of data from cardiothoracic database for patients undergoing isolated aortic valve replacement (AVR) between 2010–2014. EOA of different valve types were obtained from the respective manufacturers. EOAI was determined using EOA divided by body surface area of the patient. Data was analysed using SPSS 24.

**Results**

240 patients were identified (149 (62%) males and 91 (38%) females). Mean age 74 ± 8.7.

Over 11% (27/240) of patients have severe PPM. Moderate to severe PPM was significantly higher in patients with BMI > 25 (64.6%) compared to BMI < 25 (37.1%) p < 0.001. Severe PPM was present in over 28% of Hancock II valves. Moderate PPM was present in 88% of Mitroflow, 68% of Hancock II, 51% of St Jude Epic, 27% of St Jude Trifecta, 7% of Sorin Soprano, 3% of Perimount Magna Ease with p < 0.001. Mortality at 7 years was 56% in moderate to severe PPM versus 54% in mild/non-significant PPM. Average valve sizes used were Hancock II 22.7 ± 2, Mitroflow 22.7 ± 2.1, St Jude Epic 22.8 ± 2, St Jude Trifecta 22.9 ± 2.1, Sorin Soprano 22.8 ± 2.1 and Perimount Magna Ease 22.8 ± 2.1.

**Conclusion**

In our study, the incidence of moderate to severe PPM varied significantly depending on the type of bioprosthesis. Additionally, overweight and obese patients had significantly higher risk of moderate to severe PPM. We suggest that in this group of patients, the EOA of valve type should be carefully considered. This is particularly important as the Valve Academic Research Consortium recommends lower cut-offs for moderate/severe PPM in patients with BMI > 30.

### A28 Are There any Predominant Clusters of Pathology in Patients with Aortic Aneurysms Presenting for Aortic valve and Root Repair?

#### Sriskandarajah, Sanjeevan, Mr; Abdul Hakeem, Muhammad, Mr; Popescu, Florentina, Miss; Kenawy, Ayman, Mr; Harrington, Deborah, Miss; Othman, Ahmed, Mr; Kuduvalli, Manoj, Mr; Field, Mark, Mr; Nawaytou, Omar, Mr

##### Liverpool Heart and Chest Hospital, Liverpool, UK

*Journal of Cardiothoracic Surgery* 2023, **18(Supp 1)**:A28

**Objective**

Patients with underlying aortic aneurysms rarely present with singular pathology leading to their aortic regurgitation. The aim of our study was to identify any common clusters of pathology in a modern sample of patients presenting for aortic valve and root reconstruction and to compare the techniques currently used against the proposed El Khoury classification.

**Methods**

Patients from a single centre who were listed for aortic valve sparing root and valve repair from August 2017 to September 2021 were included in the study. Patients with sole leaflet pathology, annular dilatation or dissection without an aortic aneurysm were excluded from the study.

**Results**

A total of 178 patients were identified during this period and 159 of those patients underwent a root and valve repair. Following application of the exclusion criteria a total of 144 patients were identified. Five main clusters were noted in these patients. Cluster 1 (El Khoury Ia) 9.7% of them 57% needed a David procedure and 43% had an ascending aortic replacement and annuloplasty. Cluster 2 (El Khoury Ib) 30.5%, 93% of them were treated with a David procedure. Cluster 3—(El Khoury Ib/Ic) 25%, 97% of them had a David procedure and 17% of them needed concomitant leaflet repair. Cluster 4 (El Khoury Ib/IC/II & Ib /II) – 18.8%, 96% of these patients had a David procedure and 89% of them also needed concomitant leaflet repair. Cluster 5 (El Khoury Ib/Ic/ III & Ib /III)—9%, 77% underwent a David Procedure with leaflet repair, with 8% undergoing a David procedure alone and the remainder 15% had an ascending aorta replacement with an annuloplasty.

**Conclusion**

The described five clusters accounted for 89.5% of patients undergoing root and valve repair with underlying aneurysms. Isolated aneurysms commonly need an annuloplasty to augment repair and isolated SOV aneurysm may require concomitant leaflet repair. David procedure alone can sometimes be sufficient in some cases of valve prolapse or restriction.

### A29 Short and Medium-term Outcomes of Different Surgical Approaches for Transcatheter Aortic Valve Implantation

#### Metwalli, AMr, Mr; Salmasi, M Yousuf, Mr; Zientara, Alicija, Ms; Duncan, Alison, Ms; Shannon, Joanne, Ms; Quarto, Cesare, Mr

##### Royal Brompton and Harefield NHS Foundation Trust, London, UK

*Journal of Cardiothoracic Surgery* 2023, **18(Supp 1)**:A29

**Objectives**

To review the early to medium-term outcomes after different surgical approaches for transcatheter aortic valve implantation (TAVI).

**Methods**

We retrospectively reviewed the survival data and patient demographics of consecutive cases who underwent TAVI using different surgical approaches; transapical, subclavian, axillary and carotid, over an 8-year period (March 2013 to September 2021).

**Results**

We performed a total of 82 TAVI procedures with different surgical approaches. The mean age was 80 ± 7 years and the age-range was 57–94 years. 80% of cases were aged 75 or older and 52.4% were 80 or older at the time of intervention. 46/82 (56%) had a Katz Index of Independence score of 5 or above, while 34/82 (41.4%) had a score of 3 or 4. The surgical approaches were either trans-subclavian/axillary 48/82 (58.5%), trans-apical 22/82 (26.8%) and trans-carotid 11/82 (13%), with 7 cases (8.5%) performed under elective cardiopulmonary bypass support due to poor ventricular function. The observed survival rate at 30-days, 1 year, 3 years and 5 years was 97.5%, 87%, 71% and 54%, respectively. Survival analysis found no difference between trans-apical and trans-subclavian approaches (logrank p = 0.981). Cox regression analysis found no influence of key co-variates on survival, including age, renal function, peripheral vascular disease and LV function (p > 0.05).

**Conclusion**

Different surgical approaches for TAVI including subclavian, transapical and carotid approaches, are viable alternative approaches for patients who are not suitable for the trans-femoral approach.

### A30 Minimal Access Aortic Valve Replacement: Impact on Outcome in Elderly Patients

#### Sharma, Sobaran, Mr

##### Morriston Hospital, Swansea, Swansea, UK

*Journal of Cardiothoracic Surgery* 2023, **18(Supp 1)**:A30

**Objective**

We assessed the Impact of mini-AVR (j sternotomy) aortic valve replacement for isolated Aortic valve replacement against Full sternotomy in Elderly patients and compared the outcomes.

**Methods**

We retrieved the operative records of elderly patients aged 70 years and over undergoing isolated aortic valve replacement between 2006 to March 2020.demographic and peri-operative data between two group undergoing Full sternotomy (Group A) and mini-AVR (Group B) were compared.

**Results**

658 patients (Group A) and 182 patients (Group B) underwent isolated aortic valve replacement with Full sternotomy and J partial sternotomy incisions respectively. There was an increased proportion of comorbidities in Group B as reflected in the significantly greater Logistic EuroSCORE (8.9% vs 10.4%,p < 0.05).Cardiopulmonary bypass (101.8 vs 77 min, p < 0.001) and Cross-clamp times (82.7 vs 64.3 min,p < 0.01) were shorter in Group B. The cardiac intensive care utilisation more than 24 h (49.7% vs 38.3%was significantly lower in Group B, p = 0.050), who interestingly also had a significantly shorter post-operative hospital stay(11 vs 7.6 days,p < 0.05). There was a significantly lower re-operation for bleeding (5.5% vs 1.7%, p < 0.05). There was a significantly lower usage of packed cell units in Group B (2.3 vs 1.5 units, p = 0.009).

**Conclusions**

We have demonstrated that minimal access (mini-AVR) approach for aortic valve replacement can provide substantial clinical benefits in the elderly and comorbid patients, in addition to utilising fewer hospital resources such as postoperative care facilities, length of stay and blood transfusion.

### A31 Blood Transfusion and 10-year Survival After Minimally Invasive AVR Versus Conventional AVR: A Propensity-matched Analysis

#### Poon, Sam, Mr; George, Joseph, Mr; Sharma, Sobaran, Mr; Kumar, Pankaj, Mr

##### Morriston Hospital, Swansea, UK

*Journal of Cardiothoracic Surgery* 2023, **18(Supp 1)**:A31

**Objective**

This study sought to investigate whether minimally invasive aortic valve replacement (Mini-AVR) required fewer blood transfusions and its impact on long term survival compared to conventional aortic valve replacement (AVR).

**Method**

A retrospective cohort study was carried out in a single centre. 274 patients received Mini-AVR and 1036 had AVR. A matched logistic regression study was carried out for these patients.

**Results**

The mean age of patients was 70 years old. 53% of patients in the Mini-AVR group received blood transfusion in comparison to 63.1% in the AVR group (p < 0.05). The mean unit of blood transfused was 1.8 and 2.5 units ( p = 0.045) in the mini-AVR and AVR group respectively. Both groups have comparable in-hospital mortality (Mini AVR 1.38% vs. AVR 1.06%, p > 0.05). After adjusting differences in peri-operative risk factors, there was no significant difference in 10 years survival (66% vs 60%, p > 0.05).

**Conclusion**

Mini-AVR is associated with fewer blood transfusions compared to AVR. There is a trend toward lower blood transfusion and better 10-year survival but these differences were not statistically significant.

### A32 The Impact of Blood Transfusion on Survival Following Isolated Aortic Valve Replacement: A ten-year Follow-up Result

#### Poon, Sam, Mr; Suhail, Sadiq, Dr; Chan, Jeremy, Mr; George, Joseph, Mr; Sharma, Sobaran, Mr; Kumar, Pankaj, Mr

##### Morriston Hospital, Swansea, UK

*Journal of Cardiothoracic Surgery* 2023, **18(Supp 1)**:A32

**Objective**

Blood transfusion is common in cardiac surgery and the long-term impact remains unclear. We aim to investigate the impact of red blood cells transfusion on survival following aortic valve replacement.

**Method**

A retrospective cohort study on 490 consecutive patients who underwent isolated aortic valve replacement (AVR) from January 2007 to September 2011 was undertaken. The mean duration of follow-up was 10 years and the mean age of patients was 69. The overall 10-year survival was analyzed in relation of red blood cells transfusion. A matched propensity score based on logistic regression analysis was performed.

**Results**

Overall 39% of patients received blood transfusion. The mean pre-operative haemoglobin was 13.2 gm/dL and mean unit of blood transfused was 2 units. Following propensity matching, perioperative blood transfusion was associated with poorer survival in 10 years compared to patients who had no blood transfusion (71.4% vs. 61%, p < 0.001, HR 1.24 95% CI 1.13–1.37, p < 0.001). A subgroup analysis on the number of blood units transfusion showed that 1 and 2 units of blood transfusion did not adversely impact on survival but for patients receiving 3–4, and more than 5 units of blood products, there is a positive correlation for significant reduction in 10 years survival. (Hazard ratio (HR) for 3–4 units was 2.0 (95% CI 1.2–3.3, p = 0.005) and more than 5 units HR 2.9 (95% CI 1.8–4.7, p < 0.001).

**Conclusion**

Blood transfusion is associated with reduced long-term survival following aortic valve replacement. Patients receiving more than 3 units of blood had a significant decrease in survival compared to expected survival. Pre-operative patient optimization may improve long-term outcomes by reducing the likelihood of blood transfusion.
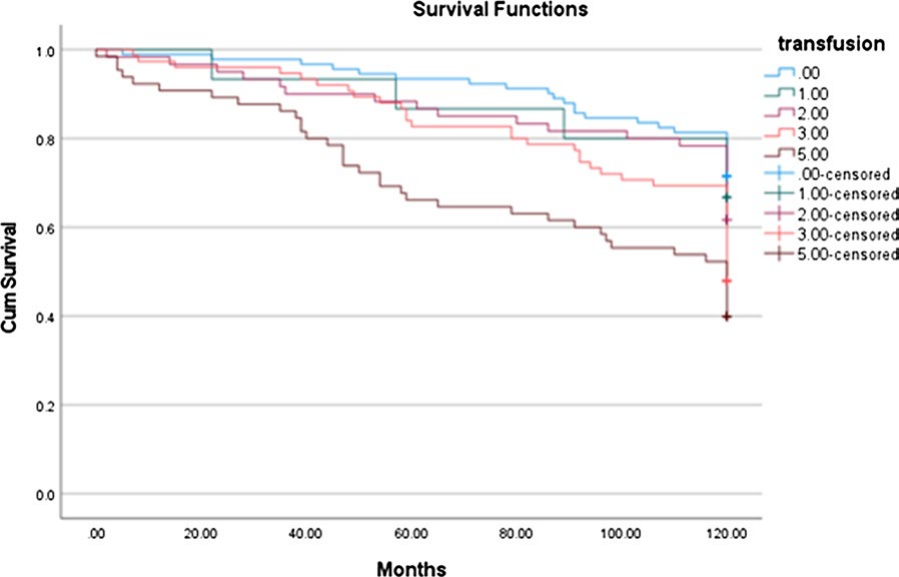


Figure 1. A 10-year Kaplan–Meier (KM) survival curve following blood transfusion in AVR was plotted. A subgroup analysis on the impact of the number of blood products.

## Adult Cardiac Coronary

### A33 Modelling Long Term Outcomes in Patients with Heart Failure Revascularized with CABG or PCI using Hospital Episode Statistics

#### Pathak, Suraj^1^, Dr; Lai, Florence^1^, Mrs; Miksza, Joanne^1^, Ms; Petrie, Mark^2^, Prof; Murphy, Gavin^1^, Prof

##### ^1^Glenfield Hospital, Leicester, UK; ^2^University of Glasgow, Glasgow, UK

*Journal of Cardiothoracic Surgery* 2023, **18(Supp 1)**:A33

**Objectives**

The REVASC-HF-UK trial, a trial of revascularisation strategies (PTCA/CABG) for ischemic heart failure (IHF) patients, was modelled using the Hospital Episode Statistics (HES) Admitted Patient Care (APC) data set and the national death registry.

**Methods**

Patients undergoing isolated CABG and high-risk stenting (HR-PTCA) in England between April 2012 and March 2015, with a preceding diagnosis of IHF within 2 years of the index procedure, were identified. Outcomes of interests included all-cause mortality and MACE. Treatment effects were estimated using regression adjustment (RA), propensity score matching (PSM) and instrumental variable analysis (IVA).

**Results**

2462 patients were identified in the CABG arm and 1033 patients were identified in the HR-PTCA arm. Risk of death was lower for CABG than HR-PTCA (unadjusted OR for 1, 3, 5-year mortality = 0.54, 0.38, 0.39 respectively). Risk of MACE outcomes was lower for CABG (unadjusted OR for 1, 3, 5-year MACE = 0.52, 0.40, 0.38 respectively). This treatment effect favoring CABG persisted after RA, PSM and IVA methods were applied.

**Conclusion**

In patients with IHF, risks of death and MACE events at 1, 3 and 5 years were lower after CABG than HR-PTCA.

### A34 Impact of Preoperative Atrial Fibrillation on Outcomes After Elective Coronary Artery By-pass Grafting: A Large Database Analysis

#### Fudulu, Daniel^1^, Mr; Dimagli, Arnaldo^1^, Mr; Dong, Tim^1^, Mr; Gemelli, Marco^1^, Mr; Chan, Jeremy^2^, Dr; Sinha, Shubhra^1^, Miss; Benedetto, Umberto^1^, Prof; Angelini, Gianni^1^, Prof

##### ^1^Bristol Heart Institute, Bristol, UK; ^2^Bristol Royal Infirmary, Bristol, UK

*Journal of Cardiothoracic Surgery* 2023, **18(Supp 1)**:A34

**Objectives**

Atrial fibrillation (AF) is the most common sustained arrythmia in adults with a prevalence of 2–4% in adults with significant impact on mortality and mortality. In the cardiac surgery population, perioperative AF is associated with increased mortality, morbidity and excess of healthcare costs. Currently, pre-operative AF is not included as a risk factor in the most commonly used risk prediction tools (EuroSCORE II or STS). The objective of our study was to assess if preoperative, non-valvular AF is predictor for mortality and post-operative stroke by interrogating a very large cardiac audit dataset from Europe (UK National Cardiac Audit Dataset).

**Methods**

We included all isolated, elective coronary artery by-pass grafting procedures performed between February 1996 and March 2019. Incidence of AF was 3% on a total sample size of 244,801 patients. We have used a generalised linear mixed model to assess the effect of preoperative AF on mortality and stroke after adjusting for the relevant confounders derived from EuroSCORE 2. Confounders considered included: age, gender, neurological dysfunction, renal dysfunction, recent myocardial infarction, pulmonary disease, unstable angina (CCS4), NYHA class, pulmonary hypertension, diabetes on insulin and peripheral vascular disease. We have treated the hospital and operating consultant as random effect variables. We have also tested interactions between the pre-operative AF and pre-operative LV function our model.

**Results**

Preoperative AF was significant predictor for increased mortality (OR: 1.65, CI 1.42–1.91, P < 0.001) and postoperative CVA (OR:1.31, CI 1.07–1.59, P = 0.008) after CABG (attached figure). We found no significant interaction between pre-op AF and LV function for mortality or stroke outcomes.

**Conclusion**

Our study suggests that preoperative atrial fibrillation is associated with an increased risk for perioperative mortality and stroke in patients undergoing coronary artery bypass grafting.

### A35 Transit Time Flowmetry During off-pump Coronary Artery Bypass Surgery: How to Make Clinical Data Findable, Accessible, Interoperable and Reusable

#### Halfwerk, Frank^1^, Dr; Mariani, Silvia^2^, Ms; Hagmeijer, Rob^3^, Dr; Clare, Connie^4^, Dr; Grandjean, Jan^1^, Prof

##### ^1^Thoraxcentrum Twente, Medisch Spectrum Twente, Enschede, The Netherlands; ^2^Department of Cardio‐Thoracic Surgery, Maastricht University Medical Centre (MUMC), Maastricht, The Netherlands; ^3^Dept. of Engineering Fluid Dynamics, University of Twente, Enschede, The Netherlands; ^4^4TU.ResearchData, Delft University of Technology, Delft, The Netherlands

*Journal of Cardiothoracic Surgery* 2023, **18(Supp 1)**:A35

**Objectives**

Increasingly more clinical studies are published under the Open Access publishing model making them accessible online to everyone for free. Often, the only aspect that is not yet open is the data underlying these publications. Publishing data improves reproducibility and reliability of research, it increases visibility of research, and accelerates innovation. Furthermore, unique and highly valuable data from i.e. rare cardiac diseases or surgical techniques is not available to everyone.

The aim of this study is to present a best practice for publishing clinical data. A clinical study on intraoperative transit-time flowmetry during off‐pump coronary artery bypass surgery and the impact of coronary stenosis on competitive flow is used as an example.

**Methods**

Data is published according to the FAIR principles: Findable, Accessible, Interoperable and Reusable. To be ‘Findable’, a unique digital object identifier (DOI) was assigned to the dataset, and metadata described the content, contact information, location, items and definitions. The data is ‘Accessible’ for everyone under Open Access. To be ‘Interoperable’, MeSH and STROBE standards were used. Finally, to be’Reusable’, the data were made readable by others, and a license permitting data reuse was assigned.

**Results**

Data for 50 study patients were refined, and patient data anonymised. Date of birth information was grouped by age intervals, so it can be openly published in an external repository. Transit time flow measurements, definitions, study protocol, and the variable list were described. The dataset was made publicly available in the 4TU.ResearchData repository. Researchers should be attributed when data is reused under a CC-BY licence.

**Conclusion**

For cardiac surgery studies, it is feasible to publish data alongside Open Access peer-reviewed journal articles. The FAIR principles for clinical data management should be incorporated in the design and implementation of future clinical studies.

### A36 0ff-pump Coronary Artery Bypass Grafting Reduces Inhospital Mortality & Need for Renal Replacement Therapy in Patients with Moderate Renal Dysfunction

#### Garg, Sheena^1^, Ms; Raja, Shahzad^1^, Mr; Bhudia, Sunil^1^, Mr; De Robertis, Fabio^1^, Mr; Marczin, Nandor^1^, Dr; Layson, Rhae^2^, Dr; Lim, Ru jin^2^, Dr; Adikoesoema, Mohamad Shafiq^2^, Dr

##### ^1^Royal Brompton & Harefield NHS Trust, London, UK; ^2^University College London, London, UK

*Journal of Cardiothoracic Surgery* 2023, **18(Supp 1)**:A36

**Objective**

Moderate renal dysfunction (eGFR 30–59 mL/min/ 1.73 m2) has been consistently identified as a major predictor for postoperative renal failure and increased mortality after on-pump coronary artery bypass grafting (CABG). We analysed our institutional database to determine the impact of offering off-pump CABG on in-hospital mortality and need for renal replacement therapy (RRT) for this high-risk cohort of patients.

**Methods**

From January 2007 to December 2019, 2850 patients with moderate renal dysfunction underwent isolated first-time CABG at our institution. Multivariable logistic regression was used to investigate the effect of off-pump CABG on in-hospital mortality and need for RRT. Propensity score matching was used to compare the 2 matched groups.

**Results**

Over the study period, 1383 off-pump CABG and 1467 on-pump CABG were performed for this cohort. Fewer in-hospital deaths (11 [0.80%] vs 25 [1.81%]; p = 0.029) and reduced need for RRT (3 [0.22%] vs 9 [0.65%]; p = 0.048) was observed for the matched off-pump group compared to on-pump group. Off-pump CABG was associated with a significantly lower incidence of in-hospital death (odds ratio: 0.44; 95% confidence interval [0.21–0.89]) and need for RRT (OR: 0.33; 95% CI [0.09–1.23]).

**Conclusion**

Off-pump CABG should be preferentially offered to patients with moderate renal dysfunction.

### A37 Making CABG Less Invasive: Lessons Learned from Setting up an Endoscopic Vessel Harvesting Programme

#### Gradinariu, George^1^, Mr; Sobhun, Ganesh^2^, Mr; Olivar, Marimel^2^, Miss; Sereda, Victor^3^, Dr; Mahmood, Zahid^1^, Mr; El-Shafei, Hussain^4^, Mr; Sutherland, Fraser^1^, Mr

##### ^1^Golden Jubilee National Hospital, Glasgow, UK; ^2^Getinge UK; ^3^BMI Ross Hall Hospital, Glasgow, UK; ^4^Aberdeen Royal Infirmary, Aberdeen, UK

*Journal of Cardiothoracic Surgery* 2023, **18(Supp 1)**:A37

**Objectives**

Endoscopic vessel harvesting (EVH) for coronary artery bypass grafting (CABG) is known to offer patients faster recovery, fewer wound complications and shorter hospital stay. Our aim was to make CABG less invasive and we set out to establish an EVH programme in our unit. We describe the obstacles and challenges encountered as well as results from our first series of cases.

**Methods**

We first performed a comprehensive analysis of the expectations, needs and views shared across all involved players in the patient’s journey. Areas of concern were highlighted and addressed in a systematic fashion. We used a novel on-table high-definition surgical monitor, along with latest generation EVH harvesting tools and standard image capture/CO2 insufflation. Consecutive patients undergoing isolated CABG with EVH were recruited. Data on risk factors, wound complications, patient satisfaction and length of stay were collected.

**Results**

Three main areas of concerns were identified and addressed before the programme started: Resistance to change, Fear of the learning curve and Perception of added cost. Eleven patients were recruited. Mean age was 61 years [95% CI 55–67 years]. 7 out of 11 (64%) patients had one or more risk factors for post-operative wound complications. The long saphenous vein was harvested endoscopically in 8 patients (73%) and the radial artery in 3 (27%) patients. The median number of grafts was 3 [range 2–5]. There were no wound complications. All patients expressed a high level of satisfaction. Median post-operative length of stay was 5 days [range 4–6 days]. At a median follow-up of 3 months there were no late wound complications, no admissions to hospital or adverse events reported.

**Conclusion**

In our quest to make CABG less invasive, we successfully established an EVH programme and performed our initial series of cases with excellent outcomes. The combination of devices was easy to use and integrate into the standard CABG theatre footprint and procedure.
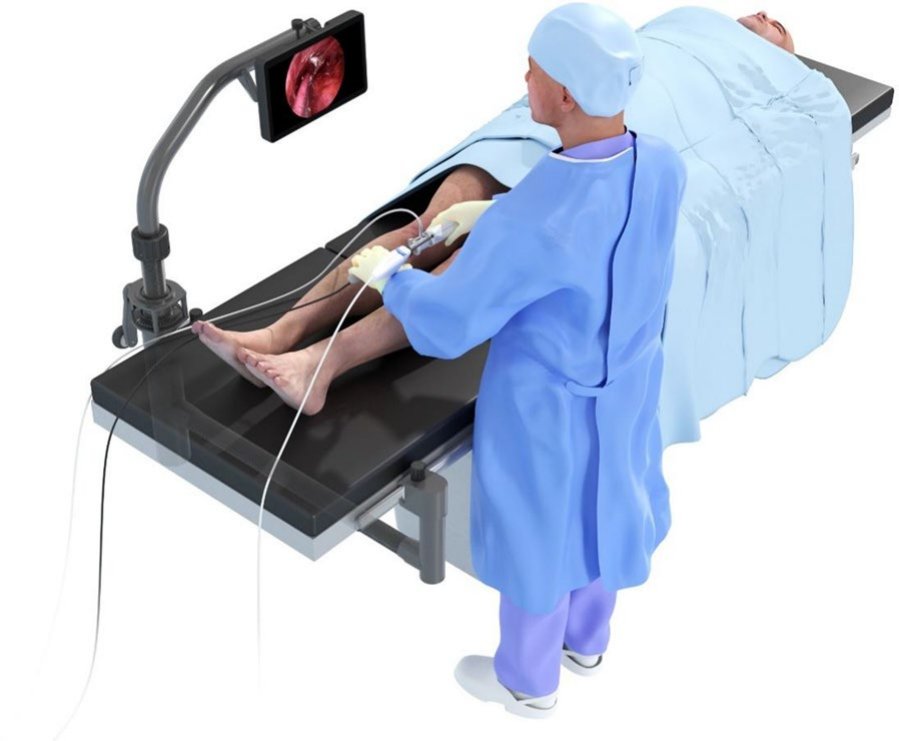


### A38 Emergency Off-pump Coronary Artery Bypass Grafting: A Myth or Reality?

#### Coppola, Giuditta, Miss; Farmidi, Abu Ali, Mr; Garg, Sheena, Miss; De Robertis, Fabio, Mr; Bahrami, Toufan, Mr; Bhudia, Sunil, Mr; Raja, Shahzad, Mr

##### Harefield Hospital, London, UK

*Journal of Cardiothoracic Surgery* 2023, **18(Supp 1)**:A38

**Objectives**

Emergency coronary artery bypass (CABG) is still considered a high-risk procedure due to the mortality and the post-operative morbidity compared to elective CABG. Although off-pump CABG(OPCABG) is a relative contraindication in emergency, we analyzed outcomes of 12-year-single center emergency OPCABG experience and compared it with om-pump CABG.

**Methods**

We retrospectively analysed prospectively collected data from institutional database from January 2007 to December 2019. During the study period 249 patients underwent an isolated emergency CABG.

**Results**

Mean age of study population was 66.14 years (± 11.86) and 193 patients (77.5%) were male. OPCABG was performed in 107(43%) patients. More distal anastomoses were performed in on-pump cohort (47.7% vs 69%; p = 0.001). Fewer patients required postoperative IABP in off-pump cohort (2.8% vs 9.9%; p = 0.053). All other outcomes including in-hospital mortality and mean length of hospital stay were similar for the two cohorts.

**Conclusions**

Emergency CABG still remains a challenge for the cardiac surgeon even in expert hands. However, OPCABG can be offered with comparable outcomes to patients needing emergency surgical revascularization in a high volume OPCABG centre.
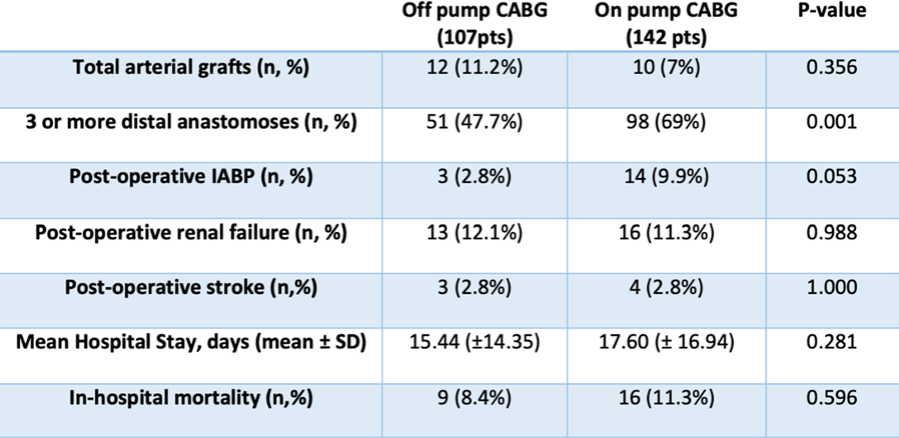


### A39 Role of CX3CR1 in Patients with Myocardial Ischaemia and Reperfusion Injury

#### Panahi, Pedram, Mr; Cormack, Suzanne, Dr; Mohammed, Ashfaq, Dr; Spyridopoulos, Ioakim, Prof

##### Newcastle University, Newcastle, UK

*Journal of Cardiothoracic Surgery* 2023, **18(Supp 1)**:A39

**Objectives**

Revascularisation of the blocked coronary artery in new onset ST segment elevation myocardial infarction (STEMI) can be achieved by primary percutaneous coronary intervention (PPCI). A complication of this is ischaemia–reperfusion injury (I/R-injury) which results in further damage to the ischaemic myocardium, accounting for up to 50% of the final infarct size in animal experiments. Interferon-γ secreting CD4 + T-cells, expressing the chemokine receptor CX3CR1, have been shown to mediate I/R-injury in animal models. Accordingly, CX3CR1 involvement in I/R-injury has been implicated in animal studies. The aim of this study was to determine the role of CX3CR1 in STEMI patients with I/R-injury.

**Methods**

42 acute STEMI patients undergoing PPCI were prospectively recruited. Blood samples, collected at various time points relative to reperfusion, were analysed using flow cytometry.

**Results**

(i) Effector T-cells had the highest CX3CR1 expression and a cytotoxic phenotype. (ii) Effector T-cell absolute count dropped after reperfusion. Effector T-cell CX3CR1 expression also dropped immediately after reperfusion, but then increased up to 24 h after reperfusion. Effector T-cells in HCMV seropositive individuals expressed higher levels of CX3CR1. (iii) Presence of microvascular obstruction was associated with lower CX3CR1 expression in the effector compartment.

**Conclusion**

CX3CR1, expressed at higher levels in HCMV seropositive individuals, is activated after reperfusion and coincides with an acute reduction in the effector T-cell population size. It is conceivable that these cytotoxic effector T-cells marginalise within the coronary microvasculature after reperfusion and contribute towards development of microvascular obstruction, a component of I/R-injury.

### A40 A Single-Centre Analysis of the use of Bilateral Internal Mammary Artery Graft in a Y-configuration in Coronary Artery Bypass Graft Surgery in Ireland

#### Whooley, Jack, Dr; Weedle, Rebecca, Dr; White, Alexandra, Dr; Soo, Alan, Mr

##### University Hospital Galway, Galway, Ireland

*Journal of Cardiothoracic Surgery* 2023, **18(Supp 1)**:A40

**Objectives**

Internal mammary artery grafts are the most durable conduits in coronary artery bypass graft (CABG) surgery resulting in improved long-term survival. The use of bilateral internal mammary artery (BIMA) grafts is especially advocated in younger patients. Concerns surrounding sternal wound dehiscence and technical difficulty have slowed the adoption of this technique. We aimed to assess our 5-year experience in performing BIMA grafting in a Y-configuration for CABG.

**Methods**

A retrospective review of patients undergoing CABG using BIMA in a Y-configuration between May 2016 and May 2021 was performed. All patients under 60 who underwent non-emergency CABG with at least two grafts that did not involve BIMA in the study period were also reviewed. Patient demographics, EuroSCORE II, operative details, post-operative length of stay (LOS), deep sternal wound infection (DSWI) and other complications were collected. Unpaired student t-test was used to compare the groups.

**Results**

Twenty-eight patient underwent CABG using BIMA in the 5-year study period. Eighty patients younger than 60 years of age underwent non-emergency CABG with other conduits in that time period. Patient demographics were similar between the groups. There was no difference in bypass time, but there was a significantly shorter cross-clamp time in patients. There was no significant difference in transfusion requirements or length of stay post-operatively between the groups. DSWI occurred in three patients in the BIMA cohort.

**Conclusion**

Using BIMA grafting in a Y-configuration is feasible, with shorter cross-clamp time and comparable length of stay. There is however an increased risk of deep sternal wound infection in patients with BIMA grafts. Patient selection remains an important consideration.

### A41 Enhanced Recovery After Surgery Protocols in Patients Undergoing Coronary Artery Bypass Graft Surgery: A Systematic Review and Meta-analysis

#### Elango, Madhivanan^1^, Dr; Kotta, Prasanti Alekhya^2^, Dr; Papalois, Vassilios^1^, Prof

##### ^1^Imperial College London, London, UK; ^2^King's College London, London, UK

*Journal of Cardiothoracic Surgery* 2023, **18(Supp 1)**:A41

**Objectives**

Enhanced recovery after surgery (ERAS) protocols are a multi-modal, multi-disciplinary approach to the management of the surgical patient in the pre-operative, intra-operative and post-operative phases. These protocols aim to reduce post-operative complications while decreasing the length of hospital stay. This study aims to systematically review the evidence for ERAS implementation in coronary artery bypass graft (CABG) surgery.

**Methods**

A database search was performed on MEDLINE and Embase to look for all studies comparing ERAS/fast-track protocols with standard protocols in patients undergoing CABG (both off-pump/on-pump). Observational and randomised controlled trials were included; studies including other cardiac operations and incomplete ERAS protocols were excluded. The primary outcome was hospital length of stay (LOS). Secondary outcomes included ICU LOS and time to extubation. Data were analysed on Review Manager 5.4.1 with a random-effects model.

**Results**

Seven studies were identified with a total of 2683 patients (1242 in ERAS group, 1441 in non-ERAS group). ERAS protocols were associated with a significant decrease in LOS (mean decrease 2.21 days, 95% CI 1.59–2.84 days). ERAS protocols were also associated with a significant decrease in ICU LOS (mean decrease 16.0 h, 95% CI 8.93–23.1 h) and time to extubation (mean decrease 11.2 h, 95% CI 4.01–18.3 h).

**Conclusions**

ERAS protocols were associated with a decreased time to extubation, ICU LOS and hospital LOS, all of which are known to be beneficial to patient satisfaction and reduced cost operation. ERAS protocols have been implemented in other fields of surgery and this analysis suggests the ERAS prinicples should be incorporated in coronary artery bypass grafting. More prospective work is needed to ascertain which elements should be included in a potential ERAS protocol given the heterogeneity of protocols in these studies.

### A42 Using Hospital Episode Statistics Data to Investigate the Effect of Frailty on Revascularisation Rates of Patients with Acute Coronary Syndrome

#### Miksza, Joanne, Ms; Lai, Florence, Ms; Roman, Marius, Dr; Murphy, Gavin, Prof

##### University of Leicester, Leicester, UK

*Journal of Cardiothoracic Surgery* 2023, **18(Supp 1)**:A42

**Objectives**

We investigated the impact of frailty on survival and revascularisation rates in patients with acute coronary syndrome (ACS) and whether frailty explained the regional differences in revascularisation rates.

**Methods**

Patients with an ACS diagnosis between 2010 and2015 were identified from Hospital Episodes Statistics (HES). Frailty was defined by the Hospital Frailty Risk Score (HFRS) using HES records within two years prior to ACS diagnosis. All-cause mortality at 1 and 5 years were compared among patients with low (HFRS < 5), mid (5–15) and high (15 +) frailty scores. Regional revascularisation rates by Clinical Commissioning group (CCG) adjusted for frailty and patient factors including age, sex, ethnicity, and comorbidities were investigated using funnel plots.

**Results**

The final cohort included 1,422,004 ACS patients of whom 3.5% had a high frailty risk and 10.1% an intermediate frailty risk. ACS patients with a high frailty risk had higher mortality during the year after their ACS event (low risk: 12.4%, intermediate risk: 34.5%, high risk: 48.2%, p < 0.05). Patients with high frailty were 84% (CI: 83%-86%) less likely and intermediate risk 67% (CI: 66%-69%) less likely to receive a revascularisation procedure compared to low risk patients. 6 out of 210 (2.3%) CCGs had a standardised ratio which was outside the limits of the 99.8% confidence interval.

**Conclusion**

Frailty is associated with poorer survival and a difference in revascularisation rates in ACS patients. Regional variation in revascularisation persisted after adjustment for frailty and other patient factors suggesting unwarranted variation in the provision of care for ACS patients.

### A43 Predictors of Permanent Pacemaker Implantation Following Coronary Artery Bypass Graft, a 20 Year Experience of a Single UK Centre

#### Leone, Francesca, Dr; Elshafie, Ghazi, Mr; Loubani, Mahmoud, Prof

##### Hull University Teaching Hospitals, Hull, UK

*Journal of Cardiothoracic Surgery* 2023, **18(Supp 1)**:A43

**Objectives**

To identify predictors of permanent pacemaker implantation (PPMI) following coronary artery bypass graft (CABG) over the last 20 years.

**Methods**

We undertook a retrospective review of all patients who underwent isolated CABG between 1999 and 2020 in a single UK centre, excluding patients with preoperative complete heart block or pacemaker in situ, and identified those who had PPMI in hospital postoperatively. The data was taken from a large hospital registry. We analysed the data using IBM SPSS Statistics Version 27.

**Results**

7881 patients had CABG and did not require PPMI after surgery (CABGA only group), 67 patients (0.85%) required PPMI after surgery. Predictors for PPMI post CABG were preoperative arrythmia (p < 0.001), number of previous myocardial infarction (MI) (p < 0.001), post-operative MI (p < 0.001), unstable angina within 30 days (p < 0.001) and the extent of coronary artery disease (p = 0.025).

**Conclusions**

Patient who are at high risk of PPMI post CABG are patients with preoperative arrythmia, previous MI, post-operative MI, preoperative unstable angina and the patient with complex coronary artery disease. We recommend that this risk should be addressed in the preoperative assessment and to be included in the consenting process.
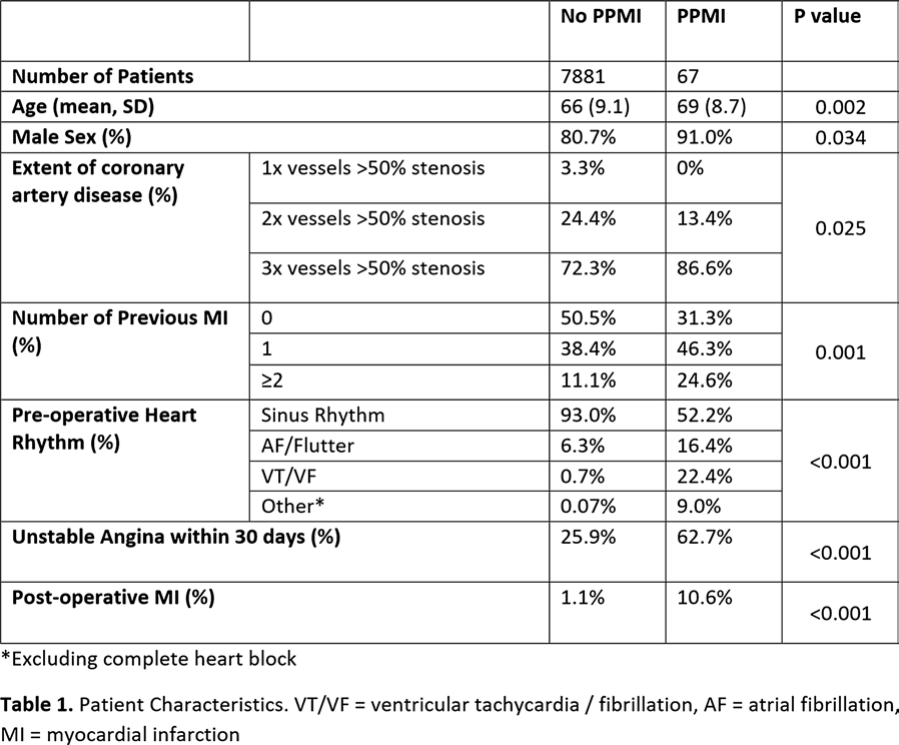


### A44 Management of Anomalous Right Coronary Artery Arising From Left Sinus of Valsalva with Interarterial Course—Movie

#### Divya, Aabha, Dr; De Silva, Ravi, Mr

##### Royal Papworth Hospital NHS Trust, Cambridge, UK

*Journal of Cardiothoracic Surgery* 2023, **18(Supp 1)**:A44

https://www.youtube.com/watch?v=sFr6J6ACeqI

### A45 Stuck on You; Entrapment of a Coronary Catheter Following Previous Transcatheter Aortic Valve Replacement

#### Ike, David Ikenna, Dr; Balmforth, Damian, Mr; Jarral, Omar, Mr; Roberts, Neil, Mr

##### St Bartholomew's Hospital, London, UK

*Journal of Cardiothoracic Surgery* 2023, **18(Supp 1)**:A45

**Introduction**

The number of patients undergoing percutaneous coronary intervention (PCI) post-TAVI is increasing. Entrapment of diagnostic catheters is a rare complication that presents significant challenges to clinicians.

**Results**

We present a case of an 89-year-old lady who underwent a coronary angiogram post TAVI which was subsequently abandoned due to entrapment of the JL catheter. Multiple manoeuvres by the interventional team to dislodge the JL catheter proved unsuccessful. A subsequent multi-disciplinary team (MDT) discussion deemed surgical retrieval of the retained catheter with cardiopulmonary bypass high risk due to her advanced age and co-morbidities.

A decision was made to perform a right brachial arteriotomy and the proximal portion of the catheter lying in the radial artery was excised. This allowed flexion of the arm at the elbow but the distal remnant of the catheter between the ascending aorta and mid-brachial artery remained in-situ. The thrombosis risk of the catheter remaining in-situ was mitigated by the commencement of a direct oral anticoagulatant. After five-months of follow-up, the patient reports no neurovascular abnormalities or symptoms associated with either the surgery or the retained catheter.

**Conclusion**

As TAVI expands into the low-surgical risk cohort, such complications may be expected to become more common place. The development of both TAVI and catheter technology should be focused towards reducing this complication. In the absence of an existing evidence base, the MDT can provide essential guidance on the best treatment options on a case-by-case basis.

Informed consent to publish had been obtained.

### A46 Endoscopic Radial Artery Harvesting Without Using a Tourniquet is Feasible

#### Joseph, Benny, Mr; AlShiekh, Mahmoud, Dr; Petrou, Mario, Dr; De Robertis, Fabio, Dr; Bahrami, Toufan, Dr; Gaer, Jullien, Dr; Bhudia, Sunil, Dr; Raja, Shahzad, Dr; Stock, Ulrich, Prof; Smail, Hassiba, Dr; Khoshbin, Espeed, Dr

##### Harefield Hospital, Uxbridge, UK

*Journal of Cardiothoracic Surgery* 2023, **18(Supp 1)**:A46

**Background**

The endoscopic radial artery harvesting results in good cosmetic results and minimal neuralgias. It is also more acceptable to the patients therefore better than traditional open technique. The procedure is typically performed using a tourniquet pressurized to 250 mmHg for the duration of radial harvest. We present our experience of endoscopic radial harvesting with and without the use of a tourniquet.

**Methods**

This is a cross-sectional single institutional study of endoscopic radial artery harvesting with or without the use of external arm tourniquet. The study period was between June 2016–October 2021. Since January 2018 all endoscopic radial artery harvesting was performed without the use of tourniquet. The harvest was performed by the same surgical practitioner. Data was collected and analysed using an excel programme.

**Results**

There were 131 endoscopic radial artery harvests. The proportion of cases done without vs with tourniquet was 101 to 30 respectively. There were no incidents of reopening for bleeding or haematoma in either group. There were no incidents of radial artery damage. The visibility in each method was adequate however slightly better when a tourniquet was applied. Furthermore, there was no time pressure for harvesting the conduit within twenty mins as is the case when tourniquet is used.

**Conclusion**

Harvesting of radial artery endoscopically without external tourniquet is feasible and produces comparable results with the technique using tourniquet in this series.

### A47 Ultra-fast Track Protocol after Left Mini-thoracotomy OPCAB

#### Guida, Gustavo^1^, Dr; Bruno, Vito D^1^, Dr; De Garate, Estefania^1^, Dr; Dixon, Lauren^1^, Ms; Di Tommaso, Ettorino^1^, Dr; Angelini, Gianni^1^, Prof; Guida, Maximo^2^, Prof

##### ^1^Bristol Heart Institute, Bristol, UK, University Hospitals Bristol NHS Foundation Trust, Bristol, UK; ^2^Fundacardio charity, Valencia, Venezuela

*Journal of Cardiothoracic Surgery* 2023, **18(Supp 1)**:A47

**Objectives**

Ultra-fast track (UFT) is a protocol with potential benefits to reduce the length of stay of patients after CABG.

The aim of this study was to identify potential predictors of delayed discharge for UFT in patients undergoing left mini-thoracotomy OPCAB.

**Methods**

Retrospective single-center cohort analysis of 1095 consecutive patients who underwent left mini-thoracotomy OPCAB (870 male, 79.2%, mean age 62 ± 9) from 2002 to 2017. The average number of grafts was 2.89 ± 0.99, and the mean EuroSCORE was 3.48 ± 2.97. A postoperative hospital length of stay < 48 h, was defined as UFT and > 48 h non-UTF. A multiple logistic regression model was developed to identify preoperative factors affecting UTF.

**Results**

530 (48%) patients were discharged within 48 h (UTF group). Overall mean ITU stay was 21.79 ± 4.83 h for the UFT group and 24.88 ± 6.88, for the non-UFT group (p > 0.001). There was no difference in terms of average number of bypass grafts between groups (p = 0.7). Seven patients required re-exploration (1.3%) in the UFT group, and 15 in the non-UFT (2.7%, p = 0.12). Post operative atrial fibrillation was three times more frequent in the non-UFT than the UFT patients 3.8% vs 1.4% (p = 0.03). At multivariable analysis factors associated with delayed discharge in UFT group were preoperative NYHA class, preoperative smoking, and EuroSCORE.

**Conclusions**

UTF is a feasible strategy for left mini-thoracotomy OPCAB. NYHA class, smoking status, and EuroSCORE are independent predictors of UTF.

### A48 Post Infarction Left Ventricular Aneurysm Repair

#### Mayooran, Nithiananthan, Mr; Jakub, Marczak, Mr; Tyson, Nathan, Mr; Apicella, Giulia, Ms; Abbas, Sherif, Mr; Qureshi, Saquib, Mr; Boulemden, Anas, Mr; Birdi, Inder, Mr; Szafranek, Adam, Mr; Naik, Suren, Mr

##### Nottingham City Hospital, Nottingham, UK

*Journal of Cardiothoracic Surgery* 2023, **18(Supp 1)**:A48

**Introduction**

Surgical remodelling of Left ventricular aneurysm secondary to ischemic heart disease remains a viable option since 1950’s. Surgical ventricular restoration (SVR) reduces LV volume and creates a more helical chamber by excluding scar in akinetic segments as well as restores intrapapillary diameter which can improve cardiac function, thus improving symptoms and life expectancy. We aim to report our 25 years’ experience of LV aneurysm repair and in-hospital outcomes.

**Methods**

In this retrospective study, we analysed patients’ demographics, preoperative variables including echocardiographic findings, intraoperative data as well as post-operative outcomes.

**Results**

We found from 1996 until 2021, 45 patients (male n = 32;female n = 13) underwent LV Aneurysm repair. Mean age was 66 years. Pre op LVEF was < 30% in 48.8% (n = 22) of the patients [BA(S1]. 40 out of 45 patients had concomitant CABG with the LVA repair, patients underwent aortic valve replacement and three patients underwent concomitant mitral valve replacement [BA(S2].

LVA repair comprised of either patch plasty (DOR procedure) n = 29, External plication repair n = 9 or linear excision and repair n = 7. In-hospital mortality was 8.3% (n = 4), including two patients who died from low cardiac output and two from multiorgan failure and 3 patients developed stroke. Intra-aortic balloon pump was utilised in 28 patients (intra op n = 8, post-op n = 2, pre-op n = 18) and 28 patients had follow up echocardiogram in our records [BA(S3]. Post-op LV EF% was poor in six patients, moderately impaired in 15 patients, mildly impaired in seven patients [BA(S4].

**Conclusion**

Surgical remodeling of the left ventricular aneurysm is a feasible therapeutic option for post-ischemic left ventricular aneurysm, with acceptable early results.

### A49 10-year Survival After Isolated CABG: Does the Presence of Left Main Stem Disease Have Impact on the Long-term Outcome?

#### Chan, Jeremy, Dr; Poon, Shi Sum, Mr; Cianci, Vincenzo, Mr; Ashraf, Syed, Prof; Bhatti, Farah, Prof; Youhana, Aprim, Mr; Zaidi, Afzal, Mr; Kumar, Pankaj, Mr

##### Morriston Hospital, Swansea, UK

*Journal of Cardiothoracic Surgery* 2023, **18(Supp 1)**:A49

**Introduction**

The impact of Left main stem (LMS) disease at the time of CABG on the long-term survival after CABG is unclear. The long-term follow-up for patients with LMS who have undergone coronary revascularisation remains scattered. We aim to report the 10 year follow-up results for patients who underwent isolated CABG with or without left main stem disease.

**Method**

We performed a single centre, retrospective study including all patients underwent isolated coronary artery bypass grafting (CABG) from 2006–2010. Patients were categorised into LMS or non LMS disease. The survival rate was reviewed and collected using the Welsh Clinical Portal. Kaplan–Meier log rank and Cox-proportional hazard analysis were plotted to demonstrate the 10-year survival rate between the two groups.

**Result**

One thousand three hundred fourteen patients underwent surgical revascularisation within the selected period. 260/1314 (19.79%) of the patients had LMS disease. The mean age was 66.30 and 65.91 for non-LMS and LMS group respectively. There is no difference between the need for transfusion requirement (p = 0.31). The 10-year survival rates for non-LMS and LMS patients were 69.1% and 64.2% (HR 1.19, 95%CI 0.95–1.50, p = 0.14). The trend in survival between the two groups appear to diverge after 8 years, although this failed to reach statistical significance.

**Conclusion**

Our data demonstrated no significant difference in the 10-year survival between patients with/without LMS disease. However, there is a trend towards lower survival in LMS group after 8 years. CABG yields a good 10-year survival rate in all patients and long-term outcomes should be taken into consideration in patient needing myocardial revascularisation.
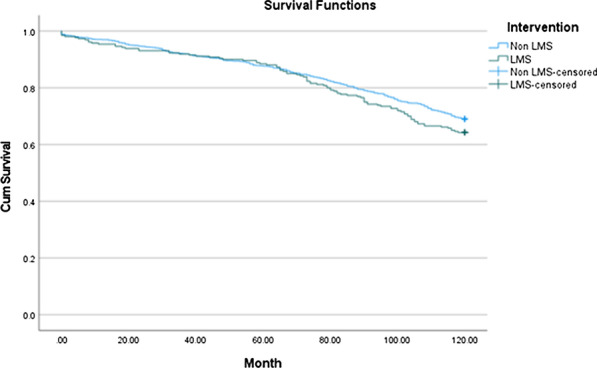


### A50 The Impact of Red Blood Cells Transfusion on Long-Term Survival After Isolated Coronary Artery Bypass Grafting (CABG)

#### Poon, Sam, Mr; Chan, Jeremy, Mr; Cianci, Vincenzo, Mr; Sharma, Sobaran, Mr; Kumar, Pankaj, Mr

##### Morriston Hospital, Swansea, UK

*Journal of Cardiothoracic Surgery* 2023, **18(Supp 1)**:A50

**Objective**

Allogenic blood transfusion in cardiac surgery is associated with adverse outcomes. We investigated the impact of blood transfusion on long-term survival after isolated CABG.

**Method**

A retrospective cohort study on 1546 consecutive patients who underwent isolated CABG from October 2007 to October 2011 was undertaken. The mean duration of follow-up is 10 years. The unit of blood transfusion was categorized into 5 groups. No transfusion, 1 unit, 2–4 units, more than 5 units, more than 10 units in order to correlate the survival outcomes based on the units of blood transfusion received. A Kaplan–Meier survival curve, log-rank analysis, logistic regression and Cox proportional hazard analysis were conducted with propensity-matched analysis.

**Results**

One thousand five hundred forty-seven were included in the study. The mean age was 66.24 (range 26–89). Overall the 1,5,10-year survival rate was 95.51%, 87.52% and 68.07%, respectively for the entire cohort. Patient who received 2–4 units (HR 1.344, 95% CI 1.083–1.668, p = 0.007), > 5 units (HR 2.46, 95% CI 1.70–3.55, p < 0.001) and > 10 units (HR: 56.08, 95% CI: 25.73–122.23, p < 0.001) of RBC transfusion have a significantly worsen 10-year survival outcome when compared with patient received no transfusion. There was no difference in survival between who received no and 1 unit of red blood cell transfusion.

**Conclusion**

In patients who underwent isolated CABG, transfusion of two or more units of red blood cells is associated with poorer survival at 10 years. Effort should be therefore be made to minimize red blood transfusion after CABG.
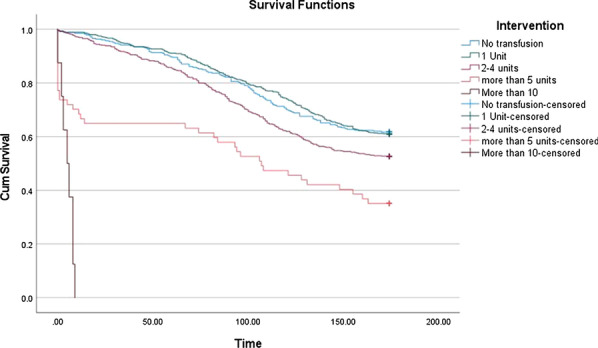


## Adult Cardiac Miscellaneous

### A51 Minimally Invasive versus Conventional Sternotomy for Primary Benign Cardiac Tumours: A Meta-analysis

#### Hussain, Azhar, Mr; Mittal, Aaina, Ms; Uzzaman, Mohsin, Mr; Iqbal, Yasir, Mr; Butt, Salman, Mr; Deshpande, Ranjit, Mr; Khan, Habib, Mr

##### Kings College Hospital, London, UK

*Journal of Cardiothoracic Surgery* 2023, **18(Supp 1)**:A51

**Objective**

The contemporary trend towards a more minimally invasive approach in general cardiac surgery have extended to involve the resection of primary benign cardiac tumours, particularly in the hands of experienced surgeons. We performed a meta-analysis of comparative studies using both approaches to highlight the differences in outcomes for primary benign cardiac tumour surgery.

**Methods**

A literature search was performed using PubMed, EMBASE and Google Scholar until January 2021. 12 publications were analysed, including a total of 877 patients in this meta-analysis. 359 (40.9%) had a minimally invasive (MT) approach compared to 518patients (59.1%) who had a median sternotomy (MS) approach during primary benign cardiac tumour surgery. The outcomes analysed include mortality, post-operative stroke, renal failure, atrial fibrillation (AF), length of hospital stay, reoperation for bleeding, wound infection, cardiopulmonary bypass (CPB) times, cross-clamp times, transfusion of red blood cells (RBC), intubation, chest drainage, and Intensive Therapy Unit (ITU) stay.

**Results**

There was a significantly reduced length of hospital stay, ITU stay, RBC transfusion, and post-operative AF with the minimally invasive approach, but increased CPB and cross-clamp time, when compared with median sternotomy.No significant difference was found in mortality or incidence of post-operative stroke.

**Conclusion**

Surgical resection of primary benign cardiac tumours via a minimally invasive approach is a safe and effective method with comparable outcomes. ICU stay, post-operative length of hospital stay and the need for reduced transfusion requirements are potential benefits with this approach.
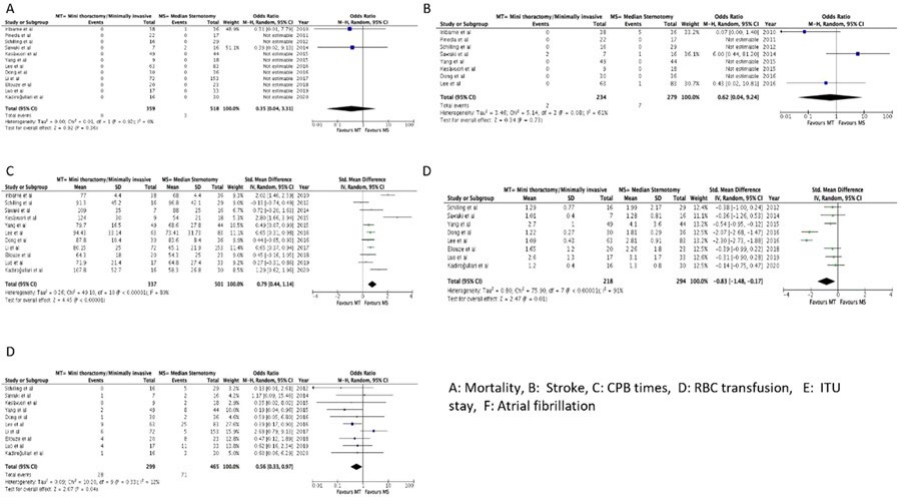


### A52 Investigating the Factors That Influence and Deter Medical Students from Pursuing Cardiothoracic Surgical Career

#### Sherif, Mohamed^1^, Mr; Linehan, Kathrine^2^, Prof

##### ^1^Leeds General Infirmary, Leeds, UK; ^2^University of Sheffield, Sheffield, UK

*Journal of Cardiothoracic Surgery* 2023, **18(Supp 1)**:A52

**Objectives**

To investigate what factors influence and deter medical students from pursuing a Cardiothoracic surgical career. Also, to find out the most popular career among our medical students and the percentage of students who wants to pursue a surgical career.

**Method**

Survey consisted mainly of open-ended questions were distributed among medical students in clinical years at the University of Sheffield (phase 2 b to phase 4) and experienced Cardiothoracic Surgeons at Northern General Hospital. Qualitative and quantitative data were collected and analysed statistically with SPSS version 27 and thematically.

**Outcome**

52 (7.1%) students and 13 consultants (22%) responded to the survey. Only ten students (19%) were interested in pursuing a surgical career, only one student (1.9%) expressed their interest in pursuing a Cardiothoracic career. There was no statistical difference between gender, age, ethnicity, year of study and the plans to join a surgical job. Three themes emerged as factors that influence students to a career in surgery: personal experience, speciality related factors, and rewards. These themes contain subthemes where we found early positive exposure to surgery as the most critical factor in pursuing a surgery career. In contrast, work-life balance was the main discouraging factor for surgery.

**Conclusion**

Our study identified three thematic factors that influence students career choices (personal experience, speciality related factors and Rewards). Cardiothoracic centres can facilitate more positive exposure to medical students to drive them toward the speciality. Focusing on providing early surgical exposure, defining more role models and highlighting intellectual and financial rewards of Cardiothoracic surgery are the keys to attracting more medical students toward the speciality.

### A53 Establishing a Video Education Library for Mitral Valve Repair: What do Trainees Think?

#### Casey, Anna^1^, Ms; Ahmed, Ishtiaq^2^, Dr

##### ^1^Brighton and Sussex Medical School, Brighton, UK; ^2^Royal Sussex County Hospital, Brighton, UK

*Journal of Cardiothoracic Surgery* 2023, **18(Supp 1)**:A53

**Objectives**

E-learning is increasingly used in surgical training but there is a lack of understanding about the needs of local cardiothoracic surgery (CTS) trainees with regards to this modality.

We aimed to understand the needs of local CTS trainees, and in future use this to develop a tailored video library of CTS procedures to improve the quality of CTS training.

**Methods**

Online questionnaires and invitations to interview were sent to all 36 CTS trainees in the South East/London area. These collected quantitative and qualitative data which was analysed using descriptive statistics and implementation theory.

**Results**

Of 36 trainees, 17 responded to the questionnaire, and 3 interviews were conducted. 8 trainees were in their ST8 year of training. Trainees felt that their use of E-learning had increased due to the Covid-19 pandemic and felt positively about the development of a new video library.

Trainees reported a rise in demand for E-learning resources over recent years, accelerated by the pandemic, which has not been matched by UK-specific development. Key features that trainees would value included: offline mobile access, explicit linkage to UK training outcomes, and self-testing capacity. Trainees reported using other video libraries, but these were linked to requirements in the United States, revealing a needed gap in the E-learning landscape for UK CTS training. Mitral valve repair was a key procedure which trainees felt would benefit from the video library format.

**Conclusions**

This project aimed to understand the potential role of a new E-learning video library resource for CTS trainees, and to describe their opinions and attitudes towards this. Our results suggest that there is a definite gap in the market for a UK-based CTS E-learning resource, especially with the impact of Covid-19 on training. Further exploration of national CTS trainees’ opinions is needed, as well as further understanding of ways to meet CTS training outcomes with E-learning modalities.

### A54 Infrastructural Failure and Equipment Malfunctions Occurring During Elective Cardiac Surgery. A Three-year Prospective Study

#### Efthymiou, Chris, Mr

##### Glenfield Hospital, University Hospitals of Leicester NHS Trust, Leicester, UK

*Journal of Cardiothoracic Surgery* 2023, **18(Supp 1)**:A54

**Objectives**

A cardiac operation can only commence after a Surgical Safety Checklist (NHS) is performed. The checklist was launched by the World Health Organisation (WHO) in 2008 and utilises a 3-time point pause where changes can be made before it’s too late.

There is however no auditing of infrastructure or surgical instrument failures occurring during procedures and little data exists regarding the effects of malfunctions. Owing to this lack of information we studied the frequency and classified the category of intraoperative equipment malfunction occurring during cardiac procedures.

**Methods**

Over a 36-month period equipment malfunctions were recorded during each procedure. Operating equipment was divided into 3 categories based on portability and function. Group 1: Theatre infrastructure and components. Group 2: Large medical equipment. Group 3: Surgical instruments.

**Results**

In 75% (196/260) of cases there was an issue with equipment failure occurring. Theatre infrastructure failures (Group 1) resulted in the cancellation of 5 cases (2%). Malfunctioning large equipment (Group 2) occurred in 2.3% of cases and included issues such as broken operating tables or TOE malfunction. In the surgical instrument category (Group 3), issues with malfunctioning needle holders accounted for the bulk of failures recorded by the study (60%). Some intraoperative instrument failures such as spontaneously closing retractors were potentially catastrophic.

**Conclusions**

Failure of infrastructure or equipment occurs in an unacceptably high proportion of cases. Some issues are overtly dangerous while others affect the flow of an operation. The most common failures are with needle holders, forceps and scissors. Preventative maintenance of key instruments and infrastructure should therefore be undertaken to prevent cancellation of cases and to reduce adverse events from occurring during a procedure.

### A55 A Survey of the Run-through Training Programme in Cardiothoracic Surgery in Great Britain and Northern Ireland Between 2013–2018

#### Dawson, Alan G.^1^, Mr; Tyson, Nathan J.^2^, Mr; Tan, Carol^3^, Ms; Rathinam, Sridhar^1^, Mr; Jahangiri, Marjan^3^, Prof

##### ^1^Glenfield Hospital, University Hospitals of Leicester NHS Trust, Leicester, UK; ^2^Nottingham City Hospital, Nottingham, UK; ^3^St George's University Hospitals NHS Foundation Trust, London, UK

*Journal of Cardiothoracic Surgery* 2023, **18(Supp 1)**:A55

**Objectives**

The Cardiothoracic Surgery run-through training programme was initiated in August 2013 in Great Britain and Northern Ireland. We aimed to evaluate the experiences of a cohort of trainees in the Cardiothoracic Surgery run-through training programme to identify its strengths and weaknesses.

**Methods**

All trainees in Cardiothoracic Surgery who were appointed at ST1 from 2013 to 2018 were invited to complete an electronic survey (SurveyMonkey). The survey comprised 69 questions and was endorsed by the SCTS and SAC in Cardiothoracic Surgery. The link to the survey was disseminated by the SCTS through Isabelle Ferner from 01 September 2020 and 31st October 2020 with weekly reminders sent. Five sections were covered in the survey: trainee demographics; ST1 application; early ST1/ST2 years; after ST2; and reflections on ST1 run-through training. Data was analysed using SPSS Version 26.0.

**Results**

Between 2013–2018, 46 trainees were appointed to the Cardiothoracic run-through programme and 34 responses were received (response rate of 74%). The majority of trainees were male (65%) with a median age of 27 years. Two-thirds decided on a Cardiothoracic career in medical school and 22 had completed an undergraduate BSc degree. The majority of trainees (41%) entered run-through from FY2. A Cardiothoracic Educational Supervisor was assigned to 88% and 81% of ST1 and ST2 trainees, respectively. The time spent in adult cardiac, thoracic, congenital and transplant was 16, 8, 6 and 9 months, respectively. The majority of respondents felt that ST1/2 prepared them well for ST3 + training and felt supported in their training. There were concerns raised regarding post-CCT employment.

**Conclusions**

This comprehensive survey of Cardiothoracic run-through training has shown that the programme prepares trainees well for ST3. Furthermore, the results of this survey will provide valuable information and guidance for the SCTS and SAC on ways that the run-through programme can be developed.

### A56 Warfarin Prescribing at a Tertiary Cardiac Unit – Why is One of the Cheapest Drugs So Expensive?

#### Yap, Trixie, Miss; Shehata, Monicka, Dr; Rizzo, Victoria, Miss; Hafiz, Imran, Mr; Avlonitis, Vassilios, Mr

##### Guy's & St Thomas' NHS Foundation Trust, London, UK

*Journal of Cardiothoracic Surgery* 2023, **18(Supp 1)**:A56

**Objectives**

Cardiac surgery patients are often discharged on warfarin due to valve procedures or atrial fibrillation. Despite the high volume of prescriptions, it appears we often get it wrong. The objective was to establish the cost implications of incorrect dosing and excess INR tests, and identify how to improve this service for our patients.

**Methods**

A retrospective analysis of prospectively collected database was carried out. All adult cardiac inpatients prescribed warfarin between 1st November – 31st December 2020 were included. Patient records were assessed for warfarin dosing errors on a predetermined proforma. The number of coagulation tests per patient was also analysed.

**Results**

A total of 29 patients were included in the analysis. Warfarin was started approximately 2.9 (± 4.26) days after surgery in intensive/high dependency care. 125 dosing errors (median 3 per patient, range 0–22) were identified (Fig. 1). There were 107 (18%) excess INR tests costing £1208.03 and 20 (mean 4 ± 2.23) excess inpatient bed days (approximate cost £8000) for 5 patients awaiting INRs to become therapeutic.
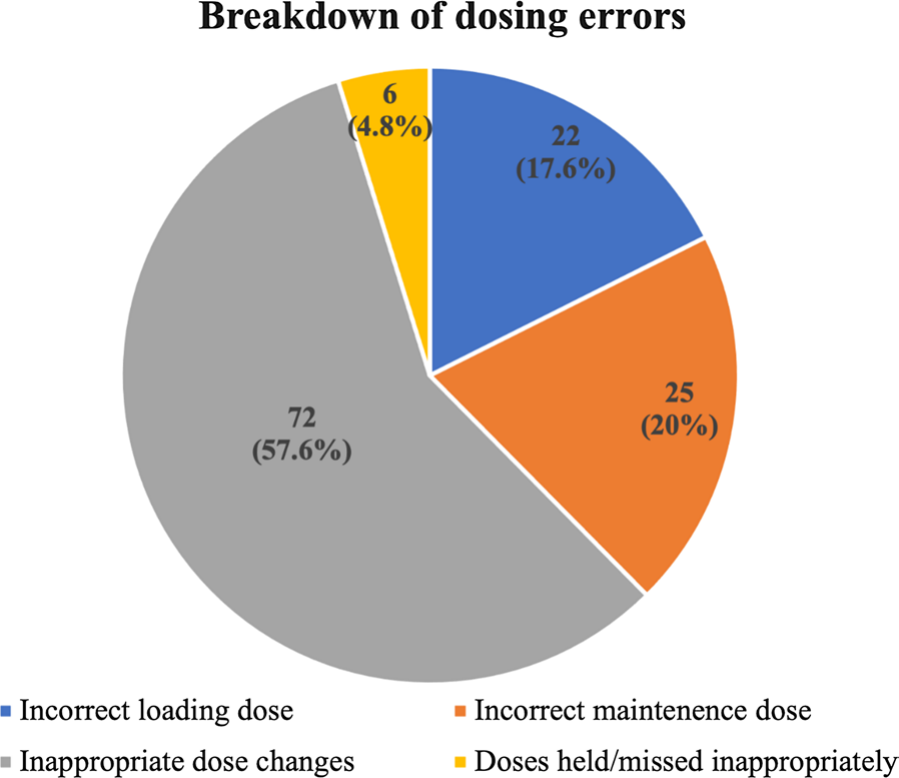


**Figure 1**

**Conclusions**

Excess dosing errors highlight poor understanding of the pharmacokinetics of warfarin, leading to excessive INR testing and prolonged patient stay, generating significant cost. Widespread education for prescribers, as well as pharmacy-led prescribing, may be the answer.

### A57 Safety of Training – A Propensity Matched Analysis of the UK National Database

#### Sinha, Shubhra^1^, Miss; Dimagli, Arnaldo^2^, Mr; Fudulu, Daniel^2^, Mr; Bruno, Vito Domenico, ^2^, Mr; Chan, Jeremy^2^, Mr; Vohra, Hunaid^2^, Mr; Benedetto, Umberto^2^, Prof; Angelini, Gianni D^2^, Prof

##### ^1^Derriford Hospital, Plymouth, UK; ^2^Bristol Heart Institute, Bristol, UK

**Objective**

*Journal of Cardiothoracic Surgery* 2023, **18(Supp 1)**:A57

Training the next generation of cardiac surgeons is a challenging aspect of surgical practice.

Increased scrutiny of individual results and alterations in patient profiles and trainee working patterns may have impacted on training and post-operative outcomes. We conducted an analysis of a large national database to compare outcomes between cases performed by consultants and trainees.

**Method**

Retrospective review of prospectively gathered database of adults undergoing cardiac surgery in the UK between January 2012&March 2019.The primary outcome was mortality within the same admission or 30 days of surgery. Cases were propensity scored and matched 1:1 without replacement. Multivariable regression models for mortality were performed. Comparisons between procedure-specific outcomes were also made.

**Results**

During the study period 216,994 patients were operated upon. Of these 42,173(19.4%) were performed by trainees. Consultants when compared with trainees operated on patients with higher EuroSCORE2 (1.74 vs 1.26, p < 0.001), more emergency/salvage patients and those with more complex operations (mitral, aortic and double/triple/redo procedures). Trainees performed less off-pump and arterial grafting and less minimally-invasive valve procedures. The operative times were lower in trainee cases (CPB:91 vs 96 min, cross-clamp:59 vs 64 min). In the matched populations the overall mortality (1.3% vs 2.5%, p < 0.001) and need for dialysis (1.6% vs 2.8%,p < 0.001) was lower in trainee cases. The outcomes were equivocal for the incidence of stroke, sternal wound infection and length of stay post-operatively. Regression analysis showed a protective effect of trainee first-operators (odds ratio 0.5,95% confidence interval:0.45–0.56). Mortality was not influenced by the consultant nor hospital unit but operative times were shorter with consultants as the first-assistant.

**Conclusions**

Appropriate patient selection and supervision can render training in cardiac surgery safe and reproducible.

### A58 Rare LV Masses: Technical Challenges—Movie

#### Deglurkar, Indu, Mr; Syed Nong Chek, Syed Aidil Hizman, Mr; Karthikeyan, Sivagnanam, Dr

##### University Hospital of Wales, Cardiff, UK

*Journal of Cardiothoracic Surgery* 2023, **18(Supp 1)**:A58

https://www.youtube.com/watch?v=h8vydxPOU2w

### A59 Comparison of Machine Learning Techniques in Prediction of In-Hospital Mortality Following Cardiac Surgery: Analysis of Over 220,000 Patients

#### Sinha, Shubhra^1^, Miss; Dong, Tim^2^, Mr; Dimagli, Arnaldo^3^, Mr; Vohra, Hunaid^3^, Mr; Sterne, Jonathan^2^, Prof; Angelini, Gianni D^3^, Prof; Benedetto, Umberto^3^, Prof

##### ^1^Derriford Hospital, Plymouth, UK; ^2^University of Bristol, Bristol, UK; ^3^Bristol Heart Institute, Bristol, UK

*Journal of Cardiothoracic Surgery* 2023, **18(Supp 1)**:A59

**Objectives**

Comparative study of statistical techniques for formulation of an in-hospital mortality risk stratification tool for adults having cardiac surgery in the UK based. The purpose of this study was to perform a thorough systematic comparison between the predominant scoring system in use [i.e. European System for Cardiac Operative Risk Evaluation (ES)II], logistic regression retrained on the present database and alternative machine learning techniques–namely random forest (RF), neural networks(NN), XGBoost and weighted support vector machine.

**Methods**

We conducted retrospective analyses of prospectively routinely gathered data on adult patients undergoing cardiac surgery in the UK between January 2012 and March 2019.We temporally split the data 70:30 into training and validation subsets. Mortality prediction models were created using the aforementioned techniques utilising the 18 variables for EuroSCORE II. Comparisons of discrimination, calibration and clinical utility were then conducted. We also reviewed changes in model performance over time.

**Results**

Of the 227,087 patients there were 6,258 deaths (mortality 2.83%). In the testing cohort, we noted an improvement in discrimination XGBoost (AUC:0.8337–0.8343, F1 score:0.276–0.280) and RF (AUC:0.833–0.834,F1:0.277–0.281) compared with ESII (AUC:0.817–0.818,F1:0.252–0.255). There was no significant improvement in calibration with machine learning and retrained logistic regression as compared with ESII, but ESII overestimated risk across all deciles of risk and over time. The calibration drift was lowest in NN, XGBoost and RF compared with ESII. Decision curve analysis showed XGBoost and RF to have greater benefit than ESII.

**Conclusions**

Machine learning techni showed statistical improvements in discrimination, calibration drift and accuracy compared with ESII. Further analysis incorporating a wider range of predictor variables would be beneficial.

### A60 A Single-centre 21st Century Experience of Triple Valve Surgery

#### Raj Krishna, Gokul, Mr; Taylor, Marcus, Mr; Nwaejike, Nnamdi, Mr; Barnard, James, Mr; Venkateswaran, Rajamiyer, Prof

##### Wythenshawe Hospital, Manchester, UK

*Journal of Cardiothoracic Surgery* 2023, **18(Supp 1)**:A60

**Objectives**

Triple valve surgery has previously been shown to be associated with high rates of mortality and morbidity. Nevertheless, patients requiring concomitant intervention on three valves represent an important subgroup of cardiac surgery patients for whom alternative non-surgical options may not be suitable. We aim to review our contemporary experience of triple valve surgery.

**Methods**

A total of 29 consecutive patients undergoing triple (concomitant aortic, mitral and tricuspid) valve surgery at a single quaternary cardiac surgery centre between 2003 and 2020 were included. Primary outcomes were in-hospital mortality, post-operative complications, post-operative length of stay (PLOS) and 1-year mortality.

**Results**

The mean age was 63.0 years (± 9.5 years) and 65.5% (n = 19) of patients were male. In total, 20.7% (n = 6) underwent urgent surgery and 79.3% (n = 23) had surgery on an elective basis. Overall, 31.0% (n = 9) patients underwent an additional concomitant procedure (coronary artery bypass grafting [n = 3], aortic surgery [n = 1], atrial fibrillation surgery [n = 5]). The mean cardiopulmonary bypass and cross-clamp times were 189.4 min (± 47.7 min) and 136.1 min (± 37.4 min), respectively. Mean logistic Euroscore was 7.4% (± 5.1%). Post-operative complications were experienced by 69.0% (n = 20) of patients and the median PLOS was 15 days (IQR 9–21 days). In-hospital and 1-year mortality were 6.9% (n = 2) and 10.3% (n = 3), respectively.

**Conclusion**

Despite a high rate of post-operative morbidity, the observed in-hospital mortality rate was relatively low and broadly similar to the expected mortality rate, as calculated by the Euroscore. For those patients surviving to discharge, the risk of death up to 1 year after surgery is also minimal. These results demonstrate that triple valve surgery remains an appropriate cardiac surgical intervention in carefully selected patients.

### A61 Cardiac Surgery in the Over in the Over 85 Population: A Single Centre Retrospective Cohort Analysis

#### Badran, Abdul^1^, Dr; Shah, Owais^1^, Dr; Badran, Dania^2^, Dr; Velissaris, Theodore^1^, Mr

##### ^1^University Hospital Southampton, Southampton, UK; ^2^Imperial College London, London, UK

*Journal of Cardiothoracic Surgery* 2023, **18(Supp 1)**:A61

**Objectives**

Rising average life expectancy in the developed world, increased incidence of cardiovascular disease with age and improved peri and postoperative care has expanded provision of cardiac surgery to an increasingly elderly population. We evaluated the outcomes of cardiac surgery in this high-risk population.

**Methods**

We retrospectively investigated patient characteristics and outcomes of those over the age of 85 years who underwent a cardiac surgical procedure over an 18-year period at our busy cardiac surgical unit.

**Results**

The total number of patients 558 with a mean age of 88 years (86–94). 323 of these patients were male with 235 female. Surgical coronary revascularisation was performed in 54% (304) with an average of 2 grafts (5–1). 70% (390) underwent an aortic valve replacement, 8% (n = 44) had a mitral valve repair/replacement and 3% (n = 15) had a tricuspid valve repair /replacement. An aortic interposition graft was performed in 1% (n = 8). Premorbidites included 44% (n = 244) were hypertensive, chronic kidney injury in 13% (n = 73), history of CVA in 8% (n = 46), 8% (n = 46) had COPD, 8% (n = 49) were diabetic and. Postoperative complications included chest infection in 20% (n = 110) chest infections, 3% (n = 16) wound infections, 16% (n = 90) developed an AKI with 5.4% (n = 30) on a background of chronic renal disease., 6.8% (n = 38) had re-exploration for post-operative bleeding.

**Conclusions**

Cardiac surgery in this patient group is safe providing definitive, reproducible results with acceptable mortality figures. Age and certain comorbidities should not be a deterrent in offering surgery to this growing cohort.

### A62 Retrograde Arterial Perfusion is Safe for Endoscopic Heart Valve Surgery in Both Young and Elderly Patients

#### Elhassan, Hind, Dr; Abdelbar, Abdelrahman, Mr; Zacharias, Joseph, Mr

##### Blackpool Teaching Hospital, Blackpool, UK

*Journal of Cardiothoracic Surgery* 2023, **18(Supp 1)**:A62

**Objective**

Minimal invasive cardiac surgery via right anterolateral thoracotomy compared with the conventional median sternotomy proven to have lower post-operative complications. We aimed to compare the neurological complications and post-operative outcomes in 2 cohort groups as well as mortality rate and survival rate up to 10 years post operatively.

**Method**

Retrospective observational study using propensity matched score for patients who had minimally invasive cardiac valve surgery between 2007–2021(n = 596) with retrograde femoral arterial perfusion.

**Result**

In our study, despite the significant difference between the two groups in their baseline individual characteristic and EuroSCORE, there were no differences between the two groups in term of post-operative outcomes both in unmatched and matched set. In data analysis we found that patients 70 years old or above had no increased risk for neurological complications (p = 0.75) compared with those below 70 years old. The same result was found after matching the data set (p = 0.60). Morality rate was also not significant between the two groups in unmatched data set (p = 0.12) and matched data set (p = 0.37). Although length of hospital stay was statistically significant in the unmatched data set (p < 0.001), there was difference between the two groups in matched data (p = 0.38). Elderly patient group do get discharged home the same as the adult group rather than been referred for further rehab or repatriated to another hospital for recuperation. (unmatched data p = matched data p = 32). Interestingly, the CPB time was significantly lower in the elderly group compared to the adult group (p = 0.036). This may be that particularly complex procedures were avoided in this age group.

The crude survival suggests no significant difference in survival rates between age groups when they are similar in term of basic characteristic.

**Conclusion**

Minimally invasive approach with retrograde arterial perfusion is a safe for a spectrum of primary cardiac valve procedure in elderly patients.
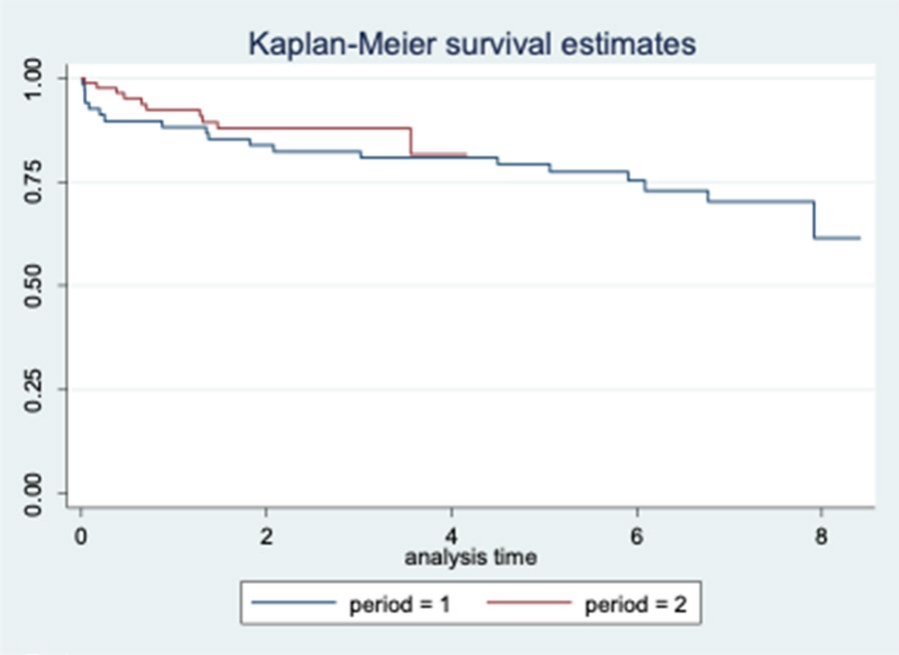


### A63 Experience and Outcomes of Redo Cardiac Surgery at a Tertiary Centre in the UK

#### Naruka, Vinci^1^, Mr; Chaubey, Sanjay^1^, Mr; Chacko, Jacob^1^, Mr; Liu, Guiqing^1^, Mr; Bola, Harroop^2^, Mr; Dixit, Prithvi^2^, Mr; Rathod, Virensinh^2^, Mr; Sabeshan, Pratheeshan^2^, Mr; Afoke, Jonathan^2^, Mr; Punjabi, Prakash P^1^, Prof

##### ^1^Hammersmith Hospital, London, UK; ^2^Imperial College London, UK

*Journal of Cardiothoracic Surgery* 2023, **18(Supp 1)**:A63

**Objective**

In recent years transcatheter interventions have increasingly been used to treat patients requiring repeat cardiac intervention. However, it is important that surgery is not disregarded as what maybe the best option for these patients. We report our experiences and outcomes of redo cardiac surgery over 9 years.

**Methods**

A prospectively collated database was analysed between 2013 and 2021 in a single centre. Outcomes were compared between the first 4.5 years to the latter 4.5 years. The primary outcome was in-hospital mortality. Secondary outcomes included stroke, new atrial fibrillation (AF), new pacemaker insertion, new renal replacement therapy (RRT), length of stay (LOS), and long-term mortality.

**Results**

166 patients underwent redo operations of which 13 (8%) had CABG, 38 (23%) AVR, 39 (23%) Aortic, 39 (23%) MV surgery, 24 (14%) Multi-valve, 13 (8%) CABG + Valve. The average EuroSCORE II was 8.31%, 34% were urgent, 19% for infective endocarditis.

Postoperative outcomes included: 3 (2%) stroke, 1 (1%) RRT, 25 (15%) AF, 10 (6%) pacemaker, LOS (10 days (7–15)), 15 (9%) hospital mortality, 62.5% alive at 3000 days.

Comparing the first 4.5 years to the second half, we performed significantly more redo-AVR (14% vs 30%), more redo-aortic (18% vs 28%), less redo-CABG (12% vs 4%) and redo-MV surgery (36% vs 13%) (p < 0.001). Our patient risk profile and operative strategy remained similar. We operated on more urgent patients (29% vs 38%,p = 0.42) with similar outcomes but less post-operative strokes (4% vs 0%,p = 0.05). There is significantly lower hospital mortality (15% vs 4%,p = 0.02) between the 2 periods, but long-term survival remains similar (p = 0.47) (Fig. 1.)

**Conclusions**

Over time, significantly lower hospital mortality and postoperative strokes were observed, while the overall long-term survival remains similar. Redo coronary and mitral valve surgery reduced across the study period whilst the proportion of redo aortic and aortic valve surgery increased.

### A64 Neurological Presentations in Endocarditis, a Cause of Delay to Surgery?

#### Badran, Abdul, Dr; Rowe, Henry, Mr; Badran, Dania, Dr; Nwakwu, Cynthia, Miss; Ohri, Sunil, Prof

##### University Hospital Southampton, Southampton, UK

*Journal of Cardiothoracic Surgery* 2023, **18(Supp 1)**:A64

**Introduction**

Infective Endocarditis can be a cause of cerebral emboli and mycotic aneurysms. Timing of surgery in patients with endocarditis who have suffered a cerebrovascular accident remains a matter of debate.

**Methods**

Data was collected retrospectively over a 4-year period and involved 198 patients. Demographic and clinical details were gathered from the patient medical records.

**Results**

A minority of patients presented with neurological symptoms 8.6% (n = 17). The majority of patients was male 82% (n = 14). The mean age was 55.4 (21–79). Of these patients 35% (n = 6) died and 65% survived to last follow-up. 65% (n = 11) were operated. The mean length of time waited before an operation was 17 days (0–51) Vs 11 days (0–62) in other presentations. Of the patients with neurological symptoms that had an operation, 27% (n = 3) died compared to 50% (n = 3) of those that did not. The survival rates for differing lengths of wait time for surgery were 80% for 7 days, 85.7% for £14 days, 75% for 28 days and 70% for 42 days.

**Conclusion**

Operating on IE within 14 days of admission provides the best survival outcomes for patients, even in recent haemorrhagic stroke. Surgical treatment is associated with a better survival rate than medical approaches.

### A65 A Systematic Review of Safety and Patient/Healthcare Provider Satisfaction with Virtual Clinics

#### Shehata, Monicka^1^, Dr; Rizzo, Victoria^2^, Miss; Sabetai, Michael^1^, Mr; Athanasiou, Thanos^3^, Prof

##### ^1^St Thomas Hospital, London, UK; ^2^Guy's and St Thomas' NHS Foundation Trust, London, UK; ^3^Hammersmith Hospital, London, UK

*Journal of Cardiothoracic Surgery* 2023, **18(Supp 1)**:A65

**Objectives**

Cardiac surgery outpatient appointments were conducted via telephone during COVID-19 in our unit. We have reviewed patient and healthcare provider satisfaction with virtual clinics in surgical specialities as a basis for a more permanent move to virtual clinics in cardiac surgery.

**Methods**

MEDLINE and Embase databases were searched for all studies evaluating virtual clinics for adult patients in surgical specialities within the past ten years. Qualitative analysis of overall patient satisfaction and safety was carried out. The Cochrane Risk of Bias Tool version 2 was used to assess risk of bias for randomised controlled trials.

**Results**

21 studies met the inclusion criteria: 16 cohort studies and 5 randomised controlled trials. 18 studies assessed patient satisfaction, two assessed healthcare provider satisfaction and one assessed both. Similar to our unit, the majority (57%) used virtual clinics for post-operative or follow-up patients. Number of patients per study ranged from 22 to 590, with a reported response rate of 20–100%. Patient and healthcare provider satisfaction ranged from 71.6%-98% and 76.5%-98% respectively. Only four studies used validated surveys, with 15 studies using study-specific surveys. Patient safety was assessed in 15 studies with no significant safety issues identified.

**Conclusions**

This review has helped to establish that virtual clinics are safe and acceptable to patients. Building on this framework, we will establish an ongoing virtual cardiac surgery service post-Covid. The use of validated questionnaires will help ongoing assessment of our service and identify contextual features that increase success of virtual clinics.

### A66 Readability of Cardiothoracic Surgery Consent Forms in Queensland

#### Dutta, Sanjay^1^, Dr; McManus, Bryan^2^, Dr; Iyer, Anand^1^, Dr

##### ^1^Princess Alexandra Hospital, Brisbane, Australia; ^2^St Vincent's Hospital, Sydney, Australia

*Journal of Cardiothoracic Surgery* 2023, **18(Supp 1)**:A66

**Objectives**

Written consent forms are a key component of obtaining informed consent from patients prior to surgery. Reading literacy among Australians is generally poor, and it is recommended that written content is at a year 7 level to make it usable for most people. The aim of this project was to assess the readability of consent forms for cardiac surgery in Queensland, Australia.

**Methods**

Queensland Health consent forms for "Aortic Surgery", "Coronary Artery Bypass Grafting and/or Valve Surgery", and "Generic Cardiac" were obtained. The readability of the sections relating to risks of surgery (Section C), patient consent (Section G), and the patient information sheet were assessed via an online readability software program, using five separate validated methods: (i) Flesch-Kincaid grade level, (ii) the SMOG (Simple Measure of Gobbledygook), (iii) Coleman-Liau index, (iv) Automated readability index, (v) Linsear Wriste formula. Statistical analysis was performed using Microsoft Excel.

**Results**

The mean ± standard deviation reading grade level from all algorithms for risks of surgery, patient consent, and patient information was 7.78 ± 2, 10.74 ± 0.76, and 7.83 ± 1.55 respectively. The "Generic Cardiac" form had the highest average grade level (9.8 ± 1.41), followed by "Coronary Artery Bypass Grafting and/or Valve Surgery" (8.67 ± 2.26) and "Aortic Surgery" (8.54 ± 2.18).

**Conclusions**

The readability of key sections of the Queensland Health consent forms is above the recommended reading grade, and a large percentage of the population would be unable to fully comprehend the information. Consideration should be made to lower the reading grade level of patient consent forms.

### A67 Carcinoid Heart Disease: Early Outcomes after Surgical Valve Replacement in Thirteen Patients

#### Mujtaba, Syed Saleem^1^, Mr; Clark, Stephen^2^, Prof

##### ^1^Morriston Hospital, Swansea, UK; ^2^Freeman Hospital, Newcastle, UK

*Journal of Cardiothoracic Surgery* 2023, **18(Supp 1)**:A67

**Objective**

To describe the early outcomes of carcinoid patients undergoing valve replacement.

**Methods**

In a retrospective study, patients with symptomatic carcinoid heart disease undergoing surgery between 2012 and 2021 were reviewed.

**Results**

13 patients (mean age 64 years (range 55–79 years) underwent surgery. Three patients had quadruple valve replacement, seven had tricuspid and pulmonary valves changed, two had tricuspid valve replacement, while one had tricuspid, pulmonary, and aortic valves replaced. Right-sided valves were replaced with biological valves in 12 patients and a mechanical valve in one patient. Left-sided valves were replaced with a mechanical valve in two patients and with a biological valve in 1 patient. Mean postoperative follow-up was 56 months (range 2-102 m, median 65 m). All had good left ventricular function except one (mildly impaired). The right ventricle was severely dilated in four patients, moderate in four, and mild in three. One patient died of heart failure 10 days postoperatively and one patient succumbed to acute carcinoid crisis 8 days after surgery. Functional improvement was noted in all survivors. All were NYHA class 1–2 at last follow up. None required a pacemaker. One patient died of their neuro-endocrine tumour at six years for a survival rate of 91%. No valve re-interventions were needed.

**Conclusion**

Carcinoid syndrome is a rare and progressive disease but valve replacement in symptomatic patients has functional and survival benefit, low early postoperative mortality, is without valve-related complications and shows functional improvement. Cardiac assessment is required in all patients with carcinoid disease to ensure that appropriate patients are put forward for surgery when symptomatic.

### A68 Effects of Inhibitor of –Catenin Responsive Transcription on Platelet-endothelial Interaction: A Molecular and Functional Study

#### Chan, Jeremy, Dr; Wadey, Kerry, Dr

##### University of Bristol, Bristol, UK

*Journal of Cardiothoracic Surgery* 2023, **18(Supp 1)**:A68

**Introduction**

Despite its high failure rate, saphenous vein graft (SVG) remains the most used conduit in coronary artery bypass grafting. Platelet activation and aggregation is involved in the early pathological pathway of SVG failure. Integrin αvβ3 was shown to be involved in platelet adhesion on the endothelial cell. Previous work showed Inhibitor of β–catenin responsive transcription (iCRT) improves endothelial barrier function and reduces monocytes recruitment in TNF α stimulated human umbilical vein endothelial cells (HUVECs). Its effect on platelet-endothelial interaction was evaluated to determinate the risk of thrombus formation prior further clinical use.

**Method and results**

Fluorescent labelled platelets were added into HUVECs with either unstimulated or stimulated with 10 ng/ml TNF-α and treated with either DMSO vehicle control or iCRT. iCRT significantly enhanced platelet-endothelial interaction in HUVECs. Similar results were seen in ex-vivo saphenous vein organ culture.

Further experiments were performed to seek for an explanation in a molecular level. Western blotting showed a reduction of integrin αv and free vWF in condensed media but not β3 in TNFα-stimulated HUVECs treated with iCRT. Quantitative PCR showed a raise integrin αv mRNA expression in TNFα-stimulated HUVECs with iCRT.

**Conclusion**

Our results demonstrated iCRT reduced protein expression of integrin αv and free vWF in condensed media in TNF-α-activated endothelial cells. However, such effects were not translated to functional assays. Nonetheless, I have reported a valid model and protocol for assessing platelet-endothelial interaction in HUVECs and ex-vivo saphenous vein organ cultures.

### A69 Are Super Obese Patients At Higher Risk for Cardiac Surgery? Results from the Last Decade

#### Mustafa, Ammar, Mr; Palima, Jeni, Ms; Hayre, Simran, Miss; Boateng, Michael, Mr; Elsiddig, Mahmoud, Mr; Rescigno, Giuseppe, Mr

##### Royal Wolverhampton NHS Trust, Wolverhampton, UK

*Journal of Cardiothoracic Surgery* 2023, **18(Supp 1)**:A69

**Objectives**

Very high BMI represents a concerning factor in Cardiac Surgery. However, previous studies have not shown a significantly increased risk. Yet, these were mainly focused on CABG operations and frequently with limited number of patients. The aim of this study was to review our in-hospital results of open-heart procedures for all comers with a BMI > 40 kg/m2 during the last 10 years.

**Methods**

Retrospective analysis of prospectively collected data of super obese patients who underwent open heart procedures (elective, urgent or emergency) of any kind between April 2011 and March 2020 in our Department. Participants’ demographics, preoperative risk factors, operative data, in-hospital mortality, postoperative complications were analysed.

**Results**

179 patients were identified. Patients preoperative characteristics are summarised in the Table. Briefly, the majority were male (56%) and Caucasians (91.6%). Mean age was 61.2 ± 9.4 years; mean SCTS Euroscore was 1.6 ± 4.1. The type of admission was elective in 65.9%, in-hospital transfer in 31.2% and emergency in 2.7%. The types of operations were isolated CABG (45.8%), isolated valve surgery (34.0%), CABG + Valve (12.8%), major aortic (3.9%) and others (3.3%). In hospital mortality was 1.6% (3 patients). Mean hospital stay was 8.4 ± 9.7 days. We recorded 2 permanent strokes (1.1%). Eight patients (4.4%) required temporary haemofiltration. In 14 patients (7.8%) some sort of ventilation support was necessary (CPAP, reintubation or both). There were 2 deep sternal wound infections (1.11%).

**Conclusions**

In-hospital results for super obese patients were good in our 10 years series. The dreadful sternal complications were not frequent. Expected and observed mortality were similar. A thorough preoperative assessment of the patient risk profile is warranted in these technically complex subjects.

**Table:** 

Characteristics	Mean SD or N (%)
Male	100 (55%)
Age (years)	61.2 9.48
Caucasian/BAME	164/15
BMI (Kg/m2)	41.9 1.6
SCTS Logistic Euroscore (%)	1.6 4.17
Elective/Urgent/Emergency	118/56/5
Diabetes	100 (55)
Peripheral vascular disease	25 (13.9)
Atrial fibrillation	28 (15.6)
Renal impairment (moderate to severe)	32 (17.8)
LV ejection fraction < 30%	8 (4.4%)

### A70 Patient Recovery from Cardiac Surgery During the Covid-19 Pandemic: 1-year Outcomes from The CardiacCovid Study

#### Sanders, Julie^1^, Professor; Bueser, Teofila^1^, Ms; Beaumont, Emma^2^, Ms; Dodd, Matthew^2^, Mr; Owens, Gareth^3^, Mr; Murray, Sarah^4^, Mrs; Clayton, Tim^2^, Prof; Oo, Aung^1^, Prof

##### ^1^St Bartholomew's Hospital, London, UK; ^2^London School of Hygiene and Tropical Medicine, London, UK; ^3^Aortic Dissection Awareness UK and Ireland; ^4^SCTS Lay representative and NICOR Patient Engagement Lead

*Journal of Cardiothoracic Surgery* 2023, **18(Supp 1)**:A70

**Objectives**

The outbreak of Covid-19 was potentially stressful for everyone, and possibly heightened in those having cardiac surgery during the pandemic. We sought to explore the effect of the pandemic on recovery up to 1-year from cardiac surgery.

**Methods**

A prospective observational study (Ethics:20/YH/0132. Clinicaltrials.gov:NCT04366167) was established. Eligible patients were > 18 years old undergoing any form of cardiac surgery between 23rd March 2020 (UK lockdown) and 4th July 2020 (large lifting of lockdown). Those too unwell or unable to give consent/complete the questionnaires were excluded. Participants completed the EQ-5D (quality of life, QoL), impact of event (IES-R) (anxiety related to COVID-19), depression (CES-D) questionnaires at baseline (T0), 1 week after hospital discharge (T1), and 6 weeks (T2), 6 months (T3) and 1 year post-surgery (T4). Questionnaires were completed electronically (Amplitude system) or on paper and returned by post.

**Results**

196 patients participated. Questionnaire completion was 196(100%), 132(67.3%), 159(81.1%) and 149(76.0%) at T0-T4, respectively. Most participants were male (147(75.0%)), white British (156(79.6%)) with an average age 63.4 years (SD 11.2) and underwent urgent surgery (104(53.1%). No patients had COVID-19 and in-hospital mortality was 1(0.5%). Overall, anxiety due to the pandemic was high (T0-T3) and was greater in women and younger patients (T0-T4). Women also had lower QoL and higher depression, although overall rates of depression were within ranges observed in other studies in non-COVID-19 times.

**Conclusions**

The COVID-19 pandemic caused greater anxiety in patients undergoing cardiac surgery with women and younger participants particularly affected. Psychological support pre- and post-operatively in further crises or traumatic times, should be considered to aid recovery.

### A71 Identifying Predictors of Short- and Long-term Outcome After Surgery for Infective Endocarditis

#### Salmasi, M Yousuf^1^, Dr; Rizzo, Victoria^2^, Dr; Comanici, Maria^3^, Ms; Abdul Khader, Ashiq^1^, Dr; Athanasiou, Thanos^1^, Prof; Marczin, Nandor^3^, Dr; Raja, Shahzad^3^, Mr

##### ^1^Imperial College London, London, UK; ^2^St Thomas Hospital, London, UK; ^3^Royal Brompton and Harefield Trust, London, UK

*Journal of Cardiothoracic Surgery* 2023, **18(Supp 1)**:A71

**Background**

This study aimed to evaluate the clinical predictors of patient outcome after surgery for infective endocarditis.

**Methods**

We conducted a retrospective analysis of all consecutive patients undergoing surgery for infective endocarditis in the period January 2015 to February 2021. Multivariate analysis and survival analysis (including Cox-regression) were conducted to assess predictors of short- and long-term outcomes.

**Results**

In the study period, 148 patients had surgical management of endocarditis. Mean age was 60.1 ± 13.9 years, 31 were females (21%), 43 patients (29%) had had previous cardiac surgery, 34 patients (25%) had prosthetic valve endocarditis, 50 patients were reported as having subacute/chronic endocarditis (vs 99 patients acute). 15 patients had double valve endocarditis, 53 (44%) patients had at least a single positive blood culture prior to surgery, of these 18 were staphylococcus. Valve tissue provided a positive culture in 66% of patients.

Short-term outcomes were as follows: 67 patients (45%) suffered at least 1 post-operative complication. There were 13 in-hospital deaths (8.7%). 23 patients required post-operative haemofiltration. 27 patients suffered pulmonary complications post-surgery. Long-term survival (at 5 years) was 88.9%.

Haemofiltration post surgery was a significant predictor for worse survival outcome (filtration 45% vs no-filtration 96%, logrank test, p < 0.001). The existence of prosthetic valve endocarditis, blood culture positivity or redo surgery were not predictors of mortality (logistic regression, P > 0.05) or survival outcome (logrank, p > 0.05).

**Conclusions**

The study highlights the strong influence of renal dysfunction on short and long-term outcomes after cardiac surgery. This calls for a more in-depth analysis of vasoplegia markers to assess the influence of vasoplegia status and end-organ perfusion on short/long-term outcomes and potential methods for patient-specific management.

### A72 How Accurate is Cardiac Surgeons' Prediction of Operative Mortality Rate?

#### Oo, Shwe, Ms; Chan, Jeremy, Dr

##### University of Bristol, Bristol, UK

*Journal of Cardiothoracic Surgery* 2023, **18(Supp 1)**:A72

**Introduction**

Accurate outcome prediction is important during both counselling and consenting processes. The accuracy of cardiac surgeons predicting operative risk is not well known. We aimed to perform a literature review to compare surgeons’ and risk models’ prediction of operative mortality.

**Method**

A systematic review was performed in accordance with the updated 2020 PRIMSA guideline. Four major electronic data bases were reviewed. The outcome was defined as 30-day/in-hospital mortality. Comparison was made between surgeons’ pre-operative prediction, risk model scoring system and patients outcomes.

**Results**

4 studies with a total number of 6795 patients were included. Both surgeons and risk prediction models over-estimated the observed mortality rate. The mean mortality estimated by surgeons and the risk prediction models was 6.90% and 6.94%, respectively. This was higher than the observed mortality (4.74%).

**Conclusion**

Both surgeons and risk assessment models overestimated the mortality rate. When compared with the risk assessment model, surgeons tended to over-predict low and moderate risk and underestimated the mortality rate for high-risk patients.

### A73 How to Maintain a Cardiac Surgery Service Amidst a Global Pandemic?

#### Mouyer, Zakariya^1,2^,

##### ^1^ Imperial College London^2^, London, UK; ^2^ University of Manchester Medical School, Manchester, UK

*Journal of Cardiothoracic Surgery* 2023, **18(Supp 1)**:A73

**Objectives**

1. What is the SARS-CoV-2 virus?
2. How has the COVID-19 pandemic affected cardiac surgery globally?
3. How did a leading cardiac surgery centre in England cope and endure the pandemic?
4. What suggestions have been looked into for future planning and service protection?

**Introduction**

The COVID-19 outbreak emerged in December 2019, it has since had a devastating effect on all health services globally. One key leading cardiac centre in UK, that provides a world-class service to its 3.2 million patient database; was heavily affected by the pandemic.

**Methods**

In this report the cardiac surgery service was assessed by analysing data collected in the fashion of an audit from four different time points: Pre-COVID, England’s 1st Peak, England’s 2nd Peak and the Present Day. Parameters included: total surgeries conducted, cancellations, cancellations due to a shortage of cardiothoracic critical care unit (CTCCU) beds and weekly averages for the aforementioned.

**Results**

Results showed a significant drop in surgical output during the first peak (79.6% decreased output from Pre-COVID—see attached figure) and a large increase in cancellations both generally (from 25.1% to 28.2%) and due to CTCCU bed shortage (from 36.2% to 45.5%). However, a strong comeback was seen during the second peak (47.8% decreased output from Pre-COVID and 2.4% fewer cancellations), and an almost complete optimisation of service was observed in the Present day (45.1% increased output from Pre-COVID and 8.9% fewer cancellations).

**Conclusion**

This trust's cardiac surgery service has adapted immensely to its adverse circumstances; this audit has reported the list of measures taken to achieve this and the recommendations to achieve future surgical optimisation. The Trust managed to optimise its service and outperformed itself both from Pre-COVID and internationally. With further refining using PLECS and Canadian Society of Cardiac Surgeons guidance, full optimisation is feasible.
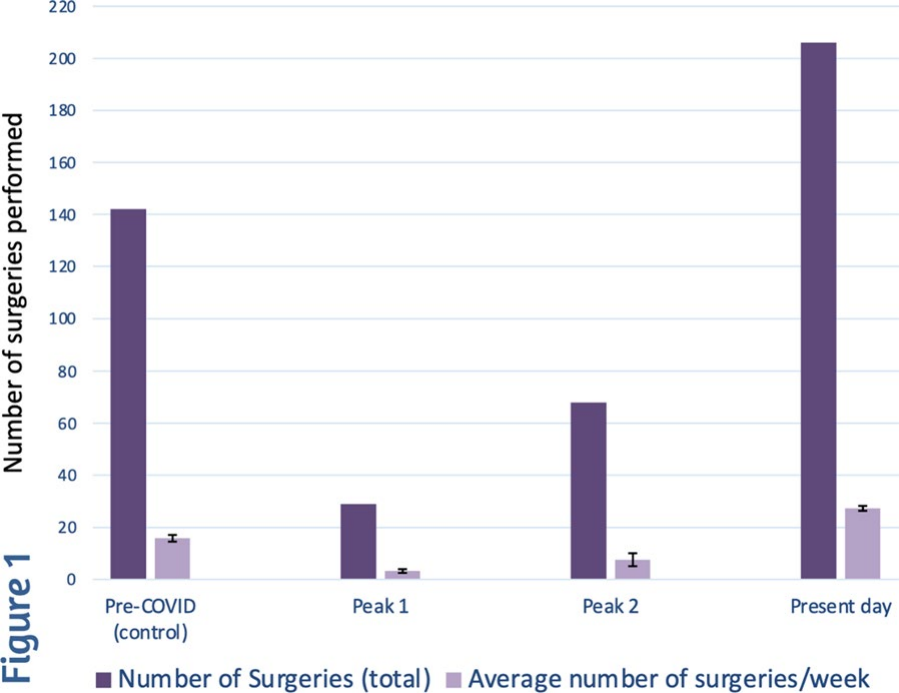


### A74 Current Trends in the Surgical Management of Infective Endocarditis: The UK-IE Survey

#### Bakr, Lubna, Miss; Raja, Shahzad, Mr

##### Harefield Hospital, Uxbridge, UK

*Journal of Cardiothoracic Surgery* 2023, **18(Supp 1)**:A74

**Objectives**

Surgical valve replacement is the cornerstone of infective endocarditis (IE) management in the presence of severe valvular destruction, uncontrolled infection, or large vegetation. The prosthetic valve choice, however, is not easy. Our aim is to explore the current trends in the surgical management of IE in the UK to improve our understanding of the current practice.

**Methods**

We developed the UK-IE survey and disseminated it to consultant cardiac surgeons around the UK through SCTS Weekly Updates.

**Results**

Biological valve was the most considered choice in the aortic position whether it was native or prosthetic valve IE. It was offered by 74% of responders to patients < 70 years and 98% to patients ≥ 70 years. Biological valves remained favourable in the mitral position in 81% of patients ≥ 70. However, mechanical ones were preferred by 76% of responders for patients < 70. Biological valves were offered to patients with double-valve IE and right-sided IE (87% and 77%, respectively). Valve repair was considered in 65% of right-sided IE and 45% of native mitral IE patients ≥ 70. Local antiseptics were considered by 58% of responders, mostly Betadine and/or topical antibiotics. Mechanical valves were preferred in young adults (18–40 years) while biological ones were preferred in women of childbearing age, injection drug users, dialysis patients and patients with liver cirrhosis. 86% of surgeons would not consider a monobloc aorto-mitral homograft or cardiac transplantation. 77% of surgeons are confident in their valve choice to prevent IE recurrence basing it on personal preference. Responses were received from across the UK (England 84%, 3.2% for each of Wales, Scotland and Northern Ireland) and the Republic of Ireland (7%) with 81% of centres having a specialist IE MDT.

**Conclusions**

Biological valves were preferred in all IE cases except young adults and mitral IE < 70 where mechanical valves were offered. 77% of choices were based on personal preference.

### A75 Right Thoracotomy Versus Conventional Median Sternotomy in Redo Cardiac Surgery: A 10-year Single-centre Experience

#### Ng Yin Ling, Clarissa^1^, Dr; Chacko, Jacob^2^, Mr; Bleetman, David^2^, Mr; Leung, Kristie^3^, Ms; Khan, Habib^2^, Mr; Whitaker, Donald^2^, Mr; Deshpande, Ranjit^2^, Mr; Wendler, Olaf^2^, Prof; Baghai, Max^2^, Mr;

##### ^1^Department of Surgery and Cancer, Imperial College London, London, UK; ^2^Department of Cardiothoracic Surgery, King's College Hospital, London, UK; ^3^University College London, London, UK; ^4^Faculty of Medicine, University College London, London, UK

*Journal of Cardiothoracic Surgery* 2023, **18(Supp 1)**:A75

**Introduction**

Minimally invasive cardiac surgery (MICS) has been gaining popularity over conventional sternotomy due to reduced invasiveness. However, in redo cardiac surgery when the risks are higher due to adhesions, few studies compare long-term outcomes.

**Objective**

We aim to compare the perioperative and long-term mortality and stroke/TIA rate in patients undergoing median sternotomy and right thoracotomy for redo cardiac surgery. Secondary outcomes were post-operative and total hospital stay, blood products used, return to theatre and new renal replacement therapy (RRT).

**Methods**

We retrospectively analysed a cohort of 127 sternotomy patients and 29 right thoracotomy patients for redo cardiac surgery between 2010–2020. All patients underwent valve repair or replacement. Patients requiring concomitant cardiac procedures were included.

**Results**

In-hospital and long-term follow-up were 100% and 98.1% complete and a maximum of 10.4 years. There were 52 deaths (33.3%) within the follow-up period. Perioperative mortality was 16.5% in sternotomy versus 3.45% in MICS, although this difference was insignificant after adjustment for logistic EuroSCORE (HR 0.38, [0.05–2.79]). In multivariate analysis, long-term mortality was significantly lower in MICS (HR 0.30 [0.10–0.85]). Adjusted Kaplan–Meier curves revealed lower long-term mortality in the MICS group (p < 0.0001). Differences in stroke/TIA rates were insignificant (odds ratio 3.23 [0.85–12.8]). There were significantly less RBC (p = 0.0001) and total blood products (p = 0.0032) used in the MICS group. All 12 patients who returned to theatre for bleeding complications were in the sternotomy group. Although mean post-operative and total hospital stay and new RRT were lower in the MICS group, these differences were insignificant.

**Conclusion**

MICS for redo cardiac surgery is a safe and effective alternative to median sternotomy without compromising both short-term and long-term clinical outcomes.

### A76 The Implementation of a Virtual Learning Environment in Facilitating Cardiothoracic Surgical Teaching – The West Midlands Experience

#### Iqbal, Yassir^1^, Mr; Iqbal, Akshay^1^, Mr; Bleibleh, Sabri^2^, Mr; Ghosh, Shilajit^3^, Mr; Graham, Timothy^4^, Mr; Ahmed, Usman^4^, Mr

##### ^1^Queen Elizabeth Hospital, Birmingham, Birmingham, UK; ^2^Department of Orthopaedic Surgery, Royal Orthopaedic Hospital NHS Trust, Birmingham, UK; ^3^Department of Thoracic Surgery, UHNM NHS Trust, Stoke-on-Trent, UK; ^4^School of Surgery, Health Education England, West Midlands, Birmingham, UK

*Journal of Cardiothoracic Surgery* 2023, **18(Supp 1)**:A76

**Objectives**

The COVID-19 pandemic has resulted in widespread disruption, and overwhelmed healthcare services globally. In addition to this, we have seen an immediate and dramatic effect on training for surgical trainees. Ultimately, these challenges have provided a "teachable" moment for trainers and trainees to advantage of, and has come in the form of innovative solutions to optimise educational endeavours utilising twenty-first century technology.

**Methods**

Prior to the pandemic, teaching was delivered to 17 higher surgical trainees through a programme of monthly lectures and face-to-face workshops, dissection sessions and cadaveric skills stations at a clinical skills lab placed within one of the regional trusts. The Postgraduate Virtual Learning Environment (PGVLE) is an online educational platform using Moodle and BigBlueButton (BBB) web-conferencing software. It has been utilised to deliver the teaching programme during the COVID lockdown.

**Results**

The monthly full day teaching programme has now been formally replaced by a virtual weekly programme covering topics pertinent to the curriculum. The significant advantage of this new system is that sessions are largely during the evening allowing for maximal attendance as trainees don’t have the added burden of organising study leave and moreover the preparatory reading has enabled for maximal gain from each session. We have adopted a "trainee-directed" approach in that all trainees are individually responsible for organising teaching sessions on a rolling rotational basis. We envisage that with time, we will be able to replicate cadaveric and dissection courses virtually with opportunity for face-to-face wetlabs to consolidate learning.

**Conclusions**

Implementing a deanery wide specialty teaching programme has given us the opportunity to develop a standardised set of processes that allow for a consistent level of education based on the cardiothoracic curriculum.

### A77 Left Video-Assisted Thoracoscopic Ablation for Persistent Ventricular Tachycardia, a Case Report

#### Moawad, Nader^1^, Mr; Harfield, Jack^1^, Mr; Rogers, Luke^1^, Mr; Podd, Steven^2^, Dr; Dalrymple-Hay, Malcolm^1^, Mr

##### ^1^Derriford Hospital, Plymouth, UK; ^2^Torbay Hospital, Torquay, UK

*Journal of Cardiothoracic Surgery* 2023, **18(Supp 1)**:A77

https://www.youtube.com/watch?v=fSYMarw8jTY

### A78 Techniques for Pulmonary Artery Reconstruction for Pulmonary Artery Aneurysm: Case Report and Review of the Literature

#### Holmes, Charlotte^1^, Miss; Freystaetter, Kathrin, Miss; Goodwin, Andrew, Mr

##### ^1^The James Cook University Hospital, Middlesbrough, UK

*Journal of Cardiothoracic Surgery* 2023, **18(Supp 1)**:A78

**Objectives**

Pulmonary artery aneurysm (PAA) is rare. Natural history of PAA is poorly understood and there are no treatment guidelines. Surgical repair is done due to risk of rupture and dissection. Due to the infrequency of these cases there is no established surgical technique. We aim to present a case study of a pulmonary artery reconstruction for a PAA and a review of the literature.

**Methods**

A case study presentation and a PubMed search using MESH terms "surgical treatment" or "reconstruction" and "pulmonary artery aneurysm" was conducted.

**Results**

A 46-year-old female presented with an 63 × 56x85 mm incidental pulmonary artery aneurysm. Pulmonary artery pressure 30mmHG. The repair was performed on cardiopulmonary bypass without cardiac arrest. The pulmonary trunk was excised 1 cm above the valve. The right and left pulmonary arteries were resected. A 22 mm Hemashield graft was anastomosed the left pulmonary artery and right pulmonary artery. An oval slit was made and a size 26 mm Hemashield graft was anastomosed end to side, forming a ‘T’ junction and then anastomosed to the remainder of the pulmonary trunk.

Surgical repair is recommended for aneurysms > 6 cm or increasing in size > 0.5 cm/0.5yrs, in symptomatic patients or if compression of adjacent structures. Pulmonary artery hypertension (PAH) increases risk of rupture and is also an indication for surgery. A variety of surgical techniques have been described including: aneurysmorrhaphy, pericardial patch reconstruction, and interposition grafts with synthetic or allografts. Replacement with a synthetic interposition graft is most common. Our case is the first to describe a "T junction" technique.

**Conclusions**

PAA are rare with no guidelines on management. Surgical repair is considered due to size, symptoms or those with PAH. Due to the paucity of cases surgical techniques vary and depend on operative findings. Our case had good outcomes and is the first describe the interposition "T junction" graft technique used.

Informed consent to publish had been obtained.

### A79 Are There Differences in Mortality and Morbidity Outcomes for Cardiothoracic Surgical Procedures Performed by Trainee Versus Attending?

#### Comanici, Maria, Dr; Salmasi, M Yousuf, Dr; Raja, Shahzad G., Mr; Attia, Rizwan Q., Dr

##### Harefield Hospital, London, UK

*Journal of Cardiothoracic Surgery* 2023, **18(Supp 1)**:A79

**Objectives**

There are increasing pressures to decrease training time in the operating room. We sought to assess the safety of training in cardiothoracic surgery comparing cases performed by trainee’s vs consultants.

**Methods**

EmBase, Scopus, PubMed and OVID MEDLINE were assessed in August 2021 independently by two authors while a third author arbitrated decisions to resolve disagreements. Inclusion criteria were articles on cardiothoracic surgery, training, and outcomes. Studies were assessed for appropriateness as per CBEM criteria. 892 results were obtained, 51 meet the specified criteria. 27 represented best evidence (2-Meta-analyses, 1-RCT and 24 retrospective cohort studies).

**Results**

474,160 operative outcomes were assessed including 434,535 CABG (431,329 on-pump vs 3206 off-pump), 3090 AVR, 1740 MVR/repair, 26,433 mixed, 3565 congenital and 4797 thoracic. 398,058 cases were performed by trainees and 75,943 by consultants. 159 cases were indeterminate. There were no statistically significant differences in the patients’ pre-operative risk scores. All studies excluded extreme high-risk patients in emergency setting, those with poor left ventricular function and re-operation cases that were operated on by consultants. There were no differences in CPB and clamp times for CABG, times for valve replacement and repair cases were longer for trainees. There were no differences in the post-operative outcomes including peri-operative myocardial infarction, resternotomy for bleeding, stroke, renal failure, ITU and total length of stay. One study reported no differences on angiographic graft patency at 1-year. There were no differences in in-hospital or mid-term mortality out to five-years.

**Discussion**

Published data indicate that trainees can perform cardiothoracic surgical procedures in dedicated high-volume units with outcomes comparable to those of trainers. Academic programs should focus on strategies to maximise trainees’ exposure as primary operating surgeons.

### A80 Left Ventricular Aneurysm Repair: Long-Term Results of a Beating Heart Autologous Endoventriculoplasty Compared to Current Common Practice

#### Halfwerk, Frank, Dr; Jansen, Martin, Mr; Plonek, Tomasz, Dr; Grandjean, Jan, Prof

##### Thoraxcentrum Twente, Medisch Spectrum Twente, Enschede, The Netherlands

*Journal of Cardiothoracic Surgery* 2023, **18(Supp 1)**:A80

**Objectives**

Left ventricular aneurysm (LVA) formation is a severe complication after transmural myocardial infarction. Over the last 60 years, different surgical ventricular reconstruction (SVR) techniques have been developed, but there still is no clear consensus about the most appropriate technique. This study analysed short and long-term outcomes of a new SVR technique by using autologous endocardium compared to other more commonly used techniques (e.g. plication, patch).

**Methods**

We retrospectively reviewed 95 patients receiving SVR due to a LVA between 2005 and 2019 in our centre. Patients underwent either on-pump beating heart endoventriculoplasty using autologous endocardium as described by Grandjean (see Figure) or a more commonly used technique (e.g. patch, plication). Early surgical outcomes, long-term survival and postoperative cardiac function were compared.

**Results**

Mean age of patients was 67 [61–74] years. Pre-operative left ventricular ejection fraction (LVEF) was 33% [22–40%]. 52 (55%) patients had NYHA III/IV classification. No significant difference in baseline characteristics, in-hospital mortality or in-hospital complications were found. 1, 3 and 5-year postoperative LVEF in the autologous group were 40%, 43% and 36% compared to 45%, 44% and 39% in the control group (p = 0.21). Survival rates at 1, 5 and 10 year were 93%, 62% and 24%, and did not differ between groups.

**Conclusions**

The new SVR technique, using autologous endocardium was noninferior compared to longer existing SVR techniques. Beating-heart SVR is feasible and can be used in patients with poor left ventricular function where aortic cross-clamp and subsequent ischemia might lead to problems with weaning from cardiopulmonary bypass.
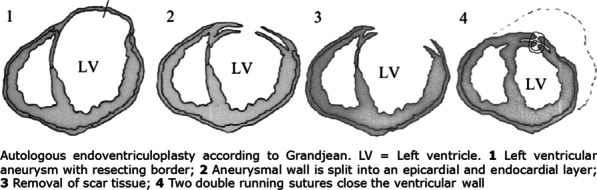


### A81 What Impact Does Enhanced Recovery After Surgery (ERAS) Have on Caregivers of Lung Cancer Patients and How is This Reflected in Their Experience?

#### Johns, Joelle, Miss; Longbone, Tyler, Mr; Boele, Florien, Dr; Pompili, Cecilia, Dr

##### University of Leeds, Leeds, UK

*Journal of Cardiothoracic Surgery* 2023, **18(Supp 1)**:A81

**Introduction and Objectives**

Enhanced recovery after surgery (ERAS) is a multimodal care pathway which focuses on patient-centered interventions and reduces both post-operative complications and patient morbidity. Research has highlighted the profound implications of a cancer diagnosis and treatment on caregiver quality of life (QoL). Therefore, our objective is to explore the impact of ERAS implementation on caregiver experiences after lung cancer surgery.

**Methods**

A qualitative prospective study was done to explore caregiver experiences after lung cancer surgery in a single institution. Remote semi-structured interviews (SSI) were conducted, transcribed and thematically analysed.

**Results**

Eight caregivers aged 43–84 participated. Thematic analysis yielded 5 main themes: (1) Impact of caregiving experiences on QoL; (2) Emotional distress; (3) Impact of COVID-19 on caregiver experiences; (4) Support; (5) Communication with the healthcare team. Caregivers highlighted detrimental impacts on caregiver QoL and emotional distress. Caregivers were generally satisfied with pre-operative care but not post-operative care. A proportion of caregivers felt support provided could be improved through better communication from staff, better management of pain and more caregiver education.

**Conclusions**

COVID-19 has affected the care provided with more remote consultations. Better support for caregivers could improve caregiver and patient outcomes. Further research is required to improve patient and caregiver experience.

### A82 Factors Predictive of Adverse Outcomes in Patients Undergoing Surgery for Infective Endocarditis

#### Varghese, David^1^, Mr; Gradinariu, George^1^, Mr; Awad, Wael^2^, Mr; Sheikh, Amir^2^, Mr; Mohite, Prashant^1^, Mr; Sadia Aftab, Sadia^1^, Mrs; Oyebanji, Tunde^3^, Mr; Phillips, Derek^1^, Dr; Doshi, Hari^1^, Mr; Morcos, Karim^1^, Mr; Curry, Philip^1^, Mr

##### ^1^Golden Jubilee National Hospital, Glasgow, UK; ^2^St Barts Hospital, London, UK; ^3^Royal Victoria Hospital, Belfast, UK

*Journal of Cardiothoracic Surgery* 2023, **18(Supp 1)**:A82

**Background**

Infective endocarditis (IE) is associated with high morbidity and mortality. Surgery can be challenging but highly effective. The aim of this study was to identify preoperative risk factors associated with adverse outcomes after surgery.

**Methods**

We conducted a retrospective analysis of all patients undergoing surgery for IE between 2012 to 2020 at our unit. We collected patient demographics, laboratory and imaging results, operative details and post-operative outcomes. The primary outcome was in-hospital mortality. Data was analysed using SPSS and Unistat statistical software. Binary logistic regression was used to identify factors associated with the primary outcome. Results are expressed as median values (Q1 to Q3), percentages and total number of cases for categorical variables.

**Results**

158 patients underwent surgery for IE, median age was 59 years (48 to 68), Logistic EuroSCORE was 10.3% (6.0 to 22.7), 115 (73%) were male and 141 (89%) were non-emergency operations. There were 65 isolated aortic valve procedures, 61 isolated mitral procedures, 7 isolated tricuspid, 23 combined double valve procedures and 2 triple valve procedures. There were 142 valve replacements (88 tissue, 54 mechanical), 30 repairs and 3 other procedures performed either alone or in combination. The in-hospital mortality was 5.7% (9/158 patients). Univariate logistic regression identified WCC (OR 1.140 [1.020–1.274], p = 0.021), albumin (OR 0.832 [0.737 – 0.939], p = 0.001) and total protein (OR 0.899 [0.830–0.974], p = 0.009) as factors associated with mortality.

**Conclusion**

High WCC, low albumin and low total protein levels are predictors of adverse outcomes after surgery for infective endocarditis. Many factors are involved in timing of surgery. These findings underline the importance of preoperative medical optimization of the nutritional status as well as maximising the attempt to control the infective process with targeted antibiotic therapy within the window of time available.

### A82 A UK First Use of Remote Video Assisted Surgical Training for Cardiac Surgery. An NTN Experience

#### Karsan, Rick, Mr; Beattie, Gwyn, Mr

##### Royal Victoria Hospital, Belfast, UK

*Journal of Cardiothoracic Surgery* 2023, **18(Supp 1)**:A82


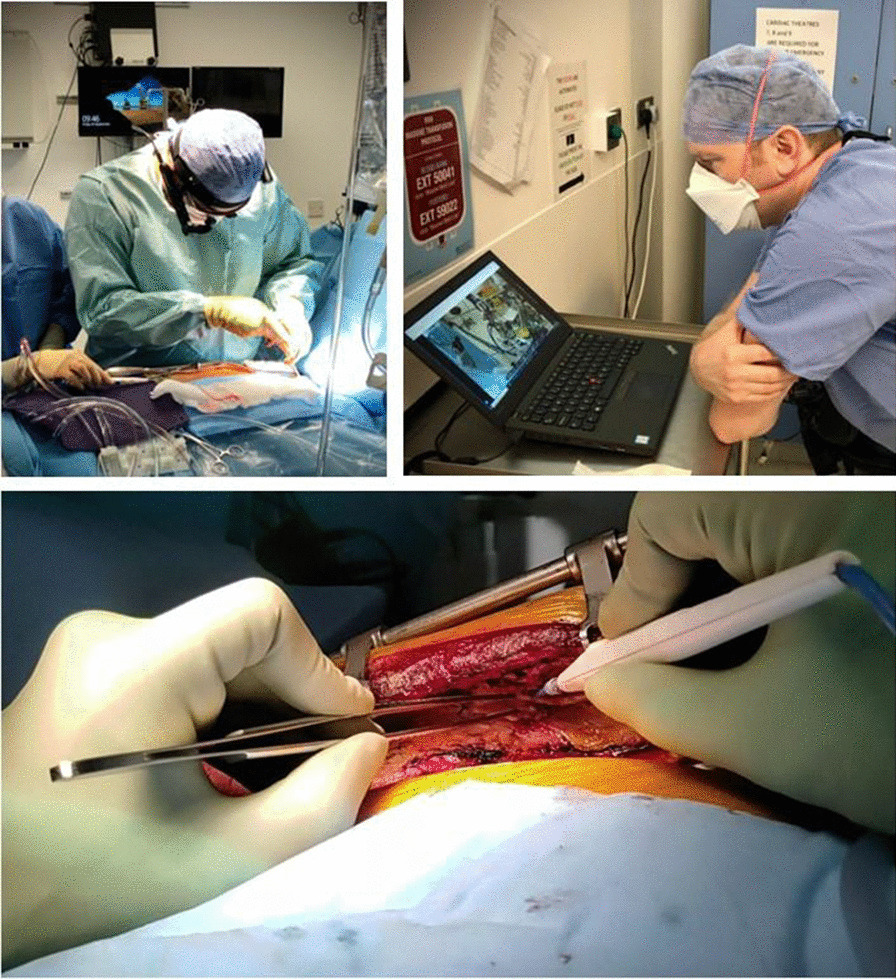


With Cardiothoracic Surgery within the UK becoming more condensed with implementation of a 7-year curriculum, the importance of hands-on training has never been more pertinent to develop surgical skills and increase logbook numbers to attain CCT. The use of simulation and models; as well as reviewing digital media continues to have a greater and more expansive role in training however, there is always the limitation these cannot replicate the experiences encountered in patients.

We present our experience of the first UK use of live-streamed video assisted training, with a specialised camera equipped headset worn by the trainee to allow trainers to visualise a trainee’s actions live and supply instruction whilst away from theatre. This was particularly helpful in difficult to observe areas such as internal mammary artery harvesting. As with all technological advancement and the dynamic nature of training in Cardiac Surgery, there are both pros and cons to this tool.

Through our experiences, we have found trainees have built up their logbooks to include significantly more trainer unscrubbed. Ultimately trainees have found they developed skills and confidence faster.

We have utilised this new tool to train trainees allowing them to build their skills and logbook. We provide a detailed account of our experiences with live-streamed training and the potential future role for such tools in Cardiothoracic Surgery training.

### A83 The Effects of Dietary Iron Deficiency on Mitochondrial Function and Iron Metabolism in Murine Model

#### Tomkova, Kristina^1^, Miss; Cabolis, Katerina^2^, Miss; Wozniak, Marcin^1^, Dr; Sajic, Marija^2^, Dr; Murphy, Gavin^1^, Prof

##### ^1^University of Leicester, Leicester, UK; ^2^University College London, London, UK

*Journal of Cardiothoracic Surgery* 2023, **18(Supp 1)**:A83

The relationships between global iron deficiency, cellular iron metabolism, and mitochondrial function are poorly defined. Therefore, we proposed to explore the link between these processes in a murine model with iron deficiency induced through an iron-restricted diet. A total number of 88 mice were divided into three groups: 1) control group (n = 39), 2) iron deficient (ID) group (n = 21), and 3) iron repleted (IR) group (n = 28).

The results showed that dietary iron deficiency results in decreased iron levels in heart tissue and serum; however, the iron content of the mitochondrial fractions was not altered. Iron concentration in heart tissue but not in serum was corrected by iron repletion. Tissue ferritin and haemoglobin levels were decreased in both ID and IR groups. The IRP1 expression, IRP1 activity, mtDNA copy number and mtDNA damage were not affected by either iron deficiency or iron repletion. The enzymatic activities of mitochondrial complex II and III were decreased in ID mice, but were returned to normal levels in the IR group. The activities of complex I and IV as well as the protein expression of all four complexes was not affected by iron deficiency.

Based on these results, we conclude that dietary iron deficiency deregulates global iron metabolism as evidenced in decreased iron, haemoglobin and ferritin levels. However, this dysregulation is not sufficient to trigger the expected increase in IRP1 mRNA binding. This dysregulation in iron availability also led to disruption of mitochondrial function. As overall mitochondrial health was not affected, we hypothesise that this disruption affects the mitochondrial respiratory chain directly on protein level, resulting in inefficient energy production. We believe this link between iron metabolism disruption and mitochondrial dysfunction provides insights into potential molecular mechanisms of physical symptom of iron deficiency and anaemia.
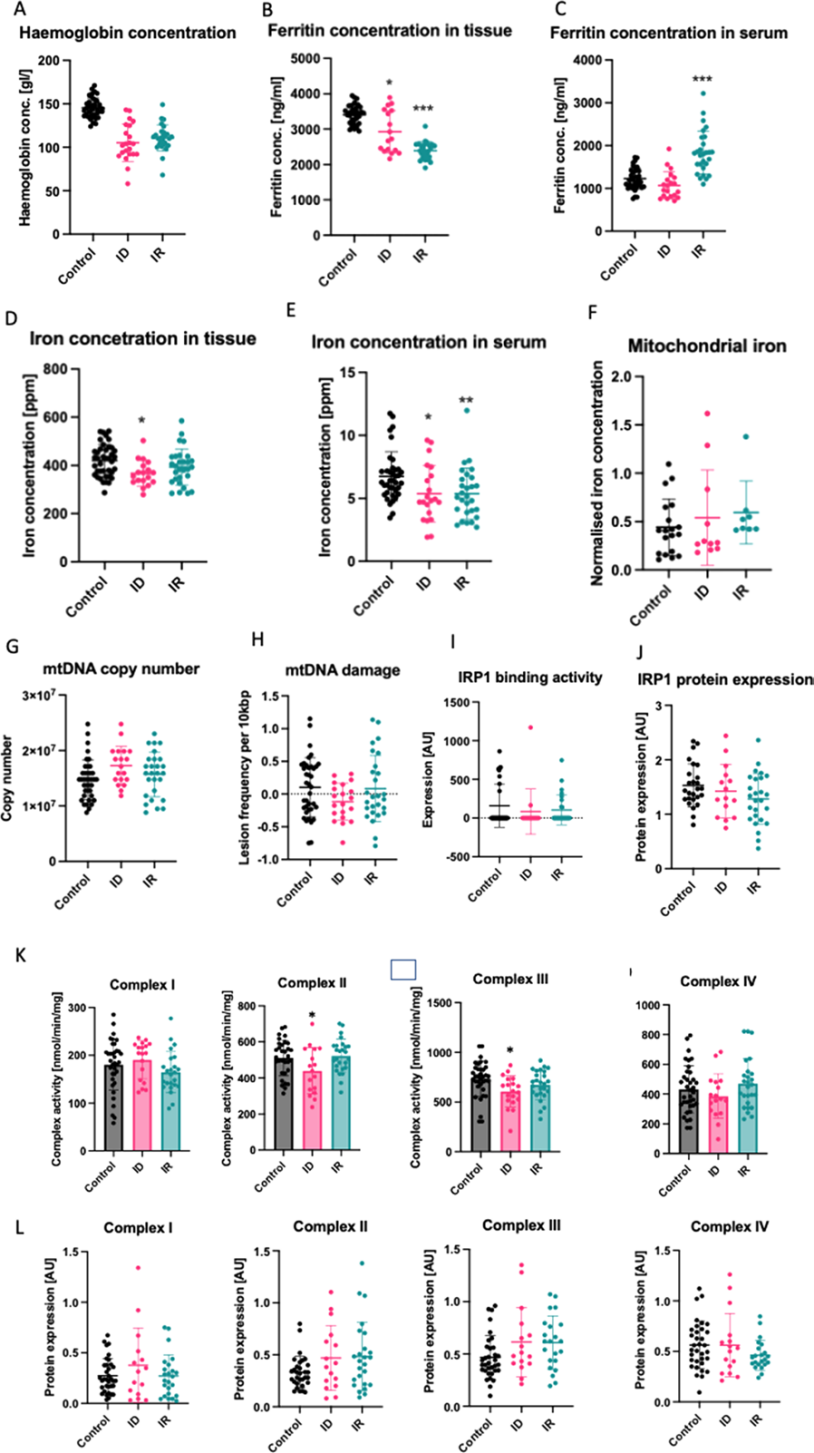


### A84 Rigid Sternal Fixation to Prevent Sternal Instability & Dehiscence in High-risk Patients After Cardiac Surgery: Early Experience

#### Alam, Ruhina, Miss; Holland, Luke, Mr; Modi, Amit, Mr; Hyde, Jonathan, Mr

##### University Hospital Sussex, Brigton, UK

*Journal of Cardiothoracic Surgery* 2023, **18(Supp 1)**:A84

**Background**

Median sternotomy is the standard approach for most cardiac surgical procedures. Wire cerclage remains the primary median sternotomy closure technique where side-to-side movement is the only process addressed with no focus on stabilisation (fixation) to prevent cranio-caudal micromovement. In a multicentre randomized trial, rigid plate sternal fixation (RPF) compared with wire cerclage resulted in improved sternal healing and reduced sternal complications, as well as significantly less pain. Introduction of a structured and clinically based patient-adjusted risk score to predict the likelihood of sternal instability or dehiscence can help the surgeon in their choice of sternal closure technique and inform the use of rigid plate fixation in patients with a high risk- score.

**Predictive Risk-score**

We developed a literature-based predictive risk-score system (Table 1). A score of 8 or more suggests a significantly higher risk of sternal dehiscence and indicates supplementary fixation.
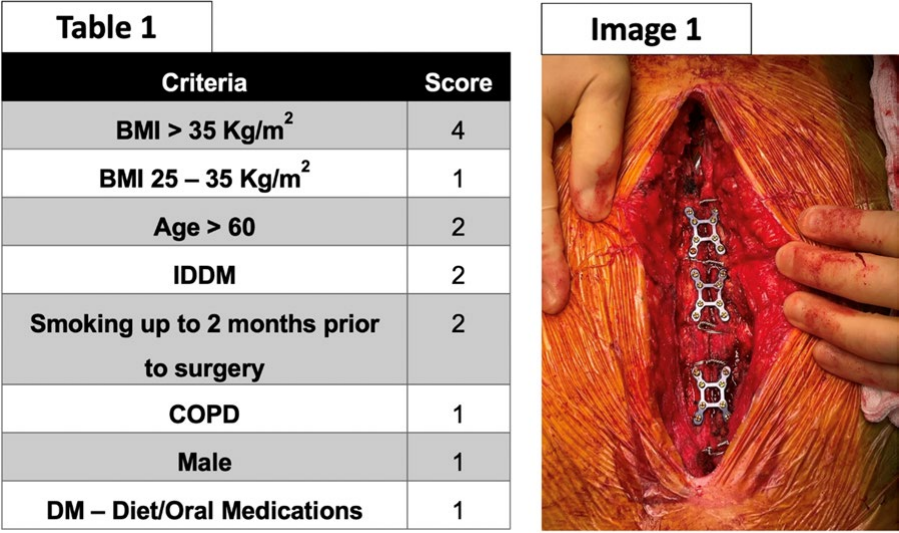


**Early Experience**

These patients are, by definition, a very high-risk demographic for sternal problems. 3 patients with a score of 8 or above were eligible and underwent RPF with SternaLock Blu RPF system (Image 1). They were assessed by daily pain score (Day 1/2/3/4/7 and at 6 weeks),use of morphine PCA and ease of return to daily activity. All 3 had a pain score of 4 or less after 24 h, 2 didn’t require PCA and 0 required oral opiates on discharge. They all only required paracetamol after 2 weeks. All 3 mobilised easily on D1 and were fit for discharge on D4. By 6 weeks, all 3 had returned to their pre-operative activity level.

**Conclusion**

Our limited early experience is very promising, and we feel RPF should be incorporated into the ERAS pathway to aid early recovery. The technique is easily learnt & reproducible, and despite adding a small relative cost, the avoidance of long-term stays associated with sternal dehiscence makes it very financially effective.

### A85 Circulating Cell-Free Mitochondrial DNA and Cytokines as Predictors of Cardiovascular Susceptibly for Atrial Fibrillation after Cardiac Surgery

#### Naase, Hatam^1^, Mr; Caruso, Vincenzo^2^, Mr; Evans, Paul^3^, Prof; Athanasiou, Thanos^1^, Prof

##### ^1^Imperial College London, London, UK, St Mary's Hospital, London, UK; ^2^Guy's and St Thomas' NHS Foundation Trust, London, UK; ^3^University of Sheffield, Sheffield, UK

*Journal of Cardiothoracic Surgery* 2023, **18(Supp 1)**:A85

**Objective**

To identify any relationship between postoperative atrial fibrillation (POAF) and level of specific inflammatory markers, such as circulating cell-free mitochondrial deoxyribonucleic acid (ccf-mtDNA), circulating cytokines or transcription factors.

**Methods**

This prospective cohort study was constituted by 88 patients (mean age 67 ± 7 years) who underwent coronary artery by-pass graft (CABG) or aortic valve replacement (AVR). The levels of inflammatory markers were measured preoperatively in all the patients.

**Results**

The prevalence of POAF after AVR, on-pump and off-pump CABG was 50%, 27.3% and 27.2%, respectively. All the biomarkers tested were significantly elevated preoperatively in patients who later developed POAF; at multivariate analysis, ccf-mtDNA and immune interferon-alpha(IFN-α) were the only independent predictors for the development of POAF.

The preoperative levels of ccf-mtDNA were higher in patients with ischemic heart disease than in those with no ischemic coronary disease. However, the overall incidence of developing POAF was higher in AVR than CABG (AVR:50%; on-pump CABG: 28%; off-pump CABG:27%).

**Conclusions**

An increase in the circulating levels of either ccf-mtDNA or IFN-α may predict POAF development, preoperatively. Prophylactic therapy with anti-inflammatory may be beneficial to reduce the incidence of POAF in patients with a high preoperative level of ccf-mtDNA.
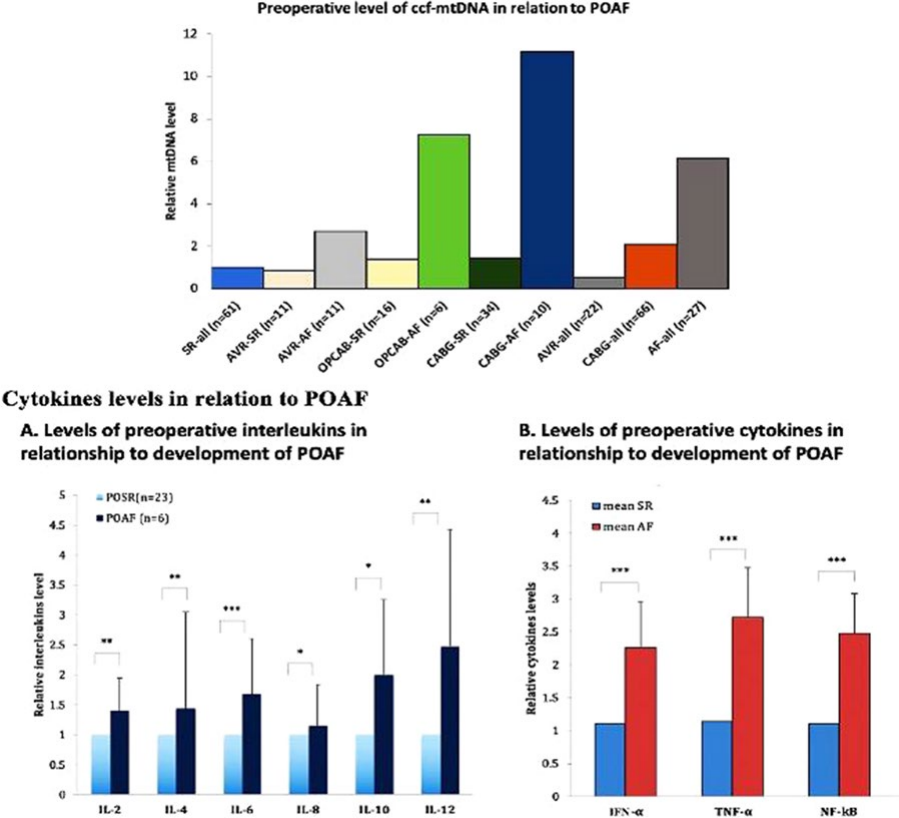


### A86 Cultural Competence is Essential for a Good Patient Experience; how do we get There?

#### Layton, Georgia R., Miss; Marsico, Roberto, Mr; Low, Mei Ken, Dr; Abbasciano, Riccardo, Mr; El-Dean, Zein, Mr; Bingley, Patricia, Ms; Zakkar, Mustafa, Mr

##### Department of Cardiac Surgery, Glenfield Hospital, University Hospitals of Leicester NHS Trust, Leicester, UK

*Journal of Cardiothoracic Surgery* 2023, **18(Supp 1)**:A86

**Introduction**

Lack of culturally competent (equitable, accessible and non-discriminatory) care may contribute to patient dissatisfaction with treatment and hinder compliance to therapy and outcomes.

We set out to assess patient satisfaction with doctor-patient interaction in the out-patient cardiac surgery clinic of an ethnically and socio-economically diverse city (Leicester) and whether mismatch of doctor-patient culture contributed to the patient experience.

**Methods**

All new patients attending in-person cardiac surgery out-patient clinic between May and August 2021 were provided with a two-stage questionnaire to complete anonymously; the first prior to consultation, the second after.

**Results**

116 patients completed the questionnaire (65% response rate). Patient demographics are listed in Table 1. 6% (n = 7) of respondents did not provide data on gender, employment status, ethnicity or preferred language. 8.6% (n = 10) did not provide data of their qualifications.

**Table:** 

Category	Total number, n = 116	% of total respondees
Male: Female	76: 33	65.5: 28.4
Eployed	31	26.7
Unemployed	15	12.9
Retired	63	54.3
Qualifications	65	56.0
No qualifications	41	35.5
Preferred language English: Non-English language	106: 3	91.4: 2.6
Ethnicity: White British	95	81.9
Ethnicity: other than White British	14	12.1

Patients reported a mean satisfaction with their doctors’ communication skills of 4.7 (range 3–5) and a mean overall satisfaction of 4.8 (range 3–5), on a scale where 1 was very poor and 5 was excellent. Respondents were more than 80% Caucasian. Ethnicity of participants did not match the ethnic diversity of the region.

**Conclusions**

Patients of non-white British ethnicity are either not accessing or not being identified for referral to our cardiac surgery unit. Although a good level of patient satisfaction was reported, the demographics of the participants demonstrate potential barriers preventing the provision of culturally competent care. Further work is needed to identify and address the barriers in healthcare environments which are disproportionately impacting patients from non-white ethnicities.

### A87 The Perceived Influence of COVID-19 on Core Surgical Training in the UK

#### Panahi, Pedram^1^, Mr; Seraj, Shaikh Sanjid^2^, Dr; Veeralakshmanan, Pushpa^3^, Miss; Unsworth-White, Jonathan^1^, Mr

##### ^1^University Hospitals Plymouth NHS Trust, Plymouth, UK; ^2^Walsall Healthcare NHS Trust, Walsall, UK; ^3^University Hospitals Birmingham NHS Trust, Birmingham, UK

*Journal of Cardiothoracic Surgery* 2023, **18(Supp 1)**:A87

**Objectives**

Surgical training has been affected by COVID-19 from the early stages of the pandemic. Here, we aim to carry out a detailed investigation of its perceived impact on core surgical trainees and their surgical career progression in the UK.

**Methods**

An online survey was devised using Google Forms which was distributed to core surgical trainees across the UK.

**Results**

75 trainees responded, 35 were in the first year and 40 in the second year of their surgical training programme. There was a median number of 10 days (Interquartile range 0–30) of redeployment and 2 days (Interquartile range 0–14) of sick leave due to confirmed / suspected COVID-19. A drop was observed in respondents’ global perception of their portfolio quality and 42 respondents (56%) felt that operative experience was the most impacted portfolio domain. The least impacted domains according to a calculated summary statistic were the ability to deliver teaching and work on leadership / management qualities. Eight respondents (11%) achieved an Annual Review of Competence Progression outcome 10.1 and 3 (4%) achieved an outcome 10.2. 63 respondents (84%) felt more stressed as a result of the pandemic and 44 respondents (59%) indicated that they have lost confidence as a surgeon due to the pandemic.

**Conclusion**

Amongst the respondents, a marked negative impact was observed in several domains affecting both surgical training and career progression. Allocated theatre time was the most adversely affected domain. These factors need to be addressed in surgical training schemes as the pandemic passes.

### A88 A Review of Radical Nephrectomy with Caval Thrombectomy for Renal Cell Carcinoma Over Ten Years

#### Panahi, Pedram, Mr; Enemosah, Ibrahim, Mr; Yao, Lucy, Dr; Aroori, Somaiah, Mr; McInerney, Paul, Mr

##### University Hospitals Plymouth NHS Trust, Plymouth, UK

*Journal of Cardiothoracic Surgery* 2023, **18(Supp 1)**:A88

**Objectives**

Renal cell carcinoma (RCC) with inferior vena cava (IVC) extension demands surgical excision to achieve a curative outcome. This retrospective study reviewed the immediate and long-term outcomes of radical nephrectomy and IVC thrombectomy in a tertiary centre.

**Methods**

Nine patients underwent radical nephrectomy with IVC thrombectomy from 2009 to 2019 at University Hospitals Plymouth NHS Trust. Five of these patients had IVC thrombus extending to the intrahepatic IVC; of these, one extended further into the right atrium. One patient required cardiopulmonary bypass (CPB).

**Results**

The mean operation duration was 224 min (range 155–498). One death was observed during index admission at 18 days post-operatively; this was the CPB patient who developed significant post-operative complications including haemorrhage and mesenteric ischaemia. For other patients, the survival range was 16 months to 8 years (75% two-year survival, 12.5% five-year survival). Worse outcomes were observed in patients with pre-operative metastatic disease beyond the IVC: one case affecting aortocaval lymph nodes and two others with pulmonary involvement. Despite adjuvant chemoradiotherapy, three (75%) had cancer recurrence within eight months.

**Conclusions**

Radical nephrectomy and caval thrombectomy without CPB is a safe procedure. The amount of tumour burden does not equate to a lower long-term survival.

### A89 Validity and Reliability of Radial Artery Assessment Techniques in Coronary Artery Bypass Grafting—A Systematic Review

#### De Franco, Vincenzo, Mr

##### Royal Papworth Hospital NHS Foundation Trust, Cambridge, UK

*Journal of Cardiothoracic Surgery* 2023, **18(Supp 1)**:A89

**Objectives**

Accuracy and consistency of screening tests implemented for the pre-operative assessment of radial artery (RA) graft, prior to surgical coronary revascularisation, has been debated for many years. Correct RA assessment is crucial prior to their surgical harvesting to avoid post-operative complications. A systematic review was conducted with the aim of evaluating and comparing the validity and reliability of the most commonly adopted RA assessment techniques.

Is ultrasonography more accurate and reliable than the modified Allen test, pulse-oximetry and plethysmography, in RA assessment for patients undergoing coronary revascularisation?

**Methods**

A systematic search was undertaken, appraising relevant primary research studies published between 2010 and 2020. MEDLINE, PubMed, CINHAL, Scopus and EMBASE databases were consulted, to access studies relating to the assessment of RAs during coronary artery bypass grafting. Included articles were reviewed and selection criteria applied, data findings were extracted for analysis, narrative synthesis and conclusions drawn. Critical appraisal of the included studies was performed using the modified Downs and Black checklist.

**Results**

Nine studies addressing the research question were included in the review. Seven studies identified the reduced validity and/or reliability of the MAT, four of which highlighted the poor sensitivity, poor specificity and the subjectivity of the screening test. Two studies established that pulse-oximetry and plethysmography, used in combination with the MAT, offer more objective results than an isolated MAT, also impacting on sensitivity and specificity. Ultrasonography provides important insight into the morphological characteristics of RAs, providing an accurate and reliable anatomical RA assessment.

**Conclusions**

The review suggests that ultrasonography is superior in RA assessment, enabling selection of RA segments with favourable morphological features, optimising surgical outcomes.

### A90 A Rare Case of Bi-atrial Myxoma via Multiple Fenestrations in the Atrial Septum

#### Salem, Agni, Miss; Shanmugananthan, Selvaraj, Mr

##### Liverpool Heart and Chest Hospital, Liverpool, UK

*Journal of Cardiothoracic Surgery* 2023, **18(Supp 1)**:A90

**Objectives**

Myxomas are a rare type of benign intracardiac tumour (between 66–80% occur in the left). Bi-atrial myxomas are even rarer (< 2.5% occurrence) and we would like to present such a rare and different case.

**Methods**

A 48 yr old female presented to her local hospital with a 6-month history of weight loss, pre syncopal episodes and shortness of breath. Computed tomography was performed that showed a large bi-atrial mass passing the mitral valve with partial extension into the left ventricle. The echocardiogram was suggestive of a 70 mm myxoma which was impinging on the mitral valve, resulting in mitral pseudo-stenosis. Through midline sternotomy and under cardiopulmonary bypass the tumour was confirmed to be bi-atrial invading through a multiple-fenestrated septum and involving the roof of the left atrium. Both atrium and septum were opened like a book and the myxoma was carefully excised in its entirety along with a rim of the septal tissue and part of the roof of left atrium. The septum and both atrium were reconstructed using bovine pericardia patch.

**Results**

The patient recovered well and the post-op echocardiogram showed no inter-atrial shunt and a normal mitral valve with no residual mass.

**Conclusions**

Although extremely rare, bi-atrial myxomas in a fenestrated atrial septum are amenable for safe surgical resection and successful reconstruction. It is therefore important to be aware of such cases and identify them early to provide appropriate surgical management.

Informed consent to publish had been obtained.
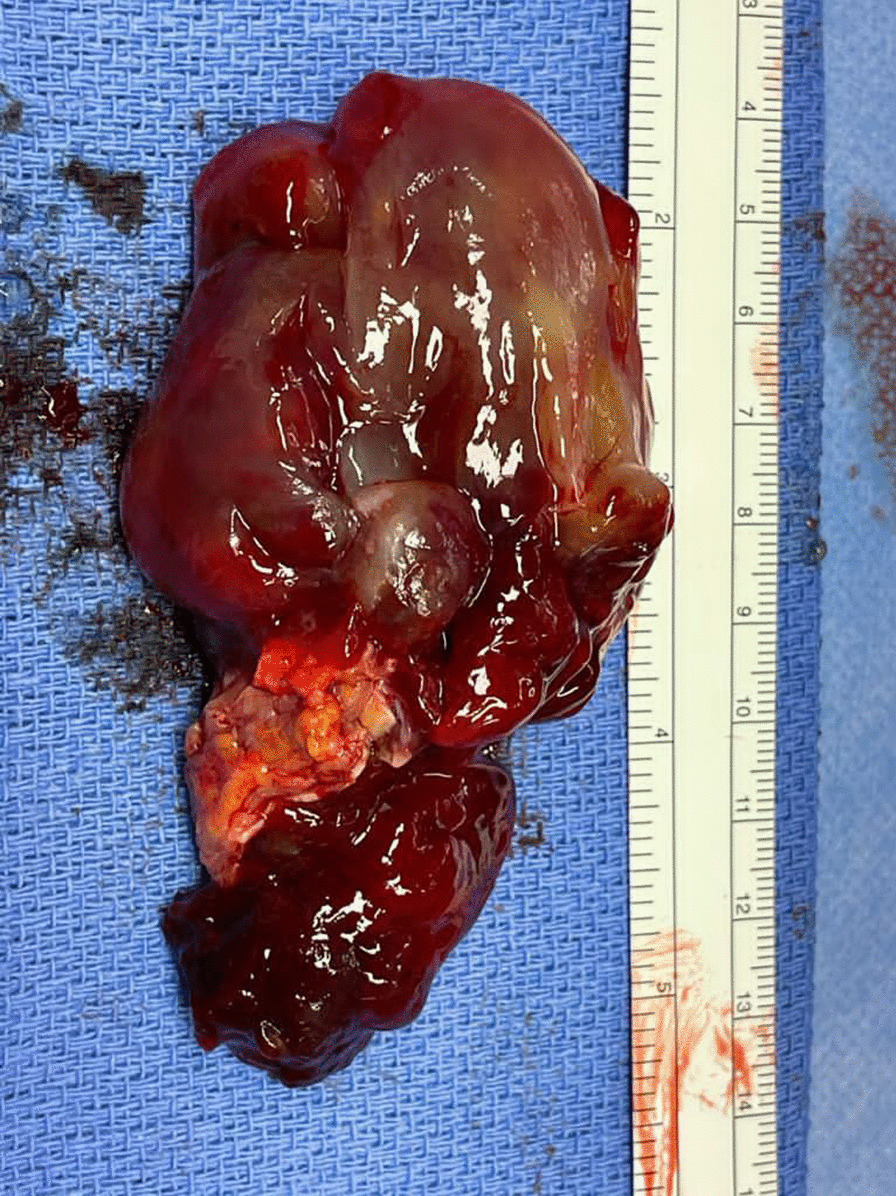


### A91 Audit and Quality Improvement of the Management of Hyponatraemia in Post-cardiopulmonary Bypass Surgical Patients

#### Samaraweera, Dulan^1^, Mr; Duval, Jean-Luc^1^, Dr; Crush, Jos^2^, Dr; Taghavi, John^1^, Mr

##### ^1^Royal Papworth Hospital NHS Foundation Trust, Cambridge, UK; ^2^West Suffolk NHS Foundation Trust, Bury Saint Edmunds, UK

*Journal of Cardiothoracic Surgery* 2023, **18(Supp 1)**:A91

**Objectives**

Hyponatraemia is the most common electrolyte abnormality in hospitalised patients and is associated with increased morbidity and mortality. Post-cardiopulmonary bypass (CPB) patients are particularly prone to developing hyponatraemia due to massive fluid shifts, diuresis, and the possibility of co-existing pump failure. We sought to increase the recognition and appropriate management of hyponatraemia to reduce its adverse sequelae.

**Methods**

Hyponatraemia was defined as serum sodium below 133 mmol/L. All patients undergoing surgery with CPB in a calendar month were retrospectively examined for the development and management of hyponatraemia. Interventions included teaching sessions, the introduction of a flow chart for early recognition/management of hyponatraemia by junior staff and optimisation of medicinal prescriptions were done over four months period. Their effectiveness was reassessed in the following calendar month.

**Results**

The first and second cycles included 146 and 123 patients with comparable demographics.

**Table:** 

	Parameter	First cycle	Second Cycle	Total
Preoperative risk factors	Mean Age (years)	66.5	66.8	
	No. of Females (%)	31(21.2%)	34(27.6%)	65(24.5%)
	Pre-op renal impairment	16(11%)	12(9.8%)	28(10.4%)
	Hypothyroidism	12(8.2%)	7(5.7%)	19(7.1%)
Type of surgery	CABG (isolated and combined)	70(47.9%)	59(48.0%)	129(47.9%)
	Valve surgery (including redo)	38(26.0%)	34(27.6%)	72(26.8%)
	PTE (pulmonary thromboembolectomy)	17(11.6%)	13(10.5%)	30(11.1%)
	Complex aortic surgery including aortic dissection	16(11%)	14(11.4%)	30(11.1%)
	Other	5(3.4%)	3(2.4%)	8(2.9%)

Ten had preoperative hyponatraemia (3 and 7 from each group). Fifty-eight (39.7%) and 46(37.4%) developed hyponatremia, with eight (5%) and one (0.8%) classified as severe (Na < 125 mmol/L) from respective groups. Symptoms were observed amongst Seven and three from patient groups while three and one patients from each exhibiting complications attributable to hyponatraemia.

Overall, patients would commonly develop hyponatraemia on postoperative day 3 (median). Those with hyponatraemia would stay an extra day in the hospital (11 vs 10). Thirty-day mortality was five (3.4%) and four (3.3%) for two groups, and one from each group had hyponatraemia. Female sex, advanced age, preexisting renal failure and hypothyroidism increased the risk for developing hyponatraemia.

**Conclusions**

Hyponatraemia is a common finding amongst post-CPB patients. Increasing awareness and promoting early intervention may reduce the severity and improve outcomes.

### A92 Atrial Fibrillation Ablation: A Single Surgeon Real-world Experience

#### Massey, John, Mr; Sharkey, Annabel, Ms; Hunter, Steven, Mr

##### Northern General Hospital, Sheffield, UK

*Journal of Cardiothoracic Surgery* 2023, **18(Supp 1)**:A92

**Objectives**

Atrial fibrillation (AF) is a major cause of morbidity and mortality. Surgical ablation for AF has shown to be effective at restoring sinus rhythm with resulting improvement in quality of life. There have been a number of studies comparing varying lesion sets, ablation modalities and techniques for dealing with the left atrial appendage. Our objective is to show that a single surgeon with a high volume of AF ablation practice can demonstrate high rates of freedom from AF.

**Methods**

Data was gathered retrospectively from a prospectively populated database between September 2013 and March 2021. Patients were stratified according to whether AF ablation was undertaken as a lone procedure or with concomitant cardiac surgery. The primary endpoint was freedom from AF at discharge from surgical follow-up (6-12 months). Secondary endpoints included 30-day mortality, long-term survival, rate of permanent pacemaker implantation and rate of DC cardioversion.

**Results**

317 patients underwent AF ablation (88 AF ablation only, 229 as a concomitant procedure). 60.8% of patients had long-standing persistent AF. Freedom from AF; at first follow-up (6 weeks to 3 months) 79.9%, at clinic discharge (6 months to 1 year) 83%. Rate of DCCV; in-patient 5.7%, out-patient 11.7% (58.8% success rate). 3.8% of patients had a catheter ablation post-operatively with 66% maintaining sinus rhythm at one year. Rate of PPM 5.9%, 30-day mortality 2.2% (average logistic EuroSCORE 6.55%) (overall survival see Fig. 1).

**Conclusions**

This study shows that in a real-world setting surgeons undertaking high volumes of AF ablation can achieve high rates of success with minimal complications. Our standard practice is test for entry and exit block at the end of every procedure.

### A93 The Single Clamp Box With Radio-frequency Device vs Conventional Box Lesion for Atrial Fibrillation Ablation

#### Massey, John, Mr; Sharkey, Annabel, Ms; Hunter, Steven, Mr

##### Northern General Hospital, Sheffield, UK

*Journal of Cardiothoracic Surgery* 2023, **18(Supp 1)**:A93

**Objectives**

Bipolar radiofrequency devices are commonly used to perform the pulmonary vein isolation and left atrial posterior wall lesions during surgical atrial fibrillation ablation. Usually, this is performed by clamping each line of the lesion set separately. The single clamp box lesion (which avoids opening the left atrium) has been described by our centre. We aim to show that the single clamp box is as effective as the standard box lesion in terminating atrial fibrillation.

**Methods**

Data was gathered retrospectively (September 2013 and March 2021). Patients were stratified according to whether they underwent a standard box lesion or a single clamp box lesion; patients were subdivided as to whether they underwent a full Cox-Maze IV or just the left-sided lesion set. The primary endpoint was freedom from atrial fibrillation at discharge from surgical follow-up (6-12 months). Secondary endpoints were x-clamp time, cardio-pulmonary bypass times, length of stay in hospital and rate of implantation of permanent pacemaker.

**Results**

138 patients (123 cox maze IV, 15 left-sided lesions only) underwent a single clamp box radiofrequency ablation, with freedom from atrial fibrillation at discharge from clinic of 88%. 123 patients had single clamp cox maze IV and 76 had standard cox maze IV (separate lesions to complete the box), freedom from atrial fibrillation 86.8% vs 88% (p = 1.0). No statistically significant difference in x-clamp time, cardio-pulmonary bypass time, length of stay in hospital or permanent pacemaker rate. 15 patients underwent a single clamp left-sided box only vs 116 patients who underwent standard left side lesion set alone. Freedom from atrial fibrillation 100% vs 79.2% (p = 0.58). No statistically significant difference in x-clamp time, cardio-pulmonary bypass time, length of stay in hospital or permanent pacemaker rate.

**Conclusion**

We have shown that the single clamp box is equivalent to the standard box when using radio-frequency for AF ablation.

### A94 Consent in Cardiac Surgery: A National Multicenter Audit Cardio-Thoracic Interdisciplinary Research Network

#### Abbasciano, Riccardo^1^, Mr; Al Attar, Nawwar^2^, Prof; Alam, Ruhina^3^, Miss; Alkalbani, Rawa^4^, Miss; Ansaripour, Ali^5^, Dr; Anzaar, Ahamed Akkeel^6^, Mr; Argyriou, Amerikos^7^, Mr; Avlonitis, Vassilios^8^, Mr; Bhudia, Sunil^9^, Mr; Booth, Karen^10^, Ms; Brown, Joshua^11^, Mr; Chan, Jeremy^12^, Mr; Dandekar, Uday^13^, Mr; Dearling, Jeremy^14^, Mr; Deehan, Blathnaid^15^, Mr; Di Tommaso, Ettorino^16^, Mr; Dixon, Lauren^17^, Miss; Gradinariu, George^18^, Mr; Green, Jordan^19^, Dr; Harky, Amer^20^, Mr; Harrington, Bertie^21^, Mr; Hasan, Ragheb^22^, Mr; Horsfall, Gregory^23^, Dr; Jawarchan, Angila^24^, Miss; Jones, Mark^25^, Mr; Kho, Jason^26^, Mr; Kumar, Pankaj^27^, Mr; Layton, Georgia R.^28^, Miss; Limbachia, Devan D.^29^, Mr; Mahoud, Loubani^30^, Prof; Makam, Rishab^31^, Mr; Moawad, Karim R.^32^, Mr; Modi, Amit^33^, Mr; Morais, Carlos^34^, Mr; Murphy, Gavin^35^, Prof; Nguyen, Bao^36^, Mr; Nwaejike, Nnamdi^37^, Mr; Petrou, Mario^38^, Mr; Philip, Bejoy^39^, Mr; Rajakaruna, Cha^40^, Mr; Rizzo, Victoria^41^, Miss; Rochon, Melissa^42^, Miss; Rogers, Luke J.^43^, Mr; Sayeed, Rana^44^, Mr; Singhania, Asmita^45^, Miss; Vaja, Ricky^46^, Mr; Wali, Anuj^47^, Mr; Wilson, Keith^48^, Mr; Wilson, Ian^49^, Mr; Zakkar, Mustafa^50^, Mr

##### ^1^Leicester Clinical Trials Unit, Leicester, UK; ^2^University Hospitals of Leicester, Leicester, UK; ^3^Golden Jubilee National Hospital, Glasgow, UK; ^4^Royal Sussex County Hospital, Brighton, UK; ^5^Oxford University Hospital, Oxford, UK; ^6^Liverpool Heart and Chest Hospital, Liverpool, UK; ^7^Manchester Royal Infirmary, Manchester, UK; ^8^Guy's and St Thomas' NHS Foundation Trust, London, UK; ^9^Royal Brompton & Harefield NHS Foundation Trust, London, UK; ^10^The Newcastle upon Tyne Hospitals NHS FT, Newcastle, UK; ^11^Belfast Royal Victoria, Belfast, UK; ^12^Swansea Bay University Healthboard, Swansea, UK; ^13^University Hospital Coventry and Warwickshire NHS Trust, Coventry, UK; ^14^Patient & Public Involvement Initiative; ^15^Bristol Heart Institute, Bristol, UK; ^16^Hull and East Yorkshire Hospitals NHS Trust, Hull, UK; ^17^University Hospitals Plymouth NHS Trust, Plymouth, UK; ^18^Wythenshawe Hospital, Manchester, UK; ^19^John Radcliffe Hospital, Oxford, UK; ^20^Imperial College London, London, UK

*Journal of Cardiothoracic Surgery* 2023, **18(Supp 1)**:A94

**Objectives**

Patient and Public Involvement and Engagement events coordinated following the Priority Setting Partnership and identification of "Infection Prevention" as a key research priority has recently generated intrigue around the consent process for adult cardiac surgery. Discussions have eluded to the sentiment that "patients cannot provide wholly informed consent without being aware of all the associated risks and their incidence". This view has been upheld with updates in the legal standard expected following the Montgomery case in 2015. This Supreme Court judgement ruled that doctors "must take reasonable steps to ensure that patients are aware of all the risks that are material to them". This national multicentre audit aimed to illustrate and describe the current practise of the consent process in adult cardiac surgery.

**Methods**

Consent forms and clinic letters were prospectively reviewed for all consecutive patients undergoing cardiac surgery over a 2-week period between 18th – 31st October '21. Data relating to the type of surgery, grade of individual taking consent, documented risks and quantification of this risk were collected.

**Results**

Seventeen (/35) UK cardiac centres participated and a total of 420 consecutive patients were included. The urgency of the cases was elective (44.5%), urgent (50%) and emergency (6.2%). 236 patients were reviewed in clinic preoperatively and of these the risks of surgery were documented in 60.2% of clinic letters. Four centres used a pre-filled document/sticker whilst the remainder were handwritten. The consent form was signed by a consultant (27.1%), SpR (69.8%) and SHO (3.1%). A summary of the commonly documented risks and quantification of these risks is provided.

**Conclusions**

Variation in both the complications documented and quantified risk of these complications occurring exists across UK practise in adult cardiac surgery. What do patients want and need to know to provide informed consent?

**Table:** 

Complication	Documented (%)	Quantified risk documented (%)
Mortality	99.3	80.8
Stroke	92.9	57.9
MI	54.5	20.5
Renal failure	67.4	17
Arrhythmias	77.6	17.8
Wound infection	82.6	12.7
Bleeding	86.4	18.7

### A95 Association of Low Haemoglobin Prior to Elective Cardiac Surgery With Need for Blood Transfusion and Outcomes

#### Shoeib, Mohamed, Mr; Mahmood, Zahid, Mr

##### NHS Golden Jubilee, Glasgow, UK

*Journal of Cardiothoracic Surgery* 2023, **18(Supp 1)**:A95

**Introduction**

Pre-operative anaemia is a common finding in patients undergoing cardiac surgery (20–30%) (Hogan M. et al., 2014). The degree of preoperative and intraoperative anaemia is correlated to increased morbidity and in-hospital mortality in patients undergoing elective cardiac surgery (Ranucci M et al., 2013). Blood markers suggest that > 50% of cases of pre-operative anaemia are related to Iron deficiency, with outcome improvement following IV iron replacement therapy (Hung, M. et al., 2015).

**Objectives**

Assess the association of anaemia with use of pre-and post-operative blood transfusions, length of ICU and hospital stays and mortality. To improve recognition and management of anaemia prior to elective cardiac surgery.

**Methods**

Retrospective study of 1577 patients post elective cardiac surgery (CABG and/or valve surgery) in our institution during the period from January 2016 till January 2018. We examined the associations anaemia has with; the need of blood transfusions (PRBC), lengths of stay (ICU and in-hospital) and mortality (30 day and 1 year).

**Results**

Compared to patients without anaemia, those who had anaemia required transfusion more often (60% vs 35%, P < 0.001) and received more units of bloods (median (IQR): 1 (0–2) vs 0 (0–1); p < 0.001). Pre-operative Hb levels were inversely correlated with Age (*P* < 0.001), total days in hospital (*p* < 0.001) and hours in intensive care unit (*p* < 0.016).

**Conclusions**

Preoperative screening and optimization of haemoglobin level would significantly preserve resources and minimize the risks linked to postoperative blood transfusion. Preoperative anaemia was linked to significant increase of postoperative blood transfusion in both men and women. However, no significant difference in prevalence of preoperative anaemia based on gender.

### A96 Severe Factor XII Deficiency in the Context of Cardiopulmonary Bypass: The Challenges of Intraoperative Heparin Monitoring

#### Theodore, Sigrid, Dr; Haworth, Kobi, Dr; Scarrott, Helen, Ms; Butler, Chris, Dr; Shah, Pallav, Dr

##### Townsville University Hospital, Queensland, Australia

*Journal of Cardiothoracic Surgery* 2023, **18(Supp 1)**:A96

**Objective**

Severe factor XII (FXII) deficiency causes an elevated baseline activated clotting time (ACT). Options for intraoperative heparin monitoring during cardiopulmonary bypass (CPB) include normalising the ACT with fresh frozen plasma (FFP) prior to heparinisation or the use of anti-factor Xa (anti-Xa) levels. We present a case comparing these methods with the use of rotational thromboelastometry (ROTEM).

**Methodology**

Baseline blood samples were collected from a patient with severe FXII deficiency undergoing CPB. A single dose of FFP was given prior to heparinisation, followed by successful CPB and protamine reversal. Matched ACT, anti-Xa and ROTEM samples were collected intraoperatively and the results compared. Serial post-operative activated partial thromboplastin time (aPTT) samples illustrated the time course of FXII level decline.

**Results**

Elevated baseline aPTT and ACT normalised following the administration of FFP. Subsequent ACT, anti-Xa and ROTEM levels reflected the anticipated coagulation status. Anti-Xa levels were labour intensive and delayed the initiation of CPB. ROTEM results were proportional to the ACT and anti-Xa results, but did not influence clinical management. The patient had no major complications and was discharged six days postoperatively. The discharge aPTT level remained below baseline (82 s at 144 h) following a single intraoperative FFP dose.

**Conclusion**

Administration of FFP followed by point-of-care ACT testing was the most efficient method of intraoperative heparin monitoring. ROTEM provided confirmation of coagulation status but was inadequate as a sole technique for intraoperative heparin monitoring.

### A97 A Proposed Method to Widen Participation in Cardiothoracic Surgery Across UK Medical Schools

#### Sahdev, Nikhil, Dr; Zibdeh, Omar, Dr; Raja, Shazad, Mr

##### Royal Brompton and Harefield Hospital, London, UK

*Journal of Cardiothoracic Surgery* 2023, **18(Supp 1)**:A97

**Objectives**

Cardiothoracic surgery (CTS) does not form part of the undergraduate curriculum in majority of medical schools across the UK. Of the small proportion that do, there is still relatively minor exposure compared with other specialties. This coupled with the fact there are fewer centres that offer CTS, makes it challenging for students to access the speciality. Furthermore, from 2023, CTS is only accessible via ST1 entry; therefore, students have a limited time frame to explore the speciality to enable informed career planning. Proposed is an educational model that can be implemented across the country that would allow students to discover CTS and its pathway to entry.

**Methods**

In most MBBS programme specifications there is flexibility for students to choose a specific module to study (student-selected component). This 6-week model would allow students opportunities to assist on the wards, clinic and theatre, becoming active members of the cardiothoracic team. Importantly they will spend time with doctors of all grades to understand what the speciality entails. Teaching sessions will encourage active learning in the form of bedside teaching, simulation sessions and on-calls. Uniquely, there will also be workshops surrounding the entry pathway into CTS.

**Results**

To measure the efficacy of the model, we will analyse short and long-term results. In the short-term we will assess the effectiveness of the clinical content taught by arranging a pre and post-course examination. Similarly, a pre and post-module questionnaire will be utilised to gauge interest in CTS and knowledge pertaining to the entry pathway. In the long-term we will assess whether our model contributed to students to apply to CTS by following students up in 3 years’ time.

**Conclusion**

This peer-reviewed educational model can easily be replicated across medical schools allowing students to gain increased exposure to CTS. Thus, attracting hardworking and committed individuals to the speciality.

## Adult Cardiac Mitral Valve

### A98 Use of Anti-Thrombotic Medications After Heart Valve Surgery: A Cross-Sectional Survey of Contemporary Practice in the UK

#### Shah, Benoy^1^, Dr; Laskar, Nabila^2^, Dr; Akowuah, Enoch^3^, Mr; Briffa, Norman^4^, Prof; Cartwright, Neil^4^, Mr; Kendall, Simon^3^, Mr; Chambers, John^5^, Prof

##### ^1^University Hospital Southampton, Southampton, UK; ^2^Barts Heart Centre, London, UK; ^3^South Tees Hospitals NHS Trust, Middlesbrough, UK; ^4^Northern General Hospital, Sheffield, UK; ^5^Guy's & St.Thomas' Hospitals, London, UK

*Journal of Cardiothoracic Surgery* 2023, **18(Supp 1)**:A98

**Objectives**

North American and European guidelines vary in their recommendations on use of anti-thrombotic drugs after heart valve surgery. The aim of this cross-sectional survey was to understand current practice amongst UK (UK) cardiac surgeons.

**Methods**

Using the SCTS database, NHS hospital websites and direct e-mail confirmation, all UK consultant cardiac surgeons were e-mailed a link to an online survey. The survey asked their current practice regarding use of anti-platelet and/or anticoagulant drugs (and their duration) following bioprosthetic aortic valve replacement (AVR), mitral valve replacement (MVR) and mitral valve repair (MVrep) for patients in sinus rhythm with no other clinical indication for antithrombotic medications. We also asked about choice of anticoagulant (warfarin vs NOAC) in patients undergoing MVrep that are in atrial fibrillation (AF).

**Results**

We identified 260 consultant cardiac surgeons in the UK, of whom 103 (40%) replied to the survey. We found wide variation in practice amongst surgeons in all fields. After AVR, the main answers were: lifelong aspirin (64%); 3 months aspirin (25%); and 3 months anticoagulation followed by lifelong aspirin (8%). After MVR, the choices were: anticoagulation for 3 months then lifelong aspirin (37%); lifelong aspirin only (35%); 3 months anticoagulation only (16%); and 3 months aspirin only (10%). After MVrep in sinus rhythm, the choices were: lifelong aspirin (42%); 3 months anticoagulation then lifelong aspirin (26%); and 3 months anticoagulation only (19%). After MVrep for AF patients: surgeons recommended warfarin (38%), a NOAC (37) or either warfarin or a NOAC (25%).

**Conclusions**

There are wide variations in practice across the UK regarding use of anti-thrombotic drugs after heart valve surgery. This reflects a lack of high-quality evidence and underscores the need for randomized trials to address these questions.

### A99 In-hospital Mortality From Right Ventricle (RV) Failure Post Mitral Valve Surgery, an Observational Exploratory Analysis

#### Apicella, Giulia, Miss; Abbas, Sherif, Mr; Szafranek, Adam, Mr; Naik, Surendra, Mr; Boulemden, Anas, Mr; Nicou, Niki, Miss; Birdi, Inderpaul, Mr; Qureshi, Saqib, Mr

##### Nottingham City Hospital, Nottingham, UK

*Journal of Cardiothoracic Surgery* 2023, **18(Supp 1)**:A99

**Introduction**

Acute RV dysfunction post mitral valve surgery can have catastrophic outcome and the pathogenesis remains unclear. We reviewed our experience with aims of identifying the underlying explanatory clinical characteristics.

**Methods**

Multivariate logistic regression analyses of peri and post-op including echocardiographic characteristics of mitral valve cases either isolated or concomitant (coronary, tricuspid and aortic valve surgery) between 1996 and 2019 that died in hospital after index mitral valve surgery were undertaken.

**Results**

A total of 1748 patients underwent mitral valve surgery. Overall sixty-three (3.6%) patients died during index hospital admission. Forty-two (62%) patients retained their normal RV function post-op and died of unrelated causes. Sixteen patients (23.5%) had impaired RV function pre-op and 43% of them died of cardiac failure. Pre-op RV impairment was strongly associated with significant tricuspid regurgitation requiring concomitant correction: odds ratio (95% confidence interval); 6.6(1.1, 38.5) p = 0.03 and ischemic mitral pathology; 6.2 (1.4, 27.5) p = 0.016. Five patients (7.4%) had new onset post-op RV failure of unexplained etiology and died of this. In the multivariate regression analyses; age, sex, logistic EuroSCORE, bypass and cross-clamp times, pulmonary hypertension, mitral with or without concomitant tricuspid valve surgery, mitral repair vs. replacement and ischemic or non-ischemic etiologies were deemed non-significant predictors of acute post mitral RV failure.

**Conclusions**

Whereas impaired RV is often encountered in mitral valve ± tricuspid valve cases, sudden catastrophic RV failure in these patients with preserved RV pre-op is uncommon. The traditional operative and non-operative factors fail to be strong contenders to predict this behaviour of the right ventricle.

### A100 Mitral Valve Surgery for Degenerative Mitral Valve Regurgitation in Patients with Left Ventricular Dysfunction: A Systematic Review and Meta-analysis

#### Mohamadzade, Navid^1^, Mr; Montaque, Morgan^1^, Mr; Bruno, Vito D.^2^, Dr; George, Sarah^1^, Prof

##### ^1^Bristol Medical School – Translational Health Sciences – University of Bristol, Bristol, UK; ^2^Bristol Heart Institute, Bristol, UK, University Hospitals of Bristol and Weston NHS Foundation Trust, Bristol, UK

*Journal of Cardiothoracic Surgery* 2023, **18(Supp 1)**:A100

**Introduction**

Degenerative mitral regurgitation (DMR) precipitates left ventricular dysfunction (LVD), which deleteriously impacts post-operative outcomes following mitral valve (MV) surgery. Current clinical guidelines provide weak evidence supporting surgery in patients with LVD. This meta-analysis aimed to investigate the short/long-term outcomes after MV surgery for DMR patients with a reduced left ventricular ejection fraction (LVEF) compared to a normal LVEF.

**Methods**

In accordance with the Preferred Reporting Items for Systematic Reviews and Meta-analysis statement, a pre-defined protocol was used to conduct a systematic review of the literature across three databases. Raw data extraction and pooled analyses for odds/hazard ratios (OR/HR) were performed for: in-hospital/30-day mortality; long-term survival; and post-operative major adverse cardiovascular and cerebrovascular events (MACCE).

**Results**

A meta-analysis was conducted using 10 observational cohort studies. Pooled analyses for the following outcomes were calculated by combining all reduced LVEF percentage cut-offs from all studies. In-hospital/30-day mortality had a pooled OR of 6.50[2.87–14.72]. Pooled HRs for overall long-term survival and post-operative MACCE were 2.36[1.82–3.06] and 1.97[1.43–2.70], respectively. Long-term survival in only patients with LVEF ≤ 60% had a pooled HR of 2.00[1.47–2.73].

**Conclusion**

DMR patients with reduced LVEF experience significantly worse short/long-term post-operative outcomes compared to those with normal LVEF. Future studies are required to elucidate whether surgery is more beneficial than conservative management in these patients.

### A101 Propensity Matched Comparison of Outcomes Following Minimally Invasive vs Conventional Mitral Valve Repair

#### Folaranmi, Omowumi^1^, Dr; Allam, Mohamed^1^, Mr; Kendall, Simon^1^, Mr; Goodwin, Andrew^1^, Mr; White, Ralph^1^, Mr; Takyi, Christopher^2^, Dr; Akowuah, Enoch^1^, Mr

##### ^1^South Tees Hospitals NHS Foundation Trust, Middlesbrough, UK; ^2^Newcastle University Medical School, Newcastle, UK

*Journal of Cardiothoracic Surgery* 2023, **18(Supp 1)**:A101

**Objectives**

Recent NICE guidelines suggest minimally invasive mitral valve repair surgery should be offered to all patients who are suitable but comparative data supporting this approach is lacking. The aim of this study was to compare outcomes following both minimally invasive and conventional approaches to mitral valve repair.

**Methods**

This study retrospectively compared outcomes between patients undergoing isolated mitral valve repair at our institution between 2015 to 2020. Euroscore II was used to generate the propensity scores for patients in the conventional and minimally invasive groups. Of 238 patients, 1:1 propensity score matching was performed for the closest neighbours yielding 152 matched cases in total with 76 patients in each group.

**Results**

For the 152 matched patients, there was no difference in mean Euroscore II (1.53 ± 1.02 (p = 1.000). There were no cases of in-hospital mortality in the minimally invasive group, and 1 case out of 76 in the conventional group. Mean total length of hospital stay was significantly lower in the minimally invasive group (6.68 ± 3.61vs8.62 ± 6.08, p < 0.014). Following surgery, patients with mild residual mitral regurgitation or less were 94.7% in the minimally invasive group vs 96.1% in the conventional group, p = 0.6804. Incidence of re-operation for bleeding was not significantly different (2.6% in the minimally invasive group vs 3.9%, p = 0.6513) neither was the rate of blood transfusion at 9.2% in both groups.

**Conclusion**

This study shows that minimally invasive mitral valve repair is as safe as conventional mitral valve repair. A significant difference in length of stay after surgery may have resource and quality of life implications.

### A102 Redo Surgery for the Mitral Valve: Can be Performed Safely and Offers Good Long-term Survival

#### Massey, John, Mr; Sharkey, Annabel, Ms; Braidley, Peter, Mr

##### Northern General Hospital, Sheffield, UK

*Journal of Cardiothoracic Surgery* 2023, **18(Supp 1)**:A102

**Objectives**

Redo-surgery to address mitral valve pathology in patients who have undergone previous cardiac surgery remains the gold standard. Although the procedure is demanding we aim to show that it can be performed safely with good long-term survival in an era where transcatheter procedures are becoming more available.

**Methods**

Data was gathered from a prospectively populated database between January 2007 and July 2021. All patients undergoing redo-surgery to deal with the mitral valve, performed by a single consultant were included. The primary endpoint was mortality. Secondary end-points include in-hospital length of stay, in-hospital mortality and catastrophic surgical re-entry.

**Results**

93 patients met the inclusion criteria; 57 isolated mitral valve surgery, 23 mitral + concomitant surgery, 13 repair of paraprosthetic leak. 76% of patients had undergone a previous mitral procedure (35% replacement, 58% repair, 7% valvotomy), 17% had a patent LIMA to LAD. 18% had pulmonary hypertension, 4% of patients had ischaemic MR and 12% had functional MR (26% had previous coronary artery surgery). 99% had redo-sternotomy, 1% had right thoracotomy. 2% of patients had a catastrophic re-entry, median length of stay 9 days (3–135), 2% in hospital mortality (average logistic euroscore 18.67%), median long-term survival 182 months by Kaplan–meier.

**Conclusion**

This study illustrates that redo-surgery to deal with the mitral valve is safe with a low in-hospital mortality despite this being a high-risk patient population. We have also demonstrated that these patients go on to have good long-term survival.

### A103 Does Concomitant Tricuspid Valve Repair Impact the Outcomes of Minimal Access Endoscopic Mitral Valve Surgery?

#### Karuppannan, Mukesh, Mr; Abdelrahman, Abdelbar, Mr; Saravanan, Palanikumar, Dr; Knowles, Andrew, Dr; Laskawski, Grzegorz, Mr; Argyle, Rachel, Dr; Zacharias, Joseph, Mr

##### Blackpool Victoria Hospital, Blackpool, UK

*Journal of Cardiothoracic Surgery* 2023, **18(Supp 1)**:A103

**Objective**

The objective of this study was to review the impact of concomitant tricuspid valve surgery (TVS) on short and long-term outcomes of endoscopic mitral valve surgery (EMVS).

Methods

Patients who underwent endoscopic minimally invasive mitral valve surgery between 2007 and 2020 at a single institution were reviewed. Patients were primarily grouped by those undergoing isolated EMVS against EMVS + TVS. Short and long-term outcomes were analysed from a prospectively collected departmental database.

**Results**

A total of 329 patients underwent EMVS out of which 52 (15.90%) underwent concomitant TVS. Patients undergoing EMVS + TVS were at higher risk at baseline (LogEUROscore 6.97 vs 3.74 for EMVS group). Cardiopulmonary bypass times (180.14 ± 32 vs. 164 ± 42 min; p < 0.001) and aortic occlusion times (125.92 ± 30 vs. 108.04 ± 36 min; p < 0.001) were longer in the EMVS + TVS group. Operative mortality was higher but acceptable and below the predicted mortality (1.92% for EMVS + TVS vs 0.72% isolated EMVS, p = 0.55). Permanent pacemakers were required more frequently in the EMVS + TVS group (0.37% vs 5.36% p < 0.03). All other complication rates were similar. Mean length of postoperative hospital stay was 9.34 days for EMVS + TVS while it was 7.19 days for EMVS group. Long term survival at 8 years was comparable (82.70% EMVS + TVS vs 88.09% for EMVS,p = 0.45).

**Conclusion**

Despite longer operative times, EMVS + TVS has similar postoperative outcomes to isolated EMVS.We would recommend addressing the tricuspid valve, if indicated, during an endoscopic mitral valve procedure.

**Table:** 

Perioperative variables	EMVS + TVS (n = 52)	EMVS(n = 277)	p-value
Age range(years)	37–88(70.65)	20–92(62.11)	0.03
Female {n, (%)}	35 (67.30%)	119 (42.96%)	0.02
Logistic Euroscore,{Mean (Range)}	6.97 (1.5–24.04)	3.74(1.5–43.23)	< 0.001
CPB time,mins (mean)	180.14 (SD 32)	164 (SD 42)	< 0.001
Aortic occlusion time,mins (mean)	125.92 (SD 30)	108.04 (SD 36)	< 0.001
30 day mortality {n, (%)}	1 (1.92%)	2 (0.72%)	0.54
Mean ICU stay(days)	1.27 (SD 0.64)	1.41 (SD 3.22)	0.003
Heart block requiring PPI {n, (%)}	3 (5.76%)	1 (0.36%)	0.03
Prolonged ventilation(> 48 h) {n, (%)}	1 (1.92%)	9 (3.24%)	0.02


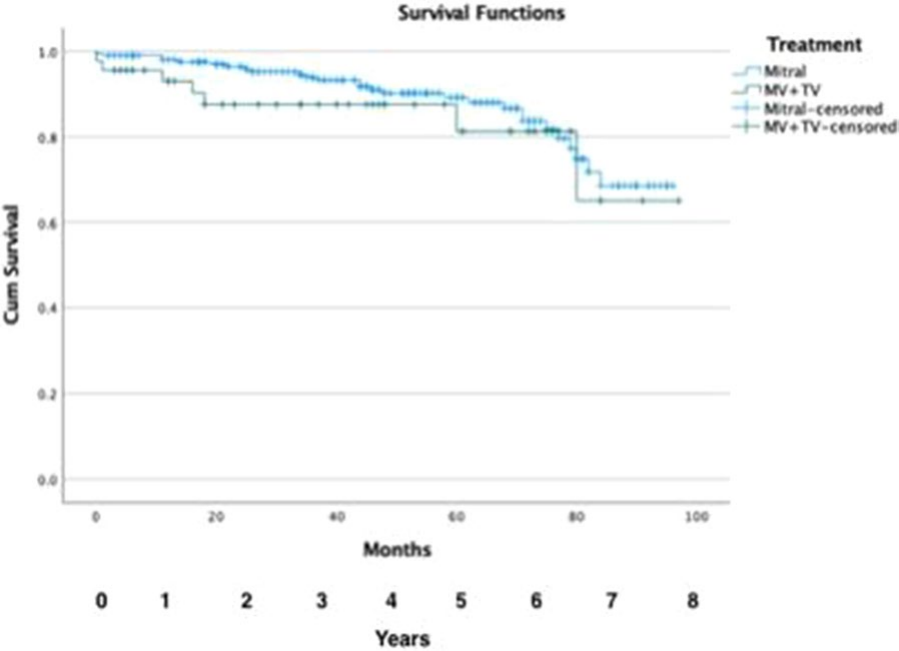


### A104 Current Practices in Mitral Valve Interventions Across the UK and Ireland

#### Naruka, Vinci^1^, Mr; Arjomandi Rad, Arian^2^, Mr; Chacko, Jacob^1^, Mr; Liu, Guiqing^1^, Mr; Afoke, Jonathan^1^, Mr; Punjabi, Prakash P^1^, Prof

##### ^1^Hammersmith Hospital, London, UK; ^2^Imperial College London, London, UK

*Journal of Cardiothoracic Surgery* 2023, **18(Supp 1)**:A104

**Objectives**

In recent years, major findings on concomitant procedures and perioperative management have occurred in Mitral Valve (MV) surgery. Therefore, we sought to evaluate the current practices in MV interventions across the UK and Ireland.

**Methods**

In September 2021, all consultant cardiac surgeons were identified through an electronic search of the SCTS database and sent an online survey of 14 questions. Data was recorded on a central database and analysed.

**Results**

80% out of 50 consultants participating indicated MV repair as their specialty. 66% performed > 150 operations/year and 54% had 10+ years of experience.

Anticoagulation post-MV repair: if sinus rhythm 40% use Aspirin while 20% use Vitamin K antagonist (VKA), both alone for 3 months only; if atrial fibrillation (AF) 44% use DOAC and 20% VKA, for life. In bioprosthetic MV replacement (MVR): if sinus rhythm 26% use VKA and 24% Aspirin, for 3 months only; if AF 38% use DOAC for life and 28% use VKA for 3 months followed by DOAC for life. 76% performed concomitant tricuspid valve repair for moderate tricuspid regurgitation with annular diameter > 40 mm. 54% indicated ischaemic MV surgery in patients undergoing CABG if moderate mitral regurgitation with ERO > 20mm2 and regurgitant volume > 30 ml. The preferred management was: MVR if predictors of repair failure identified (52%), downsizing annuloplasty ring (26%) with additional subvalvular procedures (14%). 63% of surgeons with 0–15 years’ experience prefer MVR if predictors of repair failure were identified while 60% with 15 + years’ experience preferred downsizing annuloplasty ring. For AF in cardiac surgery, 40% perform ablation with biatrial lesion and 22% with left-sided only. 86% perform concomitant Left Atrial Appendage Occlusion irrespective of AF ablation with a left atrial clip.

**Conclusion**

These results demonstrate a variable practice for MV surgery, and a degree of lack of compliance with surgical intervention guidelines and anticoagulation strategy.

### A105 Medium-term Outcomes of Surgical TOE-guided Mitral Valve Repair Surgery

#### Kho, Jason^1^, Dr; Metwalli, Amr^2^, Mr; Amin, Fouad^3^, Dr; Missouris, Constantinos^3^, Prof; Jin, Xu Yu^4^, Dr; Petrou, Mario^2^, Mr

##### ^1^St Thomas's, London, London, UK; ^2^Royal Brompton Hospital, London, UK; ^3^Wexham Park Hospital, Slough, UK; ^4^John Radcliffe Hospital, Oxford, UK

*Journal of Cardiothoracic Surgery* 2023, **18(Supp 1)**:A105

**Objectives**

To review the early to medium-term outcomes of patients who underwent mitral valve (MV) surgery performed by a dedicated mitral team.

**Methods**

We retrospectively reviewed the operative and survival data of consecutive patients who underwent MV surgery over a 10-year period (November 2011–2021) performed by a single surgeon (MP) guided by the same surgical TOE specialist (XYJ).

**Results**

This team performed a total of 272 MV operations; 163 were repairs (MVr) with mean age 63 ± 15 years and calculated mean EuroSCORE-II was 2.7 ± 3.0. All cases underwent two-stage planning of repair strategy by detailed TOE and surgical assessment. The repairs were resectional (49.7%) or non-resectional (50.3%); 97.5% included an annuloplasty. Cross-clamp and cardiopulmonary bypass times for isolated MVr ranged from 44 to 110 and 58 to 150 min respectively. Success rate after 166 attempted MVr was 98.2% (163/166) demonstrated as no or trivial mitral regurgitation, normal pressure gradient and valve area on post-bypass TOE. Mean survival after isolated MVr was 100% at 30 days and 98.0 ± 2.0% at 5 and 9 years. Freedom-from-reintervention at 9 years was 100% in entire cohort.

**Conclusion**

In our experience, meticulous pre-bypass TOE analysis and correlation with surgical patho-anatomy involving the same cardiac surgeon and dedicated TOE specialist results in excellent MV repair rates and medium-term outcomes.

### A106 Roboti Versus Conventional Mitral Valve Surgery: Do the Costs Outweigh the Benefits?

#### Ahern, Shane, Dr; NiDhonnchu, Tara, Ms

##### Department of Cardiothoracic Surgery, Cork University Hospital, Cork, Ireland

*Journal of Cardiothoracic Surgery* 2023, **18(Supp 1)**:A106

**Objectives**

Comparisons between robotic and conventional mitral valve surgery have varied. The former confers many clinical benefits to patients. However, the high costs associated with the robotic approach act as a deterrent to its uptake. Therefore, the objectives of this research are to identify the intraoperative and post-operative differences, as well as cost differences between robotic and conventional mitral valve surgery.

**Methods**

A systematic review of five databases was performed to identify research comparing robotic and conventional mitral valve surgery. Meta-analysis of clinical and cost data was carried out.

**Results**

Fourteen studies with a combined population of N = 3,635 were included. Meta-analysis revealed significantly longer cardiopulmonary bypass and cross-clamp times for robotic surgery, but many significantly improved clinical outcomes. These included improved all-cause mortality, decreased duration of ventilation, shorter ICU and overall length of stay. Operative costs were significantly higher for robotic surgery but were offset by post-operative savings.

**Discussion**

The increased complexity associated with robotic mitral valve surgery significantly increase operative times and expense. Despite this, robotic surgical led to improved post-operative outcomes. This translated to significant saving in costs in the post-operative period, enough to offset the high intraoperative costs, making robotic surgery increasingly attractive.

### A107 Open Transatrial Transcatheter Mitral Valve Replacement in Patients with Mitral Annular Calcification Undergoing Concomitant Aortic Valve Replacement

#### Holland, Luke^1^, Mr; Narayana, Ashok^2^, Mr; Hildick-Smith, David^2^, Prof; Trivedi, Uday^2^, Mr

##### ^1^Guy's Hospital, London, UK; ^2^Royal Sussex County Hospital, Brighton, UK

*Journal of Cardiothoracic Surgery* 2023, **18(Supp 1)**:A107

**Objectives**

Mitral valve (MV) surgery in the presence of mitral annular calcification (MAC) remains a surgically challenging procedure with well-recognised complications including paravalvular leak, atrioventricular fistula, left ventricular rupture and coronary artery injury. Morbidity and mortality from surgical valve replacement in this context is significant, reflective of the technical challenges and co-morbidities often present in this patient population. In recent years, reports of antegrade transcatheter MV implantation using balloon-expandable prostheses have emerged.

**Methods**

We present a series of four patients with MAC who underwent double (mitral and aortic) valve replacement with a surgical aortic valve and an open transcatheter mitral valve. We discuss technical points of surgery and reflect on lessons learned from our experience.

**Results**

All four patients underwent surgery via median sternotomy. All four had technical and procedural success, as defined by the Mitral Valve Academic Research Consortium. The 30-day mortality was zero, although there was one late mortality after a prolonged intensive care admission. The three patients who survived to discharge had no paravalvular leak on follow-up echocardiography.

**Conclusions**

Our experience suggests patients with aortic and mitral stenosis in the presence of MAC can be treated with a combined operation incorporating surgical AVR and a balloon-expandable "TAVI-in-MAC". This adds to the growing body of evidence supporting the use of these valves during open cardiac surgery.

### A108 Surgical Outcomes for Patients With and Without Mitral Annular Disjunction who Undergo Mitral Valve Surgery

#### Kwok, Chun Shing, Dr; Bennett, Sadie, Mrs; Tafuro, Jacopo, Mr; Brumpton, Marcus, Dr; Bardolia, Caragh, Dr; Heatlie, Grant, Dr; Duckett, Simon, Dr; Ridley, Paul, Mr; Nanjaiah, Prakash, Mr

##### University Hospitals of North Midlands, Stoke-on-Trent, UK

*Journal of Cardiothoracic Surgery* 2023, **18(Supp 1)**:A108

**Objectives**

The objective of the study is to determine the prevalence of mitral annular disjunction (MAD) in patients who undergo mitral valve surgery and surgical outcomes for these patients.

**Methods**

All patients who underwent mitral valve surgery between 2013 and 2020 and had pre-surgical transthoracic echocardiographic images that could be reviewed were included.

**Results**

A total of 185 patients were included in the analysis and 32.4% (n = 60) patients had MAD (average length 8.5 mm). No differences were observed comparing MAD to no MAD according to age, sex and comorbidities but patients without MAD had significantly higher surgical risk scores. Coronary artery bypass grafting took place in 19.5% of patients and 60.5% had a ring annuloplasty. A greater proportion of patients with MAD had to return to theatre but this was not statistically significant (10.0% vs 4.1%, p = 0.18). There was no difference in in-hospital complications and one-year mortality. Two patients out of 51 patients with follow-up echo scans had MAD post-surgery.

**Conclusions**

MAD is common in patients who undergo mitral valve surgery. Patients with MAD have similar surgical outcomes to patients without MAD. Surgery on the mitral valve in patients with MAD appears to correct the structural abnormality at follow-up.

### A109 Minimally Invasive Mitral Valve Repair: A Systematic Review and Meta-analysis of Randomised, Active Control Trials

#### Ganesananthan, Sharmananthan^1^, Mr

##### ^1^University College London, London, UK; ^2^West Middlesex University Hospital, Middlesex UK

*Journal of Cardiothoracic Surgery* 2023, **18(Supp 1)**:A109

**Objectives**

The conventional approach for mitral valve surgery is midline sternotomy (MS), but recent observational data have suggested comparable safety of minimally invasive (MI) approaches. Hence, we sought to pool the outcomes of randomised, active control trials (RCTs) to better elucidate outcomes of the MI approach.

**Methods**

Embase, Medline, Google Scholar and PubMed databases were searched from conception to October 2021 for RCTs comparing MI and CS approaches in patients requiring mitral valve surgery. Non-randomised and non-English studies were excluded. The primary outcomes were all cause mortality, operating time, length of hospital and ICU stay, Aortic Cross Clamp Time and cardiopulmonary bypass time. Analysis was conducted using Random Effects Model of meta-analysis using R software.

**Results**

Four studies were included in the final quantitative analysis with a total of 440 patients (Males: 206; Females: 234). Mean follow-up period was 1.31 years. There was no significant differences between MI and MS approach for all-cause mortality [relative risk (RR) 1.23, 95% Confidence Interval (CI) 0.02–70.84; P = 0.63].

**Conclusions**

Our study provides evidence that MI approach for mitral valve surgery is safe. However, larger sample sized RCTs with longer follow-up data are needed to better elucidate efficacy outcomes. We await UK Mini Mitral RCT that aims to serve as a robust, well-powered trial to answer this question.

## Adult Cardiac Scientific & Experimental

### A110 Developing Patient-specific Arterial Disease Models Using Endothelial Colony Forming Cells

#### Thammandra, Vamsi, Mr

##### St George's University of London, London, UK

*Journal of Cardiothoracic Surgery* 2023, **18(Supp 1)**:A110

**Objectives**

Endothelial colony forming cells (ECFC) offer an unparalleled opportunity for understanding endothelial physiology in health and disease. However, whether ECFC phenotype is representative or entirely different to that of venous or arterial endothelium is unknown. Furthermore, the putative progenitor-like nature of ECFCs may provide an opportunity for their instruction towards a specific endothelial sub-type of interest for disease studies e.g. arterial endothelium.

**Methods**

The arterio-venous phenotype of ECFC, arterial EC (HAEC) and venous EC (HUVEC) was assessed using qPCR, western blot and immunofluorescence of several canonical markers. Methods for the induction of an arterial phenotype in ECFCs were explored using recombinant DLL4 (rDLL4) and a protocol developed for arterialisation of induced pluripotent stem cells using arterial differentiation media.

**Results**

Differential gene expression was not observed between HAEC and HUVEC except for the "gold standard" marker for arterialisation, HEY2. ECFCs did express both arterial and venous markers at a protein and mRNA level. Induction of arterial markers in ECFCs using rDLL4 was weak in comparison to HUVEC. Arterial differentiation media resulted in the marked induction of several arterial markers however L690 (IMPase inhibitor) consistently had high expression of all markers except DLL4.

**Conclusion**

We cannot ascertain the phenotypic resemblance of ECFC to either arteries or veins due to the lack of distinction between HAEC and HUVEC. However, ECFCs did express moderate levels of both arterial and venous markers potentially indicating their uncommitted endothelial status. Initial experiments of induction in ECFCs have shown promising results, thus with further optimisation, we propose that ECFCs could be used to form robust in vitro arterial endothelial disease models.

### A111 Single-cell Sequencing to Investigate Metabolic Stress in the Pathology of Organ Injury Following Cardiac Surgery

#### Sheikh, Sophia, Miss; Wozniak, Marcin, Dr; Murphy, Gavin, Prof

##### University of Leicester, Leicester, UK

*Journal of Cardiothoracic Surgery* 2023, **18(Supp 1)**:A111

**Objectives**

Organ injury is a major cause of health complications and in-hospital mortality following cardiac surgery. Our research explores whether patients’ baseline metabolic status is the principal contributor to organ injury and dysfunction following surgery. We hypothesise differential transcriptome and corresponding chromatin accessibility profiles are identified in patients of differing metabolic states through using single-cell sequencing technologies.

**Methods**

We optimised methods for single-cell RNA sequencing (scRNA-seq), single-nuclei RNA sequencing (snRNA-seq) and single-nuclei ATAC sequencing (snATAC-seq) using murine heart tissue and right atrial biopsies from patients recruited to our ongoing Ob-CARD trial (NCT02908009). Chromatin immunoprecipitation (ChIP) qPCR using Ob-CARD leukocytes was optimised to confirm epigenetic heterogeneity between metabolic states relative to acetylated H3 and H4 histone subunits.

**Results**

Results showed different preparation methods of cardiac tissue using mouse and Ob-CARD samples produced a poor representation of cardiomyocytes in scRNA-seq data. Certain nuclei preparation methods provided greater cardiomyocyte representation in snRNA-seq data compared to cells. Ob-CARD patient leukocytes derived from peripheral whole blood collected at pre- and post-operative time points similarly underwent scRNA-seq to investigate transcriptome differences in circulating immune cells. snATAC-seq was successfully performed in mouse heart tissue, requiring validation in Ob-CARD samples.

**Conclusions**snRNA-seq and snATAC sequencing are viable methods for patient cardiac tissue samples to provide insight into cell-type specific differential gene expression and chromatin accessibility profiles of all major cardiac cell types. This will help to delineate differences between patients of different metabolic states and perhaps later inform the development of effective therapeutic strategies.

### A112 The Role of Calcitonin in Prevention and Management of Postoperative Atrial Fibrilation

#### Krasopoulos, George^1^, Professor; Moreira, Lucia^2^, Dr; Sayeed, Rana^3^, Mr; Robinson, Paul^2^, Dr; Mehat, Neelam^2^, Mrs; Reilly, Svetlana^2^, Prof

##### ^1^Oxford Heart Centre, Oxford, UK; ^2^Radcliffe Department of Medicine (Cardiovascular Division), University of Oxford, Oxford, UK; ^3^Cardiothoracic Surgery, Oxford Heart Centre, Oxford, UK, Oxford University Hospitals NHS Foundation Trust, Oxford, UK

*Journal of Cardiothoracic Surgery* 2023, **18(Supp 1)**:A112

**Objectives**

Atrial fibrillation (AF) is the commonest cardiac arrhythmia and a major therapeutic challenge. We recently discovered that atrial cardiomyocytes (CMs) secrete cardiac calcitonin (CT). The direct effects of CT signalling on CMs function, arrhythmogenicity and post-cardiac surgery AF (poAF) are unknown.

**Methods**

An cohort of 38-patients that underwent cardiac surgery had their pre-operative/postoperative circulating CT levels and their incidence of poAF recorded. A further study involving 110-patients has been designed to evaluate the changes in circulating CT and Pro-Calcitonin (PCT) on the new-onset of poAF.

**Results**

The pre-operative levels of CT are associated with a ~ 2.8-fold reduction in the incidence of poAF. Patients with poAF also failed to recover supressed CT levels 3-days after the surgery. In vitro experiments in freshly isolated atrial guinea pig atrial cardiomyocytes have shown that CT exerts its effects via binding to CT-receptors (CTR). Functional studies in animal found that CT administration inhibits spontaneous calcium-release events and calcium transient amplitude induced by pacing in CMs.

**Conclusions**

Our findings suggest that CT potently supresses cell arrhythmogenicity. We are planning to evaluate this further with our new study that is focused on poAF, aiming to translate our findings into direct benefiting the clinical management of AF.

### A113 In-hospital Patient Mobilization Quantification After Cardiac Surgery Using Accelerometers: What do Patients do?

#### Halfwerk, Frank^1^, Dr; Klaassen, Randy^2^, Dr; Lynch, Winston^1^, Mr; van Delden, Robby^2^, Dr; Veltink, Peter^3^, Prof; Grandjean, Jan^1^, Prof

##### ^1^Thoraxcentrum Twente, Medisch Spectrum Twente, Enschede, The Netherlands; ^2^Human Media Interaction Lab, University of Twente, Enschede, The Netherlands; ^3^Dept. of Biomedical Signals and Systems, University of Twente, Enschede, The Netherlands

*Journal of Cardiothoracic Surgery* 2023, **18(Supp 1)**:A113

**Objectives**

After heart surgery patients stay for 4–7 days in the hospital and start their rehabilitation the day after surgery with physiotherapy training. Patients infrequently mobilize during their surgical ward stay, as patients are unaware why mobilization is important. Furthermore, patients’ progress of mobilization activities is not available. The aim of the MOV_E_M_E_NTT study was to use accelerometers with artificial intelligence algorithms for quantification of in-hospital mobilization after cardiac surgery.

**Methods**

Patient activities lying in bed, sitting in a chair, standing, walking, cycling on an exercise bike, and walking the stairs were defined to measure patient mobilization. An accelerometer (AX3, Axivity) was postoperatively placed on both the upper arm and upper leg. An artificial neural network algorithm classified the activities. The primary endpoint was each activity duration performed between 7 a.m. and 11 p.m. Secondary endpoints were intensive care unit and surgical ward stay. A subgroup analysis was performed for male and female patients.

**Results**

29 cardiac surgery patients were classified with an intensive care unit stay of 1 (1–2) night and surgical ward stay of 5 (3–6) nights. Patients spent 41 (20–62) min less time in bed for each following hospital day (p < 0.001). Although patients practiced in the morning, they laid more in bed in the afternoon. Standing (p = 0.004), walking (p < 0.001), and walking the stairs (p = 0.001) increased during hospital stay. No differences between men (n = 22) and women (n = 7) were observed for all endpoints.

**Conclusion**

The approach presented in this study is applicable for measuring all six activities and for monitoring postoperative recovery of cardiac surgery patients. A next step is to provide remote monitoring with wearable sensors to guide patient-specific cardiac rehabilitation.
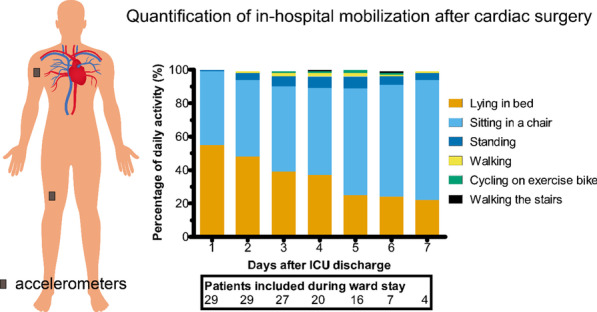


### A114 Innovating a Brain Protection Device for Cardiac Surgery and Cardiac Arrest: A Cool Solution Using Diffusion-Absorption-Refrigeration Technology

#### Slim, Naim, Mr; Salmasi, Mohammad Yousuf, Dr; Harraz, Asma, Ms; Markides, Christos, Prof; Athanasiou, Thanos, Prof; Casula, Roberto, Mr

##### Imperial College London, London, UK

*Journal of Cardiothoracic Surgery* 2023, **18(Supp 1)**:A114

**Objectives**

Cardiac surgery and cardiac arrest are associated with ischaemic cerebral injury secondary to microemboli and global cerebral haemodynamic changes during cardioplegia. Systemic neuroprotective strategies achieve an effective reduction in core temperature but are associated with pneumonia, myocardial dysfunction and coagulopathy. This study aimed to create a computational model of a smart brain cooling device.

**Methods**

We investigated the feasibility of a novel cooling system that induces selective cooling with diffusion-absorption-refrigeration (DAR) technology—a refrigeration method with an array of existing commercial uses. A computational model was developed using the gPROMS platform, whereby the application of a topical coolant at freezing point was simulated on a head and neck model, with adaptation of the cerebral circulation to reflect the haemodynamic changes of cardiac arrest. Core brain temperature was measured against time, and the unit power requirement to provide cooling and running costs were also calculated.

**Results**

During the cardiac arrest simulation, core brain temperature fell by −0.37C without neck cooling and −2.31C with neck cooling. Grey matter temperature fell by −0.71C with neck cooling and −2.63C with neck cooling. The cooling power required to sustain these temperatures was approximately 69.15W at onset and 36.13W to maintain cooling throughout device application, which is achievable with one DAR unit with a cost of £144–180 and ongoing running costs of 5–8p/h.

**Conclusion**

Our study has demonstrated that brain cooling can be achieved with a combination of head and neck cooling using existing commercially-available refrigeration technology. The low power requirement and running cost suggest the feasibility of a portable battery-operated device that can be deployed in hospitals and in the pre-hospital setting.
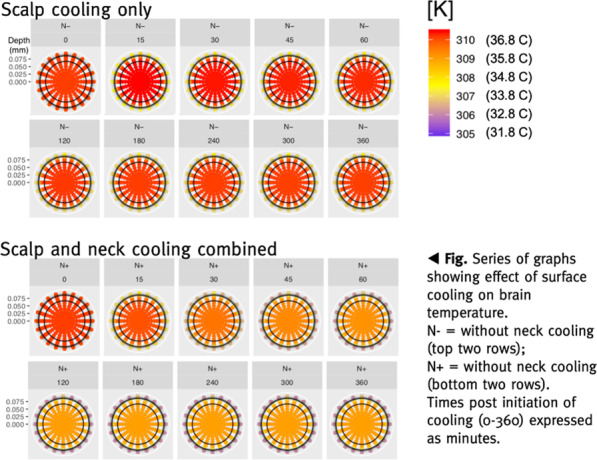


### A115 The Role of Extracellular Matrix in The Regulation of Vascular Smooth Muscle Phenotype ad Function During Vascular Calcification

#### Chan, Shie Wei, Miss

##### Cardiff University, Cardiff, UK

*Journal of Cardiothoracic Surgery* 2023, **18(Supp 1)**:A115

**Objectives**

1. Characterize expression and alterations in HA and related proteins in VSMCs before and after osteoblastic differentiation.

2. Evaluate the effects of cytokines associated with heightened inflammation in PD on: a) VSMC differentiation b) calcium/phosphate generation and c) alterations in HA and related proteins.

3. Investigate the causal relationship between alterations in HA identified in Aim-1 and Aim-2 to VSMC-osteoblast differentiation and calcium/phosphate generation.

**Methods**

Quantitative reverse transcriptase PCR (RT-qPCR) and immunocytochemistry were used to confirm osteogenic differentiation by assessing for osteogenic markers (RUNX2, osteopontin, attenuated alpha-smooth-muscle-actin). Primary human vascular smooth muscle cells were grown in-vitro and osteoblastic differentiation of these cells was promoted by incubating with osteogenic medium (ascorbic acid 2 phosphate, glycerol 2 phosphate, dexamethasone). Alternations in HA and related proteins were investigation using RT-qPCR and immunocytochemistry.

**Results**

RESULTS 1: Differentiation of VSMCs to Osteoblastic Phenotype.

RUNX2 and osteopontin are established markers of VSMC-osteoblast differentiation and of VC. Alpha-SMA is a marker of VSMC.

RESULTS 2: HA expression following VSMC to osteogenic differentiation.

RESULTS 3: Alterations in HAS Synthase expression following VSMC to osteogenic differentiation.

Osteogenic differentiation is associated with marked changes in HAS3 isoenzyme expression.

(Images for results 1,2 and 3).

VSMC to osteogenic differentiation is associated with marked changes in HA expression and in the enzymes/proteins involved in HA synthesis, degradation and binding, suggesting that alteration in HA matrix may play a role in VSMC pathobiology during vascular calcification. Establishing a causal link between these changes and VSMC differentiation during VC may identify novel therapeutic targets for CKD specific cardiovascular disease.

### A116 Investigating Estimated Blood Loss and Haemoglobin Level After Cardiac Surgery; A Potential New Transfusion Trigger?

#### Soliman, Nadine^1^, Miss; Hayes, Timothy^2^, Dr; Rajamiyer, Venkateswaran^2^, Mr; Grant, Stuart W^1^, Mr

##### ^1^University of Manchester, Manchester, UK; ^2^Manchester University NHS Foundation Trust, Manchester, UK

*Journal of Cardiothoracic Surgery* 2023, **18(Supp 1)**:A116

**Objectives**

Currently haemoglobin (Hb) is used as the primary trigger for post-operative blood transfusion following cardiac surgery. Haemodilution secondary to administration of intravenous fluids both intra- and post-operatively can contribute to a fall in Hb irrespective of any blood loss. This study aimed to investigate the relationship between post-operative Hb levels and estimated blood loss after cardiac surgery.

**Methods**

A review of the literature was performed to identify methods to estimate blood loss that only require routinely observed post-cardiac surgery data. Data were collected for patients who underwent adult cardiac surgery over a consecutive six-week period. Estimated blood loss (VL_RBC_) was calculated using the OSTHEO method. VL_RBC_ was compared to percentage change in Hb (g/dL) from baseline to the post-operative nadir Hb. Pearson’s correlation coefficients were computed for Hb and VL_RBC_.

**Results**

A total of 40 patients were included. The majority (n = 27) were male and the mean age was 67.1 (SD ± 9.2). Mean pre-operative Hb was 138.4 g/dL (SD 14.4), the mean post-operative Hb nadir was 93.3 g/dL (SD 12.9). The mean percentage fall in Hb from pre-op to nadir was 32.3% (SD 8.4), which was significantly greater than the mean percentage VL_RBC_ of 12.6% (SD 4.0), p < 0.001. Percentage fall in Hb correlated well with percentage VL_RBC_ (R2 = 0.824). A total of 4 patients had a fall in Hb below 80 g/dL, in these patients the mean fall in Hb from baseline was 38.5% (SD 9.5), and the mean VL_RBC_ was 15.3% (SD 4.2).

**Conclusions**

VL_RBC_ calculated using the OSTHEO method correlates well with post-operative changes in Hb after cardiac surgery. Estimated blood loss is consistently less than the fall in Hb. This method can be calculated using easily available data that accounts for changes in circulating blood volume and patient weight. Further work is required to explore whether VL_RBC_ could replace Hb as a transfusion trigger after cardiac surgery.
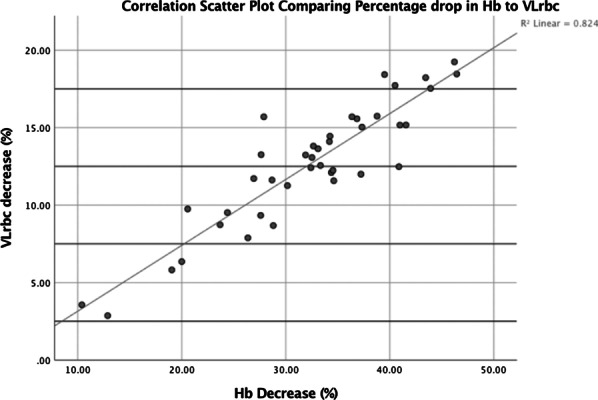


### A117 How to Test Adhesive Strength: A Novel Biomechanical Testing for Aortic Glue Used in Type A Dissection Repair

#### Zientara, Alicja^1^, Ms; Tseng, Yuan-Tsan^2^, Dr; Salmasi, Mohammad Yousuf^1^, Dr; Quarto, Cesare^1^, Mr; Stock, Ulrich^3^, Prof

##### ^1^Royal Brompton Hospital, London, UK; ^2^Imperial College & Magdi Yacoub Institute, Harefield, UK; ^3^Harefield Hospital, Harefield, UK

*Journal of Cardiothoracic Surgery* 2023, **18(Supp 1)**:A117


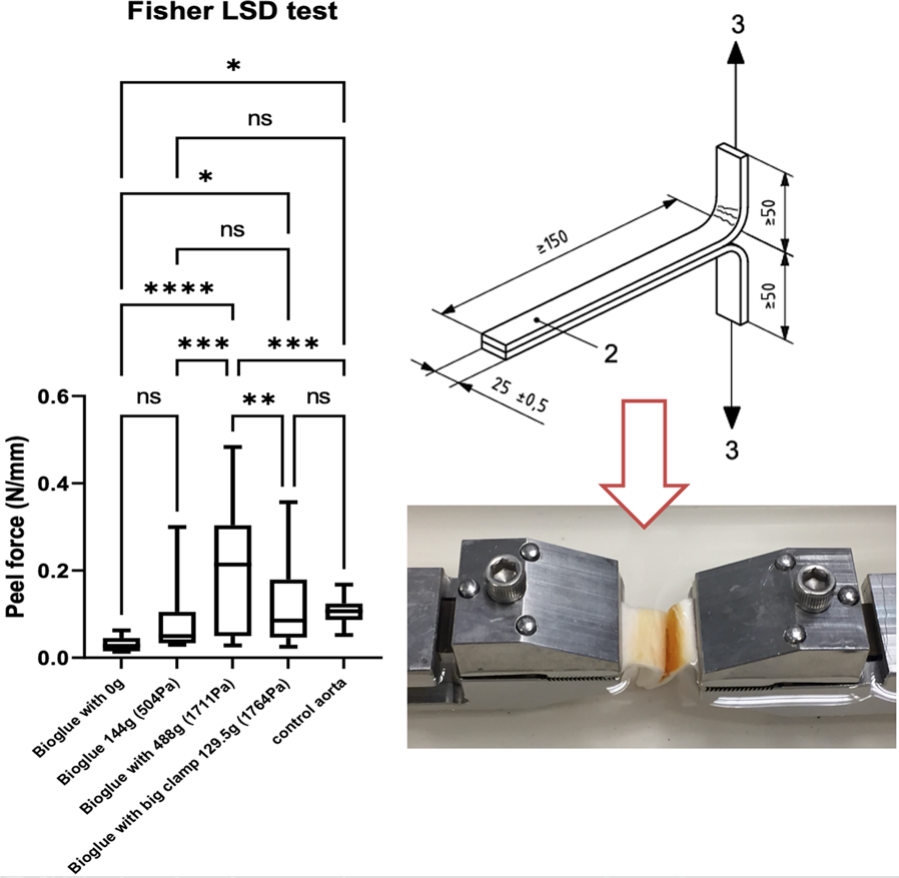


**Objective**

The widely used Bioglue (Cryolife®) represents the gold standard component in the repair of type A dissections. Despite broad acceptance the adhesive strength of the glued aortic layers has not been tested and quantified so far. The aim was to demonstrate the preliminary results of the peel force of a standard tissue glue in an in vitro model simulating a type A dissection.

**Methods**

This study is based on the adaptation of the adhesive T-peel test (EN ISO 11339:2010) for the determination of the peel strength of adhesives by measuring the peeling force of a T-shaped bonded assembly of two flexible tissues. Measurements were performed on juvenile ascending porcine aorta using a Bose Electro Force Planar Biaxial Test Bench Instrument®. Aortic samples, glued with Bioglue, were tested and compared to normal unpeeled controls. Four conditions of different sample pressure were tested: zero pressure according to the manufacturer’s recommendation (n = 7), slight pressure (504 Pa) (n = 11), moderate pressure (1711 Pa) (n = 7) and pressure applied by a Borst clamp with a force of 1764 Pa (n = 19). T-test was applied for statistical significance.

**Results**

The median peel force (± SD) with zero pressure was 0.027 N/mm (± 0.018), with slight pressure 0.05 N/mm (± 0.086), and with moderate pressure 0.214 N/mm (± 0.157). The samples using the Borst clamp reached 0.085 N/mm (± 0.096). The unpeeled controls reached a force of 0.11 N/mm (± 0.031). Bioglue with moderate pressure performed better than samples without (p < 0.0001) and with slight pressure (p = 0.0005) and had also a higher force than the unpeeled controls (p = 0.0008).

**Conclusion**

The performance of Bioglue in a model for aortic dissections demonstrated increased peel force after applying a moderate pressure on the aortic sample in contrast to slight or no pressure as per the manufacturer’s recommendation. The novel T-peel test offers an attractive method to test tissue glues in general in a defined in vitro environment.

### A118 Utilisation of National Early Warning Score (NEWS) and Assessment of Patient Outcomes Following Cardiac Surgery

#### Jacob, Abiah S, Ms; Kumar, Niraj S, Mr; Trevarthen, Thomas, Mr; Awad, Wael I, Mr

##### Barts Heart Centre, London, UK, St. Bartholomew's Hospital, London, UK

*Journal of Cardiothoracic Surgery* 2023, **18(Supp 1)**:A118

**Objectives**

NEWS was introduced to standardise triaging of patients with clinical deterioration to reduce mortality, with NEWS > 5 initiating Critical Care Outreach Team (CCOT) engagement. We investigated involvement of CCOT based on NEWS following cardiac surgery and subsequent patient outcomes.

**Methods**

Patients undergoing cardiac surgery between October 2020 and October 2021 were studied. Patient characteristics, clinical and physiological causes triggering CCOT review were probed. Outcomes following CCOT review were evaluated.

**Results**

72 of 1588 (4.53%) patients (mean age 64.9 ± 2.5 years, EuroSCORE 7.13) initiated 91 calls to CCOT following surgery. Mean NEWS score on CCOT activation was 5.78 (95% CI: 5.29–6.26), with 21/91 (23.1%) activations from patients with NEWS < 5. The most common NEWS parameters contributing to activations were oxygen therapy (mean: 1.76) and systolic blood pressure (mean: 1.20). CCOT activations led to 12 transfers to ITU and 18 to HDU; 4/72 (5.56%) patients suffered cardiac arrest; 4/72 (5.56%) had emergency resternotomy; mean length of post-operative hospital stay was 18.3 ± 3 days; in-hospital mortality was 6.94% (5/72 patients).

**Conclusion**

NEWS is a useful way of initiating CCOT involvement for patients with acute clinical deterioration. The early involvement of CCOT and standardised recommendations on patient management thereafter, may lead to improved patient outcomes.

### A119 snoRNAs: A Genetic Marker to Inform the Choice of Conduit for Coronary Artery Bypass Grafting (CABG) and Potential Mechanism of Action

#### Kumar, Ujjawal, Mr; Hamilton, Russell, Dr

##### Department of Genetics, University of Cambridge, Cambridge, UK

*Journal of Cardiothoracic Surgery* 2023, **18(Supp 1)**:A119

**Objectives**snoRNAs at the 14q32 genetic locus are associated with failed venous grafts after CABG (Håkansson et al., 2019). It is hypothesised that the snoRNAs show structural similarity to the spliceosome, a cellular assembly involved in pre-mRNA transcript splicing. We aimed to investigate the similarity of snoRNAs to the spliceosome and propose a mechanism for their role in cardiovascular disease.

**Methods**snoRNA and spliceosomal RNA sequences from the human reference genome (GRCh38) were obtained from Rfam, an online database of non-coding RNAs. 2D and 3D structural modelling of the snoRNAs were undertaken using ViennaRNA and SimRNA respectively. Utilising multiple computational tools, sequential (1D) and structural (2D and 3D) similarities between individual snoRNAs and the spliceosome were investigated in order to identify candidate snoRNAs for in-depth pairwise comparison.

**Results**

We identified that a vast majority of the forty snoRNAs (in the 14q32 locus) showed some structural similarity to the spliceosome. 3D structural prediction was undertaken for snoRNAs that showed the greatest 1D and 2D similarity, identifying specific snoRNAs with high degrees of structural similarity to the catalytically active site of the spliceosome.

**Conclusions**

We, therefore, propose that these snoRNAs mimic the spliceosomal structure and interfere with spliceosomal function. Variation in the snoRNAs could lead to mis-splicing of pre-mRNA and subsequent pathological tissue remodelling. Remodelling is a key part of tissue response in the venous grafts after CABG. The mis-splicing due to variation in these snoRNAs could explain the significantly raised graft failure rates. These snoRNAs are not associated with high graft failure rates with arterial conduits for CABG. Thus, genetic screening for these snoRNAs could inform the choice of conduit for CABG, improving patient outcomes by reducing likelihood of graft failure and need for reintervention.

### A120 Variation in Cellular Regulation with Patient Weight Categories within Atrial Tissue

#### Adebayo, Adewale^1^; Eagle-Hemming, Bryony^1^, Ms; Lai, Florence^1^, Mrs; Joel-David, Lathishia^1^, Mrs; Murphy, Gavin^2^, Prof; Wozniak, Marcin^1^, Dr

##### ^1^University of Leicester Glenfield Hospital, Leicester, UK; ^2^Glenfield Hospital, Leicester, UK

*Journal of Cardiothoracic Surgery* 2023, **18(Supp 1)**:A120

**Objectives**

We examined the hypothesis that cellular regulation dysfunction underlies observed paradox in which higher body mass index may be beneficial for categories of surgery patients.

**Methods**

Adult cardiac surgery patients were recruited and demographic data obtained. Atrial biopsies were collected during cardiopulmonary bypass. Transcriptome data was acquired for samples from 53 patients, and a panel of targeted metabolites was analysed in samples from 57 patients.

**Results**

Sixteen patients had a BMI below 25, 31 had a BMI between 25 – 30 and 19 above 30. Weight groups significantly differed in age and haematocrit levels. Normal-weight patients were more diverse in their metabolite expression, while overweight and obese patients appeared more homogeneous. Statistical analysis identified more differentially expressed (DE) biological pathways in overweight vs normal-weight comparison as well as obese vs normal-weight. It also identified genes involved in regulation of translation, lipids and muscle contraction were expressed in a biphasic pattern, which potentially mimics the obesity paradox. Metabolite analysis identified 9 compounds with differing levels in obese, normal-weight and overweight groups including specific carnitines and pentose-pathway metabolites.

**Conclusions**

The results support our hypothesis of multi-omic changes in myocardium. Specific links with transcripts, biological mechanisms and direct clinical impact require further investigation.

## Congenital

### A121 Outcomes from Ross Procedure in Adults with Previous Aortic Valve Intervention

#### Visan, Alex, Dr; McPherson, Iain, Mr; Generali, Tommaso, Mr; De Rita, Fabrizio, Mr; Jansen, Katrijn, Dr; Coats, Louise, Dr; Hasan, Asif, Mr; Nassar, Mohamed, Mr

##### Freeman Hospital, Newcastle, UK

*Journal of Cardiothoracic Surgery* 2023, **18(Supp 1)**:A121

**Objectives**

Modified Ross procedure continues to deliver excellent outcomes for aortic valve disease in the young adult population. Our objective was to assess if a stepwise approach with repair and replacement strategies prior to Ross protects autograft longevity and function.

**Methods**

Single tertiary, congenital centre with 158 patients (age 16–60) undergoing Ross procedure from 1997–2019. Patients divided into primary Ross (no previous aortic valve intervention) and secondary Ross groups, with coarsened exact matching used to ensure balanced distribution of age, sex, weight and valve pathology. Primary outcome was autograft failure (a composite of time from Ross to diagnosis of severe aortic regurgitation, redo aortic valve repair or replacement). Secondary outcomes included survival, autograft reoperation rate, and presence of dilated autograft (> 40 mm). Time to event outcomes analysed using the Kaplan–Meier method and compared using log-rank testing. Univariate and multivariate Cox-proportional hazard models were used to identify time-dependent predictors of autograft failure.

**Results**

103 (65.2%) patients underwent primary Ross and 55 (34.8%) underwent secondary Ross. After matching, the secondary Ross group showed superior freedom from autograft failure (p = 0.039). 20-year survival was 96.5% (92.8–100) and 20-year freedom from re-operation was 53.5% (36.6–78.2). Male sex was associated with increased risk of neo-aortic root dilatation (OR 4.05, p = 0.02). Newer operative techniques (after 2011) were associated with lower risks of neo-aortic root dilatation (OR 0.20, p = 0.002).

**Conclusion**

In patients who have undergone previous aortic valve interventions, Ross procedure has a lower risk per year follow-up of pulmonary autograft failure compared with primary Ross. Our results appear to justify a stepwise approach to aortic valve disease, with conservative strategies for the treatment of aortic valve disease adopted in the first instance.
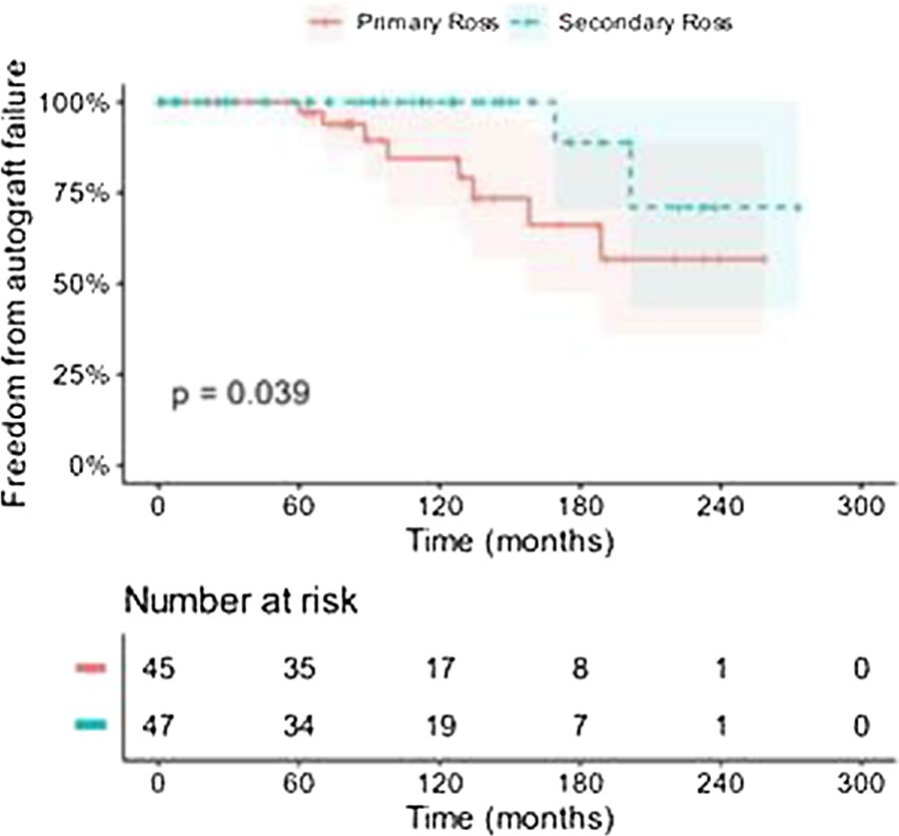


### A122 Necrotising Enterocolitis Pre Cardiac Surgery. Damned if you, Damned if you don't

#### Boyle, Mark^1^, Dr; Khodaghalian, Bernadette^2^, Dr; Jones, Caroline^2^, Dr; Guerrero, Rafael^2^, Mr

##### ^1^St Thomas's Hospital, London, UK; ^2^Alder Hey Children's Hospital, Liverpool, UK

*Journal of Cardiothoracic Surgery* 2023, **18(Supp 1)**:A122

**Objectives**

Infants with congenital heart disease (CHD) have the potential to develop Necrotising Enterocolitis (NEC) with devastating consequence. However, simply a suspicion of NEC can delay cardiac surgical intervention causing increase in mortality and morbidity in patients awaiting a "time critical" procedure. This highlights the necessity of accurate diagnosis. We assessed the incidence of NEC in infants with CHD awaiting surgery and the subsequent impact on outcomes at our centre.

**Methods**

This retrospective cohort study over a 24-month period utilised data obtained from NICOR and local surgical databases. We included infants < 90 days of age with a diagnosis of CHD and a diagnosis of NEC prior to cardiac surgery requiring bypass. We collected data on demographics, cardiac lesion, feeding patterns, biochemical markers, diagnostic imaging, clinical assessment and patient outcomes.

**Results**

24 patients were diagnosed with NEC prior to a cardiac operation involving bypass in this period, 38% of whom were born prematurely. Within this cohort, 38% of patients had transposition of the great arteries, 21% arch lesions and 17% pulmonary atresia’s with lesser incidence of other lesions. Feeding was mixed and 71% were on prostaglandin at time of diagnosis. Clinical signs and biomarkers were varied. 7 patients had an abnormal x-ray, 4 patients ultrasound changes (3 of which had normal abdominal films). 7 cardiac operations were definitively delayed and 4 patients underwent surgical management for NEC. Mean ICU stay post cardiac surgery was 11 days with 0% mortality at 30 days.

**Conclusions**

The question remains are the typical diagnostic pathways appropriate for our population? Certainly, in our centre the utilisation of ultrasound is under review in light of these findings, as is a review of the feeding recommendations in all our cardiac infants. Is it time to look at new ways of diagnosing these infants, incorporating risk stratification in our usual practice?

### A123 Surgical Mitral Valve Replacement with Melody Valve in Paediatric Patients: Single Centre Experience

#### ElSherbini, Ahmed, Mr; Salih, Caner, Mr; Austin, Conal B, Mr; Jones, Matthew I, Mr; Kabir, Saleha, Mrs; Speggiorin, Simone, Mr

##### Evelina London Children's Hospital, London, UK, Guy's and St Thomas' NHS Foundation Trust, London, UK

*Journal of Cardiothoracic Surgery* 2023, **18(Supp 1)**:A123

**Objectives**

To demonstrate the efficacy of a modified stented bovine jugular vein graft (Melody valve) for surgical mitral valve replacement in paediatric patients.

**Methods**

A single centre retrospective study of patients who underwent mitral valve replacement using a modified Melody valve during the period from 2016 to 2021.

**Results**

A total of nine patients with a median age of 2.03 years (range, 3 months to 4.69 years) underwent surgical implantation of a Melody valve in the mitral position. Seven patients had previously undergone cardiac surgical procedures. Three patients had the valve implanted on an 18 mm balloon, three at 16 mm and one each at 12 mm, 14 mm and 20 mm A Ross-Konno operation was undertaken at the same in two patients. At discharge, all valves were competent with low mitral valve inflow gradients (median 5 mmHg). Median duration of intensive care unit stay after procedure was 6 days (range, 1 to 14 days). One patient, who had also undergone a Ross-Konno operation developed cardiogenic shock and required extracorporeal membrane oxygenation support for 10 days. Valve redilatation was performed in two patients for somatic growth around one year after implantation and transcatheter valve replacement for acute valve failure was undertaking in one patient around 5 years after implantation. Endocarditis occurred in 1 requiring explantation and replacement with mechanical prosthesis. At a median follow up of 1.6 years, seven patients are free from structured valve deterioration. No mortality has been reported to date.

**Conclusion**

Surgical implantation of a Melody valve in the mitral position provides a safe and durable solution for valve replacement in paediatric patients with small mitral annulus dimensions. A large prospective study is recommended and further refine of valve design are needed.

### A124 The Bilateral Remote Ischaemic Conditioning in Children (BRICC) Trial: A Two-centre, Double-blind, Randomised Controlled Trial in Young Children

#### Drury, Nigel^1^, Mr; van Doorn, Carin^2^, Ms; Woolley, Rebecca^3^, Ms; Amos-Hirst, Rebecca^3^, Ms; Jaber, Osama^2^, Mr; Kassai, Imre^2^, Mr; Pelella, Giuseppe^2^, Mr; Khan, Natasha^1^, Ms; Botha, Phil^1^, Mr; Jones, Timothy^1^, Mr

##### ^1^Birmingham Children's Hospital, Birmingham, UK; ^2^Leeds Children's Hospital; ^3^University of Birmingham, Birmingham, UK

*Journal of Cardiothoracic Surgery* 2023, **18(Supp 1)**:A124

**Objectives**

To determine whether adequately delivered bilateral remote ischaemic preconditioning (RIPC) is cardioprotective in young children with or without chronic cyanosis undergoing elective cardiac surgery.

**Methods**

Two-centre, prospective, double-blind, randomised controlled trial of children aged 3 months to 3 years undergoing complete repair of tetralogy of Fallot (ToF) or surgical closure of an isolated ventricular septal defect (VSD). Participants were randomised to receive either: bilateral RIPC (3 × 5-min cycles) using a pressure-controlled tourniquet; or sham, delivered immediately prior to surgery, with follow-up until hospital discharge up to 30 days. The primary outcome was area under the curve (AUC) for hs-troponin-T release in the first 24 h after reperfusion. Secondary outcomes included vasoactive inotrope score, arterial lactate, and lengths of stay in the ICU and hospital.

**Results**

Over 4 years, 121 children were randomised, 61 children allocated to RIPC and 60 to sham; one child in the RIPC group did not proceed to surgery so was excluded from analysis. Mean AUC hs-troponin-T was higher in the RIPC group, mean: 70.0 µg/L/hr, SD: 50.9, n = 56 versus sham, mean: 55.6, SD: 30.1, n = 58 (Mean diff: 13.2; 95% CI: 0.5–25.8; p = 0.04) (figure). Sub-group analyses did not show a differential treatment effect in cyanotic and acyanotic children (interaction p-value = 0.2); however, there may be evidence of a difference by congenital heart defect, though numbers were small (interaction p-value = 0.04): unstented ToF, mean diff: 30.9, 95% CI: 12.2–49.6; stented ToF, mean diff: 7.8, 95% CI: -27.7–43.4; VSD, mean diff: -3.2, 95% CI: -22.0–15.5. There were no differences in any secondary outcome measures.

**Conclusions**

In young children undergoing elective cardiac surgery, we found children randomised to RIPC had greater hs-troponin-T release in the early postoperative period, which may reflect increased myocardial injury, especially in ToF.
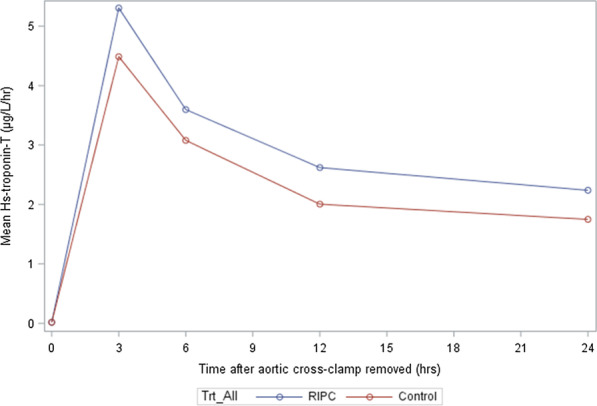


### A125 Are Chest Radiographs Necessary After Chest Drain Removal in Paediatric Cardiac Surgical Patients? – A Retrospective Analysis of 1076 Patients

#### Shetty, Gautham^1^, Dr; Zouki, Jason^2^, Mr; Lee, Geraldine^2^, Miss; Betts, Kim^3^, Mr; Justo, Robert^1^, Dr; Marathe, Supreet^1^, Dr; Alphonso, Nelson^1^, Dr; Venugopal, Prem^1^, Dr

##### ^1^Queensland Children’s Hospital, Brisbane, Australia; ^2^Queensland University, Queensland, Australia; ^3^Curtin University, Bentley, WA, Australia

*Journal of Cardiothoracic Surgery* 2023, **18(Supp 1)**:A125

**Objectives**

Chest drains are routinely placed in children following cardiac surgery. Studies have questioned the need for routine chest radiograph after chest drain removal in terms of re-interventions, radiation exposure and cost-effectiveness. The purpose of this study was to determine whether a chest radiograph can be avoided following chest drain removal.

**Methods**

A single-centre retrospective cohort study. Inclusion criteria were patients between 0 days and 18 years of age who underwent cardiac surgery between 1/1/2015 and 31/12/2019 with insertion of mediastinal and/or pleural drains. Exclusion criteria were chest drain/s in situ ≥ 14 days and mortality prior to removal of chest drain/s.

**Results**

1076 patients {median age: 292 days (IQR 62, 1956); median weight: 7.8 kg (IQR 4.1, 18.5)}; 1587 drain removal episodes—2365 drains [1347 (57%) mediastinal drains, 598 (25%) right pleural drains and 420 (18%) left pleural drains]. Chest radiographs were performed following 1301 (82%) drain removal episodes. There was no mortality related to chest drain removal. Chest radiograph was abnormal after 152 (12%) drain removal episodes [pneumothorax (n = 43, 3%), pleural effusion (n = 98, 8%) and hydropneumothorax (n = 11, 1%)]. Of these patients, clinical signs or symptoms were absent in 122 (n = 152, 80%) patients and present in 30 (n = 152, 20%). 14 (n = 152, 8%) required a change in management. 11 (n = 152, 7%) required medical management (non-invasive respiratory support or diuresis); 1 required reintubation and ventilation; 2 required chest drain reinsertion.

**Conclusion**

The incidence of clinically significant pneumothorax/pleural effusion following chest drain removal after paediatric cardiac surgery is low (0.02%). The majority of these patients were managed medically and did not require chest drain reinsertion. We conclude that not all paediatric cardiac surgical patients require chest radiographs following chest drain removal.

### A126 CardioCel for Repair of Congenital Heart Defects – Multicentre Results of Over 1000 Implants

#### Daley, Michael^1^, Dr; Marathe, Supreet^1^, Dr; Gamal, Mohamed^2^, Dr; Betts, Kim^1^, Mr; Andrews, David^3^, Dr; Brizard, Christian^4^, Prof; Venugopal, Prem^1^, Dr; Alphonso, Nelson^1^, Dr

##### ^1^Queensland Children's Hospital, Brisbane, Australia; ^2^Queensland Health, Queensland, Australia; ^3^Perth Children's Hospital, Perth, Australia; ^4^Royal Children's Hospital, Melbourne, Australia

*Journal of Cardiothoracic Surgery* 2023, **18(Supp 1)**:A126

**Objectives**

CardioCel is decellularized tissue-engineered bovine pericardium that has undergone the ADAPT**®** process to remove nucleic acid remnants, to reduce in-vivo calcification. Although there has been increasing use of CardioCel in the repair of congenital heart defects over the last decade, long-term outcomes remain to be defined.

**Method**

A multicentre review of 754 patients who underwent repair of congenital heart defects using 1047 CardioCel implants was performed. Data were collected from hospital, operation and outpatient reports. The primary endpoint was CardioCel-related surgical or catheter reintervention.

**Results**

Median age at implantation was 12 months (IQR: 3.6–84 months). Multiple patches were implanted in 214 patients (28.4%). Patches were used for PA augmentation (n = 284, 27.1%), septal defects (n = 266, 25.4%), aortic root/ascending aorta/arch repair (n = 186, 17.8%), valve repair (n = 157, 15.0%), and other (n = 154, 14.7%). Median follow-up was 20 months (0–116 months). One patient died from a CardioCel-related complication (dehiscence of RVOT patch). Freedom from CardioCel-related reintervention was 95% (95%CI: 93%-97%) and 91% (95%CI: 87%-94%) at 1 and 5 years, respectively. Thirty-six CardioCel-related reinterventions were performed, including 13 on the PAs and 9 on the aorta. Cox regression showed that neonates and infants were more likely to require reintervention than children > 1 year of age (HR = 3.96, p = 0.003; HR = 2.34, p = 0.036, respectively). Patients with implants in the aorta and PAs were more likely to undergo reintervention compared to those with septal implants (HR = 3.07, p = 0.034; HR = 2.86, p = 0.033, respectively).

**Conclusion**

CardioCel provides acceptable results for the repair of a variety of congenital heart defects. Neonates and infants required more CardioCel-related reinterventions compared to children > 1 year of age. Patients with aortic and PA implants required more CardioCel-related reinterventions.

### A127 Outcomes After Mechanical Mitral Valve Replacement in Young Congenital Group: A Single Centre 10 Years Results

#### Abousteit, Ahmed^1^, Mr; Harky, Amer^2^, Mr; Bhag, Garima^1^, Dr; Samaddar, Avisheck^1^, Dr; Kutty, Ramesh^1^, Mr; Lotto, Atillio^1^, Mr; Guerrero, Rafael^1^, Mr; Dhannapuneni, Ram^1^, Mr

##### ^1^Alder Hey Children Hospital, Liverpool, UK; ^2^Liverpool Heart and Chest Hospital, Liverpool, UK

*Journal of Cardiothoracic Surgery* 2023, **18(Supp 1)**:A127

**Objectives**

Mitral valve repair and reconstruction are favoured in congenital group especially in very young subgroups to avoid complications related to mechanical mitral valve replacement (MVR), however valve replacement with a mechanical prosthesis is still a necessity in some patients. We report our experience in this cohort of patients.

**Methods**

A retrospective single-centre study for patients who underwent MVR only with a mechanical prosthesis between 2010 and 2020. Data are presented as median, range and percentage.

**Results**

Our cohort consisted of 31 patients. Median age 4.24 years (76 days- 18.8 years), < 2 years n = 11 (35.48%). Pre-operative AVSD diagnosis n = 10 (32.25%), Shone Complex or LVOTO n = 9 (29%). Previous mitral valve repair n = 16 (51.6%) and replacements n = 6 (19.6%). Median interval from attempted repair to replacement 8.13 years (12 days -15 years). Bi-leaflet mechanical mitral valves n = 30 (96.77%) and one bi-leaflet mechanical aortic valve was used in mitral position n = 1 (3.23%). Valve prosthesis sizes ≤ 19 mm n = 10 (32.25%).

Post-operative prolonged mechanical ventilatory support > 7 days n = 6 (19.35%). Post-operative renal failure requiring dialysis n = 3 (9.97%). Septicaemia n = 2 (6.45%). Re-operation for valve thrombosis n = 2 (6.45%). ECMO required n = 3 (9.97%). Two patients died within 30 days (6.45%) (8 and 30 days). Two further in-hospital mortalities > 30 days (39 and 156 days). Mortalities age median 16.2 month (9.7- 35.7 months). Median follow-up was 2.8 years (2.5 months—9.7 years) and with no late reoperation or mortalities recorded. Ten years survival was 87.1%.

**Conclusion**

MVR with a mechanical valve is still a safe option with challenging mitral valve pathologies which are not suitable for a good repair or after failed repair attempts. Higher risk of postoperative mortality and complications are encountered in children less than 2 years old with small valve sizes.

### A128 Repair of Obstructed Supracardiac TAPVD and Rare Compression of Left Main Bronchus Between Pulmonary Venous Confluence and Aorta

#### Bader, Vivian, Miss; Noonan, Patrick, Dr; Peng, Edward, Mr

##### Royal Hospital for Children Glasgow, Glasgow, UK

*Journal of Cardiothoracic Surgery* 2023, **18(Supp 1)**:A128

A 2.5-month old baby, who presented with respiratory distress and poor feeding, Echo showed supracardiac TAPVD with obstruction. Patient needed intubation and his blood pressure was marginal. CXR showed bilateral lung congestion with left lung collapse.

The Repair was achieved via primary sutureless technique, the vertical vein was not easily identifiable from the confluence hence this was initially left alone. First two attempts to come off bypass were not sustainable with low BP and distended RV despite on iNO and high inotropes. Epicardial echo showed laminar flow at PV confluence with no sign of obstruction of flow from both left and right PV branches. There was trivial TR. LV was very under-filled with compression from severely dilated RV.

We elected to explore the vertical vein at the level of innominate vein, which was found to be engorged as well as the vertical vein. The vertical vein was tied off. The third attempt of coming off bypass was associated with good LV ejection. Patient returned to ICU with good haemodynamics and intropes were weaned to minimal. He failed extubation due to right-sided pneumothorax. He later developed left-sided lung collapse and elevated diaphragm. Bronchoscopy showed pulsatile left main bronchus compression. USS showed appropriate diaphragm motion with reduced excursion but fluoroscopy confirmed paradoxical motion. Preop CT showed slit-like compression of left main bronchus between PV confluence and descending aorta. Following plication of hemidiaphragm, and posterior aortopexy under bronchoscopy guidance, he was extubated 2 days later and discharged home after 5 days.

**Conclusion**

The need of vertical vein ligation remains debatable, and in this case, this was the mechanism of failure to come off bypass. Exploration of vertical vein risks phrenic nerve injury. Compression of left main bronchus from PV confluence is very rare and in this case, the only surgical solution is a posterior aortopexy.

### A129 Outcome of Patients Following Presentation with Tetralogy of Fallot, Pulmonary Atresia with Ductal Dependent, Confluent Pulmonary Arteries

#### Kesieme, Emeka^1^, Mr; Danton, Mark^2^, Prof; Bader, Vivian^2^, Miss; McLean, Andrew^2^, Mr; Knight, Brodie^2^, Dr; Smith, Ben^2^, Dr; Noonan, Patrick^2^, Dr; Peng, Edward^2^, Mr

##### ^1^Golden Jubliee National Hospital, Glasgow, UK; ^2^Royal Hospital for Sick Children, Glasgow, UK

*Journal of Cardiothoracic Surgery* 2023, **18(Supp 1)**:A129

**Objective**

To evaluate our outcome of management in patients with pulmonary atresia, ventricular septal defect (PA-VSD) with ductal dependent, confluent pulmonary arteries.

**Methods**

A total of 66 patients, who presented with PA-VSD between 1997–2021, met the following inclusion criteria: confluent branch PA, ductal dependency, no major aorto-pulmonary collaterals that required unifocalisation. All patients were reviewed from the point of first presentation to last follow-up. Late survival was estimated from Kaplan–Meier curve.

**Results**

55 patients had palliative procedure (45 shunt, 8 PDA stent, 2 RF valvotomy vs 7,11% early primary repair following IV Prostin, 4,6% died before any procedure). Overall interstage mortality was 6(11%) (13.3% post-shunt vs 0% post-catheter; p = 0.6), with no difference between eras (15%, 4/27 after year 2005 vs 7%,2/28 prior; p = 0.4). 49%(27) post-palliated patients required reintervention; one-third had > 2 reinterventions: shunt reintervention (13/45), additional shunt (14/45), PDA stent (shunt-2, dilatation-1). 3 had concomitant arterioplasties during shunt, 7 required branch PA reintervention (6 post-shunt, 1 PDA stent) with a total of 13 reinterventions (11-catheter, 2-surgical). The median duration between palliation and primary repair was 15.6 months (range 2.8–72.3). In-hospital mortality rate after complete repair was 3.6% (2 previous palliation, 0 in primary repair group). No in-hospital mortality occurred post-repair after year 2005 (0/49 vs 2/6, 33% prior; p = 0.01). 33(60%) of patients had a total of 58 re-interventions post-repair, most commonly for branch PA(23,40%) and conduit (18, 31%). The overall survival rates for all patients at 10 and 20 years were 78% after being born with the diagnosis.

**Conclusion**

Interstage reintervention and mortality remained significant. With low in-hospital mortality post-repair in the current era, the role of early corrective surgery should be considered.

### A130 Outcome After Neonatal Bilateral Pulmonary Artery Banding and Ductal Stenting as Initial Palliation to Balance Pulmonary and Systemic Circulations

#### Abba, Paola^1^, Miss; Jaber, Osama^2^, Mr; Friedrich, Orsolya^2^, Dr; Bentham, Jamie^2^, Dr; Valesco-Sanchez, Daniel^2^, Dr; Pelella, Giuseppe^2^, Mr; Kassai, Imre^2^, Mr; van Doorn, Carin^2^, Miss

##### ^1^University of Turin, Turin, Italy; ^2^Leeds General Infirmary, Leeds, UK, Leeds Teaching Hospitals NHS Trust, Leeds, UK

*Journal of Cardiothoracic Surgery* 2023, **18(Supp 1)**:A130

**Objectives**

The Hybird Norwood (HN) procedure involves bilateral pulmonary artery banding and stenting of the arterial duct with the aim to balance the pulmonary and systemic circulations. Its main use is in high-risk newborns with a duct dependent systemic circulation and single ventricle, as a temporising measure prior to Norwood 1 (N1) type repairs on cardiopulmonary bypass. To a lesser extend it is used in high-risk newborns with biventricular circulations. Decision to proceed to HN is also influenced by parental insistence for active treatment. We reviewed our results in this challenging group of patients.

**Methods**

Single centre retrospecive study of all consecutive patients that underwent HN between January 2013 and October 2021.

**Results**

There were 15 patients, nine with single ventricle, and most underwent treatment in recent years. Median weight 2.5 (range 1.6–3.6) kg and median age 12 (3–27) days. Patient characteristics are in the Table. Three Patients, including two with single ventricle, died after HNN (at 0, 15 and 61 days, respectively). All deaths were in very small weight patients. Two patients progressed to N1 during the same admission, and 10 were discharged home. There was one interstage death at six weeks during anaesthesia for cross-sectional imaging in a single ventricle patient. Of the six single ventricle patients that survived HN, all successfully completed N1. Of these, one is now interstage, four have completed a Glenn shunt, and one has definitive pallation with a Sano Shunt. Of the five surviving HN patients with biventricular circulation, one died five months after a Ross-Konno procedure and the remaining four had successful biventricular repair.

**Conclusions**

HN gives reasonable short and intermediate survival in this group of high-risk patients. Very low body weight appears a significant risk factor for adverse outcome.

### A131 VATS Lobectomy for 14-Month Baby

#### Wang, Lu, Ms; De Rita, Fabrizio, Mr; Pagliarulo, Vincenzo, Mr

##### Freeman Hospital, Newcastle, UK

*Journal of Cardiothoracic Surgery* 2023, **18(Supp 1)**:A131

https://www.youtube.com/watch?v=0fV7vKnEC_A

### A132 Robotic Reconstruction of Agenesis of the Left Hemi-diaphragm in a Patient with Trisomy 21

#### Kouritas, Vasileios, Mr; Hogan, John, Mr; Saad, Haisam, Mr; Alqudah, Obada, Dr; Szafron, Bartlomiej, Mr; Francis, Jonathon, Dr; Fuentes-Warr, Joana, Mrs; Kadlec, Jakub, Mr; Bartosik, Waldemar, Mr

##### Norfolk and Norwich University Hospital, Norwich, UK

*Journal of Cardiothoracic Surgery* 2023, **18(Supp 1)**:A132


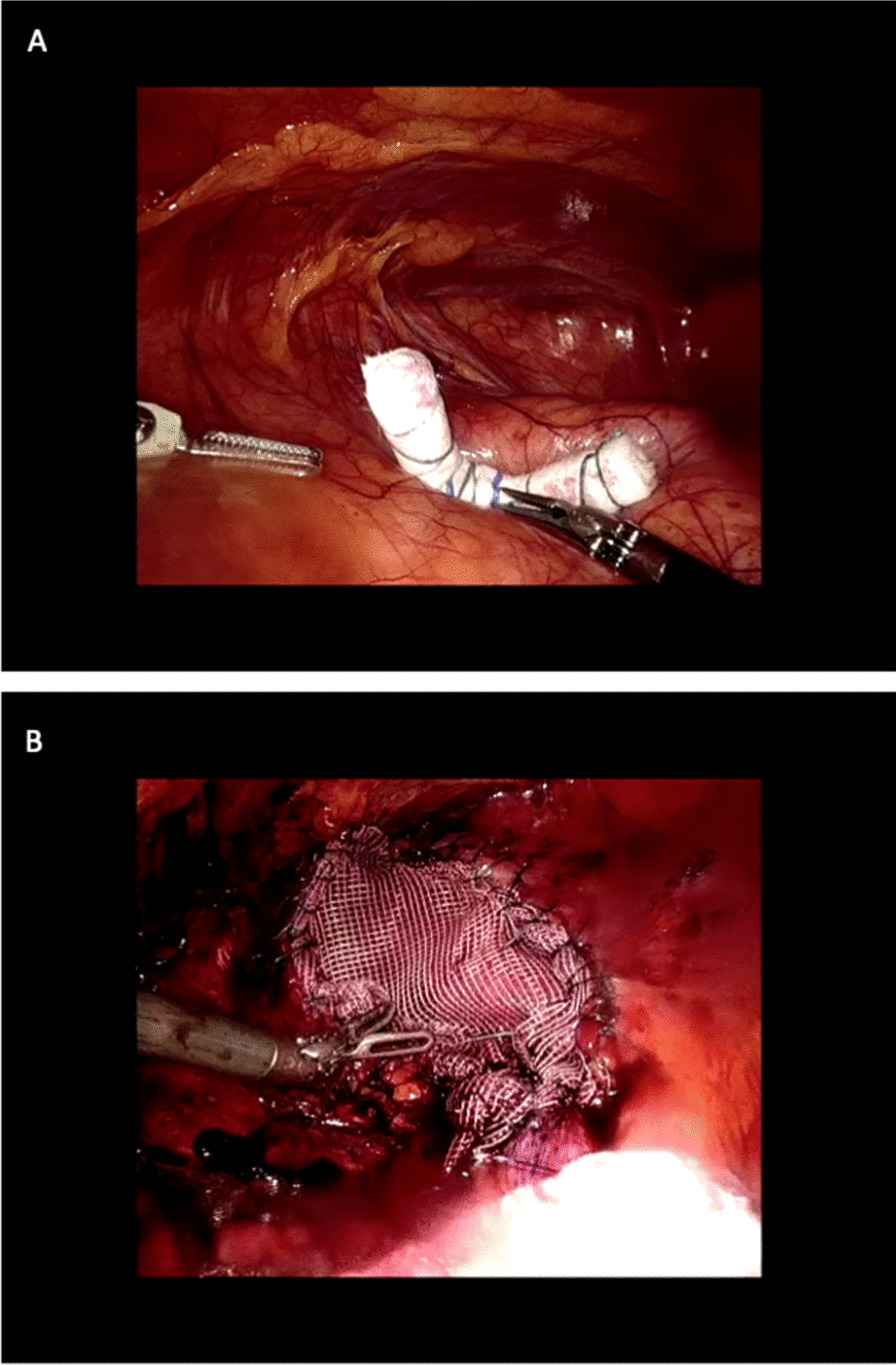


**Objectives**

We present the case of a Trisomy-21 patient who was diagnosed with agenesis of his left hemi-diaphragm which was successfully reconstructed via a robotic approach.

**Case presentation**

A 47-year-old Trisomy 21 patient was referred to our department because of a huge posterolateral agenesis of his diaphragm causing severe breathlessness. He underwent a robotic reconstruction with the DaVinci X robotic system using 3 × 8 mm and 1 × 12 mm ports. A Parietex 15 × 15 cm was initially fashioned inside the abdomen and then a dual mesh strengthened the reconstruction. A single 24Fr drain was left in situ. The patient reported minimal pain after the procedure which was mainly managed with oral analgesia. His drain was removed on day 2 and so did his nasogastric tube and his urine catheter as he was mobilizing adequately. He was discharged home on day 4. On follow up he was found with a small pleural collection which was drained without any further issues.

**Conclusions**

We present the case of a successful robotic reconstruction of a complicated agenesis of the diaphragm in a patient who benefited from avoiding a thoracotomy/thoraco-abdominal incision. This case demonstrates the merits of persevering with a key-hole approach.

Informed consent to publish had been obtained.

### A133 Evaluating the Role of Surgical Resection and Reconstruction in the Management of Paediatric Ewing Sarcoma of the Chest Wall; A Systematic Review

#### Rice, Darragh^1^, Dr; Barrett, Sean^1^, Dr; Khan, Niall^1^, Dr; Fleck, Robert^1^, Dr; McGuinness, Jonathan^2^, Mr

##### ^1^Mater Misericordiae University Hospital, Dublin, Ireland; ^2^Children's Health Ireland at Crumlin, Dublin, UK

*Journal of Cardiothoracic Surgery* 2023, **18(Supp 1)**:A133

**Background**

The management of Ewing sarcoma in children has evolved over the last 30 years with surgical role and approach following the collaborative oncology group (COG) guidelines. This review of the literature aimed to assess how these surgical guidelines have been applied in the modern era.

**Methods**

A systematic review was conducted in accordance with PRISMA guidelines across four major literature databases. Data regarding overall survival, rate of recurrence, role of surgery, adjuvant therapy role was extracted.

**Results**

17 single centre observational studies and 8 retrospective reviews of multicentre trials met criteria for final analysis. There were 1028 patients identified, with a male predominance in their adolescent years. 5-year overall survival ranged from 35 to 89%. A review in 2003 established the role for neo-adjuvant chemotherapy before surgery with improved negative margins (77% vs 50%) and reduced post-op radiotherapy requirement(48% vs 71%). There was high variation in the degree of resection of surrounding tissue to obtain free margins so the COG guidelines for resecting a normal rib above and below and 2-3 cm margins along the rib were not really followed. If negative margins were achieved, further radiotherapy was not shown to improve survival further. However, if microscopic positive margins were present then additional radiotherapy could improve survival in some studies similar to microscopic free margin resections.

**Conclusion**

The review suggests that surgery should be included as part of multimodality treatment for most patients, with the current COG guidelines for surgical margins probably being too aggressive which may limit surgery being applied for some patients. Macroscopic free margins are an absolute, but microscopic positive margins can be compensated for by radiotherapy, and neo-adjuvant chemotherapy is an absolute requirement.

### A134 Antineutrophil Cytoplasmic Antibody-Associated Valvular Heart Disease: Unicuspid Aortic-valve Ozaki and Trans-aortic Mitral Valve Repair

#### Sinha, Shubhra^1^, Miss; Endean, Alison^2^, Dr; Kandasamy, Karikalan^2^, Dr; Ooues, Georgina^2^, Dr; Robson, Joanna^3^, Dr; Platt, Martin^3^, Dr; Turner, Mark^3^, Dr; Caputo, Massimo^4^, Prof; Mussa, Shafi^4^, Mr

##### ^1^Derriford Hospital, Plymouth, UK; ^2^Royal Cornwall Hospitals NHS Trust, Truro, UK; ^3^University Hospitals Bristol and Weston NHS Trust, Bristol, UK; ^4^Bristol Heart Institute, Bristol, UK

*Journal of Cardiothoracic Surgery* 2023, **18(Supp 1)**:A134

We present the case of a previously well 18-year-old man who presented with a 3-week history of fever, epistaxis, earache, hearing loss, haemoptysis,per-rectal bleeding and haematuria. Examination identified a murmur. Further investigations led to a diagnosis cANCA/PR3-positive associated vasculitis/GPA with multiorgan involvement. Pre-operative echocardiography and cardiac magnetic resonance imaging showed a fenestration in the right coronary cusp(RCC) of a trileaflet aortic valve and an anterior mitral valve leaflet(AMVL) defect with severe regurgitation of both valves and a mildly dilated left ventricle with preserved function. Discussions were undertaken between the cardiology, cardiac surgery, rheumatology, ENT, microbiology and anaesthetic teams to optimise the patient prior to surgery and discuss the potential risk of haemorrhage, infection and recurrence of valvular regurgitation. He underwent unicuspid aortic leaflet replacement using glutaraldehyde-treated autologous pericardium (Ozaki) and transaortic mitral valve repair with a bovine patch. Avoiding the use of prosthetic material negated the need for lifelong anticoagulation and reduced the infection risk in a young immunosuppressed patient. The pre-discharge echocardiogram showed mild AR and no MR with good biventricular function. The patient was followed-up in clinic 6 weeks post-operatively and continued to make a good recovery. The echocardiogram showed normal left ventricular dimensions, no significant valvular abnormalities and excellent aortic valve haemodynamic function (peak gradient 5.3 mmHg; maximum velocity 1.2 m/s). Regular echocardiograms and close rheumatological follow-up are planned.

Informed consent to publish had been obtained.

### A135 The Impact of COVID-19 on Surgery for Congenital Heart Disease in the UK: Pilot Study

#### Sinha, Shubhra^1^, Miss; Cocomello, Lucia^2^, Ms; Suseeladevi, Arun K^2^, Mr; Baquedano, Mai^2^, Ms; Struzik, Ewa^3^, Ms; Austin, Conal^3^, Mr; Lawlor, Deborah A^2^, Prof; Caputo, M assimo^4^, Prof

##### ^1^Derriford Hospital, Plymouth, UK; ^2^University of Bristol, Bristol, UK; ^3^Guy's and St Thomas' NHS Trust, London, UK; ^4^Bristol Heart Institute, Bristol, UK

*Journal of Cardiothoracic Surgery* 2023, **18(Supp 1)**:A135

**Objective**

We report a pilot study to quantify the change in case-mix and operative volumes in congenital heart disease (CHD) surgery in the UK secondary to the first COVID-19 lockdown. This has clear implications for health provision planning and may have an impact on the clinical outcomes of this patient cohort.

**Methods**

Prospective multi-centre, cross-sectional, observational study based in the UK on consecutive patients with CHD admitted for heart surgery from 02/01/2020 to 06/07/2020. We examined changes in patient characteristics (i.e. age and clinical urgency) and complications pre- and post- the first lockdown on 23/03/20.

**Results**

317 patients underwent cardiac surgery during the study. There was an average decrease of 4 cases per week (pre-lockdown: 166; post-lockdown: 151, Fig. 1) and mean age at operation (pre-lockdown:8.2 ± 15.5 years,post-lockdown:2.7 ± 6.2 years; 95% confidence interval(CI) of difference: 3–8.1 years; p < 0.001) following lock-down. Surgical priority was also different pre and post-lockdown (Elective-55% vs 39%; Urgent-39% vs. 58%; Emergency-6% vs 3.3%). There was a 2.6% increase in hospital mortality (pre-lockdown 0%;post-lockdown 2.6%; 95% CI of the difference:-0.54% to -5.8%;p0.11) and an overall an increase in all other major complications, except sepsis. However these latter differences were not statistically significant.

**Conclusions and Relevance**

During lockdown CHD operations were more likely to be urgent and involve younger patients, resulting in a shift toward increased mortality and complications. These results need to be confirmed at national level.
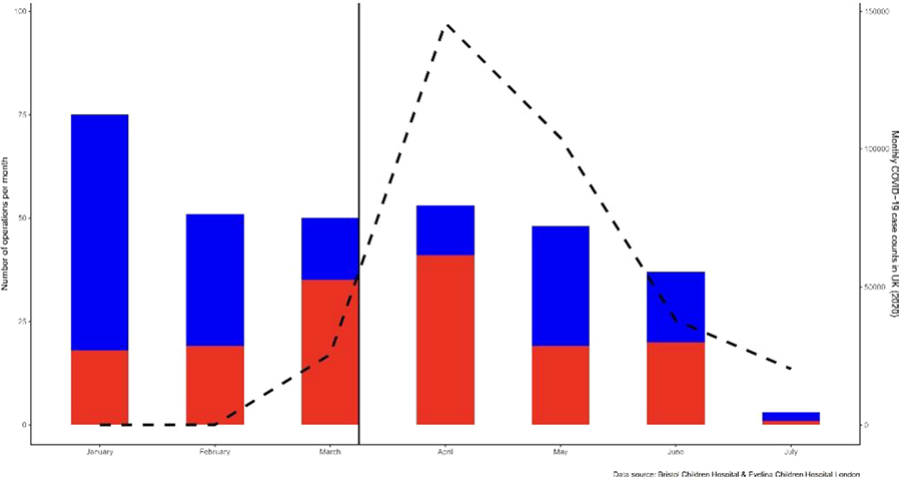


### A136 Our experience on Personalised External Aortic Root Support (PEARS) application to paediatric population

#### Redondo, Ana, Ms; Austin, Conal, Mr

##### Evelina London Children's Hospital, London, UK

*Journal of Cardiothoracic Surgery* 2023, **18(Supp 1)**:A136

**Objectives**

The PEARS technique, has been safely used in over 500 patients worldwide. The original concept was to stabilize dilatational aortopathy in Marfans syndrome, and with its proven efficacy its use has been expanded to other aortopathies. Here we report our unique experience in the paediatric age group.

**Methods**

We have reviewed our single institutional results in the application of PEARS in patients aged 18 years old and younger. We review the baseline diagnosis, aorta dimensions, intraoperative data, and short-term morbidity.

**Results**

21 patients in the paediatric cohort have undergone PEARS procedure since 2012. Mean age was 15.28 years (range 9 to 18). Most patients (42.85%) had Marfan’s Syndrome with aortic root dilatation (mean diameter 4.25 cm). Other diagnosis included bicuspid aortic valve aortopathy (n = 5), DORV (n = 2), interrupted aortic arch and post dilated Free root Ross (n = 2), dysplastic aortic valve having paediatric Free Root Ross PEARS (n = 2) and TGA (n = 1). 7 patients had previous sternotomies. 5 had a reduction aortoplasty before PEARS application. 10 had their surgery done off bypass. Mean postoperative maximum aorta diameter was 3.4 cm. Mean reduction in aortic diameter post-PEARS application was −0.87 cm. Follow-up imaging consisted of echocardiogram, MRI or CT-scans, showing stable diameters. One patient had to be reopened for pericardial effusion, while another one had to be re-operated three years later for severe aortic regurgitation which had been corrected during the PEARS post delayed arterial switch.

**Conclusions**

PEARS is an effective procedure in the paediatric Marfan’s syndrome patients and even other complex congenital conditions developing aortic dilatation.

We have proven PEARS to be a safe procedure providing stable aortic dimensions and non-interference with the aortic valve in the paediatric population.

An interesting subset has shown reversal of aortic insufficiency with PEARS application that reduces the aortic dimensions.

### A137 On-table Extubation as Part of Enhanced Recovery After Cardiac Surgery in Paediatric Population in a Tertiary Centre

#### Ashry, Amr^1^, Dr; Boyle, Mark^2^, Dr; Sunny, Jesvin^2^, Dr; Arnold, Philip^2^, Dr; Kutty, Ramesh^2^, Mr; Lotto, Attilio^2^, Prof; Guerrero, Rafael^2^, Mr; Dhannapuneni, Ramana^2^, Mr

##### ^1^Assiut University Hospital, Assiut, Egypt; ^2^Alder Hey Children's Hospital, Liverpool, UK

*Journal of Cardiothoracic Surgery* 2023, **18(Supp 1)**:A137

**Objective**

To report outcomes following on-table extubation after cardiac surgery in paediatric population.

**Methods**

Retrospective study for paediatric patients who underwent on-table extubation as part of enhanced recovery at Alder Hey Children’s Hospital, Liverpool between January 2019 and December 2019. Outcomes were to assess postoperative complications, i.e., re-intubation, bleeding, renal failure and arrhythmia in PICU.

**Results**

A total of 154 patients who were extubated on table were included. Mean age at time of operation was 4.9 ± 10.4 years. 38% of cases (n = 59) were < 1 year old. Ventricular septal defect (17.5%, n = 27) was the most common lesion, followed by atrial septal defect (11.7%, n = 18). Cardiopulmonary bypass and aortic cross-clamp time were 68.8 ± 49.4 and 32.6 ± 35.3 min, respectively. There was no mortality in our cohort, whereas 85% (n = 131) had no complications during PICU stay. Eight patients (5.2%) required re-intubation for respiratory failure and two patients (1.3%) needed re-intubation following arrhythmias. The mean length of PICU and postoperative hospital stay were 1.5 ± 1.5 and 7.6 ± 8.9 days, respectively.

**Conclusion**

On-table extubation as part of enhanced recovery after cardiac surgery is feasible and safe in paediatric population. There was no mortality and low rate of complications.

### A138 PEARS (Personalised External Aortic Root Support) Applicated at a Late Stage to Recover Failing Autograft after Ross Operation

#### Redondo, Ana, Ms; Austin, Conal, Mr

##### Evelina London Children's Hospital, London, UK

*Journal of Cardiothoracic Surgery* 2023, **18(Supp 1)**:A138

**Objective**

Ross operation is the aortic valve replacement procedure of choice in growing paediatric patients and young adults. Free root Ross operation is associated with autograft dilatation and ultimately valve failure in some patients.

PEARS has been successfully deployed since 2004 in Marfan patients, and this concept lead to our utilisation of this technique to support, reduce aortic size and recover aortic valve insufficiency in failing Ross patients.

**Methods**

Three male teenagers aged between 15 and 16 years had several echo studies showing increasing autograft dilatation and new moderate aortic valve insufficiency in two of them, 3 to 4 years after having undergone free root Ross operation. Maximum aortic root diameter ranged between 4.5 and 4.8 cm. They had up to 5 previous cardiac procedures, including interrupted aortic arch repair and VSD closure.

CT scans were performed and PEARS were manufactured by Exstent to produce a 20% reduction sized prosthesis of the current aortic dimensions.

Reduction PEARS were applied to the whole aortic root from the subcoronary ventriculoarterial junction to beyond the distal suture line of the autograft. One was applied off bypass and two required beating heart cardiopulmonary bypass.

**Results**

All patients had successful application of the 80% PEARS prosthesis and in both cases of moderate aortic valve insufficiency this was abolished or reduced to trivial. The first patient had a transient arm weakness. Aortic root size was reduced for more than 1 cm in all cases, and it showed stable dimensions in further imaging. Post-operative hospital stay ranged from 4 to 9 days.

**Conclusion**

PEARS can be applied in a ‘reduced’ fashion to stabilise the dilating aortic root in failing Ross operations and recover and abolish moderate aortic valve insufficiency. This new technique should be considered early in Ross patients undergoing follow-up with dilating autograft root and associated worsening aortic valve insufficiency.

## Nursing & AHP Forum

### A139 The Cardiac surgery internatiONal Nursing and alliEd professional researCh network: CONNECT

#### Sanders, Julie^1^, Professor; Fredericks, Suzanne^2^, Prof; Martorella, Geraldine^3^, Dr; Wynne, Rochelle^4^, Prof

##### ^1^St Bartholomew's Hospital, London, UK; ^2^Ryerson University, Toronto, Canada; ^3^Florida State University, Florida, USA; ^4^Western Sydney University, Penrith, NSW, Australia

*Journal of Cardiothoracic Surgery* 2023, **18(Supp 1)**:A139

**Objectives**

The number of nursing and allied professional (NAP) clinical academics is low, particularly in cardiovascular surgery. This undoubtedly affects the ability of the profession to advance and improve healthcare delivery and patient outcomes and experience. Since there is global recognition that increasing NAP clinical academics is needed, we sought to establish an international NAP network to strengthen collaborative NAP cardiovascular surgery research through shared initiatives including supervision, mentorship, workplace exchange programs and multi-site clinical research.

**Methods**

CONNECT is a virtual network established by NAP clinical academics from the UK, Canada, Australia and USA. A website (https://www.qmul.ac.uk/whri/research/connect/) and twitter profile (@CONNECTcardiac) were established and the network was launched at the European Society of Cardiology Association of Cardiovascular Nursing and Allied Professional (ACNAP) EuroHeartCare conference in June 2021.

**Results**

CONNECT has attracted members from the UK, Denmark, Norway, Canada and Australia and followers from around the World. The first of a series of webinars will be hosted in November 2021. A program of collaborative research work is being mapped. Efforts to increase the awareness of CONNECT, while continuing to attract new members globally, continue. By March 2022 we plan to present an update on membership and showcase opportunities for NAP cardiovascular researchers.

**Conclusions**

Although early in development, CONNECT has attracted global interest and is providing opportunities for international supervision/collaboration. In time, it is anticipated the network will foster and develop NAP-led cardiovascular surgery research to address global cardiac surgery challenges.

### A140 Results from the Implementation of a Modified Enhanced Recovery Programme in a Cardiac Surgical Intensive Care Unit—A Comparative Study

#### Tuff, Cheryl^1^, Mrs; McNeilly, Graham^2^, Dr; Brown, Donna^3^, Dr; Chaney, Ursula^3^, Ms

##### ^1^Belfast Health and Social Care Trust, Belfast, UK; ^2^Royal Victoria Hospital, Belfast, UK; ^3^University of Ulster, Londonderry, UK

*Journal of Cardiothoracic Surgery* 2023, **18(Supp 1)**:A140

**Objective**

To evaluate the impact that a modified enhanced recovery program has had on ventilation times, critical care length of stay and hospital length of stay in a Cardiac Surgical Intensive Care Unit (CSICU). Project implemented in response to data received from NCBC re critical care length of stay being higher than the national average.

**Methods**

Quantitative approach taken and data were retrospectively collected from two years (April 2015- March 2016 and April 2018-March 2019). Data collected included gender, age, risk scores, surgical procedure, bypass and cross clamp times. Data was also collected for ventilation times, critical care stay and hospital length of stay. Data analysis was performed using SPSS (version 27).

According to the Raosoft online sample calculator, a sample size of 278 in each group was required to provide 95% confidence level with 5% margin of error.

Inclusion criteria was data from cardiac surgical cases between the dates given. Exclusion criteria included TAVIs, cases nursed with open chests, intra-aortic balloon pump, nitric oxide and continuous renal replacement therapy post-operatively.

**Results**

**Table:** 

Year	Ventilation Time (Hours)	Critical Care Length of Stay (Days)	Hospital Length of Stay (Days)
2016–2016 (Pre-ERAS)	11.57	5.21	13.32
2018–2019 (Post-ERAS)	9.08	4.26	13.85

Statistically significant reduction in critical care length of stay (data abnormally distributed therefore Mann–Whitney U Test performed, significance level < 0.050, result < 0.001).
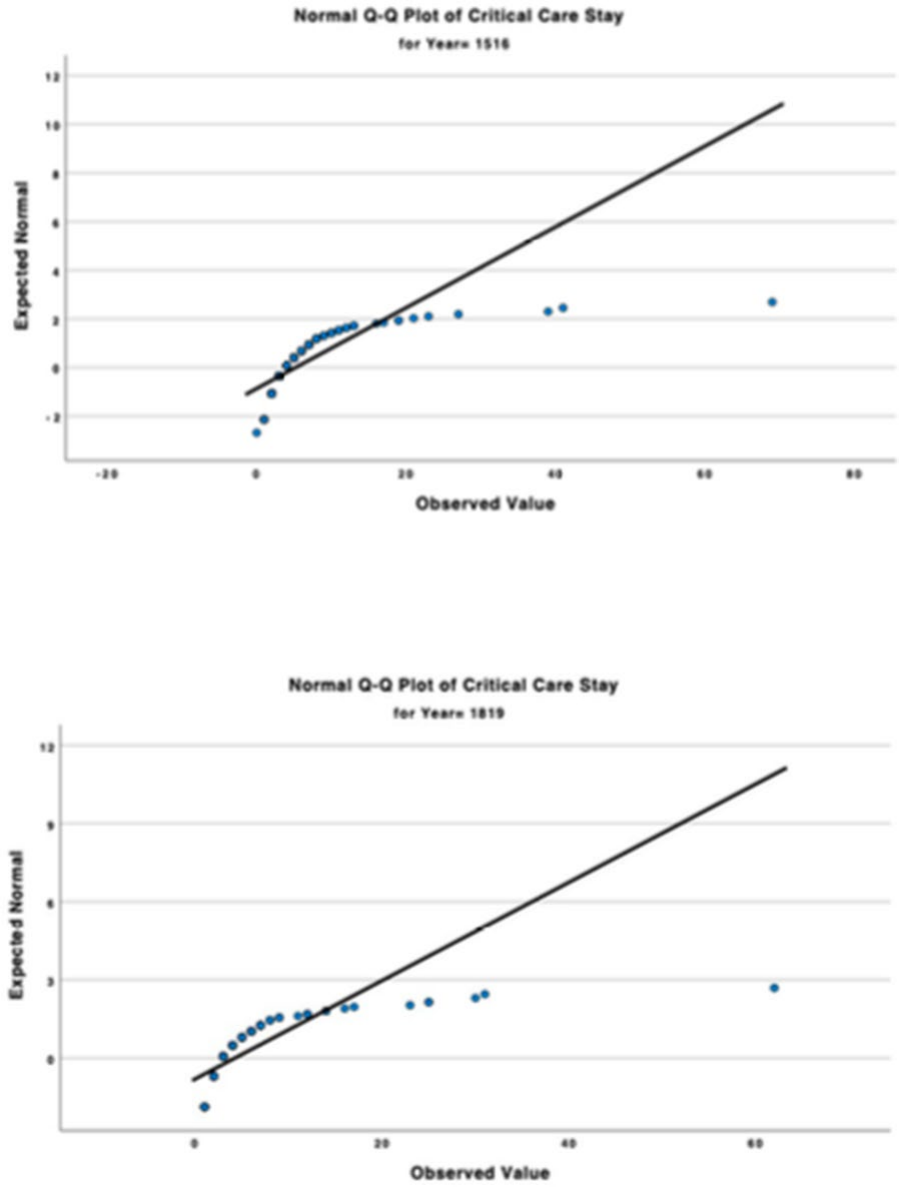


Insignificant reduction in ventilation times (11.57 h vs 9.08 h).

Hospital stay was unchanged (13.32 days vs 13.85 days).

Readmissions to critical care were similar for both years (1.07% each year).

**Conclusions**

Enhanced recovery measures show promising results in cardiac surgery patients, and have contributed to the reduction of critical care length of stay and ventilation times. Further work to be carried out in an attempt to uncover barriers to progress on hospital length of stay. Future work should include complication rates and experiences of patients and staff in the delivery of an ERAS programme.

### A141 What are the Experiences of Health Care Professional's (HCP) Providing Guidance for Patients Following Surgery for Emergency Type A Aortic Dissection?

#### Hewitt, Kathryn^1^, Ms; Inman, Chris^2^, Dr

##### ^1^Queen Elizabeth Hospital, Birmingham, UK; ^2^Birmingham City University, Birmingham, UK

*Journal of Cardiothoracic Surgery* 2023, **18(Supp 1)**:A141

**Background**

Following surgical intervention for Type A Aortic Dissection (TAAD), the remaining aorta is predisposed to further disruption, thus, life-long surveillance, patient guidance and support should exist.

**Method**

Following ethical approval, semi-structured, face-to-face interviews using an IPA methodology were conducted. Eight purposely selected participants, four nurses and four surgeons, meeting inclusion and exclusion criteria were identified to provide views emerged from HCPs most involved with care. Participants’ experiences were explored to answer the research question. Interviews were recorded and transcribed verbatim and analysed using an IPA approach.

**Findings**

Three super-ordinate themes were identified; Holistic Awareness, Information Provision and Clinical Uncertainty, alongside further sub-ordinate-themes. Original ideas were identified including the importance of family involvement post TAAD for continuing care and need to support patient recall. The prevalence of patient stress, anxiety and depression, exacerbated by the shock of unsuspected, debilitating diagnosis and the emergency situation were identified as original and key themes, with importance placed on consistent patient education and psychological support to address this. The study highlighted concern surrounding the concept of ‘postcode lottery’ care, including staff education and awareness, and a paucity of national and local guidance to help institutes reach and maintain a standard of patient-centred-care. The findings identified shortfalls in current practice and acknowledged the importance of the MDT and the potential for ACPs to address these shortfalls.

**Conclusion**

This IPA study offers a contribution to understanding of the phenomenon outlined and provides insight to challenges faced by HCPs to provide quality guidance. This study is concluded by ascertaining areas of improvements within healthcare policy and thus identifying recommendations for practice and need for further research.

### A142 Short Duration ECMO as a Saviour for Pulmonary Hemorrhage in Post Chronic Thrombo Embolic Pulmonary Hypertension (CTEPH) Cases

#### Selvaraj, Sam, Mr; P V S, Prakash, Mr; Rajamani, Selvakumar, Mr; Shetty, Varun, Dr; Venu, Gokul, Mr; Thekumkattil T, Thomas, Mr

##### Narayana Health Hospitals, Bangalore, India

*Journal of Cardiothoracic Surgery* 2023, **18(Supp 1)**:A142

**Objective**

The role of Short Term ECMO (Extra Corporeal Membrane Oxygenation) in Chronic Thrombo Embolic Pulmonary Hypertension (CTEPH) cases as a saviour in post-surgery complication of pulmonary Hemorrage.

**Methods**

CTEPH cases are extremely challenging to operate and in some circumstances it possess a high incidence of pulmonary Hemorrhage soon after the endarterectomy. We report our modest experience of six cases which required VA ECMO due to severe pulmonary hemorrhage post-surgery. Central VA ECMO was instituted in all the cases to tackle the crisis scenario of Hypoxia, Hypercarbia, Hypotension and Bleeding. The VA ECMO enabled us to come off from Cardio Pulmonary Bypass and gave us a fighting chance to encounter the emergency situation. Patients were offered single lung ventilation to isolate the soiling of healthy lung from the severe pulmonary bleeding getting into the air compartment of the lungs. **We could completely reverse the Heparin on ECMO with Protamin(Target ACT ~ 140 Sec)**. ECMO provides better ventilation perfusion ratio with good tissue perfusion TEG played a vital role to transfuse the blood products to the patient appropriately. Topical agents were used to arrest the bleed. The cell saver was used to salvage the red blood cells.

**Results**

All the six cases were weaned off from ECMO Successfully with the Average ECMO run of 6 Hours; Lowest being 67 min and the Maximum of 12 h. ACT was brought down to 120–140 secs in all cases. Delta P and the Ecmo Circuit was monitored closely as we reversed the heparin completely. A backup circuit was also kept ready in case of any eventuality.

**Conclusion**

Short duration of VA ECMO in patients with pulmonary haemorrhage after CTEPH helped us to reverse the heparin and to control the bleeding. VA ECMO offered to address the problem of hypotension with better hemodynamics and provided good gas exchange in this critical condition. VA ECMO was the only choice for this subset providing 100% survival results.

### A143 A Review of Mechanical Insufflation-Exsufflation as a Treatment for Patients Following Extended Pleurectomy-Decortication Surgery

#### Husemann, Zelie, Ms; Streets, Emma, Miss

##### Barts Health NHS Trust, London, UK

*Journal of Cardiothoracic Surgery* 2023, **18(Supp 1)**:A143

**Objectives**

Extended Pleurectomy-Decortication (EPD) surgery is a radical treatment option for epithelial mesothelioma and can be associated with an increased risk of post-operative complications including sputum retention (Martino et al., 2018). Mechanical-Insufflation-Exsufflation (MI:E) can be used as a treatment intervention to aid sputum clearance in other patient populations (Chatwin et al., 2003). However, there is a paucity of high-grade evidence supporting MI:E in this cohort. The aim of this review was to investigate the feasibility and safety of the use of MI:E with EPD patients and to evaluate any adverse effects.

**Methods**

All patients undergoing EPD surgery were retrospectively evaluated using electronic records over a 12-month period between June 2020–21. Outcomes included patient tolerance to the intervention and evidence of adverse effects during or after treatment.

**Results**

A total of 21 patients were included in the analysis, 1 patient was excluded due to in-hospital mortality. 9 out of 20 patients (45%) received MI:E treatment. The mean number of treatment sessions was 6 (SD ± 5.13). Of the patients who received MI:E, 1 patient experienced a transient adverse event of haemo-dynamic instability but required no medical intervention.

**Conclusions**

In this review, MI:E was safe and feasible for patients undergoing EPD surgery and highlighted no lasting adverse effects. However, a larger observational review is required to further validate this finding.

### A144 Preventable Post Discharge Problems in Thoracic Surgery Patients

#### Kenyon, Lisa, Ms; Cahill, Jo, Ms; Naidu, Babu, Mr; Kalkat, Maninder, Mr

##### Queen Elizabeth Hospital Birmingham, Birmingham, UK

*Journal of Cardiothoracic Surgery* 2023, **18(Supp 1)**:A144

**Objective**

The objective of the audit was to ascertain what problems patients experience after discharge having undergone Thoracic surgery and to discover if these were preventable by auditing adherence to our local discharge protocol.

**Method**

The last 20 patients discharged from the service were audited by reviewing their Advanced Clinical Practitioner (ACP) post discharge telephone appointment documentation and cross referencing any problems encountered with their discharge letter and medications. The National Lung Cancer Telephone Assessment tool was completed for all patients: this aids identification of post discharge problems in the areas of pain, activity, wounds, infection, diet/appetite, nausea, constipation, mobility/exercise, psychological issues, sleep and other concerns.

**Results**

Four main problems were identified: pain, constipation, wound/suture issues and lack of activity. One week after discharge 35% of patients were in significant pain, 20% of those had not been prescribed sufficient analgesia. 20% were constipated but had not been prescribed any laxatives despite being discharged with opiates. 33% were inactive; mainly due to pain, shortness of breath, fear and constipation. 25% still had wound dressings in place and a drain suture more than 7 days after drain removal. 30% had missing or incorrect suture instructions on their discharge letter.

**Conclusions**

Unfamiliarity of protocols/Enhanced Recovery After Surgery goals caused post discharge problems which were mostly preventable. Education is key for nursing, medical staff and patients. Following this audit a simple discharge checklist was introduced for staff and patients to read and sign and a discharge medication bundle and instructions were created on electronic prescribing, aiming to reduce the incidence of such problems.

### A145 Cloud-based Clinical Charting – A Technological Revolution Service Improvement to Assist in the Cardiothoracic Organ Retrieval Process

#### Nunes, Joao Pedro, Mr; Rubino, Antonio, Dr; Berman, Marius, Mr; Pettit, Stephen, Dr; Quigley, Richard, Mr; Baxter, Jennifer, Mrs

##### Royal Papworth Hospital NHS Foundation Trust, Cambridge, UK

*Journal of Cardiothoracic Surgery* 2023, **18(Supp 1)**:A145

**Objective**

Leading world technological innovation is one of the goals of the NHS. At a leading UK Cardiothoracic Transplant Centre, the Donor Care Physiologist (DCP) team has created a new cloud-based clinical chart to be used during Scouting and Donation after Brainstem Death (DBD) retrieval. Documentation is fundamental for donor monitoring, management, the decision-making process and auditing; enabling DCPs to optimise cardiothoracic organs and improving outcomes for transplant recipients.

**Methods**

To simplify documentation during Scouting and DBD retrievals, the DCP team have built a cloud-based tool. This innovative chart combines various aspects of retrieval and standardises input of real-time data. When fully implemented members of both retrieval and recipient teams will be able to access this data real-time contributing to a swift decision-making process.

**Results**

This monitoring tool will be implemented during Scouting and DBD retrievals allowing consistent data collection in order to maintain the highest standards of record keeping and safe practice. Furthermore, feedback will influence regular updates to enhance user experience.

**Conclusion**

Cloud-based Charting hopes to revolutionise local and hopefully national record-keeping during Scouting and DBD retrieval. Moreover, the creation of this tool serves as another example of how the DCP is an invaluable member of the National Organ Retrieval Services (NORS) team.

### A146 Multi-centre Comparison of Routinely Collected NHSD/HES Rates for Surgical Site Infection with Prospective Surveillance

#### Rochon, Melissa^1^, Ms; Ahmed, Ishtiaq^2^, Mr; Chiwera, Lilian^3^, Ms; Gannon, Robert^4^, Mr; Hutton, Sandra^5^, Mrs; Morais, Carlos^6^, Mr; Peters, Molly^7^, Ms; Magboo, Rosalie^8^, Mrs; Raja, Shahzad^9^, Mr

##### ^1^Royal Brompton and Harefield hospitals, part of Guy's and St Thomas' NHS Foundation Trust, London, UK; ^2^Brighton and Sussex University Hospitals NHS Trust, Brighton, UK; ^3^Guy's and St Thomas' NHS Foundation Trust, London, UK; ^4^Royal Papworth Hospital NHS Foundation Trust, Cambridge, UK; ^5^Oxford University Hospitals NHS Foundation Trust, Oxford, UK; ^6^Royal Brompton Hospital, London, UK; ^7^University Hospitals Bristol, Bristol, UK; ^8^Barts Health NHS Trust, London, UK; ^9^Harefield Hospital, Harefield, UK

*Journal of Cardiothoracic Surgery* 2023, **18(Supp 1)**:A146

**Table:** 

Identifier	SSI Dashboard SSI rate (%)	Prospective Surveillance SSI rate (%)	X2	value	Rank SSI Dashboard	Rank Prospective Surveillance
Trust A	3.4	2.2	2.138	0.1437	1	1
Trust B	4.3	3.4	0.838	0.3598	3	3
Trust C	5.1	4.0	0.49	0.4840	5	4
Trust D	9.3	6.8	2.627	0.1051	7	6
Trust E	7.0	8.0	0.113	0.7367	6	7
Trust F	4.6	5.3	0.48	0.4883	4	5
Trust G	3.6	2.4	0.861	0.3534	2	2

**Objective**

Surgical site infection (SSI) is an important quality indicator however, collecting and reporting for SSI surveillance can be resource intensive. Our aim was to compare (unadjusted) rates of SSI using a new, 'resource-light' national SSI Dashboard with data collected via trained surveillance personnel at seven Trusts undertaking coronary artery bypass (CABG) surgery.

**Methods**

Our Cardiac SSI Network determined a national dataset based on classification and diagnostic codes in Tableau™ for the period January – December 2017. National data was suppressed (5.0001). SSI were included up to 30 days, detected on primary admission and readmission to own hospital or other hospital. Chi-square testing was used to determine whether there was a significant difference between SSI rates. Probability was set at p < 0.05. Data from both sources was ranked from lowest to highest rates.

**Results**

Across the seven hospitals, there was no statistically significant difference between the unadjusted SSI rates from the Dashboard and that collected prospectively by trained surveillance personnel at each Trust (Table 1). Both sources ranked the same Trusts with the two lowest and two highest SSI rates, with variation in the middle ranks noted.

**Conclusions**

Despite controversy regarding the identification, completeness, and verification of a simple binary approach as compared to prospective surveillance, our experience suggests that Hospital Episode Statistics (HES) using our SSI Dashboard provides reasonable information on SSI rates. Research studies coding routinely collected data for efficiency and cost-savings could look at look at this source of information for SSI data, as well as looking at how to further improving SSI data capture in clinical coding. However, prospective surveillance remains superior in terms of type and causative pathogen SSI, with the additional benefit of patient-reported SSI.

### A147 Fast Tract Service Development to Improve Patient Flow

#### Bartley, Tara, Ms; De Costa, Joana, Ms; Sadler, Carl, Mr; Dunn, Nicola, Ms; Hewitt, Kathryn, Ms; Wilkinson, Gemma, Ms; Patel, Ranj, Ms; Singh, Harjot, Dr; Bate, Cerys, Ms; Rooney, Stephen, Mr

##### University Hospitals Birmingham, Birmingham, UK

*Journal of Cardiothoracic Surgery* 2023, **18(Supp 1)**:A147

**Abstract**

Patient flow through the Cardiac surgical setting is becoming increasingly challenging, this has been exacerbated by COVID and staff attrition.

This service development established an alternative cardiac surgery pathway outside critical care environment utilising the fast track concept.

**Objectives**

To develop a fast tract pathway for post operative cardiac surgery patients that doesn’t require a Critical Care bed thus mitigate against cancelation. We have created in an alternative environment in which to recover patients with early transfer to step down on the ward.

**Method**

A multidisciplinary team approach has developed as pathway through the recovery unit. A data set was established confirming a patient selection criteria, booking system, a criteria for transfer to recovery following surgery then aim for discharge the patient to the ward stepdown area at 08.30 the following morning. Patients were seen by the surgical and anaesthetic teams who documented all parameters had been meet for transfer. The recovery staff have undergone a focused education programme.

**Results**

The data set documents if the patient was listed on the bed booker and when, if the patient met the preoperative criteria for surgery and post-operative parameters for transfer to recovery and then to the ward. Weekly meetings reviewed each case identifying delays in the pathway, with real-time response to improve flow. Early results suggest the service development has enabled surgical cases that would have otherwise be cancelled, the success indicates there is the potential to expand the number of cases undertaken via this pathway.

**Conclusions**

The service development was driven by the current crisis of reduced activity being experienced in cardiac surgery units across the country. We have developed a pathway that has successfully reduced cancelations and increased cardiac surgery cases. We have demonstrated team working across units and professional groups that prioritises patient need.

### A148 A Self Assessment Tool to Evaluate the Impact of an Interactive Course Cardiac Course on Knowledge and Clinical Confidence

#### Bartley, Tara^1^, Ms; CSU-ALS, Adrian Levine^2^, Mr; CSU-ALS, Joel Dunning^2^, Mr; Bibleraaj, Bhuvaneswari^3^, Ms

##### ^1^University Hospitals Birmingham, Birmingham, UK; ^2^CSU-ALS; ^3^Wythenshawe Hospital, Manchester, UK

*Journal of Cardiothoracic Surgery* 2023, **18(Supp 1)**:A148

**Abstract**

The development of a Self Assessment Tool to evaluate pre-course and post-course learning to underpin confidence in the clinical setting for the Cardiac Advanced Life Support Course.

**Objectives**

An evaluation of students’ perception in relation to their pre and post-knowledge was developed using a Likert scale to inform the development and impact of SCTS Nurses and Allied Health Professionals Courses. This paper will review the tool as it has been adopted for use on the Cardiac Advanced Life Support Course and expanded to incorporate participants’ confidence in performing the 6 key roles.

**Method**

A self assessment tool using a Likert scale of 1 – 5 was given to course participants to score their perception of pre and post course knowledge. Questions were in regard to each lecture and practical session and also included additional questions of level of confidence in knowledge and performing each of the six key roles.

**Results**

The Likert scale asked candidates to score each session on a scale of 1 to 5, with 1 being strongly disagree and 5 strongly agree about their increase in knowledge. Responses to each lecture and practical session demonstrated 100% of candidates felt their knowledge has improved all every aspect of the course. Further analysis demonstrated that 54.03% to 90.31% felt they knowledge had increased to a 4 or 5 on the scale. 78.85% stated that they felt ‘confidence’ or ‘very confident’ in performing the six key roles post course.

**Conclusions**

The results demonstrate the importance of evaluating educational courses to inform content, level of content and of impact for candidates and their provider organisation’s perspective. In the current health care economy, it is imperative that courses are evaluated and adapted to meet both the educational, development and service delivery needs. This study provides the evidence to demonstrate these outcomes.

### A149 Implications and Challenges of Working as a Senior Cardio-thoracic Physiotherapist in a Mixed Covid-19 and Cardio-thoracic Unit

#### Plunkett, Daire, Mr; Danaher, Dervilla, Ms

##### Mater Hospital, Dublin, Ireland

*Journal of Cardiothoracic Surgery* 2023, **18(Supp 1)**:A149

**Background**

Our cardio-thoracic department consists of a ward with 33 single rooms and an 11 bed high dependancy unit. The ward was converted to a Covid-19 ward in March 2020 as the single rooms allowed for isolation of patients. The majority of cardio-thoracic surgeries were moved off-site, meaning our caseload became mostly non-surgical for a number of months. As elective surgery returned to our centre, many challenges have arisen surrounding the provision of physiotherapy care to both surgical patients as well as the medical Covid-19 admissions we continue to have.

**Methods**

It has been important to ensure appropriate prioritisation of our caseload in order to manage both cardiothoracic surgery patients as well as Covid-19 patients. A specific focus has been placed on scheduling of patients to limit the potential spread of infection, in collaboration with our infection prevention and control team. Staff education has been important throughout the pandemic to keep up to date with considerations for physiotherapy management of Covid-19.

**Results**

We continue to manage a fluctuating number of Covid-19 admissions, up to 20 beds at the time of writing, as well as a consistent cardio-thoracic service. Many skills have been applicable across both patient cohorts including knowledge and indications for different oxygen delivery devices and non-invasive ventilatory support, oxygen prescription, airway clearance techniques and management of underlying respiratory conditions.

**Conclusion**

It has been possible to maintain a safe and sustainable service with no identified cross-infection between patients in our unit. There continue to be challenges to service provision which require ongoing adaptation and flexibility.

### A150 How Far Can We Go …….. Nurses Involvement In Novel Technologies

#### Baxter, Jennifer, Mrs; Osman, Mohamed, Mr; Berman, Marius, Mr; Kaul, Pradeep, Mr; Quigley, Richard, Mr

##### Royal Papworth Hospital NHS Foundation Trust, Cambridge, UK

*Journal of Cardiothoracic Surgery* 2023, **18(Supp 1)**:A150

At our centre we have recognised and identified the importance of nurse involvement in the use of the organ perfusion device to ensure we have a resilient service and are able to evidence the fundamental role nurses play in assessing heart function following donation after circulatory death (DCD). The success of this established nursing initiative has prompted a further development with a member of our NORS nursing team being trained to dismount the heart from the device following support and training from our retrieval surgeons. This trial further evidences the vital role, innovative thinking and constantly extending roles of our nursing team along with the desire for our surgeons to help empower our nursing team.

Training and support enabled a nurse to dismount hearts from the perfusion device ready for transplantation; this was achieved under the guidance from experienced in-house surgeons. Through the use of reflection on best practice we have designed and introduced a competency pack including Standard Operating Procedure (SOP) to reflect this novel development in our nursing practice. The SOP contains; a step by step guide to the process, questions and answers, a troubleshooting guide, videos and pictures of equipment and process and competencies to ensure safe practice.

We now have a nurse who is competent in leading the dismount of a heart from the perfusion device, with the clinical ability to give instruction of the process and independently dismount the heart and pass to the implanting surgeon.

By sharing this experience from a centre which has the greatest volume of DCD heart retrievals in the world, lessons can be learnt to benefit other transplant centres by evidencing the contribution and benefits of extended roles within the nursing team. Guidelines are in place for best practice based upon this experience and the aim moving forward will be to increase nurse involvement in this technique empowering nurses and increasing both skills set and knowledge.

### A151 The Introduction of a Chest Drain Protocol Post Cardiac Surgery in the National Centre for CardioThoracic Surgery in Ireland

#### McKeon, Elaine, Ms; McKeon, Elaine, Ms; Kinsella, Aisling, Ms

##### Mater Misericordiae University Hospital, Dublin, Ireland

*Journal of Cardiothoracic Surgery* 2023, **18(Supp 1)**:A151

**Objectives**

The aim of this study is to evaluate the introduction of a chest drain protocol in the National centre for Cardiothoracic surgery. The protocol prescribes the prompt removal of chest drains post-surgery and evaluates if this was safe or could lead to complications such as an increase in pleural effusions.

**Methods**

This is a pilot study, data collected for this study includes patients over 16 years of age admitted for cardiac surgery. The inclusion criteria are elective surgery, first cardiac operation, CABG or single valve surgeries and patients with an EF > 40%. Data was collected between postoperative day one and four on all enrolled patients. Further data included day of discharge, any post drain removal complications such as recurrent effusions and pain scores.

**Results**

Preliminary results from the initial pilot evaluation of 10 patients have illustrated no increase in pleural effusions with the use of the chest drain removal protocol. It is anticipated to have complete data on the initial six months of this study available for March 2022.

**Conclusion**

The introduction of a chest drain protocol is paramount to evaluating current practice, ensuring this is both safe, sustainable and in keeping with best practice.

### A152 The Importance of Physiotherapy in Elective Thoracic Surgical Patients: Data Collection

#### Nielsen, Louisa, Miss; Cooper, Taylor, Miss; Badran, Abdul, Mr; Alzetani, Aiman, Mr

##### University Hospital Southampton, Southampton, UK

*Journal of Cardiothoracic Surgery* 2023, **18(Supp 1)**:A152

**Objective**

Review of thoracic physiotherapy interventions, current referral criteria and service need.

**Method**

Prospective data collection of 180 elective thoracic patients treated by a physiotherapist from January 2021 to June 2021.

**Results**

Overall our referral criteria appears to appropriately highlight those high-risk thoracic patients requiring physiotherapy. 88% of elective patients were reviewed day 1 postoperatively (D1PO) by a physiotherapist. Barriers to this were weekend staffing (53%), patients in level 2 or 3 areas (37%) and human error (10%). Weekend D1PO caseload accounted for 25% of the elective thoracic service, yet only 62% were reviewed. Mean Hospital length of stay (LOS) was increased for those not reviewed D1PO compared to those that were. In level 2/3 areas, mean hospital LOS was longer with no physiotherapy review D1PO (6.25 vs 5 days). Advanced respiratory techniques were required in 36% of patients not reviewed D1PO compared to 7% who were. LOS was increased for patients who had open surgery (5.6 days) compared to VATS (3.6 days), and LOS was reduced in both groups when the patient was seen by physiotherapy D1PO.

**Conclusion**

Our current thoracic referral criteria appears appropriate. We found that elective thoracic patients are more likely to have a reduced LOS and are less likely to require advanced respiratory techniques when reviewed D1PO. This highlights a need for potential service developments within our weekend and Level 2/3 elective thoracic caseload to optimise all patients within service.

### A153 The Experience of Managing a CardioThoracic Surgery Ward in the Covid-19 Pandemic

#### Jones, Mary Elizabeth, Miss; Sheridan, Nina, Miss; Brown, Rachel, Mrs

##### Mater Misericordiae University Hospital, Dublin, Ireland

*Journal of Cardiothoracic Surgery* 2023, **18(Supp 1)**:A153

**Introduction**

In March 2020 the cardiothoracic (CT) ward was identified to be one of the hospitals Covid-19 units. 18 months later and the ward has remained Covid-19 (20 beds) combined with 13 CT beds.

**Methods**

Patient safety, safe staffing, team well-being and retention are some of the considerations that have challenged the CT managers over the past 18 months. The increase in acuity and the skills required for the care of the deteriorating Covid-19 patient has been well placed in a CT ward. The knowledge and skills of CT nurses was paramount to the successful transition of caring for this acute and diverse population.

**Results**

100% of staff were upskilled to care for patients requiring non-invasive ventilation compared to 50% previously. No cross infection of Covid-19 was identified between March 2020 and October 2021. Despite the high level of care provided by the CT nursing team, 25% have resigned due to the loss of their speciality coupled with the impact of the pandemic professionally.

**Conclusion**

The CT ward and team have been well placed to care for this diverse group of patients however staff well-being is a consideration that needs to continue to be supported as the numbers of resignations have indicated.

### A154 Lessons Learnt: Implementing ISLA Proactive Surgical Wound Surveillance in Three Cardiac Hospitals

#### Rochon, Melissa^1^, Ms; Connolly, Katie^2^, Ms; Fabroa, Sheena^2^, Ms; Morais, Carlos^3^, Mr; Nkolimbo, Casim^4^, Mr; Masood, Sehar^4^, Ms; Lukban-Bunalade, Russel^4^, Mrs; Ferrett, Jessica^4^, Ms; Bagona, Lhea^4^, Ms; Metwalli, Amr^1^, Dr

##### ^1^Royal Brompton and Harefield Hospitals, part of Guy's and St Thomas' NHS Foundation Trust, London, UK; ^2^King's College Hospital NHS Foundation Trust, London, UK; ^3^Royal Brompton Hospital, London, UK; ^4^Harefield Hospital, Harefield, UK

*Journal of Cardiothoracic Surgery* 2023, **18(Supp 1)**:A154


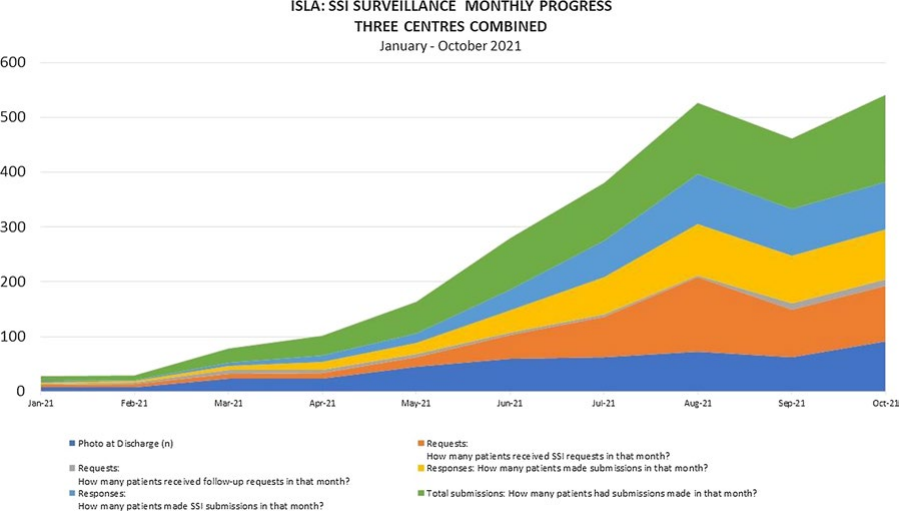


**Objectives**

A nurse-led initiative to design, procure and implement a digital SSI surveillance using a visual component (photographs) across the patient pathway.

**Methods**

Following a successful application for funding, 1) we ran a competitive procurement process for an SSI app for in-hospital and community use by patients and clinicians; 2) we designed, tested and implemented the system at multiple sites, 3) we trained staff to use the app and 4) we set up standard operating procedures for reviewing (and if necessary referring) patient submissions. To determine overall technology acceptance, we used a five-point Likert scale electronic survey which patients could self-select to complete.

**Findings**

Between April – October 2021, 66 nursing staff were trained on ISLA at three hospital sites. A total of 454 cardiac surgical patients received a photo at discharge using ISLA, and a total of 756 patient submissions were received. Overall, patient feedback has been extremely positive (mean 4.56 out of a possible 5 for patient satisfaction with the app based on 133 responses). Key challenges identified were redeployment of champions due to Covid, training, resources, and testing and refining the new technology. Local champions, senior support, IT project leads and patient engagement were key drivers to successful implementation.

**Conclusions**

This nurse-led project for SSI surveillance focuses on improving patient experience and outcomes after surgery. From our experience we have designed a blended implementation strategy (toolkit) to help spread and sustain the project.

### A155 Introduction of a Nurse-Led Chest Drain Clinic

#### Kelly, Michelle, Ms; Eaton, Donna, Prof; Redmond, Karen, Prof; Shanahan, Ben, Mr; Brown, Rachel, Ms

##### Mater Misericordiae University Hospital, Dublin, Ireland

*Journal of Cardiothoracic Surgery* 2023, **18(Supp 1)**:A155

**Introduction**

Chest drain management and outpatient review has previously been carried out ad-hoc by the Thoracic surgical team. The advantages of a nurse-led outpatient review for specific conditions have been well established in other areas such as nurse-led respiratory clinics. A need for a protocol-driven nurse-led ambulatory chest drain service was identified within our unit.

**Methods**

The Thoracic CNS underwent training for management of chest drains. A retrospective review of patients discharged home with either an intercostal drain or indwelling pleural catheter insitu who required outpatient follow-up was performed.

**Results**

144 clinical episodes involving outpatient/ambulatory chest drain management were identified over a 12-month period. In addition, the indwelling pleural catheter cohort are daycase procedures which require follow up at 1–2 weeks post-procedure and at the time of catheter removal. This accounts for a further 160 outpatient episodes over the past year. Of these patients, there were no reports of any safety issues on discharge.

**Conclusion**

It is a safe and cost-saving initiative to introduce a nurse-led chest drain outpatient service for this population. Furthermore, this will reduce outpatient appointment episodes and allow for increased new referrals to be seen in a timely fashion in the consultant-led clinics.

### A156 The Use of Simulation Training to Provide Advanced Emergency Nursing Management of Deteriorating Cardiothoracic Patients in a High Dependency Unit

#### McKeon, Elaine; Ms; Brown, Rachel, Ms

##### Mater Misericordiae University Hospital, Dublin, Ireland

*Journal of Cardiothoracic Surgery* 2023, **18(Supp 1)**:A156

**Introduction**

Simulation is widely used for training in healthcare. It was expected that simulation would address training needs and improve the clinical assessment and treatment skills of nurses in a cardiothoracic high dependency unit (HDU).

**Methods**

Pre and post-training evaluation was completed. Training sessions took place in the HDU and were facilitated by the clinical facilitator who provides bedside education and support for all nursing team members. Interactive mannequins were manipulated to change physiological variables based on clinical scenarios mirroring real patient situations within this patient cohort.

**Results**

Attendees were asked to assess and treat an interactive mannequin based on real patient scenarios. 100% of attendees reported simulation addressed their training needs, improved skills, confidence, and inter-professional communication when in real-world situations.

**Conclusion**

Integration of simulation in nursing is widely recommended, it helps combine theory and practical skills for solving increasingly complex scenarios and preparing for future clinical practice in a supportive learning environment. It is planned to explore engaging the wider MDT and involving consultant led scenarios and feedback and developing CALS within the unit.

### A157 Proactive Surgical Wound Surveillance Using ISLA: Preliminary Findings from Two Hospitals

#### Metwalli, Amr^1^, Mr; Garg, Sheena^2^, Ms; Rochon, Melissa^3^, Ms; Morais, Carlos^1^, Mr; Jawarchan, Angila^2^, Mrs; Quarto, Cesare^1^, Mr; Petrou, Mario^4^, Mr; Raja, Shahzad^2^, Mr

##### ^1^Royal Brompton Hospital, London, UK; ^2^Harefield Hospital, Harefield, UK; ^3^Royal Brompton and Harefield Hospitals, part of Guy's and St Thomas' NHS Foundation Trust, London, UK; ^4^Royal Brompton and Harefield Hospitals, part of GSTT, London, UK

*Journal of Cardiothoracic Surgery* 2023, **18(Supp 1)**:A157

**Objectives**

Surgical site infections (SSI) are a leading cause of surgical revision, readmission, surgical sepsis and an important source of antimicrobial resistance. GIRFT has called for postoperative wound monitoring as an intervention to prevent SSI and reduce the severity. We introduced ISLA for proactive surgical wound surveillance and retrospectively audited opportunities for earlier detection of concerns.

**Methods**

Wound images submitted ≥ 7 days after discharge from 269 patients were scored by two independent reviewers (SpR Grade). Reviewer agreement for image quality, healing or non-healing status was calculated using Cohen’s kappa. Readmission days and surgical revision data was collected for patients who used ISLA and patients with routine follow up care (non-ISLA). Comparison was made using Fisher exact test and significance was set at p < 0.5.

**Findings**

Reviewer agreement for images which would prompt referral for face-to-face review by healthcare worker was Moderate (95.91% agreement; Cohen’s k: 0.455) and Fair for concerns over potential infection (92.16% agreement; Cohen’s k: 0.333). Reviewer agreement regarding image quality was 94.80%. No patients submitting to ISLA required readmission for SSI or reoperation for wound infection (0/269) compared with 10 out of 914 patients who received routine follow up. Non-ISLA patients required a combined total 253 bed days and 23 theatre slots for wound management. Although the risk of readmission for SSI was not significant (p = 0.129), the risk for return to theatre for wound revision was significant between the two groups (p = 0.0043).

**Conclusion**

Proactive surgical wound surveillance via ISLA may help to identify wounds at risk of infection and provide an opportunity for early intervention to reduce the risk of more serious infection. Future work to refine categories and the standard operating procedure for image reviews (including reply to patient and referrals) is planned.

### A158 The Clinical Impact of the NHSEI Covid-19 Harm Review on Patients Currently on the Cardiac Surgical Waiting List: One Unit's Experience

#### Bannister, Christina, Ms; Clapon, Ioana, Miss; Shinn, Oksana, Miss; Ohri, Sunil, Prof

##### University Hospital Southampton NHS Foundation Trust, Southampton, UK

*Journal of Cardiothoracic Surgery* 2023, **18(Supp 1)**:A158

**Objectives**

In Sept 21 a Harm Review was undertaken to clinically review P2 patients waiting for cardiac surgery in the Covid-19 era. P2 patients, according to the RCS, need surgery that can be deferred for up to 4 weeks; patients with severe AS, MR & unstable coronary symptoms. The aim was to identify patients who were increasingly symptomatic; to maintain safety for those waiting, to reassess in clinic any patient with worsening symptoms, & to reprioritise any deteriorating patient due to clinical need &/or admit them directly for urgent surgery.

**Methods**

117 P2 patients received a call to ascertain their current clinical symptoms. According to the NHSEI guideline used each patient's waiting time was identified along with the reasons for the delay, patient/GP involvement & the current clinical harm rating (none, mild, moderate or severe harm). Patients with similar or no worsening symptoms were rated as mild & those with progressive symptoms but who felt they did not want a surgical review were rated as moderate. The third group with progressive symptoms needing an outpatient review were planned to be rated at time of reassessment to accurately identify their clinical harm rating & reprioritise if necessary.

**Results**

Of 117 patients contacted 34 were given a mild & 33 a moderate rating. The 3rd group of 50 are currently being reassessed within the outpatient footprint over the Autumn period. A snapshot outpatient clinic with 3 Harm Review patients seen resulted in 2 patients continuing on the waiting list as P2 priorities, however the 3rd patient had deteriorated significantly & was admitted from the clinic & listed as an inpatient for urgent cardiac surgery.

**Conclusions**

The Harm Review is a useful tool to ensure safety for patients waiting for cardiac surgery. Most patients found the calls reassuring & welcomed the continued contact during prolonged waiting times. The review enables the surgical team an effective means of expediting deteriorating patients surgery.

### A159 Demonstrating the Need for an Occupational Therapy Saturday Working Service

#### Chadwick, Amy, Mrs; Magpantay, Amil, Mr

##### Royal Papworth Hospital NHS Foundation Trust, Cambridge, UK

*Journal of Cardiothoracic Surgery* 2023, **18(Supp 1)**:A159

**Objectives**

The purpose of the study is to determine the need for an occupational therapy Saturday service, in a tertiary cardio-thoracic hospital. It is acknowledged that without AHPs in hospital settings, there would no flow, as patient’s length of stay would be increased and discharges would grind to a halt (NHS Improvement and NHS England, 2018). Part of this involves providing rehabilitation, which is crucial for people aged 80 years and over, as ten days spent in a hospital bed equates to ten years of muscle wasting (NHS England, 2016).

Furthermore, Wu (2020) explains that increasing evidence is demonstrating that the social isolation and loneliness from COVID-19, is having profound effects on people’s physical and mental health. This has been noticed in the authors' practice setting, as patients who are living with frailty are more deconditioned and taking longer to recover post-surgery.

**Methods**

An audit of patients referred to the occupational therapy department during July – August 2021 will be completed by 10/12/21.

Multi-disciplinary team (MDT) opinion on the perceived benefits and challenges of occupational therapy Saturday working will also be sought via a questionnaire, to assist forming the justification for funding extra staffing.

**Results**

The following information will be obtained from the audit:

- How many new patients could have been assessed on a Saturday, compared to the following Monday
- How many patients could have been discharged from the service and/or hospital over the weekend
- How many ongoing patients could have received a follow-up session

The questionnaire data will be collated to determine the main benefits and challenges to providing a Saturday working service.

**Conclusions**

The study will conclude through analysis of results, whether providing an occupational therapy Saturday service is potentially able to enhance quality of care, shorten length of hospital stay and be cost-effective.

### A160 First Assistant for the Thoracic Robotic Surgery Programme

#### Brown, Rachel, Mrs; Redmond, Karen, Prof; Eaton, Donna, Prof

##### Mater Misericordiae University Hospital, Dublin, Ireland

*Journal of Cardiothoracic Surgery* 2023, **18(Supp 1)**:A160

**Introduction**

In 2019 a multi-speciality robotic programme was introduced to the hospital. A service need was identified for a trained first-assistant to optimise the thoracic programme. We determined that this need would be best met by a nurse-led service.

**Methods**

The Thoracic ANP underwent training to be the first-assistant, this included theoretical and practical skills supported by a thoracic consultant. Training included a competency-based programme; a basic surgical skills course, the robotic first assistant course, hands-on robotic training with observation and supervision from the thoracic consultant.

**Results**

111 Robotic procedures (80 in main hospital, 31 in other centres) have been completed since the start of the programme. The robotic programme was suspended during the Covid-19 pandemic. April 2021 allowed a limited re-introduction with the option to operate in other institutions. Over 90% of all cases the ANP has scrubbed as first-assistant or supporting a junior doctor. There have been no intra or post-operative adverse events; 1 conversion to open for surgical bleeding with no significant blood loss and a smooth transition from robotic to open.

**Conclusion**

It is a safe and cost-saving initiative which has facilitated streamlining and trouble-shooting of current practice improving peri and post-procedural care for this population.

### A161 Non- Pharmacological Nursing Interventions to Relieve Pain in Adult Critical Care

#### Giblin, Siobhan, Ms

##### Galway University Hospital, Galway, Ireland

*Journal of Cardiothoracic Surgery* 2023, **18(Supp 1)**:A161

**Introduction**

- Pain remains an unmet need for many critically ill patients, with up to 70% of patients reporting moderate to severe pain during their ICU stay.
- Unrelieved acute pain causes patient distress and can transition to chronic pain with long lasting physical, psychological and emotional implications.

Following an extensive literature review the following interventions were found to have the most robust evidence base:

- Simple Massage.

- Music therapy.

- Cognitive engagement.

- Family involvement.

- Cryotherapy.

- Early mobility.

- Reducing environmental stressors.

**Implications for Nursing**

- Increased awareness and time management skills required to integrate aspects into routine care.
- Acquiring physical resources to implement interventions.
- Evaluating results of intervention on alleviation of pain.

**Implications for the Patient/Family**

- Reduced need for breakthrough analgesia, increased efficacy from analgesic regime.
- Increased satisfaction in critical care journey.
- Reduced risk of complications during their ICU stay e.g. pneumonia, delirium.

**The Future**

- Further rigorous research.
- Development of nursing intervention protocol for pain management within critical care.
- Virtual Reality.

### A162 Every Little Success: Learn Well, Help More, Explore Further, Influence Greater

#### Wang, Yi, Ms.

##### Royal Brompton and Harefield hospitals, part of Guy's and St Thomas' NHS Foundation Trust, London, UK.

*Journal of Cardiothoracic Surgery* 2023, **18(Supp 1)**:A162

This abstract presents a fulfilment in nursing educational career development and achievement in lifelong learning as a nurse. It describes a journey from a laybacked, under-developed nurse working way up towards to nurse educator and facilitator. It demonstrates the paramount in motivation and determination intrinsically while expressing inspiration and encouragement extrinsically in every little success throughout the learning journey. The presentation shares the author's learning experience as a learner and addresses the significance of being a good teacher in academic and clinical settings. After years of layback and so-called relaxed life, the author finally became fed up with being low self-valued and out of date in technologies. A loud voice from the depth of the author's heart screamed for a change. Having been inspired and encouraged by lecturers and clinical teachers in their advanced educational achievement, the author has been motivated and determined to fulfil nursing education goals and future development in nursing research. Thoughout a hard but gaining leanring jouenry, the author has achieved some academic status with all supports, such as winning the professional fellowship and successfully being appointed an educatioal role to support and facilitate educational events and activities within the Education Committee as well as holding an educational position in another nursing professional body. The author strongly believes that only when we learn well, we can help more, and to be brave to explore further, to make greater influence on others, and together, we make change!

### A163 Designated Preoperative Assessment Clinic for Cardiac Surgery- Pathway to Enhanced Recovery After Cardiac Surgery

#### Rajan, Lekha, Mrs; Kinsella, Aisling, Mrs.

##### Mater Misericordiae University Hospital Dublin, Dublin, Ireland.

*Journal of Cardiothoracic Surgery* 2023, **18(Supp 1)**:A163

**Introduction**

Enhanced Recovery after Cardiac Surgery (ERAS) is a multimodal, transdisciplinary approach to promote recovery of patients undergoing surgery throughout their surgical journey. Introduction to ERAS was rolled out in February 2019 in the national cardiac surgery unit where 900 cardiothoracic surgeries done each year.

**Aim**

The ERAS program aims to reduce complications and promote earlier return to normal activities for our patients. It also aims to reduce the hospital length of stay, to reduce cancellations due to inadequate patient workup for surgery and therefore optimise activity.

**Methods**

A dedicated pre-op assessment clinic was set up to optimise patient assessment and workup prior to surgery. This included collecting length of stay (LOS) data, surgical site infection (SSI) rates and patient satisfaction score.

**Results**

The Post-operative LOS improved in 2019 in compared to 2017 with 20% improvement in discharge within 7 days; this has been difficult to measure in 2020 as all surgical activity was reduced during the covid 19 pandemic. Despite the pandemic impacting activities with ERAS almost 50% of patients were discharged within 7 days post-surgery. Furthermore, the introduction of pre- op decolonisation illustrated a reduction in the SSI 7.6% in 2019 to 3% in 2020. The patient feedback reported 90% of the patient and family received adequate information to prepare for surgery and discharge planning.

**Conclusion**

The introduction of a dedicated cardiac ERAS programme has shown a reduction in length of stay, reduced SSI and improved patient information, education and support by dedicated ERAS Clinical Nurse Specialist in Cardiac Surgery.

### A164 SSI Champions: Managing Surgical Site Infection Prevention Strategies Through Inter-disciplinary Network Collaboration

#### Magboo, Rosalie, Mrs; Antolin, Randolph, Mr; Arcegono, Trixia Mikaela, Ms; Basilio, Kristia, Ms; Blair, Joyce Beverly, Mrs; Sebastian, Luzviminda, Mrs; Uy, Cheryl, Mrs.

##### St Bartholomew's Hospital, London, UK.

*Journal of Cardiothoracic Surgery* 2023, **18(Supp 1)**:A164

**Objective**

Surgical site infection (SSI) is the most dominant healthcare-associated infection affecting surgical patients. A recent national survey of SSI prevention strategies demonstrated significant variation in care in cardiac surgery centres, which is also reflected locally. The aim of this project was to standardise local practices for the prevention of SSI after heart surgery.

**Methods**

A cross-department network of SSI champions has been established to facilitate the implementation of a revised protocol and standardisation of local practices for SSI prevention. A series of multidisciplinary teaching was delivered to keep the staff informed and promote adherence to the new local SSI prevention protocol. Regular audits on ten SSI prevention strategies were conducted to assess compliance. Run charts were produced to analyse trends, with annotations marking the interventions made.

Kurt Lewin’s model of unfreezing, changing and refreezing was utilised to guide the champions’ initial strategy to implement the SSI prevention protocol. Subsequently, his force field analysis framework was used to critically examine the driving and restraining factors affecting stakeholders’ acceptance of change.

**Results**

Compliance rate to each prevention strategy varied considerably between 23–100%. The main driving forces in the implementation include: visibility of the SSI champions in the clinical area, ongoing feedback on each department’s practices and collaborative working with the multidisciplinary team. Differences in patient management, quick changeover of staff and staffing shortages were seen as significant restraining factors.

**Conclusion**

There is a variable uptake of the protocol but the visibility of SSI champions has been instrumental in embedding the agreed standard local practice for SSI prevention after cardiac surgery. The challenges faced will be addressed in further PDSA cycles. Future audit will include comparison of SSI rate pre and post protocol implementation.

### A165 Introduction to the shared role of the Lung Cancer Nurse (Between two Dublin Hospitals)

#### Gallagher, Deirdre^1^, Miss; Redmond, Karen^1^, Prof; Eaton, Donna^1^, Prof; Cormican, Liam^2^, Prof; Sheridan, Nina^1^, Ms; Fitzpatrick, Tracey^1^, Ms; Brown, Rachel^1^, Ms.

##### ^1^Mater Misericordiae University Hospital, Dublin, Ireland; ^2^Connolly Hospital Blanchardstown, Dublin, Ireland.

*Journal of Cardiothoracic Surgery* 2023, **18(Supp 1)**:A165

**Background**

In Ireland there are 8 designated centres for LRAC. While an increasing number of patients with lung cancer will be referred to a LRAC, it is acknowledged that an increasing cohort of patients will be diagnosed through other referral streams straight to MDT. The shared role of the LCN was introduced in 2019. Two Dublin Hospitals are involved, and this includes the centre of excellence for Thoracic Surgery & Oncology and a non-surgical non LRAC site. The diagnostic and treatment services between these two sites have been linked since the 1980s.

**Aim**

The aim of this abstract is to evaluate the value of the role of the LCN and how the service and patients have benefited.

**Methods**

This study is a retrospective review of data across both sites. This data will inform how many patients required input from a dedicated LCN.

**Results**

The introduction of the LCN had a positive impact on the thoracic surgery service and meets the needs of this patient cohort. Across the two sites, 500 new patient referrals have been received by the LCN over a period of 22 months to date.

**Conclusion**

It could be argued that considering patient numbers, it may justify the need for a dedicated trained LCN at centres not designated to the treatment of patients with lung cancer.

### A166 A 3 case- Series Review of Intra- operative Nursing Care During Combined Navigational Bronchoscopy Cone Beam CT and Image-guided Robotic Surgery

#### Arcegono, Trixia, Ms; Colombino, Anna Maria, Ms; Jingco, Florence, Ms.

##### St. Bartholomew's Hospital, London, UK.

*Journal of Cardiothoracic Surgery* 2023, **18(Supp 1)**:A166

**Objective**

With the increasing public preference for minimally invasive thoracic surgeries, innovative approaches to conducting lung resections have been fast-rising. A novel approach of combined Navigational bronchoscopy Cone Beam CT Guided Fiducial Marker insertion and image-guided robotic-assisted lung resection had been conducted in 2020. This warranted the need for collaborative discussion within the intra- operative nursing team to establish patient safety throughout the case.

**Method**

The team analyzed a 3-case series using the seven-step Knowledge-to-Action (KTA) Process Framework by Graham, et al. (2006) to reflect on intra- operative safety and patient care during the complex surgical procedures.

**Findings**

The four main concepts emerged during the discussions were (a) patient care and safety, (b) radiation protection, (c) specialist skills proficiency and (d) collaborative team communication. Deliberations on these core topics were used to evaluate surgical outcomes, consider more cost-effective measures and standardize intra- operative nursing practice.

**Conclusion**

The increasing frequency of novel surgical approaches concurrently highlights the need for collaborative communication in ascertaining intra- operative safety and patient care. A problem-based learning approach as displayed in evidence-based practice models encourage critical thinking and active learning not only within the nursing groups but also within the wider multidisciplinary teams.

### A167 Developing the Use of Ultrasound-Guided Peripheral Cannulation in a Team of ACPS in Cardiothoracic Surgery: A Practice Development Project

#### Webb, Vicky, Mrs.

##### University Hospitals Plymouth NHS Trust, Plymouth, UK.

*Journal of Cardiothoracic Surgery* 2023, **18(Supp 1)**:A167

**Objectives**

To conduct a practice development project to design and implement an education package. This education package will introduce the skill of ultrasound-guided peripheral cannulation (USGPC) to a team of Advanced Clinical Practitioners (ACPs) in Cardiothoracic Surgery.

**Methods**

This project uses a practice development methodology as it aims to improve the clinical effectiveness of the team and patient experience of care, by teaching individual ACPs a specific, evidence-based skill. NHS Improvement recommends the use of a Plan, Do, Study, Act (PDSA) cycle to structure implementation of a proposed change (NHSI, 2018). For this project, the education package is the focus of the PDSA cycle. Each stage in the cycle employs relevant theory and methods; learning theory, facilitation, simulation and questionnaire.

**Results**

The primary outcome of the project is a revised and refined education package for USGPC. The education package was subjected to a PDSA (NHSI, 2018) methodical approach to improve its content and delivery. The questionnaire and reflection results recommend immediate changes that can be applied to the education package before future deliveries; source a new video for the audio-visual component, and elaborate on the topic content for ANTT, use of local anaesthesia and evidence-base.

**Conclusions**

Overall, this PDP has achieved the aim of developing USGPC in cardiothoracic surgery by producing and implementing an education package. Clear recommendations for future practice have been drawn from a critical discussion of the process. Further, the methodology and methods used to form a template for other similar projects within cardiothoracic surgery.
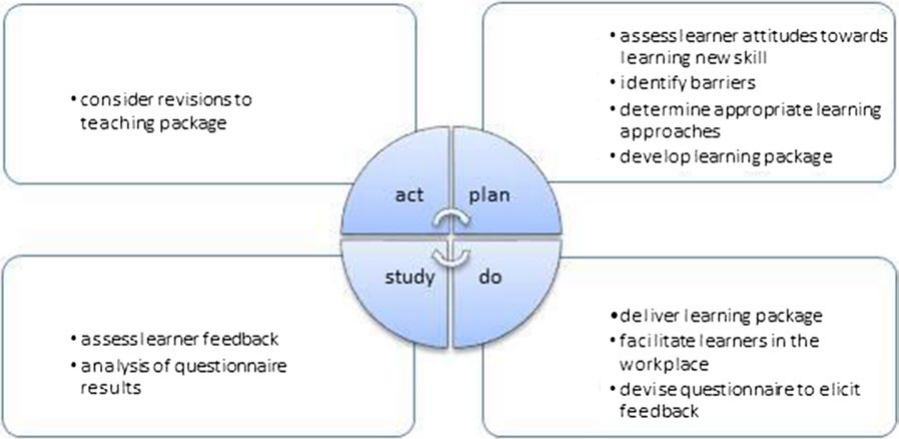


### A168 How To Set Up a Nurse—Led Small Aneurysm Clinic

#### Ahearn, Una, Miss; Harrington, Deborah, Miss; Field, Mark, Prof; Kuduvalli, Manoj, Mr; Nawaytou, Omar, Mr; Othman, Ahmed, Mr.

##### Liverpool Heart and Chest Hospital, Liverpool, UK.

*Journal of Cardiothoracic Surgery* 2023, **18(Supp 1)**:A168

**Objectives**

The nature of aortic disease means regular surveillance imaging is required pre and postoperatively, therefore volumes of patients seen in conventional consultant-led aortic clinics continually expand. In addition, referrals with incidental findings of borderline aortic aneurysms on CT scans have increased, particularly with the adoption of lung surveillance programmes. Overbooked clinics, extended waiting times and the requirement for two Consultant Surgeons in addition to three registrars in the traditional aortic clinic prompted interest in developing a nurse-led small aneurysm clinic model for patients with stable aortic disease who did not require surgical intervention.

**Methods**

The number of new patient referrals with aneurysms was audited for a year. The percentage of patients requiring surgical intervention for their aneurysm versus the percentage of patients requiring surveillance, lifestyle advice and medical management of their aneurysms was reviewed. A robust programme of nurse education and assessment was initiated. All aortic new patient referrals were initally reviewed via the usual electronic referral system by the Aortic Consultants. Patients who did not require surgical intervention were crossed referred to the Aortic Advanced Practitioner.

**Results**

Of 232 new patients who were referred with newly identified aneurysms to be reviewed in the aortic clinic, 27% required surgery and 73% required routine imaging surveillance, medical management and lifestyle advice. Our Nurse-Led Small Aneurysm Clinic, with a face to face model, opened 6 months ago and has received very positive feedback.

**Conclusions**

Despite some initial challenges our Nurse-led Small Aneurysm Clinic is very popular with staff and patients and a pragmatic solution to the burgeoning number of patients with aortic disease requiring follow-up. It has also decreased the burden in the aortic clinic, leading to a reduction in the number of Consultants required to run it.

### A169 Educating the Future Physiotherapists in Thoracic Surgery. The More the Merrier?

#### Gibb, Michelle, Miss; Chandarana, Karishma, Miss; Nakas, Apostolos, Mr.

##### Glenfield Hospital, Leicester, UK, University Hospitals of Leicester NHS Trust, Leicester, UK.

*Journal of Cardiothoracic Surgery* 2023, **18(Supp 1)**:A169

**Objectives**

The existing national shortage of clinical placements for physiotherapy students, further worsened by redeployment of qualified physiotherapists to critical care settings has led to disruption in training, and challenges in the production of a newly qualified physiotherapy workforce. Placements in Thoracic Surgery offer experience and the development of clinical decision making for patients with complex respiratory conditions in the postoperative period developing skills to work across Level 1 and Level 2 environments. We sought to evaluate the implementation of a 2:1 clinical placement physiotherapy model at a Thoracic Surgery unit in the UK.

**Methods**

From October 2020, the 2:1 clinical placement physiotherapy model was incorporated into routine Thoracic Surgery practice at our unit. 2 students were assigned to 1 educator for a 4–6-week placements (150 to 225 working hours). Feedback was obtained from both the students and the clinical educator at the end of each placement.

**Results**

Over a 12-month period, 8 students completed clinical placements on Thoracic Surgery using the 2:1 student model of training by 2 qualified physiotherapists, a 100% increase in physiotherapy students exposed to Thoracic Surgery from the previous year. All students passed the clinical placement. Qualitative feedback was recorded from both physiotherapist educators and students via confidential feedback forms (Table 1). The most common advantage identified by the students was the benefit for peer support and learning which enhanced their learning experience.

**Conclusion**

The trial of a 2:1 physiotherapy model in a busy Thoracic Surgery unit has improved clinical exposure for students with no hinderance to pass rate, promoting implementation of the model into other specialties in our Trust and evaluation of other teaching models to maximise training opportunities in the current climate.

**Table:** 

Advantages	Disadvantages
Increase in student placements offered and improved junior physiotherapist recruitment	Increased time taken to complete assessments and paperwork
Students provided peer support to each other, especially during times of self-directed practice	Less confident students felt they were being over shadowed by more confident students
80% of students reported having another student on placement felt supportive	Clinical educators felt initially hard to split time between 2 students equally
Students worked well as a pair to assist in physiotherapy projects on the ward	When students had very different learning needs the educators felt this did increase their workload

### A170 Endoscopic Harvesting Techniques are Safe and Reliable. The Oxford Experience of 9-years of Practice.

#### Djordjevic, Jasmina, Ms; Turton, Michael, Mr; Nair, Jyothi, Mrs; Eaglestone, Estelle, Mrs; Skulbedova, Nina, Mrs; D'Alessio, Andrea, Mr; Krasopoulos, George, Mr.

##### Oxford University Hospitals NHS Foundation Trust John Radcliffe Hospital, Oxford, UK.

*Journal of Cardiothoracic Surgery* 2023, **18(Supp 1)**:A170

**Objectives**

Endoscopic vein harvesting (EVH) and endoscopic radial harvesting (ERAH) for coronary artery bypass grafting is the most common harvesting technique at the Oxford Heart Centre. We are reporting the Oxford experience with EVH and ERAH, over a period of 9 years.

**Method**

Prospectively collected data of EVH and ERAH were retrospectively analysed. A subgroup analysis of 148 patients underwent postoperative CT-angiography assessment of the patency of their grafts at 3-6 months.

**Results**

2378 patients underwent CABG procedure. 2048 patients (86%) had EVH and/or ERAH. 13.5% had open vein harvesting (OVH) and 0.5% were converted to OVH. Average harvesting time per vein length is < 15 min, with negligible blood loss. ERAH was performed in 264 patients, 2 cases (< 1%) had to be converted to open technique and 17 patients had open radial harvesting. Average harvesting time for ERAH is 25 min. 4.2% of the EVH cohort developed haematomas at the harvest site (not requiring intervention) and no site infections were recorded. The OVH group had 11 (3%) cases with severe surgical site infection. 148 patients had their graft patency evaluated by CT-angiography at 6 months revealed an early occlusion rate for EVH was 8% (15 out of 187) and 2 out of 13 ERAH. The reason of occlusion was identified as competitive flow for 11 EVH and 2 ERAH. Only 4 EVH were found occluded with no obvious explanation.

**Conclusion**

Endoscopic conduits harvesting, is a safe harvesting technique with excellent reported patency rates and minimum complications rates.

### A171 Pneumatic Tourniquet in Endoscopic Radial Artery Harvesting. The Oxford Experience on How and When to do it

#### Djordjevic, Jasmina, Ms; Eaglestone, Estelle, Mrs; Turton, Michael, Mr; Nair, Jyothi, Mrs; Skulbedova, Nina, Mrs; D'Alessio, Andrea, Mr; Krasopoulos, George, Mr.

##### Oxford University Hospitals NHS Foundation Trust John Radcliffe Hospital, Oxford, UK.

*Journal of Cardiothoracic Surgery* 2023, **18(Supp 1)**:A171

**Objectives**

Endoscopic radial harvesting (ERAH) is the second most common endoscopic harvesting technique at the Oxford Heart Centre. We are reporting the Oxford experience with the use of pneumatic tourniquet for ERAH along with technical and other considerations.

**Method**

Prospectively collected data of ERAH were retrospectively analysed. The ERAH cases were divided into two groups and the results were directly compared. The technique of the tourniquet application along with tips and pitfalls were reviewed.

**Results**

247 patients with ERAH were included. ERAH procedure is done without application of systematic Heparin of 2.500 IU unless performed simultaneously with the endoscopic vein harvesting. 2 cases (< 1%) were converted to open due to the bleeding at the early phase of training, without tourniquet application. Average harvesting tome for ERAH in early experience was 50 min for first 20 cases to improve to 25 min for experienced SCP. 15 patients had ERAH with application of pneumatic tourniquet, with Heparin administration, absence of bleeding and harvesting time of 15 min. No post-operative compilations were recorded in both groups for past two years. The full indications, technique, and pitfalls in using the tourniquet for ERAH will be presented.

**Conclusion**

Endoscopic artery harvesting is a safe and reproducible technique. The use of pneumatic tourniquet during ERAH is safe when used appropriately.

### A172 Continuing Service in the Heart & Lung Transplant Ward During the Covid-19 Pandemic

#### Brennan, Michelle, Ms; Brennan, Michelle, Ms; Brown, Rachel, Mrs.

##### Mater Misericordiae University Hospital Dublin, Dublin, Ireland.

*Journal of Cardiothoracic Surgery* 2023, **18(Supp 1)**:A172

**Introduction**

At the start of the COVID-19 pandemic the National Heart & Lung Transplant ward was due to open an additional 7 new beds (increased from 7 beds previously). The new rooms along with all patients exercise bikes / foot pedals ensured a robust service at a very difficult time. Transplant patients were identified as being at high risk of acquiring the virus and having a more severe COVID-19 disease.

**Methods**

As well as opening the additional beds, we introduced new measures to keep patients and staff safe. These measures included restriction on visitors, admission risk assessments, enhanced cleaning protocols, staff vaccinations, priority vaccinations for transplant patients and weekly COVID swabbing. There was also limited staff redeployment to COVID units to ensure the ward was in the best possible position to care for immunosuppressed patients.

**Results**

We had no cross-infection of COVID-19 on the ward and maintained our service throughout the period of March 2020 to October 2021. No patients within the transplant unit swabbed positive on weekly screening.

**Conclusion**

Many wards were facing pressure on beds, we were able to double our occupancy whilst maintaining patient safety and providing a continuous service to the Transplant population.

## Pat Magee Competition

### A173 A Retrospective Review of the Prevalence of Tracheal Oesophageal Dysfunction Associated with Anomalies of the Aortic Arch

#### Bakir, Adnan, Mr; Viola, Nicola, Mr; Bharucha, Tara, Dr; Alzetani, Aiman, Mr

##### University Hospital Southampton, Southampton, UK

*Journal of Cardiothoracic Surgery* 2023, **18(Supp 1)**:A173

**Objectives**

Vascular rings cause tracheo-oesophageal compression presenting as aerodigestive symptoms. This study investigated whether therapeutic aortic arch surgical intervention alone is truly curative by detecting any persistence of aerodigestive sequelae.

**Methods**

Data were retrospectively collected for all aortic arch anomalies referred for surgery at our institution between 2005–2020. Patients were identified and categorised into a vascular ring or complex arch anomaly. Post- operative follow-up included a symptom-focused questionnaire and the most relevant outpatient clinic letters. Data were analysed with SPSS statistics.

**Results**

Our series included 117 patients; 80 (68.4%) had complex arch anomalies and no aero-digestive symptoms, 37 (31.6%) had a vascular ring and severe aero-digestive symptoms. We had an 81.2% response rate to the questionnaire. Minimal aerodigestive complications were observed in 5 complex arch anomalies (6.25%), but 16 patients with vascular rings (43.2%) had persistent aerodigestive symptoms at follow up. The mean length of follow up was 3.25 years.

**Conclusions**

Aortic arch surgery alone does not seem to completely resolve ongoing symptoms in those with preoperative tracheo-oesophageal abnormalities associated with vascular rings. Further investigations are needed to precisely identify the mechanism of persistence of symptoms in this group.

### A174 Morphology and Surgical Outcomes of Neonates with Double Inlet Left Ventricle: A Single-Centre Retrospective Study

#### Mughal, Aishah^1^, Miss; Stickley, John^2^, Mr; Crucean, Adrian^2^, Dr; Botha, Phil^2^, Mr; Khan, Natasha^2^, Ms; Seale, Anna^2^, Dr; Jones, Tim^2^, Mr

##### ^1^University of Birmingham, Birmingham, UK; ^2^Birmingham Children's Hospital, Birmingham, UK

*Journal of Cardiothoracic Surgery* 2023, **18(Supp 1)**:A174

**Objectives**

Double inlet left ventricle (DILV) is a complex form of functionally univentricular heart (FUVH). Most patients require three-stage univentricular palliation. DILV is frequently grouped with other FUVH despite morphological differences and post-surgical data fails to account for mortality before palliation. We aim to evaluate outcomes of neonates diagnosed with DILV.

**Methods**

A retrospective observational study was performed on 59 DILV patients at a tertiary paediatric cardiology unit (2006–2020) to assess cardiac morphology and outcomes. Echocardiographic imaging was reviewed and validated by a paediatric cardiologist.

**Results**

Most neonates had usual atrial arrangement (98%), two atrioventricular valves (78%) and discordant ventriculo-arterial connections (70%). Pulmonary and systemic outflow obstruction was observed in 49% and 41% of patients, respectively. 10% of patients had an unobstructed outflow. Extra-cardiac abnormalities were recorded in 9 (15%) patients. One neonate died before cardiac intervention. Of the remaining patients, 75% received cardiac intervention within 1 month of life.

Stage 1 surgery was performed in 43 (73%) neonates consisting of: a Norwood procedure (n = 21/43), systemic-to-pulmonary shunt (n = 10/43) or pulmonary artery banding (n = 12/43). Stage 2 cavopulmonary shunt was performed in 53 (90%) patients and 29 (49%) reached Fontan completion (36% awaiting Fontan) **(Fig. 1**). Stage 1 surgery had the longest hospital admission (median 19 days, IQR 13–26). Post-operative complications were recorded in 44%, 30% and 69% of patients following stage 1, stage 2 and stage 3 surgery, respectively. An unobstructed outflow was associated with lower survival (p = 0.04). Overall estimated survival at 10 years was 87%.

**Conclusion**

Whilst there is significant heterogeneity of morphologies and clinical courses amongst the DILV cohort, outcomes of neonates born with DILV are relatively favourable and should not be reported with other FUVH.
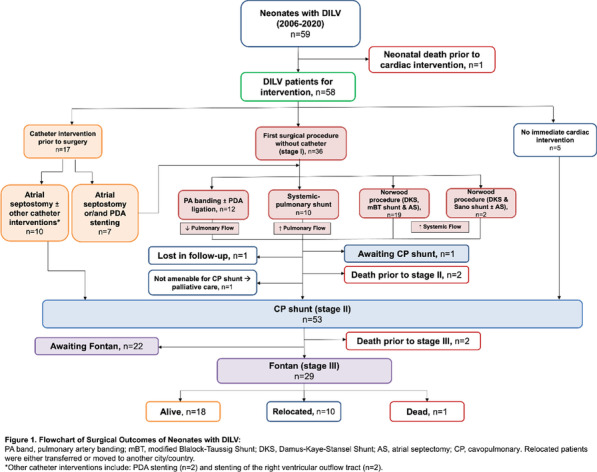


### A175 Robotic Left Upper Lobectomy with ICG Guided Wedge Resection of Lower Lobe—Movie

#### Fleet, Ben^1^, Mr; Dunning, Joel^2^, Mr

##### ^1^Lancaster Medical School, Lancaster, UK; ^2^James Cook University Hospital, Middlesbrough, UK

*Journal of Cardiothoracic Surgery* 2023, **18(Supp 1)**:A175

https://www.youtube.com/watch?v=kU8psLQ2XSA

### A176 Cardiothoracic Surgery as a Career Choice by Medical Students—Differential Response by Males and Females but not Ethnicity

#### Gnanalingham, Sathyan, Mr

##### UCL Medical School, London, UK

*Journal of Cardiothoracic Surgery* 2023, **18(Supp 1)**:A176

**Objectives**

Cardiothoracic surgery is perceived to be a Caucasian-male dominated speciality. This study explores how differences in gender, ethnicity, and disability influence medical student’s interest in considering cardiothoracic surgery as a career.

**Methods**

A 26-item online survey using Google forms was distributed amongst all 37 UK medical school’s cohorts, via social media outlets. Different factors of interest were assessed and ranked in terms of importance on a Likert scale of 1–5 (1 = not important at all, 5 = very important). Free text responses were also collated. Data was analysed using an SPSS package.

**Results**

From a total of 258 responses 62% were female and 38% were male. Males were more likely to "consider a career in cardiothoracic surgery" than females (32 Vs 19%; P < 0.001). When analysing factors that contributed to this decision, "My gender" was perceived as "not important" by more male respondents than females (78vs 42%; P < 0.0001). Furthermore, "a lack of cardiothoracic mentor of the same gender" was perceived as "important" by more females than males (24 vs 6%; P < 0.0001).

Of participants 45% were Caucasian, 44% were Asian and Asian British and 11% were other ethnic groups. There were no significant differences among the different ethnic groups, including to the question "importance of working in an ethnically diverse field" (52% Caucasians vs 57% Ethnic minorities; P = 0.7).

10% of respondents confirmed "long-standing illness or disability". Their responses to the different factors of interest did not reach statistical significance when compared with the rest of the participants.

Overall, across all participants, 73% did not feel that they had adequate exposure to cardiothoracic surgery within medical school and agreed that they would benefit from more exposure.

**Conclusions**

Our survey amongst medical students confirms that gender and not ethnicity or disability are important factors when considering a career in cardiothoracic surgery.

### A177 A Systematic Review & Meta-analysis of Post-operative Quality of Life Following Cardiac Surgery via Median Sternotomy vs Minimally Invasive Technique

#### Sinobas, Anthony^1^, Mr; Yousef, Olivia^1^, Miss; Vohra, Hunaid^2^, Mr

##### ^1^Bristol Medical School, Bristol, UK; ^2^Bristol Heart Institute, Bristol, UK

*Journal of Cardiothoracic Surgery* 2023, **18(Supp 1)**:A177

**Objectives**

Whilst there is knowledge of morbidity and mortality comparing minimally invasive (MICS) versus conventional sternotomy (CS) for cardiac surgery, such evidence does not exist for post-operative pain, psychological health, and quality of life after MICS. The aim of this study was to evaluate the above parameters with a meta-analysis of studies relevant to the subject.

**Methods**

A systematic literature search was performed of all studies comparing the post-operative pain, physical function, and psychological health after MICS versus CS. The studies included applied SF-36 and RAND-36 questionnaires to determine the above. A pooled meta-analysis was conducted to investigate differences between the two groups.

**Results**

An initial search identified 9422 papers; of which 6 studies were suitable for the final analysis. All studies included compared CS with MICS (mini-thoracotomy, thoracoscopically-assisted and robotically-assisted). Data from 857 patients in total across the 6 included studies was analysed. Pooled meta-analysis showed a relative risk of 0.24 [-0.00, 0.48; p = 0.05]; 0.14 [-0.11, 0.40; p = 0.27]; 0.32 [-0.03, 0.67; p = 0.08]; for post-operative bodily pain, physical function, and psychological health respectively.

**Conclusion**

Based on this meta-analysis, there was a statistically significant trend in favour of MICS in terms of post-operative bodily pain. We eagerly await the results of the UK Mini-Mitral trial to highlight with a greater degree of certainty the benefits of MICS over CS for mitral valvular surgery. We also suggest a greater focus on investigating differences between procedures classified as minimally invasive, with more studies comparing robotically-assisted surgery to a right minithoracotomy approach to further hone our understanding as to the best option for patients.

### A178 Early and Late Outcomes in Limited Versus Total-arch Replacement in Type A Aortic Dissection: A Meta-analysis

#### Harrington, Bertie^1^, Mr; Reynolds, Alexander^2^, Mr; Booth, Karen^3^, Ms

##### ^1^Newcastle Medical School, Newcastle, UK; ^2^Swansea Medical School, Swansea, UK; ^3^Freeman Hospital, Newcastle, UK

*Journal of Cardiothoracic Surgery* 2023, **18(Supp 1)**:A178

**Objectives**

A consensus has yet to be reached for the correct management of type A aortic dissection. A large retrospective evidence-base exists comparing limited (LAR) and total aortic arch repair (TAR). Previous meta-analysis argued the preferential use of TAR in suitably specialist centres. We aim to update this recommendation following further data publication.

**Method**

Literature searches were performed to identify studies for abstract screening. Included studies were compared to identify common outcomes, and data for such outcomes were pooled using Review Manager 5.3. Heterogeneity and publication bias were reviewed and suitable statistical adjustments were made.

**Results**

Nineteen studies underwent data-pooling, for which fifteen outcomes were analysed. Along with significantly less cardiopulmonary bypass time, LAR had significantly favourable outcomes for late neurology, low cardiac output syndrome and postoperative dialysis. All other outcomes (survival, systemic, dissection-related) were insignificant.

**Conclusions**

The retrospective data for the topic predisposes our conclusions to poor internal validity, indicating the need for randomised trials. We demonstrated increased significance in outcomes between both groups compared to the previous meta-analysis, failing to support their recommendation for TAR. Conversely, we argue that LAR should be employed in patients who are prone to stroke, AKI or have pre-existing cardiac pathology.

### A179 Outcomes of Chest Drain Management Using Only Air Leak (Without Fluid) Criteria for Removal After General Thoracic Surgery—A Drainology Study

#### Abdul Khader, Ashiq^1^, Dr; Pons, Aina^1^, Dr; Palmares, Abigail^1^, Ms; Booth, Sarah Ann^1^, Ms; Proli, Chiara^1^, Dr; De Sousa, Paulo^1^, Mr; Lim, Eric^2^, Prof

##### ^1^Department of Thoracic Surgery, Royal Brompton Hospital, London, UK; ^2^Imperial College London, Academic Division of Thoracic Surgery, Royal Brompton Hospital, London, UK

*Journal of Cardiothoracic Surgery* 2023, **18(Supp 1)**:A179

**Objective**

Chest drain management is a variable aspect of postoperative care in thoracic surgery, with different opinion for air and drain volume output. We aim to study if acceptable safety was maintained using air leak criteria alone.

**Methods**

A 9-year retrospective analysis of protocolised chest drain management using digital drain air leak cut off less than 20 ml/min for more than 6 h for drain removal in patients undergoing general thoracic surgery. We excluded patients if a chest drain was not required nor removed during admission or if patients underwent volume reduction or pneumonectomy. Withdrawal criteria was suspected bleeding or chylothorax. Postoperative films were reviewed to document post-drain removal pneumothorax, pleural effusion, and reintervention (drain re-insertion).

**Results**

Between 2012 and 2021, 1,187 patients had thoracic surgery under a single surgeon. Following exclusion and withdrawal criteria, 797 patients were left for analysis. The mean age (SD) was 61 (16) years and 383 (48%) were male. Median (IQR) duration of drain insertion was 1 (1–2) day with a median length of hospital stay of 4 (2–6) days. Post-drain removal pneumothorax was observed in 141 (17.7%), post-drain removal pleural effusion was observed in 75 (9.4%) and re-intervention (reinsertion of chest drain) required in 17 (2.1%).

**Conclusions**

Our results demonstrate acceptable levels of safety using digital assessment of air leak as the sole criteria for drain removal in selected patients after general thoracic surgery.

### A180 Heart and Lung Transplant Recipients With Pre-formed Antibodies to the Donor Can Be Safely Transplanted- A 10 Year Experience

#### Tsin Yan, Grace Ting^1^, Miss; Akbarzad-Yousefi, Arash^2^, Dr; Clark, Stephen^3^, Prof; Dark, John^4^, Prof; Parry, Gareth^3^, Dr

##### ^1^Newcastle University Medicine Malaysia, Johor, Malaysia; ^2^H&I Deparment, Newcastle, NHS Blood and Transplant, Newcastle, UK; ^3^Freeman Hospital, Newcastle, UK; ^4^Newcastle University, Newcastle, UK

*Journal of Cardiothoracic Surgery* 2023, **18(Supp 1)**:A180

**Table:** 

Lung Transplantation (Total n = 361)	With Pre-formed DSAs (n = 71), n (%)	Without Pre-formed DSA (n = 290), n (%)	p-value
Survival	52 (73%)	148 (51%)	0.005
Freedom From Bos 3	59 (83%)	252 (87%)	0.554
Lung transplant patients with pre-formed DSA (Total n = 64)	With Persistent Pre-formed DSAs (n = 11), n (%)	Without Persistent Pre-formed DSAs (n = 53), n (%)	
Survival	6 (54%)	46 (87%)	0.003
Freedom From Bos 3	8 (73%)	44 (83%)	0.086
Heart Transplantation (Total n = 199)	With Pre-formed DSAs (n = 45), n (%)	Without Pre-formed DSA (n = 154), n (%)	
Survival	31 (69%)	116 (75%)	0.541
Heart transplant patients with pre-formed DSA (Total n = 41)	With Persistent Pre-formed DSAs (n = 12), n (%)	Without Persistent Pre-formed DSAs (n = 30), n (%)	
Survival	8 (67%)	23 (77%)	0.493

Pre-formed donor-specific antibodies (DSAs) are pre-existing anti-HLA antibodies in an organ transplant recipient. Generally, pre-formed DSAs are a contraindication to transplant as they can lead to hyperacute rejection and early graft failure. Recent studies suggested that crossing low or medium-strength pre-formed DSAs do not negatively impact post-transplant outcomes. Policy now allows cardiothoracic transplantation across low or medium-strength pre-formed DSAs to improve waiting time and mortality in sensitized patients on the transplant list.

This study investigated the impact of pre-formed DSAs on 560 cardiac and pulmonary transplants in one centre. Outcomes were survival post-transplantation and freedom from bronchiolitis obliterans stage 3 (BOS 3) post-lung transplantation. 20% of lung transplant patients and 23% of heart transplant patients had pre-formed DSA (n = 71/361, 45/199). Pre-formed DSA in lung transplant patients was associated with significantly higher survival (p = 0.005) but not freedom from BOS 3 (p = 0.554). However, when pre-formed DSA persisted at one year post-lung transplantation, patients had lower survival (p = 0.003). Pre-formed DSA did not impact survival post-heart transplantation (p = 0.511).

In conclusion, cardiothoracic transplant across low to medium-strength pre-formed DSAs should be encouraged to facilitate transplantation in sensitized patients. Persistent pre-formed DSAs are an important predictor of worse survival post-lung transplantation and should be regularly monitored.

### A181 Exogenous Formaldehyde in the Exacerbation of Coronary Artery Disease. An observational Study with Meta-analysis

#### Bhaskaran, India Premjithlal^1^, Miss; Bhaskaran, Arya Premjithlal^2^, Mr; Bhaskaran, Anusuya Premjithlal^3^, Dr; Bhaskaran, Premjithlal^3^, Dr

##### ^1^Kew House School/Imperial College, London, UK; ^2^Heathfield House School, London, UK; ^3^Imperial College, London, UK

*Journal of Cardiothoracic Surgery* 2023, **18(Supp 1)**:A181

**Background**

Exposure to formaldehyde induces coronary artery disease (CAD), atherosclerosis, arrhythmia, tachycardia, ventricular or atrial fibrillation, stroke and it is linked to oxidative stress or inflammation. Higher concentrations of formaldehyde exposure can cause negative inotropic strength in the heart, sinoatrial dysfunction, which can result in bradycardia or death. The aim of this study is to identify the risk for CAD due to formaldehyde exposure and to assess the formaldehyde concentration in the atmosphere.

**Materials and methods**

PubMed, EMBASE, and ProQuest were searched, by using the terms "formaldehyde and coronary artery disease". The "Mantel–Haenszel Risk Ratio" was used for meta-analysis. For the observational component, the formaldehyde levels were obtained from a school and its peripheral areas based in west London in 17 & 8 h in 2018 & 2020.

**Results**

Overall, 204 titles or abstracts were identified from the initial search, of which full manuscripts of 91 studies were retrieved in the first phase. Later, 86 studies were excluded and five were subjected to meta-analysis. The average formaldehyde concentration across the studies ranged between 0.37 mg/m^3^ to 5.4 mg/m^3^. The risk ratio was 2.31 (95% C.I = 1.19 to 4.09) and hence for every unit (µg/m^3^) increment in the formaldehyde concentration, there was a higher risk for CAD (Fig. 1). Theformaldehydelevels were high in the morning due to the accumulation formaldehyde in the air and low in the evening in 2018. In 2020, the levels were showed almost similar pattern, but less formaldehyde in the atmosphere during the period of COVID-19 pandemic (Figs. 2–4).
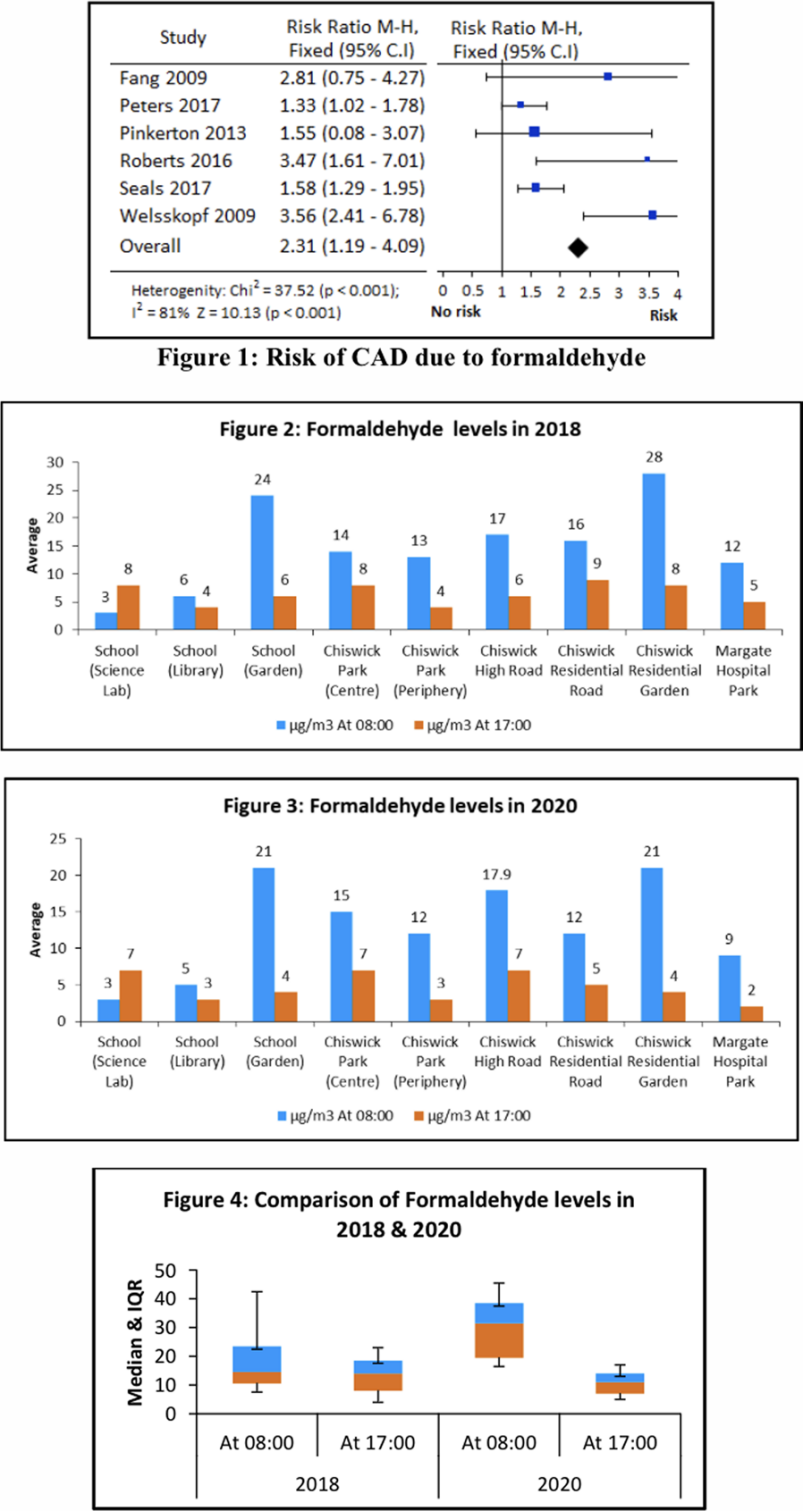


**Conclusion**

There is an association between formaldehyde exposure and CAD, including myocardial infarction and stroke. Hence a critical evaluation of electrocardiogram, echocardiogram and cardiac markers should be performed among exposed cases to prevent further complications.

### A182 Improving Access to Surgical Education via Cost-effective Interactive Live-streaming to Medical Students

#### Narang, Karamveer, Mr; Ahmadi, Navid, Dr; Hartley, James, Dr; Aresu, Giuseppe, Mr; Peryt, Adam, Mr; De Silva, Ravi, Mr; Wells, Francis, Mr; Jones, Nicola, Dr; Coonar, Aman S, Mr

##### Royal Papworth Hospital, Cambridge, UK

*Journal of Cardiothoracic Surgery* 2023, **18(Supp 1)**:A182

**Objectives**

Cancellations of operations and attempts to reduce footfall within hospitals during the COVID-19 pandemic has greatly reduced the opportunity for students to observe surgery.

Online live streaming is a novel approach to improve access to surgery and the quality of surgical education delivered to medical students. It allows students to view surgery in real-time and provided an interactive learning experience.

**Methods**

Multiple cameras were used as webcam feeds to be broadcast on a closed network such as Microsoft Teams with approval from information governance. These ranged from theatre overhead light cameras, smartphones and webcams found on trust laptops used for educational purposes.

A pre-operative seminar was arranged after the team brief during each case allowing the surgeon to interact with the students and provide small group teaching. Students were sent information about the case beforehand in order to give them an understanding of what they would be viewing on the live stream. We utilised a Bluetooth headset to then allow the surgeon to provide an interactive running commentary during the operation.

Feedback forms where used pre-and post session to gauge student’s interest in surgery and evaluate the teaching session and learning experience.

**Results**

A total of 6 live stream sessions were evaluated. 22 students attended these sessions and provided feedback.

68% (15/22) of the students said that the session was ‘very helpful’ or ‘extremely helpful’ while 4% (1/22) of students stated that the session was ‘not so helpful’ with 0% claiming it was ‘not helpful at all’. 36% (8/22) of the students claimed that these sessions had made them more likely to pursue surgery as a specialty in the future.

**Conclusions**

Interactive live streaming is highly effective using easily available, low cost technology. There are enormous potential savings with respect to travel and reducing footfall in theatre.

### A183 Biological Versus Mechanical Prosthesis for Valvulopathy in Dialysis-dependant Patients: A Meta-analysis

#### Reynolds, Alexander C.^1^, Mr; Owen, Rhiannon K.^1^, Dr; Modi, Amit^2^, Mr; Asopa, Sanjay^3^, Mr

##### ^1^Swansea University Medical School, Swansea, UK.; ^2^Sussex Cardiac Centre, Brighton, UK.; ^3^Southwest Cardiothoracic Centre, Plymouth, UK

*Journal of Cardiothoracic Surgery* 2023, **18(Supp 1)**:A183

**Objectives**

Debate continues for prosthesis choice in dialysis-dependant patients undergoing valve replacement. Biological valves have been implanted in cases of poor long-term survival, circumventing the warfarin burden. Conversely, mechanical prosthesis is indicated in those who would otherwise outlive the durability of a bio-prosthetic. Our aim is to reach consensus using the current data available.

**Methods**

Literature searches were performed to identify studies for abstract screening. Included studies were compared to identify common outcomes, and data for such outcomes were pooled using Review Manager 5.3. Heterogeneity and publication bias were reviewed, and suitable statistical adjustments were made.

**Results**

Twelve retrospective studies were included, providing sixteen outcomes for data-pooling. 5-year survival was poor in both groups, and significantly less in the bioprosthetic than the mechanical group (21.6% versus 32.6% respectively, p < 0.0001. I2 = 21%). Postoperative mortality was indifferent between both groups, alongside mediastinitis, sepsis and AF. The late incidence of stroke, gastrointestinal morbidity and venous thromboembolism was similar in both groups.

**Conclusions**

The overall survival period for the dialysis-dependant population remains poor. The retrospective data for this topic predisposes our conclusions to poor internal validity; greater long-term survival with mechanical prosthesis may be attributed to a significantly younger population, rather than survival benefit. With otherwise similar outcomes between both groups, we recommend joint decision-making in the context of case presentation and patient preference.
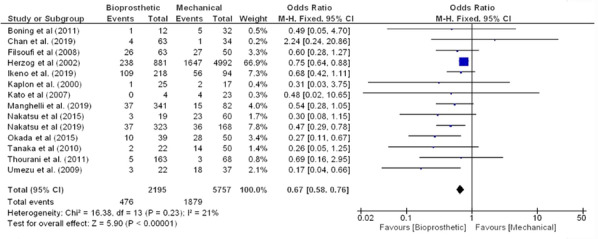


### A184 Investigating the Obesity Paradox in Cardiac Surgery

#### Ghazarians, Nareh, Miss; Walker, Antony, Mr

##### Lancaster Medical School, Lancaster, UK

*Journal of Cardiothoracic Surgery* 2023, **18(Supp 1)**:A184

**Objectives**

Obesity is associated with a number of cardiovascular risk factors. Studies have shown overweight and obese patients to have better prognosis and clinical outcomes, the obesity paradox. The aim of this study was to investigate the effect of BMI on outcomes after cardiac surgery.

**Methods**

All cardiac surgery patients at the Blackpool Victoria Hospital between January and December 2009 were retrospectively reviewed. Patients were grouped based on BMI classifications, defined by the WHO and NIH. The six groups were analysed Class III obese (BMI ≥ 40 kg/m2; n = 29; 2.6%), Class II Obese (35 ≤ BMI < 40 kg/m2; n = 68; 6.0%), Class I Obese (30 ≤ BMI < 35 kg/m2; n = 243; 21.4%), Overweight (25 ≤ BMI < 30 kg/m2; n = 496; 43.7%), Healthy weight (18.5 ≤ BMI < 25 kg/m2; n = 288; 25.4%) and Underweight (BMI < 18.5 kg/m2; n = 11; 1.0%). Pre-operative variables and post-operative outcomes were analysed using appropriate statistical methodology, with p values less than 0.05 being taken as significant.

**Results**

1135 patients were included. Obese patients were significantly less likely to receive transfusion *Χ*2(5, N = 1135) = 40.90, *p* < 0.001. There was a significant negative association between BMI and In-Hospital Mortality, with mortality decreasing with increasing BMI *Χ*2(5, N = 1135) = 16.29, *p* < 0.001. A J-shaped curve associating BMI with EuroSCORE, Later Mortality and incidence of blood transfusion could be observed.

**Conclusions**

Our study confirms the existence of an obesity paradox in cardiac surgery. This suggests a need for risk stratification methods in cardiac surgery to take BMI into account.

### A185 Short, Mid and Long Term Outcomes of Post-Myocardial Infarction Ventricular Septal Defects

#### Pengelly, Sarah, Miss

##### Cardiff University, Cardiff, UK

*Journal of Cardiothoracic Surgery* 2023, **18(Supp 1)**:A185

**Objective**

To evaluate the short/mid/long term survival of patients who underwent repair of post-myocardial infarction ventricular septal defects. To identify key factors which influence this.

**Methods**

Analysis spanned 1998–2021 covering 53 patients who underwent surgery for post-infarct ventricular septal defect at a single centre. Kaplan–Meier and chi-squared statistical analyses were conducted.

**Results**

Intra-aortic balloon pump was used in 95.2% of patients. 91.7% of ventricular septal defects were repaired with a bovine pericardium patch. 66.0% of patients also required coronary artery bypass graft surgery, with 9.4% requiring valve surgery. Arrhythmia (69.4%) was the most common complication. Overall crude in-hospital mortality was 38.9%: 38.5% for anterior and 31.0% for inferior. Overall survival at 1 year is 48.9%, 42.8% at 5 years, 30.4% at 10 years and 0.0% at 20 years. At each time point measured inferior survival probability was greater than anterior survival. Prior to 2010 in-hospital mortality was 40.0%, dropping to 37.5% from 2010 onwards. Overall survival probability at each time point was improved from 2010 onwards.

**Conclusions**

Five risk factors for mortality were identified: age 70 or higher (p = 0.004024); arrhythmia (p = 0.010658); post-operative new haemofiltration/dialysis (p = 0.046409); reason for intra-operative intra-aortic balloon pump use (p = 0.041058) for overall; and intra-aortic balloon pump use (p = 0.012432) for anterior.

### A186 Computed Tomography Scanning for Sternal Wound Infections: A Systematic Review

#### Shirke, Manasi^1^; Dominic, Cathy^1^, Miss; Nawaz, Hamza^1^, Mr; Debnath, Pradipta^2^, Mr; Sunny, Jesvin^3^, Mr; Haq, Mawiyah^4^, Mr; Harky, Amer^5^, Mr

##### ^1^; ^2^Bart's and the London School of Medicine, London, UK; ^3^University of Nottingham, Nottingham, UK; ^4^University of Central Lancashire, Preston, UK; ^5^St. George’s University of London, London, UK; ^6^Barts and the London School of Medicine, London, UK; ^7^Liverpool Heart and Chest Hospital, Liverpool, UK

*Journal of Cardiothoracic Surgery* 2023, **18(Supp 1)**:A186

**Objectives**

Sternal wound infection (SWI) is a significant risk in patients who undergo sternotomies as part of their cardiothoracic surgical procedures. Computed tomography (CT) imaging is often used to diagnose and assess sternal wound infections. Its purpose includes identifying and locating infection and any sternal dehiscence.

**Methods**

A systematic literature review across PubMed, Embase, and Ovid was performed according to PRISMA guidelines to identify relevant articles that discussed the utility of CT scanning for SWI, common features identified, patient outcomes and sensitivity/specificity. All studies discussing the role of CT imaging in sternal wound infection were included. Studies discussing both superficial and deep sternal wound infections were included. Editorials, consensus documents, commentaries, case series of less than three patients, literature reviews, and studies not in English were excluded.

**Results**

25 papers were included. 100% (n = 25) of the papers were published in peer-reviewed journals. CT scans in SWIs can be seen as a beneficial aid in diagnosing as well as determining the components of infection. Commonalities were identified such as fluid collection in the mediastinum, free gas, pleural effusions, and sternal dehiscence which point towards the presence of sternal wound infection.

**Conclusion**

CT scanning is a novel and emerging methodology for imaging in SWI and post-sternotomy complications, hence increased research is required to expand the literature on this area as well as the creation of guidelines and staging criteria for radiology professionals to identify and determine the extent of infection.

### A187 Effectiveness of a Virtual Core Surgical Training Interview Preparation Course Programme

#### Veeralakshmanan, Pushpa^1^, Miss; Panahi, Pedram^2^, Mr; Seraj, Shaikh Sanjid^3^, Dr

##### ^1^University Hospitals Birmingham, Birmingham, UK; ^2^Royal Marsden Hospital, London, UK; ^3^Walsall Hospital, Walsall, UK

*Journal of Cardiothoracic Surgery* 2023, **18(Supp 1)**:A187

**Objectives**

In the UK (UK), entry into the core surgical training (CST) programme remains a competitive process. In view of the COVID-19 pandemic, there has been an increase use of online platforms to ensure educational needs are met despite the cancellations of face-to-face courses and conferences. We developed an one-day intensive virtual CST interview preparation course and assessed the effectiveness of the course.

**Methods**

The one-day CST interview preparation course was designed, implemented and delivered virtually via Microsoft teams at an international level in November 2020. The course content were developed and delivered by core surgical trainees. The content delivered covered all the three stations of the national interview. Additionally, in view of the COVID-19 pandemic, alterations to the application and interview process were discussed in detail. All attendees were asked to complete the feedback immediately following the course.

**Results**

196 trainees attended the virtual CST interview preparation course. Over 98% of the attendees found the course to be ‘excellent’ with regards to usefulness of the course and over 99% of the attendees found the course to be ‘highly relevant’. A two-tailed paired T-test showed a statistically significant difference between pre- and post-course level of confidence for the CST interview (*T* = 9.99, *P* < 0.0001), demonstrating nearly a tenfold increase in level of confidence and preparedness for the CST interview after attending our course. Over 80% of attendees found the use of Microsoft Teams to access the course to be 'very easy', with over 85% of attendees preferring the course to be delivered virtually.

**Conclusion**

Our service evaluation of the CST interview preparation course has shown that using online platforms like Microsoft Teams for teaching can be highly effective. In view of the COVID-19 pandemic, the use of online platforms for teaching can be integrated into delivery of the surgical curriculum.

### A188 The Ability of EuroSCORE to Predict Long-term Outcomes After CABG Surgery

#### Fleet, Ben^1^, Mr; Tandon, Eisha^1^, Miss; Walker, Antony^2^, Mr

##### ^1^Lancaster Medical School, Lancastr, UK; ^2^Blackpool Victoria Hospital, Blackpool, UK

*Journal of Cardiothoracic Surgery* 2023, **18(Supp 1)**:A188

**Objectives**

The European System for Cardiac Operative Risk Evaluation (EuroSCORE), introduced in 1999 predicts in-hospital mortality for patients undergoing cardiac surgery. Many variables associated with increased surgical mortality persist post-operatively. The aim of this study was to investigate the predictive value of the logistic EuroSCORE on long-term survival after coronary artery bypass surgery.

**Methods**

Data were collected retrospectively for all patients undergoing CABG at a single centre between 1st January 2009 and 31st December 2009. Data submitted to NICOR were used for EuroSCORE and in-hospital outcomes; longer-term, all-cause mortality from NHS digital Personal Demographic Service. Low (< 3), intermediate (3–6) and high-risk (> 6) logistic EuroSCORE groups were identified and analysed using appropriate statistical methodology, with p values less than 0.05 being taken as significant.

**Results**

663 patients underwent isolated CABG procedure during the study. The 1-year, 3-year, 5-year and 10-year survival rates were 97.6%, 94.3%, 89.3% and 73.5% respectively. Kaplan Meier curves for low, intermediate and high-risk groups are shown in Fig. 1 (*p* < 0.00001). Poor left ventricular ejection fraction, serum creatinine above 200 ml, chronic pulmonary disease, extracardiac arteriopathy and pulmonary hypertension were identified as independent predictors of long-term mortality.

**Conclusions**

Our study demonstrates the logistic EuroSCORE predicted long-term outcomes following CABG surgery. This finding can inform patients of the long-term risks of CABG surgery and given the favourable results compared to percutaneous intervention, guide MDT decision making.
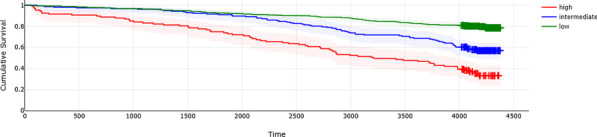


### A189 Anatomy of the Subpulmonary Stenosis in Tetralogy of Fallot: The Gateway to Eisenmenger Syndrome?

#### Patsalides, Michalis Anestis, Mr; Paterson, Scott, Dr; Spear, Michelle, Prof

##### University of Bristol, Bristol, UK

*Journal of Cardiothoracic Surgery* 2023, **18(Supp 1)**:A189

Subpulmonary stenosis resulting from the anatomical distortion observed in Tetralogy of Fallot may severely limit pulmonary arterial blood flow compromising pulmonary vasculature development and anatomy, ultimately leading to complications such as Eisenmenger syndrome.

Stenosis at subpulmonary level involves a hypertrophied antero-superior limb of the septomarginal trabeculation, antero-superior deviation of the muscular outlet septum and stenosis of the subpulmonary infundibulum of the right ventricular outflow tract. Through dissection, these structures are seen closely related, encircling the gateway to pulmonary circulation. As concluded via further literature review, if anatomically abnormal, these structures restrict pulmonary arterial blood flow. Thus, there is a in change blood flow velocity transforming laminar to turbulent flow. In combination with left-to-right shunting through a non-restrictive interventricular communication, turbulent blood flow in pulmonary arteries promotes vascular endothelial remodelling. This leads to hypertrophy of the tunica media of the pulmonary blood vessels via expression of vascular endothelial growth factor further increasing pulmonary vascular resistance, a hallmark of Eisenmenger syndrome.

Consequently, by understanding the anatomy of the subpulmonary infundibulum in Tetralogy of Fallot, its effect on pulmonary blood flow can be quantified, paving the way for using anatomical configuration and pulmonary blood flow as prognostic tools for the development of Eisenmenger syndrome.

### A190 Education Post-COVID-19; Moving Towards Hybrid Practical Skills Workshops and Online Learning

#### Raja, Momna^1^, Ms; Modi, Sahil^1^, Mr; Dhuga, Yasmin^1^, Ms; Ahmed, Ishtiaq^2^, Mr

##### ^1^Brighton and Sussex Medical School, Brighton, UK; ^2^Royal Sussex County Hospital, Brighton, UK

*Journal of Cardiothoracic Surgery* 2023, **18(Supp 1)**:A190

**Objectives**

Since the COVID-19 pandemic, we have shifted from traditional classroom-based education to e-learning. Although this pivot has made learning more accessible, surgical education has historically required practical hands-on teaching with immediate feedback. Previously, remote workshops have been critiqued due to lack of interaction and difficulty obtained adequate camera angles. In this pivotal study, we aim to assess the quality of teaching delivered in a Hybrid format.

**Methods**

As a part of the International Cardiothoracic Conference hosted by Brighton and Sussex Medical School’s cardiothoracic surgical society, 24 students were offered the opportunity to attend hybrid aortic valve replacement workshops (virtual or in-person). Virtual stations were delivered in a ‘grab-and-go’ box, including a laptop, camera, and prosthetic material. Attendees received instruments regarding the set-up, software, and the session programme a week before the workshop.

**Results**

65% of questionnaires were returned. The feedback was extremely positive with attendees rating it a 9.7 out of a total of 10. 100% of the attendees in the virtual station found the virtual set-up "easy" to use and were "extremely satisfied". When asked to rate it out of ten, scores of 9.6, 9.6 and 9.4 were given for the overall platform, audio, and video.

**Conclusion**

Despite the challenges and limitations, the future of surgical education should include such hybrid events. They are an excellent alternative with the optimal blend of in-person and remote opportunities, which can be catered to the attendee’s preference. Simulations and virtual wetlabs enable trainees to develop there purposeful practice from the comfort of their own homes without warranting concerns regarding quality of teaching.

### A191 Single Centre's Experience; Propensity Matched Analysis of Minimally Invasive vs Full Sternotomy Mitral Valve Repair

#### Raja, Momna^1^, Ms; Ahmed, Ishtiaq^2^, Mr

##### ^1^Brighton and Sussex Medical School, Brighton, UK; ^2^Royal Sussex County Hospital, Brighton, UK

*Journal of Cardiothoracic Surgery* 2023, **18(Supp 1)**:A191

**Objective**

In the past two decades, interest in minimally invasive mitral valve repair (MIMVr) has grown due to its reported benefits of less surgical trauma, better cosmesis, and faster recovery times. In this retrospective observational study, we aim to evaluate the safety and effectiveness of the new minimally invasive mitral valve repair [via mini-thoracotomy] in comparison with conventional full sternotomy (FSMVr).

**Methods**

Between 2014 and 2020, data was retrospectively collected for 254 patients (51 MIMVr and 203 FSMVr) who underwent mitral valve repair at a single institute. All pre- and post- hospital data was collected using the trust’s internal data base (Panda) and MetaVision [iMDsoft]. 45 well-matched pairs were identified using propensity score matching in R. This was done to reduce the effect of confounding baseline patient demographics on outcomes.

**Results**

After matching, there were no significant differences in baseline characteristics. The repair rate was 100%(n = 45) post-operatively in both groups but MIMVr had lower 30-day mortality [0%(n = 0) vs 2.2%(n = 1), p > 0.9]. MIMVr patients had longer cardiopulmonary bypass time(CBP) and total cross-clamp time(CCT); 75 min(95% CI; 57,88) and 38 min(95% CI; 25,49) respectively [p < 0.01 for both]. But there was a trend towards less need for transfusions [42%(n = 19) v 49%(n = 22), p = 0.66]. Moreover, they spent less time in ICU [74(50, 122) vs 89(55, 110), p > 0.9] and hospital [5(4, 8) vs 7(6, 9), p < 0.1]. There were no significant differences in post-operative freedom from mitral regurgitation, arrhythmias, pulmonary, infection and renal complications.

**Conclusion**

MIMVr is a safe alternative to FSMVr. Despite prolonged CBP and CCT times, it was associated with lower mortality, fewer blood product transfusions, reduced length of ICU and hospital stay. There were no significant differences in post-operative complications such as stroke, infections, pulmonary complications, or arrhythmias.

### A192 Promoting Interest in Cardiac Surgery; Integration of Specialised Skills Workshops in the Undergraduate Medical School Curriculum

#### Dhuga, Yasmin^1^, Miss; Raja, Momna^2^, Ms; Modi, Sahil^1^, Mr; Ahmed, Ishtiaq^3^, Mr

##### ^1^Brighton and Sussex University Hospital, Birghton, UK; ^2^Brighton and Sussex Medical School, Brighton, UK; ^3^Royal Sussex County Hospital, Brighton, UK

*Journal of Cardiothoracic Surgery* 2023, **18(Supp 1)**:A192

**Objectives**

In the UK, less than half of the medical students report getting teaching around cardiothoracic surgery, with only 10% being exposed to clinical placements. Due to poor satisfaction, we are seeing a downward trend in students wishing to pursue a career in cardiac surgery. In this study, we aim to assess if WETLAB workshop encourages the engagement of medical students in cardiac surgery.

**Methods**

We hosted a WETLAB workshop, led by a consultant cardiac surgeon. Attendees were taught how to carry out an end-to-side anastomosis on animal tissue. We used pre- and -post exposure questionnaires composed of 10 items to assess the ability of a WETLAB workshop to improve undergraduate understanding and interest in cardiac surgery. The questionnaire included questions around students’ interest in the speciality, previous exposure to the speciality and whether students want to pursue a career in the speciality.

**Results**

Out of the 12 attendees, 10 completed both the pre- and post-questionnaire. The WETLAB was well received with the overall satisfaction of 9.64 out of 10. It significantly increased attendees interest in cardiac surgery (z = -0.06, p = 0.01) but did not show any significant difference in encouraging them to pursue surgery. On quantitative analysis, 100% of attendees agreed that specialised practical workshops should be incorporated in the medical school curriculum and that if the opportunity arises, they will attend another in the future.

**Conclusion**

Specialised practical workshops have the potential to play a significant role in the medical school curriculum in order to enhance exposure to the field of cardiac surgery. This may lead to a positive impact on the number of doctors wishing to pursue this speciality in the future.

### A193 Online Careers Sessions Integrating and Exploring Work-life Balance in Heart & Lung Specialties

#### Narang, Karamveer, Mr; Ahmadi, Navid, Dr; Asemota, Oghogho, Dr; Ahmadi, Faisal, Dr; Purmessur, Rushmi, Miss; Peryt, Adam, Mr; Aresu, Giuseppe, Mr; Jones, Nicola, Dr; Coonar, Aman S, Mr

##### Royal Papworth Hospital, Cambridge, UK

*Journal of Cardiothoracic Surgery* 2023, **18(Supp 1)**:A193

**Objectives**

Restrictions due to COVID have decreased elective opportunities for medical students and foundation doctors to gain an insight into the work and life of a consultant. Work-life balance is increasingly recognised as important for a sustainable and rewarding career.

Our objective was to give delegates an exposure to both the professional and personal life of consultants and trainees in the clinical specialties in our unit and to evaluate delegates’ thoughts surrounding work life balance and how that would impact their career choices.

**Methods**

Consultants in cardiac and thoracic surgery, respiratory medicine, cardiology, and intensive care gave TED style short talks covering their professional work and work-life balance. Cardiothoracic trainees delivered talks about their work and life.

The event was run using ‘GoTo’ Webinar and questionnaires were completed by delegates integrated into the same platform.

**Results**

48 delegates attended the virtual taster afternoon ranging from medical students to foundation doctors with the majority of delegates interested in cardiothoracic surgery (73%).

79% of delegates reported an increased interest in pursuing their specialty of choice. 69% of delegates answered 5/5 or 4/5 when rating how important work-life balance would be to them when considering their future surgical specialty whilst only 4% of delegates stated that work-life balance would not be important at all (rated 1/5).

59% of delegates felt that the best work-life balance would be achieved as a consultant compared to other grades of doctors such as foundation, senior house officers and registrars. Only 12.5% of delegates rated work life balance among junior doctors as good (5/5 rating).

**Conclusions**

Work-life balance is an important factor in career choice. Historically this was set aside or otherwise considered negatively. Further research into which aspects of work-life balance make particular specialties more popular will help to optimise recruitment.

### A194 Impact of Covid-19 and Doctor-led Online Teaching Sessions on the Confidence of Medical Students

#### Hawwash, Nadin^1^, Miss; Joseph, Daniella^1^, Miss; Krishnamoorthy, Bhuvaneswari^2^, Dr; Hashmi, Syed Faisal^3^, Mr

##### ^1^University of Manchester, Manchester, UK; ^2^Edge Hill University, Ormskirk, UK; ^3^Manchester University NHS Foundation Trust, Wythenshawe Hospital, Manchester, UK

*Journal of Cardiothoracic Surgery* 2023, **18(Supp 1)**:A194

**Objectives**

The COVID-19 pandemic initially halted medical school teaching in March 2020. We aim to explore the level of confidence medical students have with learning through online case-based clinical teaching.

**Methods**

We performed a cross-sectional study of students studying at medical school in the UK (UK). Eight doctor-led teaching sessions took place on the online Zoom platform covering general and specialty medicine topics organised by The Teaching Clinic Society.

**Results**

Overall, 82 participants completed the feedback forms, including students from various institutions in the UK. 71.4% of students across the series strongly agreed that the teaching was relevant to their medical school education. On average confidence in each topic increased by 37.8% after attending the series. This was extremely statistically significant (t = 16.4999, p < 0.0001, 95% CI = -2.45, -1.92). Overall, 88.6% of attendees really liked online learning and 22% of attendees were neutral towards learning online. On average, 66.2% prefer face-to-face learning as opposed to online learning (22.8%).

**Conclusion**

In conclusion, our online case-based teaching sessions were not only shown to be relevant to the medical school curriculum but also significantly improved the confidence of medical students. Nonetheless, most students prefer traditional methods of learning.

### A195 The Effect of Residential Postcode on Outcomes Following Cardiac Surgery

#### Tandon, Eisha^1^, Miss; Fleet, Ben^1^, Mr; Walker, Antony^2^, Mr

##### ^1^Lancaster Medical School, Lancaster, UK; ^2^Blackpool Victoria Hospital, Blackpool, UK

*Journal of Cardiothoracic Surgery* 2023, **18(Supp 1)**:A195

**Introduction**

A postcode lottery is defined as the unequal provision of services such as healthcare and education based on geographical location. This is an under-researched area yet is integral to understanding the healthcare inequalities that exist and the factors influencing them. The aim of this study is to investigate the potential impact of residential postcode on post-operative outcomes following cardiac surgery.

**Methods**

Data were collected retrospectively for all cardiac surgical patients at a single centre between 1st January 2009 and 31st December 2009. Data submitted to NICOR was used to identify patient demographics, EuroSCORE and in-hospital outcomes. Subjects were grouped according to four residential postcodes BB (Blackburn), FY (Blackpool), PR (Preston), and LA (Lancaster). Pre-operative surgical risk and post-operative outcomes were compared between the different groups using appropriate statistical methodology, with p values less than 0.05 being taken as significant.

**Results**

882 patients were included in the study. We identified significant differences between the age, gender, nature of surgery, redo cardiac procedures and proximity of surgery to recent myocardial infarction between the postcode groups. The postcodes were controlled for overall EuroSCORE. Producing Kaplan–Meier curves demonstrated no significant difference between the survival rates for the different regional postcodes with *p* = 0.7870 (Figure One).

**Discussion**

Differences in pre-operative risk factors between the different postcode groups did not translate into differences in overall EuroSCORE or post-operative mortality. More work is needed to localise these findings according to more specific measures of socioeconomic status.

Figure One: Kaplan Meier survival curves following cardiac surgery according to patient’s residential postcode.
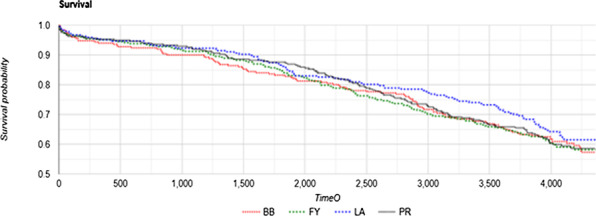


### A196 Outcomes Following Tracheostomy in Post-cardiac Surgical Patients

#### Wong, Qing Ning^1^, Ms; Avtaar Singh, Sanjeet Singh^2^, Mr; Buchan, Keith^2^, Mr

##### ^1^University of Aberdeen, Aberdeen, UK; ^2^Aberdeen Royal Infirmary, Aberdeen, UK

*Journal of Cardiothoracic Surgery* 2023, **18(Supp 1)**:A196

**Background**

Tracheostomies in post-cardiac surgery patients are performed for many reasons and are associated with poor prognosis. We investigated the overall mortality and tracheostomy related complications in a cohort of patients at a tertiary referral centre.

**Methods**

A retrospective review of 40 patients was conducted at a cardiac surgery unit between 1st August 2016 to 31st August 2021. There were 40 post-cardiac surgery patients who underwent tracheostomies during this period. Their demographic information, comorbidities, pre- intra- and post-operative status, and prospective follow-ups were interrogated from the electronic medical records and patient database. The outcomes of interest included in-hospital mortality and all-cause mortality with up to 5 years follow-up.

**Results**

The in-hospital death in post-cardiac surgery tracheostomy patients’ was 60%. The overall mortality was 70%[WQN(1] with up to 5 years follow-up. Of the in-hospital deaths, 16.7%(n = 4) had experienced tracheostomy-related complications 50%(n = 2) succumbed to them. The only statistically significant finding was the type of presentation (emergency vs urgent vs elective, p = 0.014). There were no other statistically significant differences between the preoperative and intraoperative variables between those with in-hospital mortality and patients who were discharged.
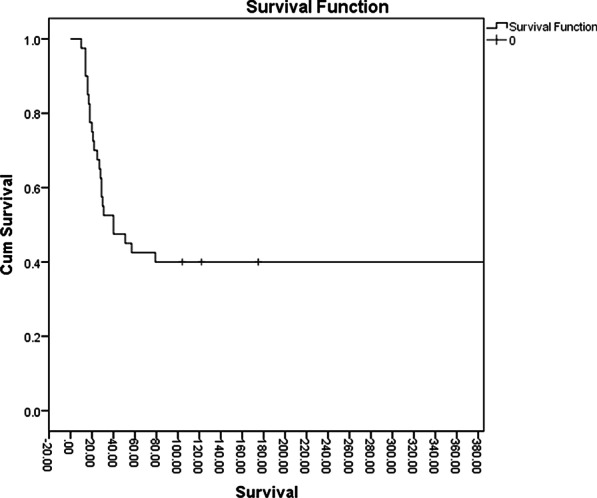


**Conclusion**

Post-cardiac surgery tracheostomy is associated with a high inpatient mortality rate. Despite the limited numbers, several trends were noted that were potential risk factors for post-tracheostomy mortality. Decisions for tracheostomy insertion in these patients should therefore be guided by a multidisciplinary team discussion as directed by the National Tracheostomy Safety Project guidelines.

### A197 Analysis of Post-operative Intensive Care Stay Length in Coronary Artery Bypass and Valve Surgery

#### Dilworth, Joseph Michael^1^, Mr; Doddakula, Kishore^2^, Mr

##### ^1^University College Cork, Cork, Ireland; ^2^Cork University Hospital, Cork, Ireland

*Journal of Cardiothoracic Surgery* 2023, **18(Supp 1)**:A197

**Objectives**

Given oversubscription of Irish ICUs, knowledge of risks for prolonged stay is key. Here, we examine the relationship between valve surgery, CABGs, EuroSCORE II (ESII), cardiopulmonary-bypass time (CPBT) and cross-clamp time (XCT) and ICU-LOS.

**Methods**

A retrospective census was taken of 833 patients undergoing CABG and/or valve surgery between 01/12/2017–31/12/2019. Kruskal-Wallace tests, Spearman correlation, receiver operating characteristics (ROC) and multiple linear regression were employed as appropriate.

**Results**

Significant differences existed between valve vs 2 valve surgeries (1.78 vs 3.91 days, p = 0.022) but not between valve + CABG vs 2 valve (2.75 vs 3.7, p = 0.462). Neither graft amounts (p = 0.101), nor specific valves operated on (p = 0.177) were significant. For ICU-LOS > 5 days, ESII’s AUC = 0.690 (p < 0.0005). XCT and CPBT correlated with ICU-LOS (rs = 0.168, rs = 0.131 respectively, p < 0.0005). Multiple linear regression predicting loge(ICU-LOS) achieved R^2^ = 0.112 (p < 0.0005). Significant factors were XCT (p < 0.0005), age (p = 0.017), and peripheral arteriopathy (p = 0.011). ESII approached significance (p = 0.056). Surgery type was insignificant (p = 0.263).

**Conclusion**

ESII’s weak-to-fair predictive ability and variables in this score achieving significance in multivariate analysis implies its weighting is not suited to ICU-LOS. XCT is superior to CPBT in ICU-LOS prediction, independent of pre-operative characteristics. Given the low R^2^, prolonged ICU-LOS was largely unpredicted, and further risk investigation and investment in capacity is recommended.

### A198 Investigating the Role of Small Nucleolar RNAs (snoRNAs) as an Early Genetic Marker of Future Adverse Cardiovascular Events

#### Kumar, Ujjawal, Mr; Hamilton, Russell, Dr

##### Department of Genetics, University of Cambridge, Cambridge, UK

*Journal of Cardiothoracic Surgery* 2023, **18(Supp 1)**:A198

**Objectives**

The PROSPER (**PRO**spective **S**tudy of **P**ravastatin in the **E**lderly at **R**isk) study identified single nucleotide polymorphisms (SNPs) associated with cardiovascular deaths. We aimed to characterise the SNPs at locus 14q32, investigate their effects on the region’s snoRNAs and the suitability of these snoRNAs as a genetic marker in individuals at risk of future cardiovascular events.

**Methods**

We filtered the published SNPs identified in our locus of interest. SNPs associated with more than one type of cardiovascular event were identified and then mapped to the genetic locus. Computational tools from the ViennaRNA suite were used to compare the variation in strandedness within the genetic locus in the wild-type gene and compared to the SNP. Using the SimRNA package and RNA contact prediction techniques, we predicted and compared the 3D snoRNA structures in the absence and presence of the SNPs, as well as investigating potential RNA–protein complexes.

**Results**

We found that these snoRNAs bind to fibrillarin, a methyltransferase, integral to nucleolar remodelling and a component of the cellular response to stresses such as chronic hypertension. The 14q32 SNPs identified by PROSPER overlap significantly with the snoRNAs, suggesting effects on snoRNA structure. We found significant differences in the strandedness of the genetic locus’ RNA between the SNP and the wild-type gene, which results in significant changes in 3D snoRNA structure with potentially drastic changes in fibrillarin binding, complex formation and function.

**Conclusions**

SNPs at the 14q32 locus lead to significant changes in snoRNA structure as well as aberrant fibrillarin complex formation and function, which may result in pathological intracellular responses to cellular stresses. Genetic screening offers the ability to potentially identify those at high risk of future adverse events and who have the most to gain from early therapy. They are also potential targets for specific genetic therapies.

### A199 Pat Magee Prize Winner—Assessing the Accuracy and Bias of Digital Symptom Checkers in Diagnosing and Triaging Myocardial Infarction Patients: Cross-sectional Study

#### Wallace, William, Mr; Chan, Calvin, Mr; Acharya, Amish, Mr; Hanna, Lydia, Ms; Normahani, Pasha, Mr; Chidambaram, Swathikan, Mr; Sounderajah, Viknesh, Mr; Darzi, Ara, Prof

##### Imperial College London, London, UK

*Journal of Cardiothoracic Surgery* 2023, **18(Supp 1)**:A199

**Objective**

To assess the accuracy of commercially available symptom checkers (SCs) in diagnosing and triaging patients presenting with myocardial infarctions (MI).

**Methods**

In this retrospective diagnostic accuracy study, SC accuracy was assessed by inputting key symptoms and biodata of 100 consecutive annonymised MI patients from a tertiary coronary intervention centre. Through a systematic search, eight SCs were identified and included. Patient biodata and presenting symptoms were inputted into each SC; outputted diagnoses and triage advice were recorded. Outcomes included (1) diagnostic accuracy as defined by SCs outputting MI as the primary diagnosis (D1), or one of the top three (D3), or top five diagnoses (D5) and (2) triage accuracy as defined by SCs outputting urgent treatment recommendations.

**Results**

Overall D1 accuracy was 48 ± 31% and varied between SCs (range: 6–85%). D3 and D5 accuracy were 73 ± 20% (34–92%) and 79 ± 14% (63–94%), respectively. Overall triage accuracy was 83 ± 13% (55–91%). 24 ± 16% of atypical cases had a correct D1. Atypical MI D3 and D5 accuracy were 44 ± 21% and 48 ± 24% respectively and were significantly lower than accuracy with typical MI cases (p < 0.01). Atypical MI triage accuracy was also significantly lower than typical cases (53 ± 20% versus 84 ± 15%, p < 0.01). D1 accuracy for atypical female MI cases was 10 ± 11%. Female atypical cases had significantly lower diagnostic and triage accuracy than typical female MI cases (p < 0.01).

**Conclusions**

Symptom checker accuracy for correctly diagnosing an MI was generally low. 17% of cases were under-triaged. Accuracy varied between symptom checkers: patients who presented with atypical symptoms tended to be under-diagnosed and under-triaged, especially if female. Thus, there is potential gender bias. This study, therefore, raises questions regarding symptom checker improvement, safety, and regulation.
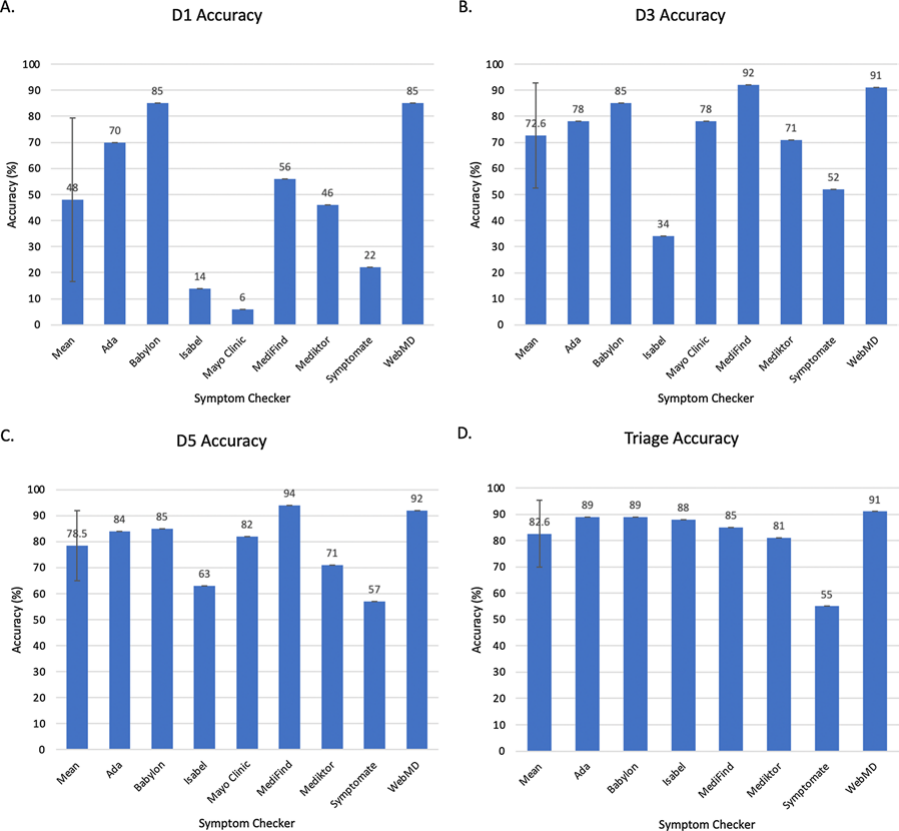


### A200 The Utility of a Half-day Practical Workshop in Improving Medical Student Perceptions and Exposure to Cardiothoracic Surgery (CTS)

#### Badran, Abdul^1^, Dr; Shah, Owais^1^, Dr; Badran, Dania^2^, Dr

##### ^1^University Hospital Southampton, Southampton, UK; ^2^Imperial College London, London, UK

*Journal of Cardiothoracic Surgery* 2023, **18(Supp 1)**:A200

**Objectives**

Undergraduate interest in cardiothoracic surgery (CTS) has stayed the same or declined over the years. Factors such as a lack of exposure in undergraduate curricula coupled with negative perceptions of the specialty are likely contributing factors.

**Methods**

We designed and delivered a hands-on half-day cardiac surgical skills course in a large medical school with the aim of providing exposure to and increasing medical student interest in the specialty. Pre and post-workshop questionnaires were utilized to investigate student perceptions of CTS and self-reported understanding and confidence in performing various cardiac surgical skills.

**Results**

There was a total of 11 attendees. All agreed that CTS involved creative/skilful surgery and being a rewarding career choice. Some negative perceptions of CTS included it being considered highly stressful (18%), a female unfriendly specialty (27%) and involving a hostile training environment (27%). Delegate self-reported understanding and confidence in performing cardiac dissection, coronary anastomosis, aortotomy closure and knot tying all increased significantly post-workshop (p < 0.05). Number of years of study did not correlate with improvement in technique (90% of delegates either strongly agreed or agreed to the statement that they were more likely to pursue a career in CTS after attending this event. All but one delegate strongly agreed that the course had positively impacted their views of CTS.

**Conclusions**

Here we demonstrate that an easily reproducible half-day practical workshop can be utilized to not only improve undergraduate perceptions of the specialty but by providing hands-on exposure, improve self-reported confidence and understanding of basic cardiac surgical skills.

## Thoracic Benign

### A201 Outcome of Emergency Lobectomy Under Extracorporeal Membrane Oxygenation (ECMO) Support in Patients with Severe COVID-19 Disease

#### Schweigert, Michael^1^, Prof; Almeida, Ana Beatriz^1^, Mrs; Dubecz, Attila^2^, Prof; Spieth, Peter^3^, Prof; Gama de Abreu, Marcelo^4^, Prof; Kellner, Patrick^1^, Dr

##### ^1^University Hospital Schleswig–Holstein, Kiel, Germany; ^2^Klinikum Nuremberg, Nuremberg, Germany; ^3^University Hospital Dresden, Dresden, UK; ^4^Cleveland Clinic, Ohio, USA

*Journal of Cardiothoracic Surgery* 2023, **18(Supp 1)**:A201

**Objective**

Not much is known about the results of non-elective anatomical lung resections in COVID-19 patients put on ECMO. Aim of this study is to analyze the outcome of emergency lobectomy under ECMO support in patients with acute respiratory failure due to severe COVID-19 disease.

**Methods**

All COVID-19 patients undergoing emergency anatomical lung resection with ECMO support at a German university hospital were included into a prospective database. The university hospital serves as only ECMO center for a population of approximately 2 million people in one of Germany´s most severely affected regions. Study period was 01.04.2020 to 30.04.2021 (first, second and third wave in Germany). Patients characteristics, indications for surgery, clinical course and outcome were analyzed.

**Results**

A total of 9 patients (median age 61 years, IQR 10 years) were included. There was virtually no pre-existing comorbidity (Median Charlson Score of Comorbidity 0.2). The mean interval between first positive COVID test and surgery was 21.9 days. Clinical symptoms at the time of surgery were sepsis (9/9), respiratory failure (9/9), acute renal failure (5/9), pleural empyema (5/9), lung artery embolism (4/9) and pneumothorax (2/9). Mean ICU and ECMO days before surgery were 15.4 and 6, respectively.

Indications for surgery were bacterial superinfection with lung abscess formation and progressive septic shock (7/9) and abscess formation with massive pulmonary hemorrhage (2/9). All patients were under veno-venous ECMO with femoral-jugular configuration. Operative procedures were lobectomy (8) and pneumonectomy (1). Weaning from ECMO was successful in 4/9. In-hospital-mortality was 5/9. Mean total ECMO days were 10.3 ± 6.2 and mean total ICU days 27.7 ± 9.9. Mean lengths of stay was 28.7 ± 8.8 days.

**Conclusion**

Emergency surgery under ECMO support seems to open up a perspective for surgical source control in COVID-19 patients with bacterial superinfection and localized pulmonary abscess.

### A202 Total Pneumonectomy for Pulmonary Gangrene

#### Schweigert, Michael^1^, Professor; Almeida, Ana Beatriz^1^, Mrs; Witzigmann, Helmut^2^, Prof; Dubecz, Attila^3^, Prof; Stein, Hubert^3^, Prof

##### ^1^University Hospital Schleswig–Holstein, Kiel, Germany; ^2^Städtisches Klinikum Dresden, Dresden, Germany; ^3^Klinikum Nuremberg, Nuremberg, Germany

*Journal of Cardiothoracic Surgery* 2023, **18(Supp 1)**:A202

**Objective**

Necrotizing pulmonary infections resulting in devitalization of an entire lung are devastating conditions with excessive mortality. Aim of this study is to shed light on the role of total pneumonectomy for infectious lung gangrene.

**Patients and Methods**

In a retrospective multi-center study from a prospective database the outcome of non-elective total pneumonectomy for infectious lung gangrene was analyzed at 6 centers in Germany, Spain and the UK.

**Results**

There were 132 patients. Median age was 58 years (IQR 18,5). Mean Charlson score of comorbidity was 2.8 (SD 2.54). Surgical procedures were total pneumonectomy (23), lobectomy (91) and segmentectomy (18). ECMO was used for 11 patients (8 lobectomy, 3 pneumonectomy). There were no significant differences in age, comorbidity and mortality (3/23 vs. 14/109; OR 1.02, 95% CI: 0.27–3.88, p = 0.98) between the pneumonectomy and non-pneumonectomy group. Preoperative respiratory failure (12/23 vs. 30/109; OR 2.87, 95% CI: 1.14–7.20, p = 0.02), pleural empyema (20/23 vs. 50/109; OR 7.87, 95% CI: 2.21–28.03, p < 0.01), sepsis (19/23 vs. 63/109; OR 3.45, 95% CI: 1.11–10.88, p = 0.03) and acute renal failure (6/23 vs. 11/109; OR 3.14, 95% CI: 1.03–9.64, p = 0.04) were significantly more common in the pneumonectomy group. Charlson Score > 3 (15/63 vs. 2/69; OR 10.47, 95% CI: 2.29–47.93, p < 0.01) and sepsis (17/70 vs. 1/62; OR 18.07, 95% CI: 2.32–140.85, p < 0.01) were associated with significant higher odds for mortality. Multivariate analysis identified preop. sepsis, pleural empyema and persistent air leak but not the extent of resection as significant risk factor for higher mortality.

**Conclusions**

In non-elective surgery for infectious lung gangrene mortality is not determined by the extent of pulmonary resection but by the burden of pre-existent comorbidity and the appearance of sepsis and septic complications. Total pneumonectomy is a life-saving option for patients with infectious gangrene of an entire lung.

### A203 Revolutionizing Surgical Side Infection (SSI) Surveillance with Personalized Digital Patient Follow-up in Thoracic Surgery

#### Mayer, Nora, Dr; Alwis, Shehani, Ms; Rochon, Melissa, Mrs; Brown, Clare, Mrs; Birdsall, Donna, Mrs; Asadi, Nizar, Mr

##### Royal Brompton & Harefield Hospitals, Part of Guy`s and St. Thomas NHS Foundation Trust, Department of Thoracic Surgery, London, UK

*Journal of Cardiothoracic Surgery* 2023, **18(Supp 1)**:A203

**Objectives**

SSI is the most costly healthcare associated infection and occurs within 30 days of surgery. Short hospital stay and remaining drains specifically expose thoracic surgical patients to SSI. Feasibility studies indicate positive patient experiences using mobile technology for wound monitoring. Aim of our study is to introduce a personalized digital SSI surveillance solution.

**Methods**

Between November 2020 and July 2021, 158 patients were added to the progressive web app (ISLA Health LTD). Wound photos were taken at discharge by medical staff (**Fig. 1A**) and uploaded 7 days after discharge by the patient (**Fig. 1B**). Patient response, satisfaction and avoided travel distance were used as early outcome measures.

**Results**

Patient response was 42% (N = 67 submissions). 3 patients (6.8% app user, 1.8% in total) were diagnosed with conservatively manageable SSI. 1985 km travel distance were avoided (**Fig. 1C**). 44 patients (66%) answered the satisfaction evaluation. None of the patients expressed concerns about sharing anonymised visual information online and 93% preferred uploading a wound photo (**Fig. 1D**) to describing the wound over the phone. 77.2% of the patients found the platform easy to use and for 88% the photo upload was unproblematic.

**Conclusion**

The digital SSI surveillance solution was successfully implemented with good response and high satisfaction rating.

### A204 A Multi-lesional Analysis of DIPNECH Lesions Over 6 Years – Should we Routinely Imaging These Patients?

#### Khor, Bo, Mr; Patel, Akshay, Mr; Shah, Tahir, Dr; Kalkat, Maninder, Mr; Hughes, Simon, Dr

##### Queen Elizabeth Hospital, Birmingham, Birmingham, UK

*Journal of Cardiothoracic Surgery* 2023, **18(Supp 1)**:A204

**Objective**

Diffuse Idiopathic Pulmonary Neuroendocrine Cell Hyperplasia (DIPNECH) is a rare disease often associated with carcinoid tumours, and is characterized by a diffuse proliferation of pulmonary neuroendocrine cells of the airway mucosa. The mainstay of diagnosis and follow-up in this condition is imaging-driven usually with computed tomography (CT). However, the optimal follow-up imaging intervals in DIPNECH patients are largely unknown. We conducted a multi-lesional analysis of DIPNECH patients and in particular the volumetric changes of DIPNECH lesions over a 6-year period.

**Methods**

We retrospectively analysed yearly CT scans from 22 patients with pathologically confirmed DIPNECH over a 6 year period. Each patient had a previous pulmonary nodule resection with confirmed DIPNECH pathologically. In each patient, we identified the 10 largest DIPNECH lesions, stratified according to anatomical location (two lesions per lobe). We measured the axial diameter (mm) and volume (mm3) in each lesion and followed these up in each subsequent CT scan to ascertain any longitudinal changes. We present the preliminary results of 250 lesions in 5 patients with CT scans over 5–6 years.

**Results**

We present preliminary data from 250 DIPNECH lesions in 5 patients over 6 years. The overall median CT follow-up was 1985 days (1450–2290 days). The median inter-scan interval was 365 days (349–826 days). No significant trend in axial CT diameter (r = 0.14, p = 0.037) or CT volume (r = 0.0093, p = NS) over time in the 10 lesions examined in each patient was noted. The overall trend in volume change over 6 years was not significant (p = 0.71 by ANOVA).

**Conclusions**

Our preliminary data suggests that pulmonary nodule change in size is slow and we could not detect any trends, irrespective of size at day zero, over 6 years of CT follow up. If confirmed by the complete data set, CT follow-up may be able to delayed rather than routine yearly scanning.
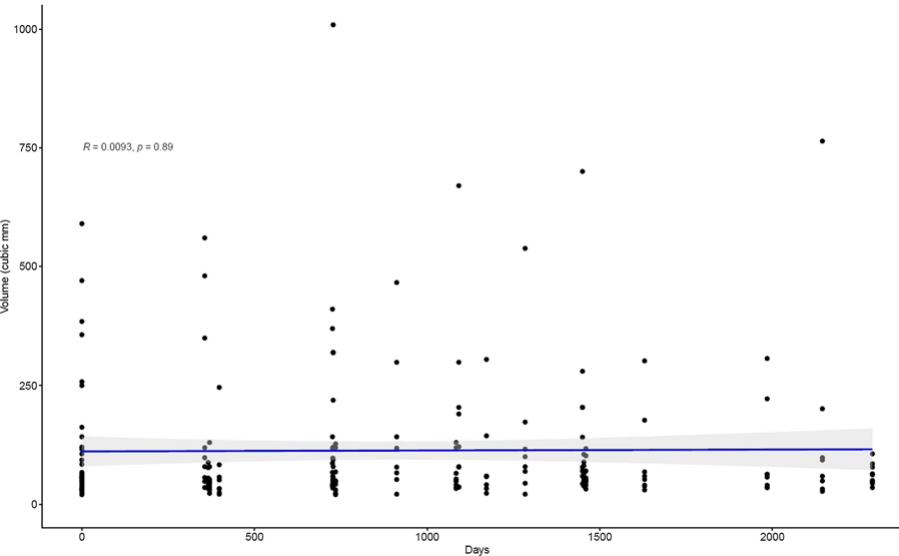


### A205 Early Experience with Customized Polydioxanone Biodegradable Tracheobronchial Stenting in Adult Airway Stenosis Following Lung Transplantation

#### Mayer, Nora^1^, Dr; Perikleous, Periklis^1^, Mr; Khoshbin, Espeed^2^, Mr; Asadi, Nizar^1^, Mr

##### ^1^Royal Brompton & Harefield Hospitals, Part of Guy`s and St. Thomas NHS Foundation Trust, Department of Thoracic Surgery, London, UK; ^2^Royal Brompton & Harefield Hospitals, Part of Guy`s and St. Thomas NHS Foundation Trust, Department of Heart &Lung Transplantation, London, UK

*Journal of Cardiothoracic Surgery* 2023, **18(Supp 1)**:A205

**Objectives**

Airway stenosis following lung transplantation (LTX) adversely affects quality of life and increases mortality. Established treatment options include bronchoscopic dilatation, debridement, stenting and lung resection. Customized biodegradable stents might offer advantages compared to conventional metal stents.

**Methods**

Patients with bronchial stenosis following LTX between 05/2019 and 10/2021 were included. Uncovered, customized, biodegradable polydioxanone (PDS) stents were inserted. Surveillance bronchoscopies for microbacterial and fungal growths were performed. Respiratory function tests, frequency of readmission and courses of antibiotics used were some of the measured outcomes.

**Results**

Seven biodegradable stents were inserted in three patients with non-anastomotic right bronchus intermedius (RIB) and left main bronchus stenosis diagnosed 2–7 months post LTX. All patients underwent multiple balloon-dilatations and two bare metal stents for bridging before BD-stent insertion were required. No bleeding or perforations were reported. One stent-migration was observed. All patients responded with relief of symptoms and steady increase in pulmonary function test after BD-stent insertion (ΔFEV1 1.56 l (50% pred.) to 3.1 l (91.5% pred.)).

**Conclusion**

Biodegradable customized tracheobronchial stenting is safe and efficient for the treatment of post-LTX airway stenosis. Stented airways remained patent while BD-stents were completely absorbed in two out of three patients. The patients presented with significant stable improvement in lung function.

### A206 Redo Minimally Invasive Pectus Excavatum Repair with Antomik Modelling Implant

#### Mulryan, Kathryn^1^, Dr; Redmond, Karen^2^, Prof

##### ^1^Beacon Hospital, Sandyford, Dublin, Ireland; ^2^Mater Misericordiae University Hospital, Dublin, Ireland

*Journal of Cardiothoracic Surgery* 2023, **18(Supp 1)**:A206

https://www.youtube.com/watch?v=0F59MlgBtjg

### A207 Management of Post-Operative Atrial Fibrillation After Lung Resection

#### Ahmed, Aaliyah^1^, Miss; Hashmi, Faisal^2^, Dr; Granato, Felice^2^, Mr

##### ^1^Manchester Medical School, Manchester, UK; ^2^Wythenshawe Hospital, Manchester, UK

*Journal of Cardiothoracic Surgery* 2023, **18(Supp 1)**:A207

This study evaluates the management of POAF in patients undergoing thoracic surgery.

This was a retrospective study which consists of patients (n = 481) including segmentectomy, lobectomy or pneumonectomy between April 2020–June 2021. 37 of these patients went into atrial fibrillation post operatively, equating to 7.7% of the total population. We looked at age, gender, continuation of beta blockers, digoxin use, type/site of procedure, length of stay (LOS) and post-operative complications. NICE guidelines (2004, amended 2016) recommend not to use digoxin for POAF.

A range of medications and combinations were used for POAF. The most common was bisoprolol (65%), followed by amiodarone (49%). 10.8% of patients received digoxin. The mean LOS was higher (3.1 days) in patients with POAF compared to those without POAF. The average LOS (days) with digoxin was 10.3, bisoprolol 9.6 and amiodarone 9.2. The CHADSVASC tool predicted 62.2% of the patients to be high risk of developing POAF.

To conclude, our study found CHADSVASC as a reliable tool to use for POAF risk prediction. Digoxin is still used as a first line agent in the clinical setting which is against NICE guideline recommendations and is associated with the longest LOS.

### A208 Impact of Preoperative Smoking Status on Outcomes following Lung Resection

#### Mantio, Kim^1^, Miss; Ahmed-Issap, Amber^1^, Miss; Jain, Shubham^2^, Dr; Habib, Akolade^2^, Dr; Spence, Angelica^1^, Miss; Brazier, Andrew^2^, Mr; Mahendran, Kajan^2^, Mr; Srinivasan, Lakshmi^2^, Miss; Ghosh, Shilajit^2^, Mr; Abah, Udo^2^, Miss

##### ^1^Keele Medical School / University Hospitals of North Midlands, Stoke-on-Trent, UK; ^2^University Hospitals of North Midlands, Stoke-on-Trent, UK

*Journal of Cardiothoracic Surgery* 2023, **18(Supp 1)**:A208

**Objective**

The study was designed to quantify the impact of smoking immediately prior to lung resection surgery.

**Methods**

We examined all consecutive lung resections at our institution from 01/01/2012 to the 07/07/2021. Variables where extracted from a prospectively filled database with missing data extracted from patient's records. Patients were divided into three cohorts; those who smoked within 1 month of surgery (current smokers), those who had smoked in the past (ex-smokers) and those who had never smoked (non-smokers). Length of ward and HDU stay was used to estimate costs per hospital episode.

**Results**

In total 2439 patients where identified, of these 481 (19.7%) had never smoked, 1450 (59.5%) were ex-smokers and 507 (20.8%) were smoking within a month prior to surgery. Pre-operative variables revealed worse lung function in current smokers when compared to ex and non-smokers. (% predicted FEV1 77, vs. 84.5 vs. 95.8, % predicted TLCO 58.3 vs. 63.4 vs. 72.2 respectively). Preoperative co-morbidity including; cardiac, vascular, cerebrovascular and respiratory disease was higher in the current and ex-smoking groups, however history of previous malignancy was higher in the non-smoking group. The average number of segments resected was 2.8 in the current and ex-smokers group and 2.2 in the non-smokers (a single wedge was classified as 1 segment). Postoperative complications were found to be significantly higher in current smokers when compared to ex-smokers. (36.9% vs. 30.5% P 0.007) In particular prolonged air-leak and postoperative respiratory tract infection (table 1). Length if ITU and overall hospital stay as well as average cost was also significantly higher in the current smokers when compared to ex-smokers and non-smokers.

**Conclusion**

Smoking immediately prior to surgery is associated with a significant increase in morbidity, length of stay and cost to the NHS. It is therefore essential to optimise this group of patients prior to intervention.

**Table:** 

	Non-smoker	Ex-smoker	Current-smoker
Overall Complications (%)	17	30.5	36.9
Arrhythmia (%)	2.7	5.8	4.7
LRTI (%)	4.6	11.2	18.1
Prolonged air-leak (%)	6.4	12.8	17
Length of stay (days)	5.5	7.6	8.3
HDU length of stay (days)	1.4	1.9	2.2
Average cost of post-operative stay (£)	3250.64	4508.04	5026.97
30-day mortality (%)	0.6	2.8	2.3


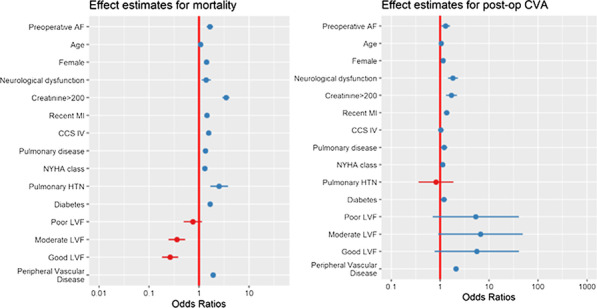


### A209 Use of Bilateral Paravertebral Blocks for Pain Management in Pectus Nuss Bar Patients

#### Williams, Jennifer, Miss; Musab, M., Mr; Devbhandari, M., Mr; Kornaszewska, M., Miss; Combellack, T, Mr; Pirtnieks, A., Mr; Valtzoglou, V., Mr

##### University Hospital of Wales, Cardiff, UK

*Journal of Cardiothoracic Surgery* 2023, **18(Supp 1)**:A209

**Objectives**

Managing post-operative pain in Pectus Nuss Bar patients can be challenging due to the complete remodelling of a young patient's chest wall. The initial week following a Nuss Bar insertion is typically the most painful period and our aim was to gain better control of this acute period. We aimed to improve early mobility, prevent hospital-acquired pneumonia, shorten length of hospital stay and improve patient satisfaction.

**Method**

Data was collected retrospectively from our PATs database for all patients undergoing a Pectus Nuss Bar insertion from January 2007 to October 2021. 69 patients were identified between 2007 and April 2016 who did not have bilateral paravertebral blocks inserted. Their post-operative outcomes were compared with 52 patients from April 2016 to October 2021 who did have bilateral PVB placed.

Pectus Nuss Bars are inserted under video-assisted thoracoscopic guidance which enables direct vision of PVB catheter placement into the right and left intercostal spaces. The PVB catheter is usually inserted into the 5th intercostal space bilaterally. Post-operatively, the bilateral paravertebral catheters remain insitu for four days and each patient has daily specialist pain team review. After four days, the fentanyl PVBs are discontinued and patients are stepped down to oral analgesia with a plan to discharge home.

**Results**

The 52 patients who underwent bilateral PVB during their Nuss Bar insertion had a 0% rate of hospital-acquired pneumonia, compared to 1.8% rate in the 69 patients between 2007 to 2016. The average length of stay was reduced to 4.8 (± 1.2) days from 5.6 (± 1.5) days. The rate of pneumothorax requiring chest drain insertion was also lower; only 2 patients between 2016 to present required a drain compared to 5 people 2007 to April to 2016.

**Conclusions**

We found in our single centre experience that bilateral PVB insertion improves Pectus Nuss Bar patient's speed and quality of recovery.

### A210 Review of Patients Discharged Post Thoracic Surgery with Chest Drain in Situ and Drain Follow-up Clinic

#### Aljanadi, Firas, Mr; Strickland, Jonathan, Dr; Montgomery, Liana, Mrs; Jones, Mark, Mr

##### Royal Victoria Hospital, Belfast, UK

*Journal of Cardiothoracic Surgery* 2023, **18(Supp 1)**:A210

**Objectives**

Persistent air leak and prolonged drainage are well-recognised complications of thoracic surgery. These complications increase the hospital stay and costs of care. Patients can be discharged with a chest tube in situ and followed up in a ward-based nurse-led clinic. We reviewed such patients and rate of readmission after discharge with a chest drain to assess the effectiveness of the drain follow-up clinic.

**Methods**

Retrospective review of our prospective database for 22 months (March 2019 to January 2021). We identified 62 patients who were discharged from the thoracic surgery ward with a chest drain and attached bag with one-way valve. Analysis focussed on indication and duration of chest drainage, complications, and readmission for any reason.

**Results**

62 patients were discharged with a chest drain in situ representing 5% of all the patients who had thoracic surgery within the study period. Median age was 67 years (range 22–85 years) with 24 females and 38 males. 52% of the patients underwent a video-assisted thoracoscopic approach, 27% of them a thoracotomy and 21% had an isolated bedside chest drain insertion. Following hospital discharge, median duration of chest drainage was 11 days [interquartile range (IQR) 7–18.75 days]. Patients had 106 review episodes in the ward-based nurse-led clinic. Indication was prolonged air leak (71%; 72 clinic reviews), persistent fluid drainage following evacuation of empyema (16%; 24 clinic reviews) and persistent fluid drainage for simple effusion (13%; 10 clinic reviews). Median length of drain stay was 30 days (IQR 19.75–54 days) for empyema, 10 days (IQR 6–16 days) for air leak and 8 days (IQR 6.5–12 days) days for simple effusion. 9 patients required readmission (14.5%) and empyema had developed in 3 patients (4.8%).

**Conclusions**

Patients discharged with a chest drain in place can be followed up in a dedicated ward-based nurse-led monitoring clinic for optimal quality of care.

### A211 A Service Evaluation of COPD Referral From Community to Secondary Care in a Community Based Screening Program

#### Bone, George, Mr; Desouza, Abigail-Sara, Miss; Woo, Edwin, Mr; Alzetani, Aiman, Mr

##### University Hospital Southampton NHS Foundation Trust, Southampton, UK

*Journal of Cardiothoracic Surgery* 2023, **18(Supp 1)**:A211

COPD is a prevalent respiratory disease that is insidious with high health and socio-economic burden. Early diagnosis, specialist referral and smoking cessation have a large influence on outcomes. A screening programme (Targeted Lung Health Check -TLHC) has been offering CT scans to ever smokers aged 55 to 74 to detect lung cancer. This has incidentally picked patients with emphysema and those with moderate to severe radiological changes were referred to a respiratory clinic/smoking cessation. This study aims to evaluate the referral pathway and its efficiency.

This study examined a cohort of participants who had a radiological diagnosis of moderate to severe emphysema as a result of their TLHC CT scan. Their route of referral from their scan to being seen in a specialist respiratory clinic and what management was delivered was recorded alongside information such as demographics, smoking status, comorbidities, ECOG performance status.

Between Sep 2019- Sep 2021 there were 274 participants were screened. Dyspnoea was noted in 214 with an ECOG > 1 in 37%. There was a diagnosis of emphysema in 42 patients and COPD in 144. All patients had moderate to severe emphysema on Low dose CT and were referred to a specialist respiratory clinic. Fifty five patient were seen within 7.7 (range 1–15 month). Covid-19 and the lack of senior respiratory clinicians were the main reason for the delay and for the minimal number reviewed. There were 143 current smokers who were signposted to local smoking cessation services but only 13 (9%) participants took part.

COPD is a major health concern in the UK and BTS has set guidelines on referring patients with advanced disease (GOLD 3/4) for specialist management including Lung volume reduction surgery (LVRS) and the recently NICE approved Endobronchial valve therapy. This study is a snapshot of how under detected this disease is and the need for a more efficient pathway from community to specialist secondary and tertiary care.

### A212 Effectiveness of Endotracheal or Endobronchial Stenting in Adult Expiratory Central Airway Collapse: A Systematic Review

#### Toale, Conor, Mr; Redmond, Karen C, Prof

##### Mater Misericordiae University Hospital Dublin, Dublin, Ireland

*Journal of Cardiothoracic Surgery* 2023, **18(Supp 1)**:A212

**Objectives**

This study analysed the available literature regarding the effect of endotracheal or endobronchial stenting on outcome measures in patients with expiratory central airway collapse (ECAC). The primary outcome measure was the change in FEV1 pre- and post-stent insertion. Secondary outcomes were changes in other physiological measures, symptomatology, and quality of life.

**Methods**

A systematic review was performed of the Embase, Pubmed, Web of Science and Cochrane library databases, according to the Preferred Reporting Items for Systematic Reviews and Meta-Analyses (PRISMA) guidelines. Articles were sought which included patients undergoing stent insertion for ECAC and reported on outcomes of interest.

**Results**

Seventeen articles were included in a narrative synthesis. Of the five studies reporting changes in FEV1 after stenting in patients with ECAC only, four could not demonstrate any significant improvement in FEV1. Only one study including patients with ECAC only recorded pre- and post-stenting FVC, and noted no significant difference. A total of 149 of 225 patients (66.22%) across five studies reported subjective improvement in one or more symptoms. The reported pooled stent-related complication rate was 0.074 per patient-month across six included studies.

**Conclusion**

Prospective observational studies with clear patient inclusion criteria are required to determine the effect of stenting on outcomes in ECAC. Available evidence does not demonstrate improvements in pulmonary function test measures after endoluminal stenting for patients with ECAC. Patient-reported outcomes such as subjective symptom improvement and quality of life measures are likely to be more useful than objective spirometry measures in determining treatment success.

### A213 The Impact of the COVID-19 Pandemic on Urgent Referrals to the National Thoracic Surgery Unit in Ireland

#### Kelly, Michelle, Ms; Eaton, Donna, Prof; Redmond, Karen, Prof; Brown, Rachel, Mrs

##### Mater Misericordiae University Hospital, Dublin, Ireland

*Journal of Cardiothoracic Surgery* 2023, **18(Supp 1)**:A213

**Introduction**

The Irish Health Service (HSE) responded to the COVID 19 pandemic by forming a surge agreement in October 2020 with Private hospitals to provide services to a number of surgical and medical specialities including Thoracic Surgery.

**Objectives**

The aim of this study was to evaluate the impact of this agreement during the COVID-19 pandemic on urgent referral waiting times for patients requiring thoracic surgical intervention.

**Methods**

The study compares the referral to surgery waiting times in 2019 compared to October 2020- October 2021. All patients referred to either of the thoracic surgeons during this period were included in this retrospective review.

**Results**

Patients referred in 2019 prior to the HSE agreement had a median waiting time of six days from referral to surgery, this is compared to a median waiting time of 2 days with the HSE agreement in place. 49% of these patients were transferred and underwent surgery within 24 h of referral.

**Conclusion**

The HSE agreement has had a dramatic impact on referral to surgery times for patients awaiting urgent transfer for thoracic surgery in Ireland.

### A214 Return to Work and Activity After Rib-Fixation for Acute Chest Trauma: A Retrospective Matched-Cohort Study

#### Blythe, Andrew, Mr; Cassidy, Roslyn, Dr; Hill, Janet, Dr; Diamond, Owen, Mr; McManus, Kieran, Mr

##### Royal Victoria Hospital Belfast, Belfast, UK

*Journal of Cardiothoracic Surgery* 2023, **18(Supp 1)**:A214

**Objectives**

Rib fractures present heavy pain and functional burdens. Surgical stabilisation of rib fractures (SSRF) improves mortality, morbidity, and length of stay (LOS). However, the literature is limited regarding functional outcomes after SSRF. Our primary outcome was to determine if SSRF improved return to work (RTW) in patients with acute rib fractures. Our secondary outcomes were pain and quality of life (QOL) scores.

**Methods**

A retrospective matched cohort study was conducted of patients with rib fractures between 2008–2020 that underwent SSRF. Inclusion and exclusion criteria were applied to ensure relevance to the study. All eligible patients who underwent surgery were matched to non-surgically managed patients. Validated PROMs were used to collect data: specifically, the Work Productivity and Activity Impairment Instrument (WPAI), the Brief Pain Index (BPI) and the EQ-5D-5L for RTW, pain, and QOL respectively.

**Results**

Of 1841 trauma patients with rib fractures 66 underwent SSRF. After inclusion and exclusion criteria, 38 pairs of patients were eligible for the study; 30 pairs completed the questionnaires, a success rate of 79%. More patients in the SSRF cohort returned to work, but the difference was not significant. There was a significant decrease in productivity in the SSRF versus the non-fixed cohort. There was no difference in pain or QOL scores between the two groups. Importantly, the SSRF group had significantly higher LOS in hospital and ICU, indicating a difference in the injury profile of the two groups.

**Conclusion**

Patients who undergo SSRF for rib fractures have similar RTW rates, pain and QOL scores compared to patients managed conservatively. However, retrospective comparison studies in this patient population are challenging due to the confounding factors of polytrauma injuries and lack of an appropriate comparison group. This is the first study that uses a validated injury-specific PROM, the WPAI.

### A215 Managing Expectations of Endobronchial Valve Insertion

#### Williams, Jennifer, Miss; King, E, Miss; Musab, M., Mr; Combellack, T, Mr; Pirtnieks, A., Mr; Kornaszewska, M., Miss; Valtzoglou, V., Mr

##### University Hospital of Wales, Cardiff, UK

*Journal of Cardiothoracic Surgery* 2023, **18(Supp 1)**:A215

Patients with severe emphysema can lead a debilitating life, suffering with significant breathlessness and a reduced quality of life. Those patients suitable for endobronchial valve (EBV) insertion need appropriate counselling regarding the risks and benefits of EBVs. The aim is to provide EBV patients with a patient leaflet to help supplement their consultant lead clinic appointments. The leaflet is designed to help provide realistic outcomes for the acute and long term benefits of EBV insertion.

There is an industry patient pamphlet for the Zephyr valve however we generated our own leaflet to describe in more detail the patient pathway in our centre for our EBV patients. Following EBV insertion our patients will remain on bed rest for the first 24 h, then have daily chest x-rays for 5 days to assess for lobar lung collapse and are covered with a weeks course of doxycycline. Patients have an upto 25% chance of a pneumothorax requiring a chest drain and therefore will have a chest drain insertion kit at their bedside. There is a 25% chance of valve migration and failure of improvement of symptoms.

Patient leaflets are to be provided in outpatient clinics prior to EBV insertion following Consultant decision to treat. Prospective data collection in the form of patient feedback forms are to be collected in their first post-operative follow-up appointments. Long term follow-up maintained with an upto date chest x-ray to ensure any valve migration and loss of collapse is identified early.

Our single centre found that some patients who had a poor outcome and little symptom relief from their EBVs were highly disappointed. Alongside Consultant counselling in clinic a patient leaflet describing the full pathway should help to manage patient expections when inserting EBVs. Endobronchial valves are not a cure for their severe emphysema however can improve symptoms. COPD unfortunately remains a progressive illness and these patients are high-risk for any intervention.

### A216 Chest Trauma- Excess Mortality Review from A Single Trauma Centre

#### Kew, Ee Phui, Mr; Hunt, Ian, Mr

##### St George's Hospital, London, UK

*Journal of Cardiothoracic Surgery* 2023, **18(Supp 1)**:A216

**Objectives**

The Trauma Audit & Research Network (TARN) has identified 99 trauma patients who died despite having high Probability of Survival (PS) from 2016 to 2018 in our centre. Of the 99 patients, 11% (n = 11) had chest trauma as the main injury. Our aim was to review this group of patients and identify any contributing factors to their mortalities.

**Methods**

Retrospective review of patients identified by TARN as excess mortality. Imaging, case notes and coroner’s reports were studied.

**Results**

Of the 11 patients reviewed, 64% (n = 7) were female and 36% (n = 4) were male, with mean age of 86.9 years old and mean PS of 87.4. After reviewing all the case notes, 27% (n = 3) of deaths were deemed unexpected. One patient died due to aspiration and lower respiratory tract infection; another death was due to possible massive pulmonary embolus; the third unexpected death was a patient who was discharged and readmitted with sepsis. Further analysis of these 3 cases revealed that there was no significant incidence or clinical mismanagement related to their unexpected deaths. However, we found a need for better and quicker anaesthetic service to provide serratus anterior block for rib fractures. This review also raised the question whether there is any benefit in fixing the ribs of patients aged 80 and above. 73% (n = 8) of the excess mortality were deemed to be ‘expected’ despite high PS as those patients were all either elderly, frail with multiple co-morbidities or were extremely unwell on admission.

**Conclusions**

Better access to serratus anterior block for rib fractures in our unit is needed. The PS calculation needs to be reviewed as the score did not correlate with the actual outcome of our patient cohort. A well-designed study is needed to investigate any benefit of rib fixation in elderly patients in terms of prognosis and quality of life.

### A217 Are we Removing Chest Drains Correctly?

#### Kew, Ee Phui, Mr; Mangel, Tobin, Dr; Mozalbat, David, Mr; Tan, Carol, Ms; Smelt, Jeremy, Mr

##### St George's Hospital, London, UK

*Journal of Cardiothoracic Surgery* 2023, **18(Supp 1)**:A217

**Objectives**

There is a risk of air entrainment into the pleural cavity during chest drain removal thereby causing an iatrogenic pneumothorax. BTS guidelines states that chest drains should be removed during a Valsalva manoeuvre or during expiration. Bell et.al and Cerfolio et al. performed randomised controlled trials comparing Valsalva manoeuvre on maximal inspiration and expiration which did not show any difference in clinically significant pneumothorax post chest drain removal. The objective of this audit was to assess how many doctors and nurses in our cardiothoracic unit know how to remove chest drain using either recognised techniques (Valsalva manoeuvre or during expiration) using a standard of 100%.

**Methods**

A survey consisting of 6 questions was conducted in our unit during January 2021. The survey explored subjects’ confidence and experience in chest drain removal, whether they have received training, their preferred technique and the rationale. The findings were presented in April 2021. Re-audit was performed using the same survey during Oct 2021.

**Results**

32 subjects were interviewed in the first cycle (16 doctors, 16 nurses). More nurses than doctors reported having experience in chest drain removal (88% vs. 56%). 87% of nurses and 69% of doctors knew the recognised techniques. More nurses than doctors reported confidence score of 4/5 and 5/5 in chest drain removal (82% vs. 44%). Only 3 subjects correctly explained that Valsalva manoeuvre increases intrapleural pressure. In the second cycle, 45 subjects were interviewed (38 nurses and 7 doctors) and all of them knew the accepted techniques.

**Conclusions**

This study revealed the need for more chest drain removal training amongst doctors and further teaching on basic respiratory physiology to healthcare professionals. The re-audit showed significant improvement with 100% of the subjects reported using the correct technique.

### A218 Targeted Surgical Management of Slipping Rib Syndrome

#### Santhirakumaran, Gowthanan, Mr; Shah, Mohammed, Dr; Hunt, Ian, Mr

##### St George's Hospital, London, UK

*Journal of Cardiothoracic Surgery* 2023, **18(Supp 1)**:A218

**Objectives**

Slipping rib syndrome (SRS) is not well recognised by physicians and many patients have multiple consultations and investigations prior to diagnosis. SRS is caused by hypermobility of the costal cartilages of the 8-10th ribs. Recent studies have demonstrated the clinical effectiveness of surgical management via rib(s) excision/stabilisation. Dynamic ultrasound of the chest wall (DUS) has shown an emerging investigative tool in aiding diagnosis of SRS, in what is traditionally a clinical diagnosis. We aimed to determine if a standardised management protocol based on a targeted excision/repair technique could determine significant symptom improvement using health outcome measures.

**Methods**

A single centre retrospective analysis of all patients (n = 18) undergoing surgical intervention for SRS between September 2019 – July 2021. We obtained data using standardised health & pain questionnaires pre-operatively and post-operatively, outcomes compared with Mann–Whitney U test.

**Results**

Figure 1 summarises the patient pathway and the treatment algorithm for SRS devised in our unit. Median length of history was 6 years—DUS identified the excursion movement of the specific rib in all cases. All 18 patients underwent a targeted rib excision with/without stabilisation. Post-operatively, mean improvement in pain at 6 weeks was 46% (p < 0.01). Other outcome measures demonstrated patients had improvement of functional symptoms by 60% (p < 0.01) and severity of symptoms of anxiety/depression by 38% (p < 0.01).

**Conclusions**

A targeted surgical approach to SRS with DUS as an important investigative tool provides demonstrable clinical benefit. This management protocol has illustrated its reproducibility in standardising effective SRS treatment.
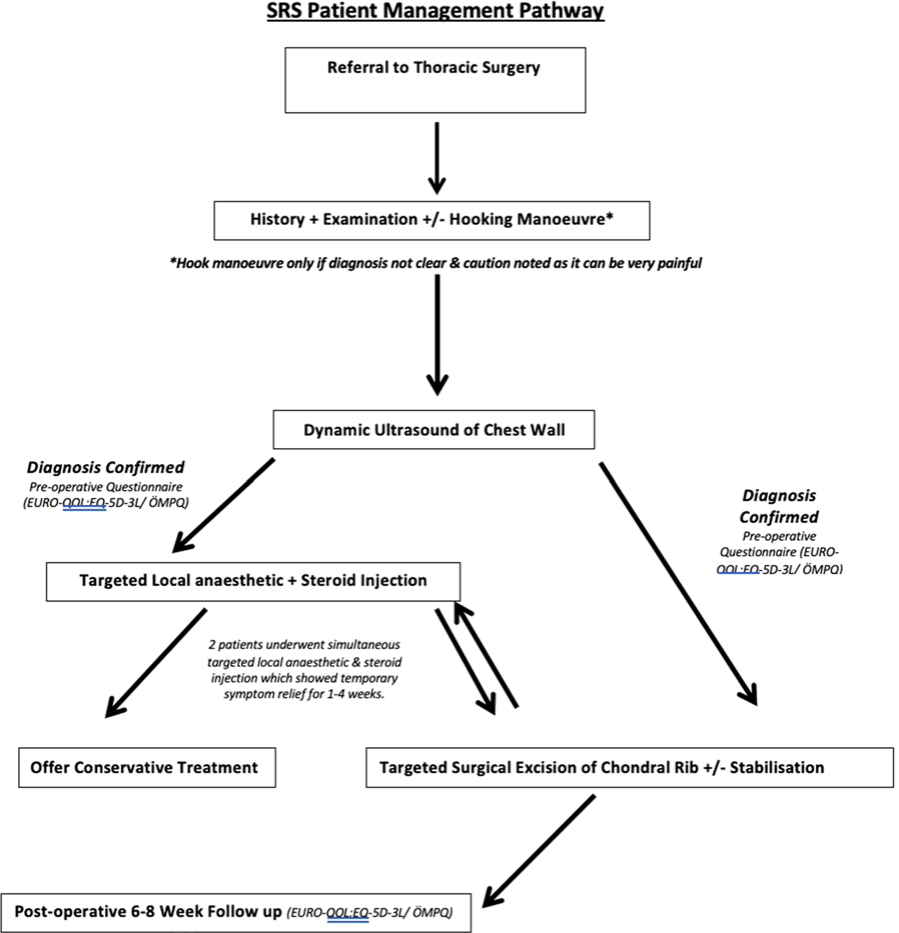


### A219 Results of Surgical Closure of Chronic Bronchopleural Fistula with Vascularized Tissues

#### Hammad, Walid, Mr

##### El-Hussein University Hospital, Al-Azhar Faculty of Medicine for boys, Cairo, Egypt

*Journal of Cardiothoracic Surgery* 2023, **18(Supp 1)**:A219

**Objectives**

This study was carried out to assess the efficacy of surgical closure of the chronic BPF using vascularized tissue buttress.

**Methods**

A prospective randomization of 28 patients with chronic BPF with or without empyema. Postoperative or non-operative etiologies were included. The classical clinical picture was a patient with pneumothorax on chest tube drainage showing varying bubbling during respiratory cycle and failure of lung to expand despite adequate pleural cavity drainage and antibiotic therapy. Fifteen patients had associated stage 2 empyema on the time of intervention. All patients were subjected to surgical intervention using different vascularized tissue transferred into the pleural cavity. These tissues were selected according to the location of the fistula, size of the residual space, and sterility of the pleural cavity. Based on proper preoperative planning and selection, the tissues used varied between intercostal muscle flap, latissmus dorsi muscle, Omental flap, or Pericardial pad of fat. Adequate preoperative drainage of the pleural cavity was mandatory. Followed by Intraoperative debridement and sterilization of the pleural cavity before closing the BPF with the tissue coverage.

**Results**

The duration of persistent air leak before deciding surgical intervention was varied significantly among our patients with a mean period of 5 ± 2.12 months. No patient required instillation of sealants through the tubes. There was immediate or early stoppage of air leak after the intervention in all patients. All patients had their drains removed before discharge. The mean hospital stay was 4 ± 1 day. Technical difficulties, deformities, chronic pain were reported.

**Conclusion**

Once the BPF develops, early recognition, drainage of the pleural space and control of the inflammatory process are critical. Surgical closure of chronic BPFs with proper vascularized tissues is an effective technique associated with low cost and lower hospital stay.

### A220 Outcomes of Video-thoracoscopic Minimally Invasive Pectus Excavatum Corrections: A Single Centre Experience from Wales

#### Devbhandari, Mohan, Mr; Koskolou, Stamatina, Dr; Williams, Jennifer, Miss; Combellack, Tom, Mr; Valtzoglou, Vasileios, Mr; Pirtnieks, Ainis, Mr; Kornaszewska, Malgorzata, Miss

##### University Hospital of Wales, Cardiff, UK

*Journal of Cardiothoracic Surgery* 2023, **18(Supp 1)**:A220

**Objectives**

To assess the outcomes of pectus excavatum repair in our centre and compare with the results from international centres of excellence.

**Methods**

Retrospective analysis of prospectively collected data on all patients undergoing pectus excavatum repair at our centre by a single surgeon from October 2014 to October 2021 were carried out. Continuous data was expressed as mean (± SD) for parametric data and median (IQR) for non-parametric data using Minitab version 19 statistical package.

**Results**

During this period a total of 92 patients underwent pectus repair surgery in out institution out of which 74 cases were operated for pectus excavatum while 18 operations were for pectus carinatum. Out of the Pectus excavatum group 10 underwent open modified Ravitch repair while a total of 64 patients underwent pectus repair surgery using Nuss bar through video assisted thoracic surgery (VATS) approach (study group). Patients in the study group were operated after spending a mean of 177 days (± 133 days) in the waiting list for surgery. There were 53 male and 11 female patients with mean age of 20.4 years (± 4.6 years). 23 of the patients were below the age of 18 while 41 patients were 18 and above. Their median post-operative stay was 4.9 days (± 1.5 days). Postoperative wound complications were seen in 5 patients which were managed successfully with local wound care. Patients were reviewed in the outpatient clinic in 6wk, 6 months, 1 year, 2 year and 3 years after which the bars were removed. Cosmetic improvement was good to excellent in all patients.

**Conclusion**

In our experience video-thoracoscopic minimally invasive Pectus excavatum correction with Nuss bar insertion produce excellent result.

### A221 Maintaining Standards of Care in Thoracic Surgery During the Sars-Cov-2 Pandemic

#### Alshammari, Abdullah, Dr; Hoffman, Ross, Mr; Chavan, Hemangi, Miss; Kaniu, Daniel, Mr; Gallesi, Jose Alvarez, Mr; Pons, Aina, Ms; Jordan, Simon, Mr; Begum, Sofina, Miss; Buderi, Silviu, Mr

##### Thoracic Surgery, The Royal Brompton Hospital, London, UK

*Journal of Cardiothoracic Surgery* 2023, **18(Supp 1)**:A221

**Objectives**

During SARS-CoV-2 pandemic measures were implemented to continue delivering high-quality thoracic surgery service. This study reports our experience in delivering elective lung resection surgery, including mortality and morbidity.

**Methods**

This is a retrospective study of all patients between March 2020 to September 2020. Data were obtained from Patient Assessment and Tracking System (PATS). During this period, a set of measures were implemented while working across different hospitals around London, including self-isolation, pre-operative SARS-CoV-2 screening, virtual consultations and remote pre-assessment. Digital platforms were employed to facilitate communication between members of the multidisciplinary team. Descriptive statistics used to analyse the data.

**Results**

A total of 214 patients included for analysis, of which 99 patients had lobectomies. The mean age was 64.4 (10–87) years and 57 were females. There was no recorded mortality. Seven patients had post-operative complications including pneumonia, respiratory failure requiring ventilatory support in the intensive care unit and one required completion pneumonectomy. The mean Thoracoscore was 1.66 (0.06 –9.5). The mean length of stay was 5.5 (1–24) days. When compared to our practice in 2019, these results are similar. None of the patients developed SARS-CoV-2 infection post-operatively.

**Conclusion**

It was possible to maintain the expected standards of care with acceptable surgical outcomes during the pandemic. This was achieved with deliberate implementation of technology, set measures, and working in collaboration with the multidisciplinary team.

### A222 The Use of Carinal Y-stents in the Emergency Management of Peri-operative Injury/dehiscence of the Major Airways

#### Smith, Edel, Dr; Brown, Rachel, Ms; Aladaileh, Mohammad, Dr; Eaton, Donna, Prof

##### Mater Misericordiae University Hospital Dublin, Dublin, Ireland

*Journal of Cardiothoracic Surgery* 2023, **18(Supp 1)**:A222

**Objectives**

Major airway injury/disruption is rare and can be difficult to manage. Surgical repair is favoured however, in patients with large/complex defects or in frail patients management may involve stenting of the airway. We present 5 patients all of whom had a carinal/Y-stent sited for urgent or emergency management of major airway defects.

**Methods**

We included all patients who had a Leufen carinal/Y-stent inserted for emergency management of major airway injury. Stents were inserted using a rigid bronchoscopy under both bronchoscopic and fluoroscopic guidance.

**Results**

The aetiology of the injury in 3 cases was intra-operative injury to the major airways; 1 during tracheal resection, 1 during thyroid surgery and 1 following an oesophagectomy. A further 2 patients had right main airway anastomotic dehiscence post-operatively, 1 post double-lung transplantation and 1 post-right upper lobe sleeve lobectomy.

In all cases the y-stent covered the airway defect, allowing all patients to be extubated following stent insertion. All patients subsequently underwent uncomplicated elective stent removal with complete resolution of the underlying defect.

**Conclusions**

In all cases the major airway disruption was successfully managed using a carinal Y-stent that was subsequently removed. No further airway interventions were required in any patient.

### A223 Robotic Approach to the Sympathetic Chain

#### Brown, Rachel, Mrs; Aladaileh, Mohammad, Mr; Toerien, Lara, Ms; Eaton, Donna, Prof

##### Mater Misericordiae University Hospital, Dublin, Ireland

*Journal of Cardiothoracic Surgery* 2023, **18(Supp 1)**:A223

**Introduction**

A robotic approach to the sympathetic chain provides superior dexterity, magnification and 3D visualization. This facilitates a highly selective sympathectomy (with division of only pre- and post-ganglionic fibres) in patients with hyperhidrosis and precise division of the stellate ganglion in patients requiring cardiac denervation.

**Methods**

All cases undergoing robotic surgery on the sympathetic chain for hyperhidrosis, facial flushing and cardiac denervation (except unstable patients) were included.

**Results**

Analysis from the Intuitive DaVinci robotic system shows the median operating time for all procedures on the sympathetic chain is 62 min. 92% of the cases cost E270 for consumables, the remaining 8% averaged a cost of E660 due to additional instruments required to manage more complex adhesions. The length of stay is reduced in the robotic group with the majority of those undergoing selective sympathectomy being done as day cases. There have been no complications.

**Conclusion**

Cost and operator times are comparable, patient length of stay is reduced in the robotic programme. We found that a robotic approach improves dexterity, visualisation and precision. This approach allowed accurate division of the sympathetic chain facilitating a highly selective sympathectomy for hyperhidrosis and allowing precise division of the stellate ganglion in patients undergoing cardiac denervation.

### A224 Reducing the Critical Care Burden of Patients Undergoing Sternotomy for Non-Cardiac Surgery at our Institution

#### Earnshaw, Charlotte, Dr; Elston, Victoria, Miss; Abdelhadi, Ahmed, Dr; Gurney, Stefan, Dr; Kamalanathan, Kajan, Dr

##### University Hospitals Bristol and Weston NHS Foundation Trust, Bristol, UK

*Journal of Cardiothoracic Surgery* 2023, **18(Supp 1)**:A224

**Objectives**

A change in local practice resulted in non-cardiac sternotomy patients being managed on the ward post-operatively, rather than defaulting to critical care. We sought to evaluate our rate of unplanned critical care admission subsequent to this intervention and make comparison of routine practice to thoracic units nationally.

**Methods**

We retrospectively reviewed electronic and paper records from our enhanced recovery database for all non-cardiac sternotomy patients from March 2016 until July 2021. We then contacted all UK thoracic centres via telephone or email to establish patterns in current national practice.

**Results**

There were a total of 23 patients. Nine patients (39.1%) spent ≥ 1 post-operative day in critical care. Seven patients were electively admitted to critical care due to co-morbidities or extent of surgery, and there were two unplanned admissions. Nationally, 60% of centres routinely send all non-cardiac sternotomies to a critical care or higher-level care area and 40% automatically provide post-operative care on the ward, unless significant patient co-morbidities exist.

**Conclusions**

The majority of UK thoracic centres manage non-cardiac sternotomy patients in critical or higher-level care post-operatively. Critical care bed capacity is a limited resource, especially in the current climate. With appropriate training of nursing and medical staff these patients can be managed routinely on a normal thoracic ward. In our institution, this change in practice has led to improvements on patient flow and reduced the burden on critical care bed capacity.

### A225 Minimally Invasive Total Thymectomy in Myasthenia Gravis

#### Santhirakumaran, Gowthanan, Mr; Hunt, Ian, Mr; Tan, Carol, Ms; Smelt, Jeremy, Mr

##### St George's Hospital, London, UK

*Journal of Cardiothoracic Surgery* 2023, **18(Supp 1)**:A225

**Objectives**

The role of thymectomy via a median sternotomy approach in treatment of myasthenia gravis has demonstrated long term clinical benefit with a multicentre randomised control trial. However, as with lung resection, a minimally invasive approach could improve morbidity and length of stay. We aim to assess the impact of minimally invasive approach on morbidity and its comparable therapeutic benefit in myasthenia gravis.

**Methods**

A single centre retrospective review of all myasthenia gravis patients undergoing minimally invasive total thymectomy between January 2019–November 2020 was performed. Data consisted of patient demographics, symptoms, pre-operative medications, post-operative length of stay, complications, 12 month follow up of symptom improvement and medication.

**Results**

A total of 21 myasthenia gravis patients (Osserman Classification 1–4), with a mean age of 37 years, underwent a total thymectomy via a minimally invasive approach. None required a conversion to a sternotomy, median post-operative length of stay was 3 days and 1 patient had a recognised complication of post chest drain removal pneumothorax requiring drain re-insertion. At follow-up, 95% of the patient cohort had improvement or complete resolution of myasthenic symptoms. 82% that pre-operatively required immunosuppression with prednisolone did not require immunosuppression or received reduced dosage. 60% had significant symptom improvement with associated reduced daily requirement or discontinuation of pyridostigmine.

**Conclusions**

Minimally invasive total thymectomy in myasthenia gravis may offer reduced morbidity and similar long-term clinical benefit to that found in the traditional open approach.

### A226 Pulmonary Complications and Mortality of Veno-venous Extracorporeal Membrane Oxygenation as Treatment for COVID-19 Pneumonitis

#### Norkunas, Mindaugas, Mr; Hoffman, Ross, Dr; Somasundram, Khevan, Dr; Aw, TC, Dr; Singh, Suveer, Prof; Shaarawy, Ezeldin, Dr; Buderi, Silviu, Mr; Begum, Sofina, Miss; Lim, Eric, Prof; Jordan, Simon, Mr

##### Royal Brompton and Harefield NHS Foundation Trust, London, UK

*Journal of Cardiothoracic Surgery* 2023, **18(Supp 1)**:A226

**Objectives**

Veno-venous extracorporeal membrane oxygenation (vv-ECMO) is effective treatment for refractory hypoxemia caused by COVID-19 virus. Our aim was to review the rate of pulmonary complications, their treatment and survival to discharge for these patients.

**Methods**

It was a retrospectively conducted analysis of prospectively collected data of patients, treated with vv-ECMO for Covid-19 infection caused respiratory failure, between March 2020 and October 2021. The end points where incidence, treatment choice and in-hospital mortality.

**Results**

During this period 166 patients (mean age 51, SD ± 13.75) required vv-ECMO. In 42 (25.3%) cases patients needed intervention to treat pulmonary complications by radiological or surgical intervention. 27 (16.2%) patients had pneumothorax and 28 (16.8%) pleural effusion/haemothorax requiring drain insertion. In 13 (7.8%) cases patients needed treatment for both. 7 (4.2%) patients required video assisted thoracoscopic surgery, six of them for haemothorax and one for recurrent pneumothorax. In-hospital mortality in intervention group was 13 (30.95%;) vs 27 (21.77%; p = 0.355) in no intervention group.

**Conclusions**

Timely and multidisciplinary-led interventions allowed this complex group of patients have statistically similar survival rate compared to no intervention group.

### A227 Impact of Extremes of BMI on Outcomes Following Lung Resection

#### Jain, Shubham, Dr; Ahmed-Issap, Amber, Miss; Habib, Akolade, Dr; Mantio, Kim, Miss; Spence, Angelica, Miss; Brazier, Andrew, Mr; Mahendran, Kajan, Mr; Srinivasan, Lakshmi, Miss; Ghosh, Shilajit, Mr; Abah, Udo, Miss

##### University Hospitals of North Midlands, Stoke-on-Trent, UK

*Journal of Cardiothoracic Surgery* 2023, **18(Supp 1)**:A227

**Objective**

BMI has been demonstrated to be an independent predictor of survival following lung resection for NSCLC, with a low BMI associated with worse survival and a high BMI associated with a protective effect. We designed this study to quantify the short-term impact of abnormal BMI on outcomes following lung resection.

**Methods**

We examined all consecutive lung resections at our institution from 01/01/2012 to the 07/07/2021. Variables where extracted from a prospectively filled database with missing data extracted from patient's records. Patients were divided into three cohorts; those with a low BMI (< 18.5), those with a normal/high BMI (18.5–29.9) and the obese (BMI > 30) post-operative complications, length of stay and 30-day mortality were examined.

**Results**

In total 2439 patients where identified, of these 2341 BMI value available and were included in the study. 60 patients (2.6%) had a BMI < 18.5, 1586 (67.7%) had a BMI of 18.5–30 and 695(29.7%) had a BMI > 30. Preoperative comorbidities including HTN, DM, previous malignancy and elevated cholesterol were higher in the obese group. However IHD, cerebrovascular disease and cardiac failure were higher in the mid-range group and peripheral vascular disease, pulmonary disease and high alcohol intake higher in the low BMI group. Postoperative complications were found to be significantly higher in the low BMI group when compared to a normal and high BMI which appeared to be protective (Table 1).

**Conclusion**

Low BMI is associated with significantly worse postoperative outcomes and a four-fold increase in mortality when compared with normal and high BMI. Obesity appears to incur a protective effect in terms of both morbidity and mortality.

**Table:** 

	Low BMI < 18.5	BMI 18.5–30	Obese BMI > 30
Overall Complications (%)	43.3	30.8	23.2
Arrhythmia (%)	10	5.2	3.7
LRTI (%)	26.7	10.8	8.9
Prolonged air-leak (%)	25	14.4	6.9
Length of stay (days)	10.2	7.5	6.3
HDU length of stay (days)	2.9	1.9	1.5
30-day mortality (%)	8.3	2.0	1.7

### A228 Is the 3D Reconstructive Scan a Useful Tool in Lung Cancer Surgery?

#### Tahhan, Ghis^1^, Dr; Combellack, Tom^2^, Mr; Pirtnieks, Ainis^2^, Mr; Valtzoglou, Vasileios^2^, Mr; Kornaszewska, Malgorzata^2^, Mrs

##### ^1^Cardiff University, Cardiff, UK; ^2^University Hospital of Wales, Cardiff, UK

*Journal of Cardiothoracic Surgery* 2023, **18(Supp 1)**:A228

**Objectives**

Lung cancer surgery considers challenging due to the complexity of vascular or bronchial variations, lesions position and the amount of margin resected. Can the three-dimensional reconstructive scan preoperatively facilitate the surgical procedure and provide surgeons with valuable information about the anatomical and tumour locational variations?

**Methods**

From March 2021-June 2021, we built 3D reconstruction scan pre-operatively for 24 thoracic surgery patients following certain criteria. Which is patient who will undergo (Segmentectomy, lobectomy or challenging wedge resection) for peripheral or central lesions, no limitation on age or gender, patients definitive diagnosed and had CT thorax with contrast preoperatively. Then the main indicator was a scalable questionnaire survey completed by surgeons postoperatively regarding the impact of scans on facilitating the procedure, making a different decision of surgical operating approaches and appreciation of vascular variations.

**Results**

The project cohort of 24 patients who 5 underwent VATS segmentectomy,16 underwent VATS lobectomy,1 underwent wedge resection and 1 underwent nucleated of central nodule. There was one case cancelled due to hidden invasion of main bronchus on the CT. Postoperatively the surgeons’ sentiments regarding the scans were very positive specially for vascular variations as all anomalous or uncommon bronchioles and vessels were accurately identified by 3D imaging. Furthermore, there was consensus about the beneficial value of scans in segmentectomy, central lesion near hilum and difficult wedge operations.There were 2 cases where 3D scans played a significant decision-making as the surgical approach was changed and a smaller resection of lung tissue was achieved.

**Conclusions**

3D reconstructive scans have significant values in segmentectomy, central lesions and decision-making of resectable amount of lung in borderline patients and constitute a very handful tool for sergeons.
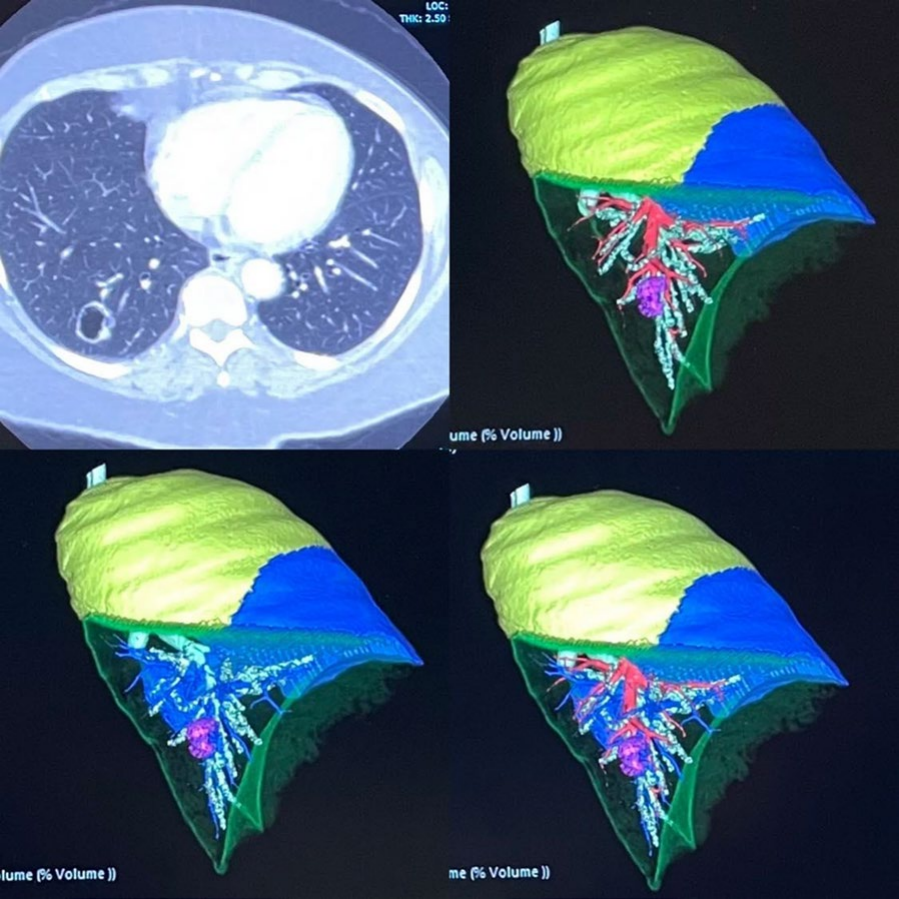


### A229 Does Robotically Assisted Thoracoscopic Volume Reduction Surgery Use Less Hospital Resources Than Bronchoscopic Lung Volume Reduction?

#### Evans, Nicholas, Mr; Perikleous, Periklis, Mr; Lee, Michelle, Miss; Colombino, Anna Maria, Ms; Baranowski, Ralitsa, Miss; Waller, David, Mr

##### St Bartholomew's Hospital, Barts Health NHS Trust, London, UK

*Journal of Cardiothoracic Surgery* 2023, **18(Supp 1)**:A229

**Objectives**

Lung volume reduction (LVR) can be performed by bronchoscope (BLVR) or surgery (LVRS). There are no randomized comparisons, but the assumption is that BLVR requires less use of hospital resources which offsets the higher cost of endobronchial valves. We have compared the two in patients suitable for both.

**Methods**

In a 4-year experience, we have performed 176 LVR procedures in 132 patients: 76 patients underwent Robotically Assisted Thoracoscopic (RATS) LVRS and 56 BLVR. We offered both approaches as a one-stop treatment, based on intra-operative assessment of collateral ventilation (CV). BLVR was the treatment of choice in CV negative patients, while CV positive patients proceeded to unilateral RATS LVRS. We compared use of hospital resources, including theatre time, high-cost consumables and hospital stay. Data also included complications, readmission to hospital and requirement for high level of care.

**Results**

No patient in the LVRS group required a redo procedure while eight had staged bilateral procedures. 26 (46%) patients in the BLVR group had two or more redo procedures, including revision bronchoscopy (n = 3), valve removal/re-insertion (n = 23), VATS for pneumothorax (n = 8).

Initial failure of EBLVR results in comparative overall hospital stay to initial LVRS.

**Conclusions**

Uncomplicated preferential BLVR appears to use less hospital resource than RATS LVRS in comparable groups. However, this advantage is lost if revision BLVR procedures are needed. Further study is needed to answer whether redo bronchoscopy should be abandoned in favour of conversion to salvage LVRS.
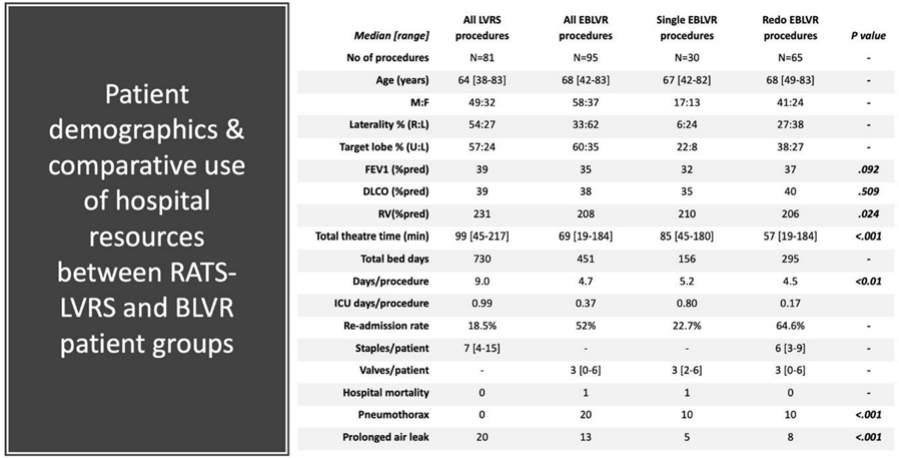


### A230 Comparing the Outcomes of Serially Performed Bilateral Lung Volume Reduction Surgeries

#### Hoffman, Ross, Mr; Alshammari, Abdullah, Dr; Alvarez Gallesio, Jose, Dr; Norkunas, Mindaugas, Dr; Buderi, Silviu, Mr; Jordan, Simon, Mr

##### Royal Brompton Hospital, London, UK

*Journal of Cardiothoracic Surgery* 2023, **18(Supp 1)**:A230

**Objectives**

Lung volume reduction surgery (LVRS) is an established treatment for advanced COPD. Some evidence suggests simultaneous bilateral lung volume reduction surgery should be avoided and that a unilateral approach may reduce postoperative morbidity in a high-risk population. Other evidence suggests staged procedures can prolong the benefit of LVRS for patients. This paper quantifies the benefits and risks of second serial LVRS by comparing the outcomes of the first and second serial LVRS operations.

**Methods**

Data extracted retrospectively for 30 patients who underwent bilateral serial LVRS from September 2007 to October 2021 at a single surgical centre are used to estimate the differences-in-differences in outcomes between the first and second serial LVRS surgeries. The mean time between the first and second surgeries was 2.9 years.

**Results**

Both groups had significant improvements in their lung function after each serial surgery, however there was no significant difference-in-difference in outcomes between serial surgeries.

**Conclusion**

There is significant benefit to be gained from second side LVRS. The risk profile of surgery is similar and the relative improvement in lung function is at least as good.
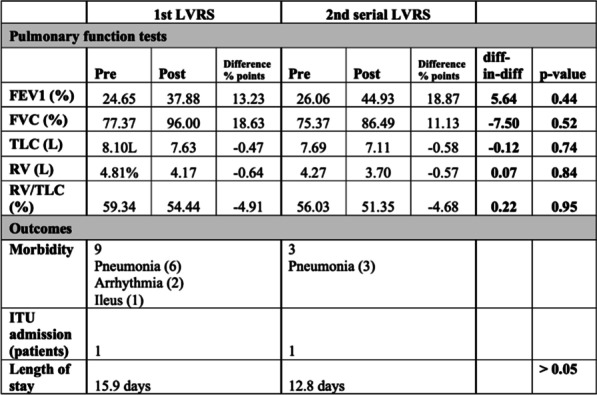


### A231 Blood Product Utilisation in Thoracic Surgery

#### Chubsey, Rachel^1^, Miss; Nithiananthan, Mayooran^2^, Mr; Rathinam, Sridhar^1^, Mr

##### ^1^Glenfield Hospital, University Hospitals of Leicester NHS Trust, Leicester, UK; ^2^Nottingham University Hospitals NHS Trust, Nottingham, UK

*Journal of Cardiothoracic Surgery* 2023, **18(Supp 1)**:A231

**Introduction**

Advances in minimally invasive thoracic surgical techniques has reduced the risk of intra-operative bleeding and requirement for blood transfusion. As a result, over-ordering of blood products may put additional strain on time and resources. The aim of this study was to compare blood product ordering and subsequent usage with recommended departmental Maximum Surgical Blood Ordering Schedule (MSBOS).

**Methods**

Retrospective data collection from ORMIS Theatre Management software and Sunquest ICE System for all patients undergoing surgery in August 2020. Data included demographics, procedure, urgency, co-morbidities, pre-operative haemoglobin and platelets, number of group and screen samples, units crossmatched and units transfused.

**Results**

57 patients were included, average age 62.03 years (50–73.5), 30 (52.6%) males. 46 (80.7%) were elective and 38 (66.6%) for malignancy. All patients had one Group and Screen sample, while 46 (80.7%) were crossmatched units of packed red cells.

Our MSBOS recommended crossmatch for patients undergoing Pneumonectomy/EPD (n = 3), Decortication (n = 4) and VATs/Open Lung resection (n = 14). Crossmatch was performed for 100%, 100% and 85.7% respectively. Of these only patients 4 (19%) received a blood transfusion.

We also regularly crossmatched patients for mediastinoscopy (n = 2), LVRS (n = 2), VATs bullectomy/pleurectomy (n = 7) and bronchoscopy (n = 13), (50%, 50%, 85.7% and 53% respectively). None of these patients required a blood transfusion.

**Conclusions**

The results suggest increased ordering of blood products for procedures associated with smaller bleeding risk such as VATs procedures and bronchoscopy. However, due to the emergency nature of intra-operative haemorrhage it is difficult to modify practice. We recommend a reduction in the number of crossmatched units for VATs, bronchoscopy and mediastinoscopy in line with Trust Guidance and re-audit of practice.

### A232 Anatomical vs Non-anatomical Resection in Patients Undergoing Lung Volume Reduction Surgery

#### Hoffman, Ross^1^, Mr; Alshammari, Abdullah^2^, Dr; Alvarez Gallesio, Jose^2^, Dr; Norkunas, Mindaugas^2^, Dr; Buderi, Silviu^2^, Mr; Jordan, Simon^2^, Mr

##### ^1^Department of Thoracic Surgery, Royal Brompton Hospital, London, UK; ^2^Royal Brompton Hospital, London, UK

*Journal of Cardiothoracic Surgery* 2023, **18(Supp 1)**:A232

**Objectives**

Lung volume reduction surgery is classically performed as a non-anatomical sublobar (wedge) resection of lung tissue. There is some literature that examines the benefit for patients undergoing a lobectomy for emphysema and co-existing cancer; however, anatomical lobectomy has not been well studied as a primary intervention for LVRS. This paper examines whether anatomical resection confers more benefit than non-anatomical resection.

**Methods**

Data were extracted retrospectively for patients who underwent LVRS from September 2007 to October 2021, at a single surgical centre. The patients were grouped for comparison by type of resection performed: anatomical resection (lobectomy/bilobectomy) or non-anatomical (sub-lobar/wedge).

**Results**

A total of 274 patients were included. Anatomical resection was performed on 23.7% (n = 65) of the patients, and for two of these patients it was for lung cancer. There were no statistically significant differences between the two groups at baseline, in terms of age, sex, BMI, FEV1, FVC, and RV/TLC ratios. However, patients who were selected for anatomical resection had a larger TLC and RV preoperatively (p < 0.05). Both groups had significant improvement in their pulmonary function tests postoperatively in terms of FEV1, FVC and TLC (p < 0.01). Comparatively, a statistically significant greater improvement from their preoperative values was found in the anatomical group vs the non-anatomical group in terms of RV (reduction of 1177.5 ml vs 494.8 ml, p < 0.05), and RV/TLC ratio (reduction of 11.3% vs 3.9%, p < 0.01). There was no statically significant difference between the length of stay, morbidity or survival (log rank, p = 0.336) between the groups.

**Conclusions**

Anatomical resection for COPD, in carefully selected patients may lead to greater improvements in postoperative RV and RV/TLC ratio. In this study, there was no significant difference in length of stay or survival between anatomical and non-anatomical LVRS.
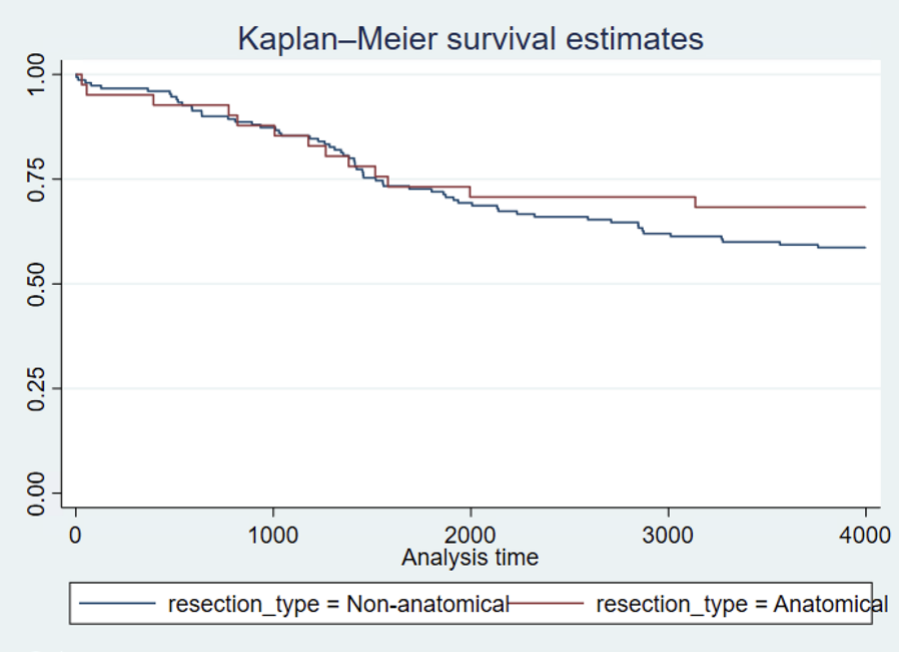


### A233 Developing Procedure Specific Consent Forms for Thoracic Surgery

#### Hoffman, Ross, Mr; Alshammari, Abdullah, Dr; Alvarez Gallesio, Jose, Dr; Buderi, Silviu, Mr

##### Royal Brompton Hospital, London, UK

*Journal of Cardiothoracic Surgery* 2023, **18(Supp 1)**:A233

**Introduction**

Cardiothoracic surgery can carry a high risk of morbidity and mortality relative to other commonly performed surgical procedures. It is crucial that the consent process for surgery is thorough, effective and comprehensive to ensure that the patient's consent is truly informed and that the surgical team is protected from medical litigation. Written consent forms are prone to error through omission, poor legibility, inaccuracy or lack of clarity.

**Methods**

We have developed and instituted 6 comprehensive procedure specific consent (PSC) forms for use in our thoracic surgery department, for which we have received excellent feedback from patients and staff.

The procedure specific consent forms include:

- Lung resection
- Bronchoscopy, including rigid bronchoscopy
- Diagnostic VATS procedure
- VATS procedure for pneumothorax
- Mediastinoscopy
- Chest wall resection

**Results and conclusion**

The use of procedure specific consent forms helps to ensure that legible and standardised information about the procedure and its risks are communicated to the patient. Patients are also able to receive their consent forms in advance of arriving for surgery, which can faciliate a more informed consent discussion. High-quality PSC form templates for thoracic surgery are scarce. In sharing this set of forms, we hope to encourage other surgical departments to shift to a PSC standard.

### A234 MGTX Impact on Thymectomy Practice

#### Badran, Abdul^1^, Dr; Allen, Claire^1^, Dr; Badran, D^2^, Dr; Pinto, Ashwin^1^, Dr; Woo, Edwin^1^, Mr

##### ^1^University Hospital Southampton, Southampton, UK; ^2^Imperial College London, London, UK

*Journal of Cardiothoracic Surgery* 2023, **18(Supp 1)**:A234

**Objectives**

In August 2016, the results of the international randomized, controlled, trial that studied the safety and efficacy of thymectomy for patients with non-thymomatous myasthenia gravis (MG), MGTX was published. We sought to review the impact this landmark trial had on surgical management in MG.

**Methods**

We retrospectively reviewed the demographics as well as clinical factors of patients with MG that underwent surgery in a 3-year period pre MGTX (2013–2016) and 3 years post MGTX (2016–2019). This resulted in a total of 21 patients being identified.

**Results**

9% (n = 2) were pre MGTX and 90% (n = 19) post. Mean age at thymectomy was 49 (47.5 post vs 58 pre), 9 were males (n = 1 pre) and 12 were female (n = 1 pre). Mean days to surgery 603 (478 post vs 1393 pre). Thymectomy was performed in 18 patients with a VATS approach (17 post, 1 pre) with 6 conversions to open which were all in the post-trial period. In one of the VATS cases (post-trial period) a subxiphoid approach was utilised. Definitive histology showed thymoma in 48% (n = 10, 8 post and 2 pre). In 4 cases there was phrenic nerve dysfunction (3 post and 1 pre). There was one laryngeal nerve injury in the post-trial period. In one patient surgery was abandoned (post-trial period) after complications during VATS and risk of sternotomy not warranted, Mean length of stay (LOS) was 4.5 days (7.3 pre and 4 post), in VATS patients LOS was 2.9 days post and 4 days pre.

**Conclusions**

There is a shorter time for diagnosis to surgery meaning patients can potentially have less medication and better control of disease earlier. There has been a significant uptake of surgery in MG patients post MGTX interestingly this is also being seen in thymomatous MG. The standard approach adopted is VATS in the post-trial period. The number of days to surgery was also significantly less in the post-trial period.

### A235 The Role of Dexamethasone in Post-operative Pain in Thoracic Surgical Patients

#### Toerien, Lara^1^, Ms; Daly-Devereux, Madeleine^1^, Dr; Weedle, Rebecca^2^, Ms; Rice, Darragh^2^, Mr; Healy, David^1^, Prof

##### ^1^St Vincent's University Hospital, Dublin, Ireland; ^2^Mater Misercordiae Hospital, Dublin, Ireland

*Journal of Cardiothoracic Surgery* 2023, **18(Supp 1)**:A235

**Objectives**

Post-operative pain in thoracic surgery is a topical and complex issue. Ineffective pain relief has been shown to impede coughing, deep breathing, and mobilisation, culminating in increased morbidity and mortality. Infiltration of glucocorticoids is used in other fields, but its efficacy in thoracic surgery in improving post-operative pain has not yet been explored.

**Methods**

We performed a double blinded, randomised control trial, in patients undergoing elective thoracic procedures, through robotic, VATS or open thoracotomies. Patients were randomly allocated to receive 8 mg Dexamethasone, 4 mg Dexamethasone, or saline (control) which was infiltrated into port/thoracotomy sites prior to incision. Our primary outcome was to measure pain scores and opiate requirements, in each of the surgical approaches. Any adverse effects and relation to steroid dose was also observed.

**Results**

59 patients were included, and allocated to one of the 3 groups, then subdivided into robotic, VATS and open. Opiate requirements were statistically significantly different between groups in the 24-to-48-h time period (χ2(2) = 8.4, *p* = 0.015), where there were less opiates used in the 4 mg group (21.4 mg) compared to the 8 mg group (36.6 mg) (*p* = 0.012) and control group (30,3 mg). However, pain scores did not appear to be significantly improved by Dexamethasone. Pain scores and opiate requirements were also lowest in the VATS group, when compared with the robotic and open cohorts. Issues with wound dehiscence/infection were noted in only 8 patients, but showed no correlation with use of Dexamethasone.

**Conclusions**

Wound infiltration with 4 mg of Dexamethasone may decrease opiate consumption post thoracic surgery in our examined population, with no negative effect on wound healing. However larger numbers are needed to validate these results.

### A236 The Surgical Management of COVID-19 Pulmonary Complication in Patients Requiring Extracorporeal Membranous Oxygenation (ECMO)

#### Law, Jacie Jiaqi, Dr; Soh, Karen Chien Lin, Ms; Aresu, Giuseppe, Mr; Coonar, Aman, Mr; Aresu, Giuseppe, Mr

##### Royal Papworth Hospital NHS Foundation Trust, Cambridge, UK

*Journal of Cardiothoracic Surgery* 2023, **18(Supp 1)**:A236

**Objectives**

COVID-19 induces capillary microthrombi and pulmonary infarction, mediating complications including pneumothoraces, necrotizing pneumonia and pulmonary haemorrhage. High mortality exists in COVID 19 patients requiring ECMO rescue therapy. Concurrently, it is recognized that high postoperative mortality exists in COVID-19 patients undergoing thoracic surgery. In this case series, we aim to explore the surgical management and post-operative outcomes in a patient cohort with the highest COVID-19 disease severity.

**Methodology**

From November 2018 to November 2021, we identified five patients fulfilling the inclusion criteria. 4 male and 1 female patient was included. The Mean age in this study was 44 years old with a median Charlson Comorbidity Index of 1. Patient demographic and operative data were retrieved from the database. Electronic patient records and ECMO database was also reviewed to obtain data on complications and survival.

**Results**

The Mean time from COVID positivity to ECMO cannulation was 8.2 days. All patients were commenced on veno-veno ECMO. 3 out of 5 patients presented with haemothorax necessitating emergency right-sided anterolateral thoracotomy. 1 right-sided salvage lower lobectomy was performed for a case of COVID-19 induced necrotizing pneumonia. Contrasting to a predominantly pulmonary haemorrhagic phenomenon pre-operatively, 80% experienced thromboembolic states post-operatively (cerebral infarct and ECMO cannulation site thrombosis). Goursaud et al. aptly describes the dilemmas of ECMO heparinisation in the context of surgical management of COVID lung complications. 2 out of 5 fatalities occurred in patients characterised by higher Charlson index pre-operatively.

**Conclusion**

To the best of our knowledge, this is the first case series to analyse patient characteristics, surgical managements, post-operative complications and survival on cases of severe COVID-19 pulmonary complications in the ECMO subgroup.

### A237 Development and Evaluation of a Novel VATS Endoscopic Camera System

#### Whittaker, George, Dr; Kogkas, Aleandros, Dr; Mylonas, George, Dr; Hanna, George, Prof

##### Imperial College London, London, UK

*Journal of Cardiothoracic Surgery* 2023, **18(Supp 1)**:A237

**Objectives**

We aimed to develop a novel VATS endoscopic camera system to enhance the field of view, allowing for increased surveillance and earlier identification of complications. Additionally, we aimed to quantitatively and qualitatively evaluate the prototype VATS camera with a comparative study and participant questionnaires.

**Methods**

We developed a final prototype from a USB camera module with a fisheye lens. Images from this sensor were captured, undistorted, and projected onto a curved screen in real-time. A prospective comparative study was conducted with surgical trainees from Imperial College London as participants. Each participant completed a psychomotor task using prototype and conventional endoscopic systems, followed by an evaluation questionnaire. Outcome measures were assessed with Wilcoxon signed-rank tests.

**Results**

Our prototype accomplished a 118-degree field of view at a resolution of 1260 × 708 pixels, though with a compromise of 7 frames per second frame rate and 231 ms latency when distortion correction was active. Participants identified simulated bleeding significantly faster (*P* = 0.0313) with no camera movements (*P* = 0.0350) compared to a conventional endoscope, although task completion time did not differ (*P* = 0.2188). Qualitative data highlighted benefits of the enhanced field of view and concerns with frame rate and latency.

**Conclusions**

We successfully developed and assessed a unique wide-angle VATS endoscopic camera. Our system shows promising results as a potentially superior alternative to current systems. Further work needs to focus on resolving frame rate and latency issues, which could be achieved by reducing resolution.

### A238 Drawbacks of Powered Air-purifying Respirators During COVID-19 Pandemic—How Voice Amplifiers Improve Communication and Outcomes During Resuscitation

#### Kutywayo, Kudzayi, Mr; Kubiak, Krzysztof, Dr; Korre, Sofia, Dr; Karia, Chiraag, Mr; Annamaneni, Rajani, Dr; Rathinam, Sridhar, Mr

##### Glenfield Hospital, University Hospitals of Leicester NHS Trust, Leicester, UK

*Journal of Cardiothoracic Surgery* 2023, **18(Supp 1)**:A238

**Background**

The COVID-19 pandemic has caused massive restructuring in policies regarding use of personal protective equipment in aerosolised environments within the hospital. Use of powered air purifying respirators has allowed health provision to continue safely in the wake of a novel droplet infection (SARS-CoV-2). A trade-off for safety is, unfortunately, the impediment in communication. We sought to investigate whether portable audio amplification equipment would help during cardiopulmonary resuscitation (CPR) in patients with COVID-19.

**Objective**

To evaluate the usefulness of portable electronic voice amplification units during resuscitation.

**Methods**

Teams consisting of 4 members each were evaluated as they ran through 2 Advanced life support (ALS) cardiac arrest simulation scenarios. One of the scenarios was performed whilst participants were using a portable electronic voice amplification unit (VAU). A survey after each scenario was conducted. Video and audio feedback was obtained. Time taken to arrive at critical points in each scenario was assessed. Verbal consent was obtained from all participants to record the simulation and the feedback.

**Results**

83.3% of the participants found it difficult to communicate with fellow members of the resuscitation team owing to the respiratory personal protective equipment. 91.7% of participants found the voice amplifier either moderately or significantly better in improving the quality of communication. Consequently, critical time points were reached quicker when resuscitation was carried out with voice amplification. Most participants felt the time added during donning by applying the voice amplifier was not detrimental when entering a COVID resuscitation area.

**Conclusion**

Use of VAUs improve communication in cardiac arrest and ultimately may help to improve resuscitation outcomes. Further study is needed, although initial results are encouraging.

### A239 Denepuncture and Requested Investigations Amongst Thoracic Surgery Patients: Service Evaluation and Cost Analysis

#### Gopalaswamy, Madhura, Dr; Calvert, Rachel, Mrs; Connelly, Leanne, Mrs; Dunning, Joel, Mr; Waterhouse, Benjamin, Mr

##### South Tees NHS Foundation Trust, Middlesbrough, UK

*Journal of Cardiothoracic Surgery* 2023, **18(Supp 1)**:A239

**Objectives**

Blood tests are a ubiquitous component of inpatient care and have been since the late 1800’s.

Resources within the NHS are finite, and this has never been more apparent.

When Beckton Dickinson (BD), manufacturers of our most commonly used blood tubes announced a global shortage of its products in August 2021, it further emphasised a need for focussing our usage of this resource.

We hypothesised that some tests requested and even some venepuncture episodes were unnecessary.

**Methods**

A retrospective service evaluation was performed cross-referencing the medical notes, pathology requests, and available results to assess appropriateness of each test in Thoracic Surgery Inpatients.

Estimated costs for each test were taken from NHS Reference Costs and from the trust pathology lab.

**Results**

We found an *average of 2.8 sets* were sent for a 4-day inpatient stay.

*Only 35% of requests included a valid indication*

*95% included additional investigations without justification*, common examples included Urate, Liver Function Tests, C-reactive Protein, and Lipid Profile.

*An average of 4 additional investigations were requested per sample* resulting in a **predicted unnecessary cost of £69.82** per patient.

**Conclusions**

Significant savings can be achieved by a change in local policy and staff education.

By extension, this could protect staff time and safeguard patients from unnecessary, if minor, invasive procedures.

## Thoracic Oncology

### A240 Anatomical Segmentectomies In A Universal Uniportal VATS Thoracic Centre During Covid-19 Pandemic: Evaluation of Outcome

#### Fang, Chen Chuan, Mr; Martin-Ucar, Antonio, Mr; Hernandez, Luis, Mr

##### University Hospital Coventry and Warwickshire, Coventry, UK

*Journal of Cardiothoracic Surgery* 2023, **18(Supp 1)**:A240

**Table:** 

	Group A (n = 33)	Group B (n = 47)	Group C (n = 26)	Total (n = 106)
Age (Median)	74	72	71.5	72
Age (Range)	50	43	36	51
Gender (n) Male	19	23	12	54
Gender (n) Female	14	24	14	52
FEV1 (%) Median, Range	82, 76	84, 108	78, 63	82.5, 108
TLCO (%) Median, Range	77.5, 73	87, 77	77, 95	80.5, 95
Convertion to thoracotomy (n)	2	0	0	2
Respiratory Complication (%)	3.0 (P > 0.05)	3.2 (P > 0.05)	2.8 (P > 0.05)	3.0 (P > 0.05)
Length of stay (days)	5.5 (P > 0.05)	5.0 (P > 0.05)	4.1 (P > 0.05)	4.8 (P > 0.05)

**Objectives**

Our aim is to determine the feasibility, effectiveness, safety and surgical outcomes of UVATS segmentectomies performed during Covid-19 pandemic.

**Methods**

All patients who received UVATS segmentectomy for treatment of lung cancer or benign diseases from the overall thoracic surgical activity from January 2019 to July 2021 were identified. They were divided into 3 groups: 2019(pre-pandemic), 2020(peak) and 2021(recovery). All cases adopted Covid-free measures which include testings, isolation, PPE and Covid-free colour-coded zoning with controlled access from preoperative preparation to post-operative care.

**Results**

A total of 106 patients underwent UVATS segmentectomy over the study period were grouped by years: Group A (January–December 2019), Group B (January-December 2020) and Group C (January–July 2021). Demographic and results of the 3 periods are presented in Table 1.

Histology findings reported 27 non-malignant cases and 79 malignant cases. Postoperative 30-days in-hospital mortality was zero and none of the patients required HDU/ITU care post-operatively. No patient was infected with Covid-19 throughout their hospital stay.

**Conclusion**

By adapting to the crisis and optimizing the skills with resources available, we were able to perform more cases of UVATS segmentectomies during the pandemic, effectively and safely.

### A241 Laser Pulmonary Metastasectomy—Movie

#### Chandarana, Karishma, Dr; Caruana, Edward, Mr; Weaver, Helen, Miss; Rathinam, Sridhar, Mr; Nakas, Apostolos, Mr

##### Glenfield Hospital, Leicester, UK

*Journal of Cardiothoracic Surgery* 2023, **18(Supp 1)**:A241

https://www.youtube.com/watch?v=yOUIc-CqzoE

### A242 Long-term Results From Uni-portal VATS Segmentectomies

#### Ariyaratnam, Priyad, Dr; Edwards, John, Dr; Rao, Jagan, Dr; Tenconi, Sara, Dr; Komber, Mohamed, Dr; Agrawal, Sanjay, Dr; Socci, Laura, Dr

##### Sheffield Teaching Hospitals, Sheffield, UK

*Journal of Cardiothoracic Surgery* 2023, **18(Supp 1)**:A242

**Objectives**

Segmentectomies have become a popular method to both diagnose and definitively treat early stage lung tumours and this in parallel to the increased interest in uni-portal VATS surgery to treat early-stage lung tumours. However, little data exists on the outcomes of uni-portal segmentectomies for lung tumours. We therefore wanted to evaluate our long-term outcomes using this technique for early stage tumours.

**Methods**

We performed 173 VATS anatomical segmentectomies between April 2015 and April 2021 for patients with suspected cancer. We matched these with uniportal VATS lobectomies using a 1:1 propensity matching algorithm.

**Results**

The mean age at surgery was 69.7 years and the percentage of males was 45.5% for segmentectomies. The mean predicted FEV1 was 91.36% and the mean predicted DLCO was 73.3%. Left-sided tumours formed the majority of resections (78%). 26% of the segmentectomies were performed by trainee surgeons under the supervision of a consultant surgeon. Upper trisegments (38.7%) formed the majority of resections. The mean tumour size was 23.8 mm. Adenocarcinomas formed the majority of tumours resected (46.8%) whilst metastases formed 9.4%. Of those that were primary tumours (N = 124), 54.% were T1 and 41.1% were T2. The mean duration of surgery was 138.9 min and the mean length of hospital stay was 5.2 days (± 4.1). There was 1 conversion to a thoracotomy. There was 1 30-day mortality. The survival analysis showed that mean 5-year survival for all tumours was 71% (± 4.9). When compared to the matched lobectomy group for T1 tumours, the segmentectomy group was 74% whilst the lobectomy group was 45% at 5 years (log rank, p = 0.02). There were 2 R1 margins at final histology. There were 2 instances of documented tumour recurrence in the segmentectomy group.

**Conclusion**

Uniportal VATS segmentectomies can be safely utilised for early-stage lung tumours without compromising long-term outcomes.
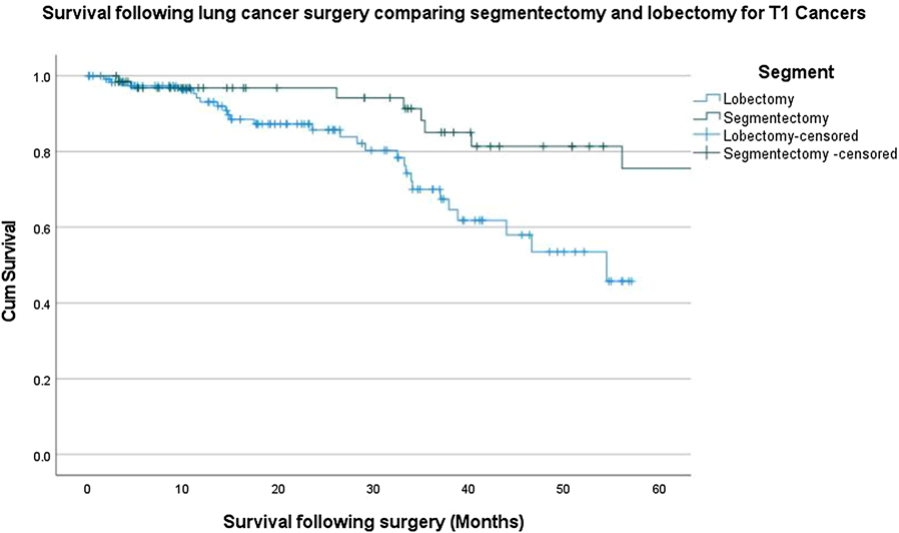

**Table:** 

Segment	Frequency
Trisegment	38.7%
S6	26%
Lingula	14,5%
Basal	11.6%
S2	4.6%
S3	1.2%
S1 & S2	1.2%
S7 & S8	0.6%
Other	1.2%

### A243 Is There a Future for Radical Mesothelioma Surgery after MARS2?

#### Lee, Michelle, Miss; Nardini, Marco, Mr; Hargrave, Joanne, Miss; Waller, David, Mr

##### Barts Thorax Centre, London, UK, St Bartholomew's Hospital, London, UK

*Journal of Cardiothoracic Surgery* 2023, **18(Supp 1)**:A243

**Objectives**

The MARS2 trial completed recruitment in Jan 2021 but the results will not be released until 2023. As the BTS guidelines state that radical surgery for malignant pleural mesothelioma (MPM) should not be conducted outside of a trial does this mean there is a moratorium on this operation until the trial reports? We assessed our surgery for MPM during and since the MARS2 trial to answer these questions.

**Methods**

In a retrospective analysis we analysed 45 consecutive patients discussed at the Mesothelioma MDT undergoing macroscopic complete resection radical surgery for MPM at Barts under single surgeon. All patients and by extended pleurectomy decortication (PD) or PD. Comparison of historical cohorts of 15 patients before and after closure of MARS2 trial. We compared referral source, speed of recruitment, patient demographics and pathology results. We cannot report on survival data.

**Results**

During MARS2 we recruited the last 15 patients (14 M:1F) for surgery in an overall period of 453 days. In this period, we operated on the last 15 patient (12 M:3F) outside of the trial in 372 days. Reasons for non-MARS2: 5 ineligibles (2 concurrent lung; 3 not considered initially pre chemotherapy); 5 patient choices; 5 due to COVID). Post MARS 2: the first 15 patients were operated upon in 224 days.

The proportion of referrals from local or distant sources has not changed since MARS 2 but 3 large referring academic centres have not referred.

**Table:** 

Median (Range)	MARS2 Period: MARS2	MARS2 Period: Non-MARS2	Post-MARS2	P Value
Age (Year)	72 (57–78)	64 (49–77)	67(46–78)	NS
BMI (Kg/m2)	28 (18–37)	26 (17–33)	26 (20–35)	NS
FEV1 (%Predicted)	65 (38–108)	83 (75–90)	63 (59–90)	NS
DLCO (%Predicted)	66 (48–79)	84 (69–98)	57 (51–98)	NS
Pre-Operative: Induction Chemotherapy	15/15 (100%)	13/15 (87%)	4/15 (27%)	0.0366
Pre-Operative: Number of Chemotherapy Cycles	2 (2–2)	3 (0–4)	2 (2–2)	NS
Extended Pleurectomy Decortication (PD):PD	12:3 (80%:20%)	9:6 (60%:40%)	9:6 (60%:40%)	NS
Post-Operative Pathology (Epithelioid:Non-Epithelioid)	10:5 (67%:33%)	8:7 (53%:47%)	14:1 (93%:7%)	0.0484
Post-Operative Pathology: Node Positive	6/15 (40%)	6/15 (40%)	4/15 (27%)	NS

**Conclusion**

Radical surgery continues after MARS2 at an increasing rate from established sources. There is a trend towards upfront less extensive surgery in more epithelioid disease.

### A244 Discordance in Cell Type after Pleurectomy/Decortication for Malignant Pleural Mesothelioma—A Possible Detrimental Effect of Induction Chemotherapy

#### Lee, Michelle, Miss; Baranowski, Ralitsa, Miss; Hargrave, Joanne, Miss; Waller, David, Mr

##### Barts Thorax Centre, London, UK, St Bartholomew's Hospital, London, UK

*Journal of Cardiothoracic Surgery* 2023, **18(Supp 1)**:A244

**Objectives**

The prognosis following pleurectomy/decortication (PD) for resectable malignant pleural mesothelioma (MPM) is known to be dependent on histological cell type. We aimed to evaluate the accuracy of preoperative assessment of histological cell type, factors affecting its accuracy and the subsequent effect on postoperative survival after PD. We aimed to identify possible improvements in preoperative workup.

**Methods**

We analysed the perioperative course of 122 patients [103 M:19F, Age 68 (33–79) years] who underwent either PD (26 patients) or extended PD (96 patients). Induction chemotherapy was given to 94 patients while 28 patients had upfront surgery. We recorded discordance between preoperative and postoperative histological findings and looked at predictive factors and survival implications.

**Results**

**Table:** 

n	Cell Type Concordance	Cell Type Discordance: Positive	Cell Type Discordance: Negative	Total	P Value
**Initial Treatment**	83	2	28	113	
Induction Chemotherapy	58	1	26	85	0.0291
Primary Surgery	25	1	2	28	
**Biopsy Method**	Cell Type Concordance	Cell Type Discordance			
Video-Assisted Thoracoscopic Surgery (VATS)	51 (69.9%)	22 (30.1%)		73	NS
Local Anaesthetic Thoracoscopy (LAT)	15 (57.7%)	11 (42.3%)		26	NS
Percutaneous	17 (60.7%)	11 (39.3%)		28	NS
**Disease Extent**: Tumour Thickness (mm)	13 (3–66)	16 (5–35)			NS
**Disease Extent**: N1	42/83 (50.6%)	20/30 (66.7%)			NS

Perioperative cell type discordance was not associated with the method of biopsy nor the extent of disease but was significantly associated with the use of induction chemotherapy.

There is currently no significant difference in survival from date of diagnosis in those who received either surgery or chemotherapy first: surgery 31 (95% CI 23.6–41.1) months vs chemotherapy 21 (95%CI 25.5–36.9) months, (p = 0.3).

**Conclusions**

We suggest a need to reconsider the routine use of induction chemotherapy in the treatment protocol in otherwise resectable MPM. If used then re-biopsy should be used to exclude biphasic disease before radical surgery to maximise postoperative survival.

### A245 Clinical Outcome of Limited Resection in Peripheral Small-sized Non-small Cell Lung Cancer: A Systematic Review

#### Abbas, Mohammed, Mr; Hashmi, Faisal, Mr; Taylor, Marcus, Mr

##### Wythenshawe Hospital, Manchester, UK

*Journal of Cardiothoracic Surgery* 2023, **18(Supp 1)**:A245

**Objectives**

Lobectomy is considered the standard surgical approach for operable non-small lung cancer (NSCLC). However, limited resection (LR) is becoming a preference in small, peripheral early stage NCSLC. Lung cancer screening programs combined with advanced computed tomography increase the likelihood of detecting small sized peripheral lung cancers. There is no consensus on whether limited resection is superior to lobectomy. To address this issue, the current study aims to assess the 5-year overall survival (OS) and recurrence-free survival (RFS) in patients who underwent limited resection for early stage NCSLC.

**Methods**

A systematic review of literature was performed using four online databases (Embase, PubMed, MEDLINE and Cochrane Library databases) according to predefined selection criteria to identify all relevant articles. We reviewed all available articles from their date of inception until January 2020. The overall survival and recurrence-free survival in limited resection group was evaluated to determine whether limited resection is satisfactory in treating small, peripheral early-stage NSCLC.

**Results**

A total of twelve studies were met the inclusion criteria for the systematic review, including a total of 2256 patients (868 patients underwent LR and 1388 patients underwent lobectomy). The mean duration of the studies was 8.25 years. The tumor size was between 0.5 to 3 cm. Overall, limited resection was associated with OS and RFS of 41 – 100% and 59.4–100%, respectively.

**Conclusion**

The current systematic review suggests that for selected patients limited resection is feasible for selected patients with early stage NSCLC and tumors < 3 m cm and located peripherally. From available data, the post-operative OS at 5-year interval and RFS rates appear to be comparable to lobectomy. Further prospective RCTs are needed to confirm these findings.

### A246 Preservation of Functional Status post Manubrial Resection for Chest Wall Sarcoma: A Single-Centre Retrospective Analysis

#### Shatila, Mohamed, Mr; Khor, Bo, Mr; El-Gamal, Islam, Mr; Khalil, Haythem, Mr; Patel, Akshay, Mr; Kalkat, Maninder, Mr

##### Queen Elizabeth Hospital, Birmingham, UK

*Journal of Cardiothoracic Surgery* 2023, **18(Supp 1)**:A246

**Introduction**

Approximately 30% of malignant, primary bone tumours are chondrosarcomas, which occur on the anterior chest wall most frequently. Patients who are treated with adequate surgical intervention tend to recover well and survival at 10 years is as high as 97%. In select cases, resection of the manubrium is warranted and aside from the aesthetic outcome, the impact on chest wall mechanics, functional status, and preservation of respiratory efficiency and loading of the chest wall are key post-operative aspects which need to be considered when undertaking radical resections.

**Methods**

We collected demographic, operative and post-operative pathological data on all patients as well as an objective assessment of functional status post-operatively using the MRC and Karnofsky grading systems. Median follow-up was 1864 days (30–4984 days).

**Results**

Twelve patients underwent manubrial resection for chest wall sarcoma between 2008 and 2021. Median pre-operative ECOG status was 0 (0–3) and MRC score was 1. Post-operatively, patients were mainly limited by pain at the operative site, however functional status was preserved if not improved in most cases (post-op median MRC score was 0) (p = NS). Median length of post-operative stay was 9 days (3–31). Fifty percent of all cases were chondrosarcoma. Overall survival was significantly improved in the chondrosarcoma cohort (p = 0.0043). Adjuvant therapy was administered to 25% of the cohort (n = 3), and recurrence occurred in 1 patient at 12 months post-operatively.

**Conclusions**

Manubriosternal resection is the best treatment modality for anterior chest wall sarcomas. It can be carried out safely with few post-operative complications. Overall survival at the maximum follow-up of 13.6 years was around 80% for chondrosarcomas. The alteration in chest wall geometry and respiratory mechanics did not result in a significant decline in post-operative functional status in these patients.
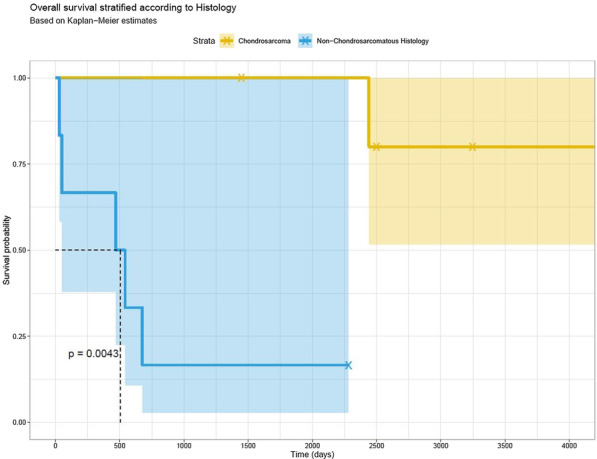


### A247 Uniportal Non-intubated SVATS Thymectomy Compared to Uniportal Intubated SVATS Thymectomy: The Technical Feasibility, Enhanced Recovery and Safety

#### Bushra, Raisa^1^, Mrs; Nizami, Maria^2^, Miss; Hogan, John^1^, Mr; Williams, Luke^1^, Mr; Peryt, Adam^1^, Mr; Coonar, Aman^1^, Mr; Aresu, Giuseppe^1^, Mr

##### ^1^Royal Papworth Hospital NHS Foundation Trust, Cambridge, UK; ^2^Guy’s and St Thomas’ NHS Foundation Trust, London, UK

*Journal of Cardiothoracic Surgery* 2023, **18(Supp 1)**:A247

**Objectives**

Subxiphoid video-assisted thoracoscopic surgery (SVATS) has been associated with less pain and subsequent opioid requirements. Furthermore, a non-intubated approach reduces sedatives and opioids required and may also enhance recovery. The current retrospective comparative study is aimed to compare the technical feasibility, enhanced recovery, safety, and adequacy of oncological resection in patients undergoing non-intubated subxiphoid video-assisted thoracoscopic thymectomy and in patients who underwent a traditional intubated subxiphoid resection.

**Methods**

We conducted a retrospective study of 42 patients who underwent Subxiphoid VATS thymectomy from September 2016 until June 2021. Among them 21 carefully selected patients underwent non-intubated SVATS thymectomy. Patients were selected for the study and were matched with regards to age, gender, comorbidities, smoking history.

**Results**

A total of 42 patients were included in the analysis of which the mean age was 59.6 years, and 52% were female. Mean age for the non-intubated group was 57.5 years and 54.5% female. Among them 12 patients had myasthenia gravis. Major complications were bleeding, mandating conversion, acute kidney injury, prolonged air leak and vocal cord palsy. In total for Group 2, there were 3 cases converted to sternotomy, compared to Group 1. Subsequently, there were two admissions to the Intensive Care Unit for Group 2 and one admission due to AKI for Group 1. The median hospital length of stay for both groups was 2 days. Complete resection was achieved in all cases in Group 1 whereas in Group 2, 4 cases were reported with R1 resection margin.

**Table:** 

Traits	SVATS non-intubated (Group 1)	SVATS intubated (Group 2)
Length of Hospital Stay (mean)	2	2
Conversion to sternotomy	1/21	3/21
ICU admission	1	2
Operating time (mean)	2 h 32 min	3 h 59 min
Resection Margin R0	100%	71.4% ( R1 = 4 patient)

**Conclusion**

Non intubated subxiphoid thymectomy is technically feasible, safe and associated with adequate oncological resection.Reduced sedation and opioid requirement lower the risk of post-operative respiratory failure and delirium thus accelerate enhance recovery. However, larger studies are required to confirm this hypothesis.

### A248 Introduction of Macmillan Community Thoracic Specialist Nurse

#### Stockdale, Stacey, Mrs; McNaught, Hayley, Mrs; Calvert, Rachel, Mrs; Connelly, Leanne, Mrs

##### South Tees Hospitals NHS Foundation Trust, Middlesbrough, UK

*Journal of Cardiothoracic Surgery* 2023, **18(Supp 1)**:A248

The Thoracic Surgical service cares for around 800 patients/ year, serving a population of 1.5million covering North Yorkshire, Teesside and County Durham who undergo thoracic procedures for diagnostic, therapeutic or palliative intent for both benign and malignant disease. The introduction of Community Thoracic Specialist Nurse who can visit the patient at home 24–48 following discharge, allowing a nurse with specialist knowledge in relation to thoracic surgery to review the patient, manage symptoms, provide reassurance and support, whist facilitating earlier discharge from hospital and reducing readmissions. We know that patients and their relatives have experienced heightened levels of anxiety relating to attending hospital and having to undergo treatment for cancer, we believe there is an increased need to protect patients requiring thoracic surgery.

We have shown a significant reduction in length of stay to average of 4.1 compared to national average of 6.6 days (GIRFT data) and we believe over the coming year this could be reduced further as LOS has increased by 1 day throughout COVID due to no Day of Surgery admissions. Our initial data shows a reduction in readmissions to 11% from 29 and 36% (VIOLET study VATS vs OPEN lobectomy). We identified appropriate patients who required readmission, issues were recognised during visit, bloods and COVID swab taken, we were able to readmit directly to our ward avoiding high-risk COVID areas such as A&E, AAU and outlying hospitals. Patient experience feedback has been overwhelmingly positive with 50% response rate. Patients have told us how valuable they feel this service is in terms of reassurance and support.

Since introducing this service we have constantly adapted to change and surpassed all of our initial expectations and achieved our initial goals in improving patient experience, reducing length of stay and reducing readmission rates.

### A249 Current UK Practice in the Management of Patients with Pulmonary Neuroendocrine Tumours

#### Mehdi, Rana, Miss; Steyn, Richard, Mr; Kalkat, Maninder, Mr; Fallouh, Hazem, Mr; Naidu, Babu, Mr; Bishay, Ehab, Mr; Shah, Tahir, Dr; Rogers, Vanessa, Ms

##### Queen Elizabeth Hospital Birmingham, Birmingham, UK

*Journal of Cardiothoracic Surgery* 2023, **18(Supp 1)**:A249

Pulmonary neuroendocrine tumours [NETs] are increasing in incidence. There is growing understanding of the multidisciplinary management of these patients. We wanted to explore the current national management of pulmonary neuroendocrine tumours by means of a survey to determine the scope of practice in relation to current guidance.

An online survey was created using SmartSurveyTM. The questionnaire was based on the 2015 European Neuroendocrine Tumour Society expert consensus for best practise for typical and atypical pulmonary NETs and the updated 2021 Lung and Thymic Carcinoids: ESMO Clinical Practise Guidelines for diagnosis, treatment and follow up. It was disseminated to all UK Thoracic Surgery Centres. The results were analysed to understand scope and variation of practise across the UK in patient management, surgical technique and follow up.

Responses were received from 17 UK Thoracic Surgery units. All centres reported awareness of guidelines; however, only 63% of respondents reported using them routinely in their clinical practise. 74% of respondents reported access to a specialist Neuroendocrine multidisciplinary team. Surgical resection techniques for typical and atypical peripheral tumours varied between centres. 84% of respondents favoured anatomical resection for peripheral carcinoid tumours, however 47% stated their strategy would change with atypical versus typical carcinoid tumours. 95% of respondents reported following International Association for the Study of Lung Cancer recommendations for lymph node dissection/sampling. Follow up procedures varied greatly between centres, with regard to who conducted follow up, the frequency and duration.

There is widespread awareness of the current guidelines with regard to the management of Pulmonary Neuroendocrine tumours. Despite this knowledge, practice is varied. More work is needed to promote joint management planning between the Lung and NET teams and improve the use of accepted guidelines.

### A250 Are Chest Drains Routinely Required After Thoracic Surgery?

#### Proli, Chiara, Miss; Abdul Khader, Ashiq, Dr; De Sousa, Paulo, Mr; Pons, Aina, Dr; Alshammari, Abdullah, Dr; Palmares, Abigail, Ms; Booth, Sarah Ann, Mrs; Leung, Maria, Mrs; Lim, Eric, Prof

##### Royal Brompton and Harefield Hospital, London, UK

*Journal of Cardiothoracic Surgery* 2023, **18(Supp 1)**:A250

**Background**

A principal bottleneck for early discharge is the presence of a chest drain after routine elective thoracic surgery. The aim of this study is to report the technique and outcomes of on-table drain removal for selected thoracic surgical procedures to facilitate day-case surgery.

**Methods**

A 5-year retrospective analysis of protocolised on-table drain removal for pleural (pneumothorax and effusions), mediastinal, pericardial, and selected lung (wedge resection) surgery, excluding lung volume reduction. Single port VATS was the standard approach and drains removed at the end of the procedure on confirmation of air leak of < 20 ml/hour by digital drain. Data on post-drain removal pneumothorax, effusion and need for further intervention were obtained by formal radiology reporting of post-procedure chest films.

**Results**

Between 2016 and 2021, we operated on 617 patients, of which 107 (17%) patients had drains removed on-table in theatre with a mean age (SD) of 58 (17) years of which 54 (51%) were male. The majority of the procedures were pulmonary wedge resections in 43 patients (40%) and pleurodesis in 23 (22%).

Post-drain removal pneumothorax occurred in 22 patients (21%), pleural effusion in 6 (5.6%). Drain reinsertion was required in 1 patient (0.9%) after pleurodesis for pneumothorax. The median (IQR) length of hospital stay was 1 day (1–2) and 14 patients (13%) discharged on the day of surgery.

**Conclusions**

Routine chest drains are not required after thoracic surgery. On table chest drain removal can be safely achieved in selected procedures paving the way for day case thoracic surgery.

### A251 Experiences of Healthcare Professionals in Surgical Oncology During the COVID-19 Pandemic: A Qualitative Study

#### Kapur, Alanah^1^, Miss; Shah, Salonee^1^, Miss; Bekker, Hilary^1^, Prof; Boele, Florien^2^, Dr; Young, Alistair^2^, Mr; Pompili, Cecilia^2^, Dr

##### ^1^University of Leeds, Leeds, UK; ^2^St James Hospital, Leeds, UK

*Journal of Cardiothoracic Surgery* 2023, **18(Supp 1)**:A251

**Objectives**

COVID-19 has been a burden for healthcare systems globally. Within the UK, the NHS faced new pressures, including burden on intensive care units, staff redeployment and delays or cancellation of elective cancer procedures. This study explores the experiences of healthcare professionals to investigate the impact of COVID-19 on decision-making in surgical oncology.

**Methods**

In this service evaluation, participants with relevant professional experience were recruited using purposive sampling. Semi-structured qualitative interviews were recorded and transcribed verbatim. Thematic analysis was used.

**Results**

Thirteen participants were interviewed, and seven themes identified. The majority described increased discussions regarding patient prioritisation during multi-disciplinary team meetings. Concerns were expressed about telephone-based pre-operative assessments with limited examination of patients prior to surgery. Participants experienced increased workload and responsibilities throughout the pandemic, although this was not perceived to influenced patient-centred decisions. Generally, participants experienced various stresses which were not thought to hinder clinical performance.

**Conclusion**

Interviews with healthcare professionals highlighted that COVID-19 has influenced clinical decisions in surgical oncology. Although changes to patient pathways were highlighted, the delivery of care was not perceived to be affected. Future research should explore the COVID-19 related changes remaining in surgical oncology and how they affect the patients’ experience of care.

### A252 5 Year Audit of Pericardial Effusion Management in a Tertiary Thoracic Unit

#### Eckersley, Martyn^1^, Dr; Baranowski, Ralitsa^2^, Ms

##### ^1^Glasgow Royal Infirmary, Glasgow, UK; ^2^St Bartholomew's Hospital, London, UK

*Journal of Cardiothoracic Surgery* 2023, **18(Supp 1)**:A252

**Objectives**

We aimed to investigate management of chronic large pericardial effusions referred to a tertiary thoracic centre over a five-year period and if these complied with the ESC guidelines.

**Methods**

A retrospective audit looking at whether referrals in a five-year period between 2015 and 2020 was performed. Records were investigated by one of the authors, basic demographic information and length of stay were recorded. Indications for intervention, previous pericardiocentesis, whether the patient was in tamponade at time of referral and specific operation that took place as well as final histology result were recorded.

**Results**

133 cases of pericardial effusion were referred to the thoracic surgery service. Of those accepted (100) average age was 51, average length of stay 10.6 days (range 2–57). Seven patients referred were in tamponade, six accepted under the care of the Thoracic team, one to cardiology for temporising pericardiocentesis.

Most underwent Video Assisted Thorascopic Surgery (VATS) (51%), followed by anterior minithoracotomy (38%). Most common indications for intervention were recurrence (30), the need for a tissue diagnosis (13), and diagnosis of malignant effusions (18). Complications experienced included re-accumulation, fast AF and post-operative nausea and vomiting.

33 referrals were not accepted. The most common reasons were not being fit for general anaesthesia or in tamponade (48%). Second was patients redirected to cardiology for consideration of percutaneous drainage first (33%).

**Conclusions**

Good compliance with ESC guidelines regarding management of chronic pericardial effusions was demonstrated, although 25% of referrals were redirected to a different service. A limitation of this audit is that the data is only from those referred to the thoracic team. The introduction of a clear clinical pathway for the management of chronic pericardial effusions to ensure patients get the necessary management promptly, per ESC guidelines, is recommended.

### A253 Is a 6-month Follow-up CT Scan After Lung Resection for Primary Lung Cancer Necessary?

#### Philip, Bejoy, Mr; Basak, Bappy, Dr; Tariq, Humaira, Dr; Shackcloth, Michael, Mr

##### Liverpool Heart and Chest Hospital, Liverpool, UK

*Journal of Cardiothoracic Surgery* 2023, **18(Supp 1)**:A253

**Objectives**

Despite the lack of high-quality evidence, most patients following lung cancer resection are followed up with CT scans. The interval of these CT scans varies between guidelines and institutions. We sought to determine the value of performing a CT scan at 6-months.

**Methods**

The CT scan reports and clinical details of 132 patients who underwent lung resection at our institution, between February and June 2018 were analysed.

**Results**

Out of 132 patients, 69 patients had a CT scan and 12 had X-rays at 6-months. 10 patients had recurrence identified on CT scans while 49 patients had no recurrence, and 10 had indeterminate findings. 12 had no recurrence on a chest x-ray. Out of the 61 patients with normal radiology at six months, only three had a recurrence on the CT scan at one year. Out of 10 patients who had indeterminate findings on the 6-month scan 5 proved not to have recurrence, two had a recurrence and 1 had a 2nd lung primary. Two died of unrelated causes. Recurrence of cancer identified on the 6-month CT scans did not appear to be related to the tumour stage, which might due to the adjuvant chemotherapy received by patients with higher tumour staging.

**Conclusions**

A CT scan at six months appears to be valuable at picking up recurrence, with few indeterminate findings. The recurrence rate on the yearly Ct scan was low if the initial CT or CXR at six months was normal, questioning the value of a scan at a year.

### A254 Lung Cancer Resection in the Absence of Pre-operative Histology: The Accuracy of Multidisciplinary Team Consensus

#### Whooley, Jack, Dr; Weedle, Rebecca, Dr; White, Alexandra, Dr; Breen, David, Dr; Soo, Alan, Mr

##### University Hospital Galway, Galway, Ireland

*Journal of Cardiothoracic Surgery* 2023, **18(Supp 1)**:A254

**Objectives**

Lung resection remains the gold-standard of treatment for non-small cell lung cancer (NSCLC). British Thoracic Society (BTS) guidelines recommends the pursuit of pre-operative histological diagnosis and staging where possible. In the absence of pre-operative histology, surgical treatment can be offered in conjunction with multidisciplinary team (MDT) and patient consensus. We aimed to perform a single-centre analysis of the accuracy of the thoracic MDT in recommending surgical resection for those patients with suspected NSCLC in the absence of pre-operative histological diagnosis over a five-year period.

**Methods**

A retrospective review was performed of patients undergoing lung resection at the recommendation of the thoracic MDT for suspected NSCLC in our unit between May 2016 and August 2021. Patients with confirmed histological diagnosis were excluded from analysis.

**Results**

234 patients underwent lung resection without pre-operative histology in the five-year period. 54.6% were female, mean age was 67.4 years. Overall, the positive predictive value of the MDT team consensus for lung malignancy in the absence of pre-operative histology was 88.9%. Of the 208 patients with confirmed malignancy on post-operative histology, this consisted primarily of NSCLC (70%), metastatic disease (17%) and carcinoid tumours (5%.) 26 patients had benign histology post-operatively, with the most common benign histology consisting of benign hamartomas (19%), organizing pneumonia (15%), benign scar tissue (15%) and granulomas (11%.)

**Conclusion**

In the absence of pre-operative histology, lung resection of suspected NSCLC is reasonable if performed in conjunction with multidisciplinary team and patient consensus, in keeping with the British Thoracic Society Guidelines.

### A255 Pre-operative Prognostic Factors for 5-year survival Following Pulmonary Metastasectomy from Colorectal Cancer. A Systematic Review and Meta-analysis

#### Gkikas, Andreas^1^, Dr; Kakos, Christos^2^, Mr; Lampridis, Savvas^3^, Mr; Godolphin, Peter^1^, Dr; Patrini, Davide^4^, Mr

##### ^1^MRC Clinical Trials Unit, UCL, London, UK; ^2^Royal Victoria Hospital, Belfast Health & Social Care Trust, Belfast, UK; ^3^Guy's and St Thomas'​ NHS Foundation Trust, London, UK; ^4^University College London Hospitals (UCLH), London, UK

*Journal of Cardiothoracic Surgery* 2023, **18(Supp 1)**:A255

**Objectives**

We seek to identify pre-operative prognostic factors and measure their effect on 5-year survival following Pulmonary Metastasectomy (PM) for Colorectal Cancer (CRC).

**Methods**

We systematically reviewed the databases of Cochrane Library, MEDLINE, Embase and Google Scholar from January 2000-April 2021 to identify pre-operative factors that have been investigated for their prognostic effect on survival following PM. Quality assessment was performed using the QUIPS tool. The prognostic effect of each identified factor on 5-year survival post PM was estimated using random-effects meta-analyses.

**Results**

We identified 115 eligible articles which included 13,294 patients who underwent PM from CRC. The overall 5-year survival after resection of the lung metastasis was 54.1%. The risk of bias of the included studies was at least moderate in 93% (107/115). Seventy-seven pre-operative factors had been investigated for their prognostic effect. Our analysis showed that 11 factors had favorable and statistically significant prognostic effect on 5-year survival post-PM. These included solitary metastasis, size < 2 cm, unilateral location, N0 thoracic disease, no history of extra-thoracic or liver metastasis, normal carcinoembryonic antigen levels both before PM and CRC excision, no neo-adjuvant chemotherapy before PM, CRC T-stage < T4 and no p53 mutations on CRC. Disease free interval at 24 months did not appear to affect 5-year survival.

**Conclusion**

We identified 11 factors that had a strong prognostic effect on 5-year survival, including single metastasis and unilateral disease. Despite the considerable risk of bias in the literature, this study comprises the most rigorous summary of the current evidence base. These findings can complement both clinical practice and the design of future research on the field of PM.
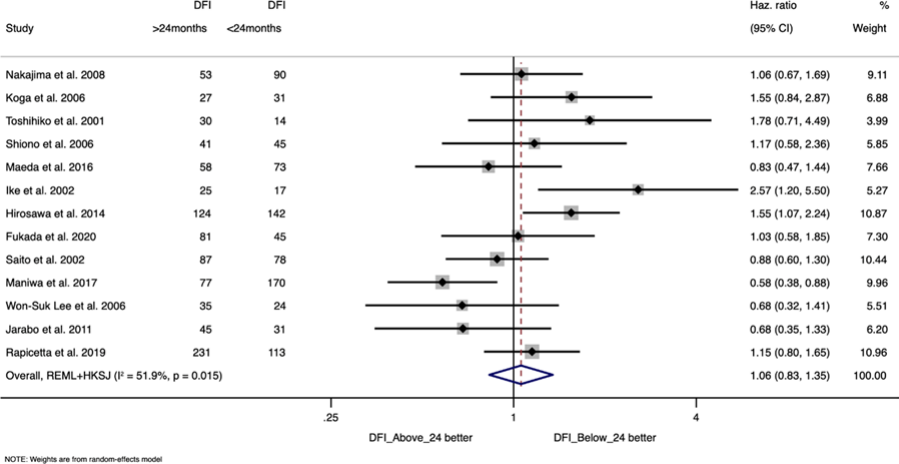


### A256 Performance of an Opt-out Integrated Pre-operative Tobacco Dependency Treatment Service

#### Brunswicker, Annemarie, Dr; Meghani, Nevan, Dr; Hewitt, Kath, Ms; Singhania, Asmita, Miss; Huddart, Helen, Ms; Ayrton, Laura, Ms; Evison, Matthew, Dr; Rammohan, Kandadai, Mr

##### Wythenshawe Hospital, Manchester, UK

*Journal of Cardiothoracic Surgery* 2023, **18(Supp 1)**:A256

**Objectives**

- Smoking tobacco is a significant risk factor for developing postoperative complications and poorer long-term outcomes following thoracic surgery.
- Current guidance is that the NHS should provide opt-out tobacco dependency treatment services to smokers at any point of contact, but provision of such services is woefully inadequate.
- Assess the performance of a new opt-out tobacco dependency treatment service (The CURE team) in a thoracic surgery service (service model = immediate ad-hoc review by a specialist tobacco dependency practitioner for all active smokers attending thoracic surgery outpatient clinics with direct supply of stop smoking pharmacotherapy).

**Methods**

- Retrospective data collection from electronic patient records and prospective patient questionnaires.
- Consecutive new patients attending thoracic surgery outpatient clinics from June to September 2021 were included.
- Key performance indicators were screening of smoking status, opt-review by the CURE team, uptake of specialist assessment, pharmacotherapy provision and quit rate.

**Results**

- 262 new patients attended the thoracic surgery service in the study period
- 40 (15.3%) were identified as active smokers
- 60% (24/40)completed specialist assessment with the CURE team in the opt-out model
- 79% (19/40) were prescribed pharmacotherapy Nicotine Replacement Therapy (NRT)
- 58% (14/24) quit smoking during the follow-up period
- 63% (25/40) of patients rated the opt-out service model as acceptable or very acceptable in a 5 point Likert scale question

**Conclusions**

- Our opt-out model ensured comprehensive screening, significant uptake of support and treatment for tobacco dependency with high quit rates.
- This service model provides a blueprint for the treatment of tobacco dependency in all outpatient services requiring investment in these services

### A257 COVID-19 Impact on Post-operative TNM Staging and Adjuvant Treatment for Primary Lung Cancer

#### Williams, Jennifer, Miss; Allen, R., Dr; Combellack, T, Mr; Kornaszewska, M., Miss; Pirtnieks, A., Mr; Valtzoglou, V., Mr

##### University Hospital of Wales, Cardiff, UK

*Journal of Cardiothoracic Surgery* 2023, **18(Supp 1)**:A257

**Objectives**

Our aim was to review the impact of COVID-19 on our primary lung cancer patients. Analysing if there was a delay in the referral pathway, acquiring PET imaging and subsequently resulting in significant post-operative TNM upstaging. We also assessed the impact COVID-19 had on those patients requiring adjuvant treatment, comparing this to the nationally quoted 66%.

**Methods**

374 patients who underwent primary lung cancer resections between January 2019 and September 2021 were identified from our PATS database. The retrospective data collected included pre-operative TNM staging via CT-PET imaging. This was compared with the post-operative surgical TNM stage. We also reviewed which patients had indications for adjuvant treatment and if this was undertaken during the first wave of COVID-19. We defined the first wave as March 2020 to September 2020, with 83 patients being identified.

**Results**

During the first wave of COVID-19 our single centre reports there was an increase in post-operative T and N upstaging; 7% and 4% respectively. The time in days between PET imaging and surgery in 2019 was 58 days and insignificantly increased to 63 days during the pandemic. The focus to maintain primary lung cancer resection in our single centre meant our waiting list for primary resections was not impacted by COVID-19 when compared to 2019 and 2021.

We observed that in patients who had T3 disease and borderline patients for adjuvant treatment during the first wave of COVID-19 were significantly less likely to receive adjuvant treatment. The quoted survival benefit for adjuvant treatment during the pandemic was 5%. During the first wave 19 patients underwent adjuvant treatment; 22% compared to the nationally quoted 66%.

**Conclusions**

Referral into the lung cancer pathway was delayed during COVID-19 resulting in T and N upstaging, rather than delay in MDT decision to offer surgical resection. Adjuvant treatment was significantly reduced during the first wave of COVID-19.

### A258 Outcomes and Characteristics of Patients with Second Primary Lung Cancer After Radical Treatment

#### Nizami, Maria, Miss; Farinelli, Eleonora, Dr; Ugur, Tugba, Dr; Ashrafian, Leanne, Miss; Pilling, John, Mr

##### Guy's Hospital, London, UK

*Journal of Cardiothoracic Surgery* 2023, **18(Supp 1)**:A258


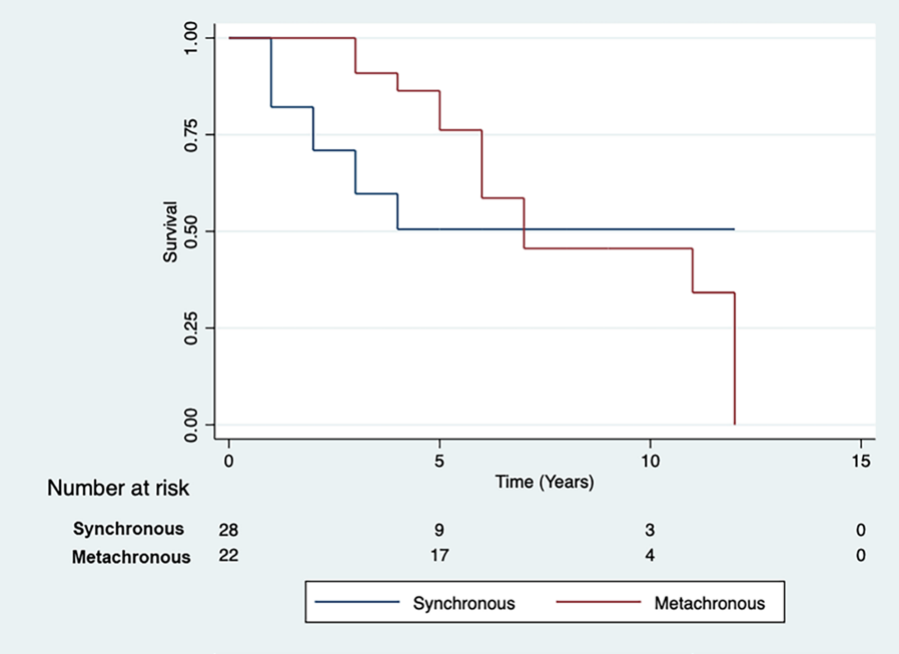


**Objectives**

Patients with non-small lung cancer (NSCLC) are at risk of developing a secondary primary lung cancer (SPLC). However, the characteristics of these group of patients at risk remain largely speculative. This study reviews our experience in the occurrence and the overall survival of SPLC.

**Methods**

We retrospectively reviewed 1366 patients undergoing radical treatment of multiple primary lung cancer from January 2010 to October 2021. Using criteria set out by Martini and Melamed [1], we categorised as synchronous SPLC when it was diagnosed within 24 months of the first primary lung cancer (FPLC) and after direct histological comparison of the different tumours. Tumours occurring after the 24 month interval were categorised as metachronous [1]. We compared the overall survival (OS) for each group.

**Results**

53 patients were identified with multiple or secondary primary lung cancer [median age 69(50–4);45.2%male] (M:22,F:31). In total 31 were synchronous, 22 were treated for metachronous tumours. The median interval between procedures for metachronous tumours is 39.5 months (25-111 months). 7 patients had further surgery for a third malignancy, 4 of which were synchronous and 3 metachronous (occurring at 45, 50 and 58 months after the second procedure). The primary lung cancer most commonly occurred in the right upper lobe, with the commonest site of second primary in the right lower lobe. The OS with synchronous SPLC was 82.1% at 1 year, 59.7% at 3 years and 50.6% at 5 years. For metachronous SPLC was 100% at 1 year, 90.9% at 3 years and 76.2% at 5 years. There was no statistical significant difference in OS (p = 0.47) between synchronous and metachronous disease (Fig. 1).

**Conclusions**

The occurrence of second primary lung cancer is not rare and a radical approach to these lesions is justified by the results. Aggressive surgical intervention is a safe and effective treatment for metachronous cancer and should highlighted within the MDT setting.

### A259 Impact of a Newly Established Robotic Program on the Practice of a Thoracic Surgery Department Amid the Covid Pandemic

#### Kouritas, Vasileios, Mr; Saad, Haisam, Mr; Alqudah, Obada, Dr; Szafron, Bartlomiej, Mr; Kadlec, Jakub, Mr; Bartosik, Waldemar, Mr; Hogan, John, Mr

##### Norfolk and Norwich University Hospital, Norwich, UK

*Journal of Cardiothoracic Surgery* 2023, **18(Supp 1)**:A259


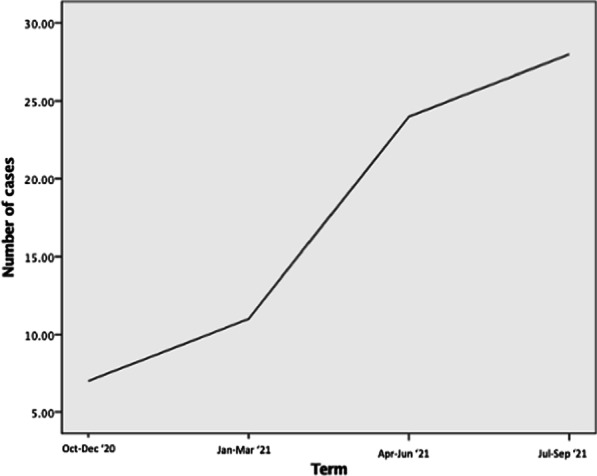


**Introduction**

Aim of this study was to evaluate the establishment of a new robotic program in Norfolk and Norwich University Hospital (NNUH) amid the Covid era.

**Patients and methods**

A Da-Vinci X system was used to establish the program.

A retrospective analysis of all robotic cases was performed using the business intelligence tool of the Trust (October 2020—October 2021) as service evaluation.

Data collected were age, gender, type of procedure, length of procedure, length of stay (LOS) and in-hospital/30-day mortality.

**Results**

During the study period 80 patients were operated on. Mainly anatomical lung resections were performed (29 lobectomies, 14 segmentectomies/bisegmentectomies) and resection of anterior mediastinal masses/thymectomies (16 cases).

The robotic cases performed increased in numbers throughout the study period (figure).

In the lung resection group, there were 2 conversions (4.6%) and 1 (2.3%) death for non-procedure related reason. The median LOS was 4 days (range 2–14) and the mean operative time was 168 ± 52.3 min. There were no R1 resections. More segmentectomies and bisegmentectomies were performed when compared to the rest of the activity.

**Conclusion**

Despite the Covid pandemic the establishment of a new robotic program was possible in the Trust showing low mortality and comparable results with the rest of the activity of the department.

### A260 Short Acting Opioid Analgesia, Breaking Through Pain in Thoracic Surgery

#### Petrov, George, Mr; O'Dwyer, Marliza, Mrs; Ryan, Ronan John, Mr; Fitzmaurice, Gerard, Mr

##### St James Hospital, Dublin, Ireland

*Journal of Cardiothoracic Surgery* 2023, **18(Supp 1)**:A260

**Objective**

Assessment of adherence to updated enhanced recovery after thoracic surgery (ERATS) analgesic protocol, incorporating elimination of Gabapentin and long-acting opioids, and evaluation of patient-reported post-operative pain.

**Methods**

Retrospective analysis of two groups of thoracic patients undergoing lobectomy or pleurectomy at distinct time points; August 2021 (Group A, n = 19) post-implementation and May 2021 (Group B, n = 23) pre-implementation, of the updated ERATS analgesic protocol in a single centre. This protocol focused on the elimination of regular prescribing of controlled analgesia apart from short acting opioids with expansion of NSAID use. We utilized the electronic patient record to evaluate adherence by analysing patient drug charts and discharge prescriptions; reported pain was assessed at 6-week clinic review.

**Results**

The majority of surgeries were VATS (A = 89%, B = 70%). Adherence to the updated protocol was 89.5%, with only 10.5% receiving long-acting opioids and/or Gabapentin, compared to 82% in Group B prior to implementation. At the 6-week post-operative review, 21% of Group A vs 13% of group B patients reported some ongoing pain.

**Conclusion**

The transition to the updated ERATS analgesic protocol in our unit has been successful, with a significant reduction in long-acting opioid and elimination of Gabapentin analgesic prescribing, minimizing patient exposure to their associated adverse effects and maintaining good pain control.

### A261 Surgery vs Oncological Treatment in High-risk Thoracic Patients: The Role of a High-risk Multidisciplinary Team Meeting (HRMDT)

#### Talukder, Shagorika^1^, Ms; Ramalingam, Aravindh^2^, Dr; Irvine, Michael^2^, Dr; Kadlec, Jakub^2^, Mr; Bartosik, Waldemar^2^, Mr; Szafron, Bartlomiej^2^, Mr; Van Tornout, Filip^2^, Mr; Kouritas, Vasileios^2^, Mr

##### ^1^Royal Papworth Hospital, Cambridge, UK; ^2^Norfolk and Norwich University Hospitals, Norwich, UK

*Journal of Cardiothoracic Surgery* 2023, **18(Supp 1)**:A261

**Introduction**

Multidisciplinary team (MDT) meetings for lung cancer are routinely utilized for decision making. There is little data on the implementation of high-risk MDTs (HRMDT) for patients referred to thoracic surgery. Oncological treatment is usually perceived as the safer alternative to a high-risk procedure. We aimed to compare outcomes between patients accepted for operative or non-operative management following HRMDT discussion.

**Methods**

Data for primary lung cancer patients discussed at the department’s fortnightly thoracic HRMDT between May 2019 and October 2021 were retrospectively analysed for baseline demographics, type and stage of cancer, total length of stay (LOS), and overall survival. Reasons for HRMDT discussion included poor lung function or cardiopulmonary exercise tolerance, technical operative challenges, multiple or severe comorbidities, advanced TNM staging, poor performance status and age > 80 years.

**Results**

115 primary lung cancer cases (mean 67 ± 10 years; 67 (58%) male) had HRMDT discussion, mostly due to poor lung function tests (33%). 78 (68%) patients received operative management. The gender, age and type of cancer were similar in the two groups. More patients with stage 2 and 3 disease were operated than sent for oncological treatment (59% vs 35%, *p* = 0.047). LOS for the surgical group was longer than the non-surgical group (median 7 vs 1 day *p* < 0.01). Surgery performed was not predictive of death by time (p = 0.629, CI 0.322—0.768). There were no in-hospital or 30-day deaths. Four deaths in total were documented in the surgical group and 3 in the non-surgical one (*p* = 0.432). Overall survival was similar between groups (Log-Rank = 0.236, *p* = 0.627).

**Conclusion**

Many patients, including those with progressed lung cancer, were ultimately offered surgery, which may otherwise not have been an option. High-risk operated patients showed similar longer-term survival as the non-operated group. Surgery may thus be safe in appropriately selected patients.

### A262 Robotic Surgery Reduces the Barrier to Widespread Practice of Segmentectomy

#### Sahdev, Nikhil^1^, Dr; Lee, Michelle^2^, Miss; Waller, David^2^, Mr; Stamenkovic, Sasha^2^, Mr; Wilson, Henrietta^2^, Miss; Baranowski, Ralitsa^2^, Miss; Lau, Kelvin^2^, Mr

##### ^1^Royal Brompton and Harefield Hospital, London, UK; ^2^St Bartholomew's Hospital, London, UK

*Journal of Cardiothoracic Surgery* 2023, **18(Supp 1)**:A262

**Objectives**

Segmentectomy offers an alternative to lobectomy but with parenchymal preservation and comparable outcomes. However, segmentectomy is technically more challenging. We report our experience of rapid adoption of segmentectomy through robotic surgery.

**Methods**

Single-institution retrospective audit of segmentectomies, from 2017–2021. Segmentectomies were performed by thoracotomy, video-assisted thoracoscopy (VATS) and robot-assisted thoracoscopy (RATS) by all 5 surgeons in one department. Data was analysed between 3 equal time periods (405 days), P1, P2, P3, representing different stages of implementation of the segmentectomy.

**Results**

217 segmentectomies were performed for proven or suspected stage I lung cancer. Number of segmentectomies increased by 348% (31(P1), 78(P2), 108(P3)); the ratio of lobectomy: segmentectomy increased from 100:13(P1) to 100:55(P3). 182(83%) segmentectomies were RATS, these increased 594% from 17(55%) (P1) to 101(95%) (P3). The median operative time fell: from 210 min(P1), 180 min(P2) to 173 min(P3).

The complexity of the segmentectomies also increased: the proportion of atypical segmentectomies increased sixfold (10%(P1) vs 65%(P3)).

Overall 30-day-mortality and 90-day-mortality was 1% and 3% respectively. Respiratory complications occurred in 30 cases (14%), persistent air leak in 59 (27%) and other complications in 15 (7%) cases. 11 cases (5%) returned to theatre and 13 (6%) required re-admission. There was no difference in complication rates between the study periods.

**Conclusions**

The number, proportion, and complexity of segmentectomies performed increased rapidly driven by the RATS approach. The complication and mortality rates remained similar in all study periods and the procedural efficacy improved, despite increasing complexity of the operations. Therefore, RATS provides a safe and effective method to enable delivery of segmentectomy as standard of care.

### A263 Animation Supported Consent for Lung Resection Procedures

#### Ike, David Ikenna^1^, Dr; Jackson-Wade, Rashaan^1^, Mr; Baranowski, Ralitsa^1^, Ms; Wilson, Henrietta^1^, Ms; Wald, David^1^, Prof; Perikleous, Periklis.^2^

##### ^1^St Bartholomew's Hospital, London, UK; ^2^Royal Brompton Hospital, London, UK

*Journal of Cardiothoracic Surgery* 2023, **18(Supp 1)**:A263

**Introduction**

Patient understanding of Lung Resection Procedures is often incomplete before consent to surgery. Innovative approaches are needed to improve the consent pathway and support shared decision-making.

**Methods**

- Video animations created by Explain my Procedure Ltd for evaluation pending subscription.
- Baseline understanding of patient understanding of Lung Resection Procedures (Questionnaire) n = 29
- Links to multi-language videos sent to patients pre-admission
- Patients provided with video books at pre-admission clinic and on admission to ward
- Post-animation patient Understanding of Lung Resection Procedures (Questionnaire) n = 30

**Results**

Figure 1 compares patient-reported understanding of the procedure, its benefits and risks and alternatives to the procedure in the no animation group and the animation group. There was a significant (P < 0.05) improvement in patient understanding in all domains following introduction of the animation to aid consent.

**Conclusion**

- Animation supported sample group showed a greater level of understanding in terms of procedure, benefits, risks and alternatives.
- Thoracic advanced nurse practitioners have found videos to be immensely useful especially with non-native English speakers.
- Patients who utilized the animations would recommend its use.
- Explain my Procedure recommended for routine use before consent to thoracic surgery.

### A264 Incidence and Resource Burden for the Management of CT Detected Ground Glass Opacities at a Tertiary Lung Cancer Service in the UK

#### Ashraf, Muhammad Arsalan^1^, Mr; Alshammari, Abdullah^1^, Dr; De Sousa, Paulo^1^, Mr; Tincknell, Laura^2^, Dr; Naruka, Vinci^1^, Mr; Booth, Sarah^1^, Mrs; Patel, Anant^3^, Dr; Proli, Chiara^1^, Miss; Docherty, Catherine^3^, Mrs; Lim, Eric^1^, Prof

##### ^1^Academic Division of Thoracic Surgery, The Royal Brompton Hospital, London, UK; ^2^Barking, Havering and Redbridge University Hospitals NHS Trust, Romford, UK; ^3^Royal Free London NHS Foundation Trust, London, UK

*Journal of Cardiothoracic Surgery* 2023, **18(Supp 1)**:A264

**Objectives**

The increased use of computer tomography (CT) for lung cancer screening and evaluation of other intrathoracic disease has led to greater awareness of ground glass opacity (GGO) lesions. We aim to evaluate the incidence of GGOs identified on CT at a tertiary lung cancer service in the UK to determine any trends and to quantify impact on time and resources.

**Methods**

We retrospectively identified patients reported with GGOs and discussed during MDT meetings held from 2017 to 2019 between the Royal Free and the Royal Brompton Hospitals. Data were collected, their demographics were reported, and annual incidence as well as further analyses on their management were calculated.

**Results**

3,731 patients were discussed at MDT meetings from 2017–2019. 53% were male, the mean age (SD) of the cohort was 68 years and 12% (438 patients) had GGOs identified on CT scans. GGO incidence showed an increasing trend between 2017 and 2019 at a frequency of 100 (9%), 159 (12%), 179 (14%) respectively. These 438 were filtered using an exclusion criterion to leave 274 individual patients. Of these, 148 (54%) were discharged from the MDT, 24 (9%) were deceased in the follow up period, and 31 (11%) were lost to follow-up; the rest remain under follow up. The median (IQR) follow-up time was 263 days (61–734) and time between scans was 89 days (32–183). 19 (10%) patients had biopsy proven pre-cancerous lesions or adenocarcinoma. 24 went on to have surgical intervention in our study period.

**Conclusion**

Over the three-year period of our study, we report an increasing trend in the identification and presentation of patients with GGOs in MDT. Combined with the extent of follow up and the risk of representing cancerous lesions, this demonstrates a significant burden in the present and future. We suggest an increased emphasis must be placed on establishing more effective pathways than those currently stipulated by existing national and international guidelines to better manage this burden.

### A265 Covid-19 Shouldn't Impact the Thoracic Surgical Training

#### Mayooran, Nithiananthan^1^, Mr; Chubsey, Rachel^2^, Ms; Kutywayo, Kudzayi^2^, Mr; Caruana, Edward^2^, Mr; Rathinam, Sridhar^2^, Mr

##### ^1^Nottingham City Hospital, Nottingham, UK; ^2^Glenfield Hospital, Leicester, UK

*Journal of Cardiothoracic Surgery* 2023, **18(Supp 1)**:A265

**Objectives**

Covid-19 pandemic has changed our lives in many ways. Surgical elective operation lists are reduced, which has had a direct impacted on surgical training. In our unit, after careful planning and implementation of strict ‘super clean pathway’ has enabled us to continue lung cancer operations till end of December 2020. This study is to assess the impact of Covid-19 pandemic in training Opportunities.

**Methods**

A single thoracic surgical firm’s pre Covid-19 (January 2019 till December 2019) case Loads of were analysed with cases performed during Covid 19 pandemic (Jan 2020 till December 2020). The following variables are collected for each year; type of operation, elective vs emergency, primary surgeon, operating surgeon, and percentage of the operation performed by Trainee as a first operator. Correlation of data was performed to analyse the influence on training opportunities.

**Results**

Pre- Covid 19 pandemic single surgeon performed a total of 328 major thoracic cases, Out of these 99% of cases were performed by trainee as a first operator. During the pandemic a total of 238 cases were performed. Which is almost 27.5% lesser activity than 2019. In the beginning of 2020 (First Quarter) The monthly case numbers remained the same. After April 2020, there is an expected gradual decrease in general case load noted. Regardless of this, the trainees performed almost 90% of these cases as first operator.

**Conclusions**

The Covid -19 Pandemic has had a considerable impact on Thoracic surgical activity after March 2020 till date. But this hasn’t impact on training opportunities in our unit. The trainees have had their fair share of operative training, Which maintained a high morale among the trainees during this difficult time.

### A266 Comparison of Efficacy of Intra-operative Regional Analgesia Techniques for Video Assisted Thoracoscopic Surgery (VATS)

#### Shenoy, Ranjeetha^1^, Dr; Kew, Ee Phui^1^, Mr; Basharat, Kamran^2^, Dr; Tan, Carol^1^, Ms

##### ^1^St George's University Hospitals, London, UK; ^2^Kingston Hospital, Kingston upon Thames, UK

*Journal of Cardiothoracic Surgery* 2023, **18(Supp 1)**:A266

**Objectives**

Poor pain control after thoracic surgery is associated with increased risk of respiratory complications and chronic pain. Paravertebral catheter (PVC) placed under direct vision has been found an effective analgesia, but poses a small risk of infection, hematoma, and pleural leak. Alternative to the PVC are single injection paravertebral block (PVB) and multi-level intercostal nerve block (INB). Our objective is to compare the efficacy of the PVC, PVB and INB.

**Methods**

Retrospective study of elective patients undergoing VATS procedure between February and April 2021. All patients received intra-operative PVB, INB or PVC, as well as morphine patient-controlled analgesia (PCA) and regular oral analgesia. The outcomes measured were PCA usage and duration, duration of chest drain, and length of hospital stay (LOS).

**Results**

61 cases were included (see table). The mean PCA usage in 24 h was the lowest in PVB compared to INB and PVC (21.53 mg vs. 28.16 mg vs. 27.63 mg respectively). The mean PCA duration was also the lowest in PVB (1.1 days) compared to INB (1.37 days) and PVC (1.4 days). The mean duration of chest drain and LOS were also shorter in PVB with 2.3 and 3.5 days respectively (vs. INB 2.31 and 3.67 days; PVC 2.87 and 3.87 days). Subgroup analysis of only lobectomy and wedge resection revealed that PVB had the lowest PCA usage in 24 h (18.53 mg vs. INB 28.13 mg vs. PVC 26.6 mg), shortest PCA duration (1 1.1 vs. INB 1.5 vs. PVC 1.36 days), shortest hospital stay (3.5 vs. INB 4.29 vs. PVC 3.91 days), and shortest chest drain duration (2.3 vs. INB 2.36 vs. PVC 3.91 days).

**Table:** 

Surgical procedures (VATS)	Overall (N = 61)	Intercostal nerve block (N = 36)	Single injection paravertebral block (N = 10)	Paravertebral catheter (N = 15)
Lobectomy	25 (40.9%)	11 (30.5%)	9 (90%)	5 (33.3%)
Wedge resection	10 (16.3%)	3 (8.3%)	1 (10%)	6 (40%)
Pleural biopsy and pleurodesis	17 (27.8%)	17 (47%)	-	-
Bullectomy and pleurodesis	6 (9.8%)	2 (5.5%)	-	4 (26.6%)
Mediastinal mass excision	2 (3.2%)	2 (5.5%)	-	-
Pericardial window	1 (1.6%)	1 (2.7%)	-	-

**Conclusions**

PVB is associated with the least PCA usage and the shortest LOS. All of the outcomes were statistically insignificant but data collection is on-going.

### A267 Are we Really Justified in Redo Surgery for a Positive Resection Margin?

#### Lee, Michelle, Miss; Alvarado, Patricia, Dr; Nardini, Marco, Mr; Waller, David, Mr

##### Barts Thorax Centre, London, UK, St Bartholomew's Hospital, London, UK

*Journal of Cardiothoracic Surgery* 2023, **18(Supp 1)**:A267

**Objectives**

To challenge the value of elective redo surgery for patients following an R1 resection for lung cancer.

**Methods**

In a retrospective 5 year analysis of a prospectively collected institutional database the perioperative course of 17 patients undergoing elective redo surgery for R1 disease was analysed. The outcome of the primary operations (Group A) was compared (by age, comorbidities and type of procedures) with the outcome of the second procedures (Group B) in terms of in hospital complications (IHC) rate and length of hospital stay (LOS).

**Results**

The primary operation was lobectomy in 0/17 (0%), segmentectomy in 4/17 (24%) and wedge resection in 15/17 (88%). The overall complication rate of Group A was 6% versus 58% of Group B. Only 1 patient (6%) had cancer detected in the second specimen. First admissions ranged between 4(2–10) days, whereas second admissions ranged from 7(3–27) days.

**Table:** 

Redo Operation	n	R1 Indication			Residual Tumour	Complications	P Value
		Parenchyma	Bronchus	Nodal		12/17 (71%)	
Segmentectomy	1/17 (6%)	1/17 (6%)	0%	0%	0%	1/12 (8%) Prolonged Air Leak (PAL)	NS
Lobectomy	16/17 (94%)	12/17 (71%)	2/17 (12%)	2/17 (12%)	1/17 (6%)	PAL 5/12 (42%), Haemorrhage 3/12 (25%), Hospital-acquired Pneumonia (HAP) 2/12 (17%), Sepsis 1/12 (8%)	NS
Pneumonectomy	0/17 (0%)	0%	0%	0%	0%	0%	NS

**Conclusion**

These results question the value of elective redo completion surgery for R1 disease in view of higher IHC, LOS and absence of cancer in the ‘redo’ specimen. Further randomised comparison with observation alone is suggested.

### A268 A 15-year Experience of Colorectal Pulmonary Metastasectomy in a High-volume Tertiary Referral Centre

#### Taylor, Marcus^1^, Mr; Singhania, Asmita^1^, Ms; Biswas, Sayan^1^, Mr; Grant, Stuart^2^, Mr; Krysiak, Piotr^1^, Mr; Fontaine, Eustace^1^, Mr; Granato, Felice^1^, Mr; Joshi, Vijay^1^, Mr; Rammohan, Kandadai^1^, Mr

##### ^1^Wythenshawe Hospital, Manchester, UK; ^2^Manchester University, Manchester, UK

*Journal of Cardiothoracic Surgery* 2023, **18(Supp 1)**:A268

**Objectives**

Results of the PulMiCC trial have challenged the role of pulmonary metastasectomy as part of the management of metastatic colorectal cancer in contemporary practice. We aimed to review our short and long-term outcomes for patients undergoing surgical resection of pulmonary colorectal metastases.

**Methods**

A retrospective analysis of electronic patient record data was performed. All patients undergoing lung resection for pathologically confirmed colorectal pulmonary metastatic disease from November 2005 to May 2021 were included. In-hospital, 90-day, 1-year, 2-year, 5-year and 10-year mortality rates were analysed. Cox proportional hazards regression analysis was used to identify factors associated with reduced overall survival. Statistical analysis was undertaken using SPSS version 28.

**Results**

In total, 619 patients underwent surgery during the study period. Mean age was 66.0 years (± 10.0) and 61.2% (n = 379) were male. Overall, 68.7% (n = 425) underwent open surgery. There were 151 (24.4%) patients who had multiple metastases resected. Median follow-up time was 45 months (IQR 23–87). In-hospital mortality was 0.3% (n = 2) and the median post-operative length of stay was 4 days (IQR 3–5). 90-day and 1-year mortality rates were 1.1% (n = 7/619) and 4.5% (n = 26/580), respectively. The 5-year and 10-year mortality rates were 40.6% (n = 165/406) and 74.0% (n = 208/281), respectively. After multivariable analysis, advanced age (HR 1.026, 95% CI 1.011–1.043, p < 0.001) and resection of more than one metastasis (HR 1.524, 95% CI 1.108–2.097, p = 0.010) were independently associated with reduced overall survival.

**Conclusion**

Although there is inherent selection bias associated with patients referred for surgical management of colorectal cancer, our results demonstrate extremely low short-term mortality and encouraging longer-term outcomes for these patients. Advanced age and the presence of more than one metastasis at the time of surgery were associated with worse prognosis.

### A269 Outcomes After Lung Resection for Primary Lung Cancer in Octogenarians: Trends Over Time

#### Taylor, Marcus^1^, Mr; King, Jenny^1^, Dr; Sinnott, Nicola^1^, Dr; Crosbie, Phil^2^, Dr; Booton, Richard^1^, Prof; Shackcloth, Michael^3^, Mr; Granato, Felice^1^, Mr; Grant, Stuart^2^, Mr; Fontaine, Eustace^1^, Mr; Rammohan, Kandadai^1^, Mr

##### ^1^Wythenshawe Hospital, Manchester, UK; ^2^Manchester University, Manchester, UK; ^3^Liverpool Heart and Chest Hospital, Liverpool, UK

*Journal of Cardiothoracic Surgery* 2023, **18(Supp 1)**:A269

**Objectives**

Despite octogenarians representing an ever-increasing proportion of patients with lung cancer, there is a paucity of evidence describing outcomes after lung resection for these patients. We aimed to evaluate outcomes for octogenarians after lung resection over time.

**Methods**

A total of 5470 consecutive patients undergoing lung resection for primary lung cancer in two UK centres were included. The cohort was divided into two groups (group 1: 2012–2015 and group 2: 2016–2019) to identify trends over time. Primary outcomes were peri-operative, 90-day & 1-year mortality and post-operative complications. Univariable analyses were used to compare outcomes between octogenarian and non-octogenarian patients.

**Results**

Overall, 9.4% (n = 513) of patients were aged 80 years or over. There was no significant difference in the peri-operative and 90-day mortality rates for octogenarians between groups 1 and 2, however the 1-year mortality rate for octogenarians was significantly lower for group 2 compared to group 1 (2012–2015: 16.5% vs 2016–2019: 10.2%, p = 0.034). There was also no significant difference in peri-operative, 90-day or 1-year mortality between octogenarian and non-octogenarian patients in group 2, but not in group 1.

**Conclusions**

Mortality for octogenarians fell significantly over time in this study. Indeed, when confined to the most recent time period, comparable rates of both 90-day and 1-year mortality for octogenarian and non-octogenarian patients were seen. Whilst preventative strategies to reduce the incidence of post-operative atrial fibrillation in octogenarians should be considered, these findings demonstrate that following appropriate patient selection, octogenarians can safely undergo lung resection for lung cancer.

### A270 Outcomes After Lung Resection for Primary Lung Cancer in Never Smokers

#### Taylor, Marcus^1^, Mr; Abah, Udo^2^, Ms; Smith, Matthew^2^, Mr; Grant, Stuart^3^, Mr; Shackcloth, Michael^2^, Mr; Granato, Felice^1^, Mr; Crosbie, Philip^3^, Dr

##### ^1^Wythenshawe Hospital, Manchester, UK; ^2^Liverpool Heart and Chest Hospital, Liverpool, UK; ^3^Manchester University, Manchester, UK

*Journal of Cardiothoracic Surgery* 2023, **18(Supp 1)**:A270

**Objectives**

Chronic exposure to tobacco smoke is the main environmental risk factor for developing lung cancer. However, an important proportion of patients diagnosed with lung cancer have never smoked. We aimed to assess whether short and long-term outcomes after lung resection were different between never smokers and ever smokers.

**Methods**

All consecutive patients undergoing lung resection for primary lung cancer between 2012 and 2018 in two UK centres were included. Patients with missing smoking status data were excluded. Any patient with a history of smoking was defined as a smoker, regardless of pack years. Primary outcomes were 90-day mortality, 1-year mortality and overall survival. Statistical tests were used to compare short-term outcomes (chi-square test) and overall survival (multivariable Cox regression analysis) between never and ever smokers.

**Results**

Of the 4955 patients included in the study, 83.0% (n = 4115) were smokers and 17.0% (n = 840) were never smokers. Smokers had significantly worse lung function, functional status and comorbidity burden. Smokers were less likely to undergo VATS surgery but were not more likely to have more advanced-stage disease. The rates of pulmonary complications were significantly higher for smokers (lower respiratory tract infection: 11.7% vs 8.5%, p = 0.007; reintubation: 3.3% vs 1.4%, p = 0.004). The 90-day mortality rate was not significantly higher for smokers (3.8% vs 3.0%, p = 0.227) but smokers had significantly higher 1-year mortality (11.6% vs 7.7%, p = 0.001). Ever smoking was associated with significantly reduced overall survival despite adjustment for stage of disease (HR 1.302, 95% CI 1.112–1.525, p = 0.001).

**Conclusions**

Never smokers represented just under one-fifth of patients undergoing resection for primary lung cancer. As expected never smokers had better lung function and less co-morbidities. This study has demonstrated that never smokers have significantly better short and mid-term.

### A271 Impact of COVID-19 in Lung Cancer Service and Disease Progression – A Single Centre Experience

#### Chan, Jeremy, Dr; Lallmahomed, Najeeba, Miss; Lhote, Francois, Mr

##### Morriston Hospital, Swansea, UK

*Journal of Cardiothoracic Surgery* 2023, **18(Supp 1)**:A271

**Introduction**

The COVID-19 pandemic has a significant impact on lung cancer resection. Limited service leads to delay in surgical treatment. Previous studies showed delay between diagnosis and surgical resection may result in disease progression and dismal outcome. While several articles summarise their experience on the impact of COVID-19 in lung cancer resection, disease progression secondary to delay in surgery remains unknown. We aim to share our experience.

**Method**

All patients underwent primary lung cancer resection from June 2020 to May 2021 were included in this study. The date between diagnosis and surgical referral, MDT and surgery date were evaluated. The tumour size and staging was compared between pre-operative imaging and histological sample.

**Results**

A total of 61 patients were included in this study. The average date between diagnosis/thoracic surgery clinic to surgery was 120 and 50 days, respectively. Upstaging in T and N staging were seen in 27.87% (17/61) and 11.48% (7/61) of lung resection cases, respectively. No differences were observed when compared between the pre and post-operative mean tumour size (25.71 cm2 vs 28.66cm2, p = 0.37).

**Conclusion**

The COVID-19 pandemic leads to a significant delay in lung cancer resection and disease progression was observed. Better resources allocation is required to improve the service after the pandemic.

### A272 The Impact of Intra-operative Conversion During Planned Video-assisted Thoracoscopic Lobectomy for Primary Lung Cancer on Short and Long-term Outcome

#### Taylor, Marcus^1^, Mr; Raj Krishna, Gokul^1^, Mr; Grant, Stuart^2^, Mr; Rammohan, Kandadai^1^, Mr; Fontaine, Eustace^1^, Mr; Joshi, Vijay^1^, Mr; Granato, Felice^1^, Mr

##### ^1^Wythenshawe Hospital, Manchester, UK; ^2^Manchester University, Manchester, UK

*Journal of Cardiothoracic Surgery* 2023, **18(Supp 1)**:A272

**Objectives**

Video-assisted thoracoscopic surgery (VATS) is recommended as the gold standard for early-stage lung cancer surgery in the UK. There is variation in the causes and rates of intra-operative conversion to open surgery. Our objective was to review the impact of intra-operative conversion from a VATS approach on outcomes after resection for primary lung cancer.

**Methods**

A total of 2622 consecutive patients undergoing anatomical pulmonary lobectomy for primary non-small cell lung cancer between 2012 and 2019 in a single UK centre were included. Primary outcomes were 90-day mortality and overall survival. Conversions were classified as due to bleeding or non-bleeding reasons. Outcomes were compared between groups using univariable analysis.

**Results**

Overall, 20.6% (n = 541) completed surgery via VATS and 79.4% (n = 2081) via thoracotomy. A total of 631 patients were planned to undergo VATS surgery giving an overall conversion rate of 14.3% (n = 90). Bleeding was the reason for conversion in 31.1% (n = 28/90) of patients. The 90-day mortality rate after conversion was not significantly different to the 90-day mortality rate for either planned open surgery (3.3% vs 3.4%, p = 0.987) or surgery completed via VATS (3.3% vs 1.1%, p = 0.099). Whilst experiencing conversion was associated with significantly reduced overall survival in comparison to completing surgery via VATS (p < 0.001), there was no significant difference in overall survival between patients experiencing intra-operative conversion and those undergoing planned open surgery (p = 0.135).

**Conclusion**

In our experience, patients experiencing intra-operative conversion have similar short and long-term outcomes to patients undergoing planned open surgery. Short and long-term mortality was lower for patients who underwent pulmonary lobectomy for primary non-small cell lung cancer via VATS compared to patients who had a thoracotomy.

### A273 Single Centre Outcomes for Lobectomy vs Sub-lobar Resection in Primary Lung Cancer

#### Brazier, Andy^1^, Mr; Mahendran, Kajan^1^, Mr; Ahmed-Issap, Amber^2^, Miss; Jain, Shubham^1^, Mr; Habib, Akolade^1^, Dr; Briant, Zachariah^2^, Mr; Menon, Sowmya^2^, Miss; Srinivasan, Lakshmi^1^, Miss; Ghosh, Shilajit^1^, Mr; Abah, Udo^1^, Miss

##### ^1^UHNM, Manchester, UK; ^2^Keele University, Newcastle, UK

*Journal of Cardiothoracic Surgery* 2023, **18(Supp 1)**:A273

**Objectives**

Lobectomy is the gold standard treatment for early lung cancer. Sublobar resection is a useful alternative in patients with limited lung function but has recently garnered evidence for its role as definitive treatment; with fewer complications and comparable survival. We designed a study to observe if our results matched current evidence.

**Methods**

We analysed all resection from 01/01/2012 to the 07/07/2021. Variables where extracted from a prospectively filled database and missing data supplemented from records. Patients were divided into three cohorts; lobectomies, segmentectomies and wedge resections. Complications, length of stay and mortality figures were examined.

**Results**

1400 patients had histology confirming primary lung cancer. 861 (61.5%) received a lobectomy, 255 (18.2%) segmentectomy and 284 (20.3%) wedge resections. Five-year survival data was available for a 353, 73 and 139 patients respectively. There were fewer complications and shorter hospital stays following sublobar resections. Five-year survival was higher following segmentectomy compared to lobectomy. This was not true for wedge resections (Table1).

**Conclusions**

Despite poorer pre-operative condition, patients who underwent sublobar resections faired better in the short term than lobectomy patients. Where sublobar resection is indicated, formal segmental resection should be performed to convey a five-year survival benefit.

**Table:** 

VARIABLE	LOBECTOMY	SEGMENTECTOMY	WEDGE
N	861	255	284
Age (mean)	69	71	71
Arrhythmia (%)	7.20	2.35	3.87
LRTI (%)	13.82	7.84	13.38
Prolonged air-leak (%)	14.75	13.73	13.03
Length of stay in days (mean/median)	8.63/5.3	7.33/5.94	6.82/5.07
HDU length of stay in days (mean/median)	2.73/1.2	1.49/1.03	1.41/0.97
30-day mortality (%)	2.67	1.96	1.41
5 year survival (%)	66.01	73.97	56.64

### A274 Nutritional Status of Lung Cancer Patients Undergoing Lung Surgery

#### Simmonds, Shanique, Dr

##### Queen Elizabeth Hospital Birmingham, Birmingham, UK

*Journal of Cardiothoracic Surgery* 2023, **18(Supp 1)**:A274

**Objectives**

The nutritional status of patients undergoing thoracic surgery is not well known and has been generally neglected on a national scale. Therefore, the aim of this audit was to assess the nutritional status in lung cancer patients pre-and -post lung cancer surgery and identify strategies for nutritional optimisation in this patient group.

**Methods**

On the day of surgery, participants completed two questionnaires – the PG-SGA which is a patient-reported nutritional assessment tool that has been validated in cancer patients and the SARC-F which is used as a predictor of sarcopaenia. Four weeks postoperatively, participants were contacted via telephone consult to once again complete both questionnaires.

**Results**

Preoperatively seven participants reported scores indicative of requiring nutritional intervention on the PG-SGA assessment. This reduced to three participants postoperatively. Preoperatively, four participants achieved scores predictive of sarcopaenia, whereas postoperatively this reduced to one participant.

**Conclusions**

There are a proportion of patients undergoing thoracic surgery for lung cancer that may benefit from pre-operative nutritional support and/or physiotherapy input to optimise them for surgery.

### A275 Lobectomy After Prior Contralateral Lobectomy: High Risk, High Reward

#### Barrett, Sean, Mr; Kennedy, Fionnuala, Dr; McLoughlin, Joseph, Mr; Keane, Colm, Dr; Ryan, Ronan, Mr; Young, Vincent, Mr; Fanning, Niall, Dr; Fitzmaurice, Gerard, Mr

##### St. James's Hospital, Dublin, Ireland

*Journal of Cardiothoracic Surgery* 2023, **18(Supp 1)**:A275

**Objectives**

Lobectomy after previous contralateral lobectomy is a radical treatment option for patients presenting with bilateral lung malignancies, intuitively representing increased perioperative risk. Due to the rarity of cases, there is limited outcome data for these patients. The aim of this study was to examine outcomes for this select patient group and we hypothesised that mortality rates would be lower compared with patients undergoing a pneumonectomy.

**Methods**

A retrospective review was performed on a prospectively collected clinical database of all patients who underwent staged contralateral lobectomy for pulmonary malignancies between January 2012 and June 2021. Sublobar resections and completion pneumonectomies were excluded.

**Results**

31 patients met the inclusion criteria with a mean age of 65.6 years. Pulmonary function tests pre-second lobectomy demonstrated a mean FEV1 of 75.4% and DLCO of 69.3%. 74% (n = 23) of cases were performed via open thoracotomy. Contralateral double lumen tubes were used for all patients with a permissive hypercapnic strategy employed in one-third of cases. 78% of second lobectomies had Stage I/II disease. The ICU admission rate was 29% (n = 9) and reintubation rate was 22.6% (n = 7). The 30- and 90-day mortality was 6.4% (n = 2). The median hospital length of stay was 9 days.

**Conclusions**

The mortality rates for staged lobectomy were lower than those expected with pneumonectomy. Although this group have a significant ICU admission rate of 29%, discharge to home and rates of survival were excellent. Consequently, we suggest that staged lobectomy is a suitable treatment strategy in carefully selected patients presenting with contralateral malignancies and pursuit of extended ICU admissions is worthwhile.

Figure 1: Outcomes following Contralateral Staged Lobectomy.
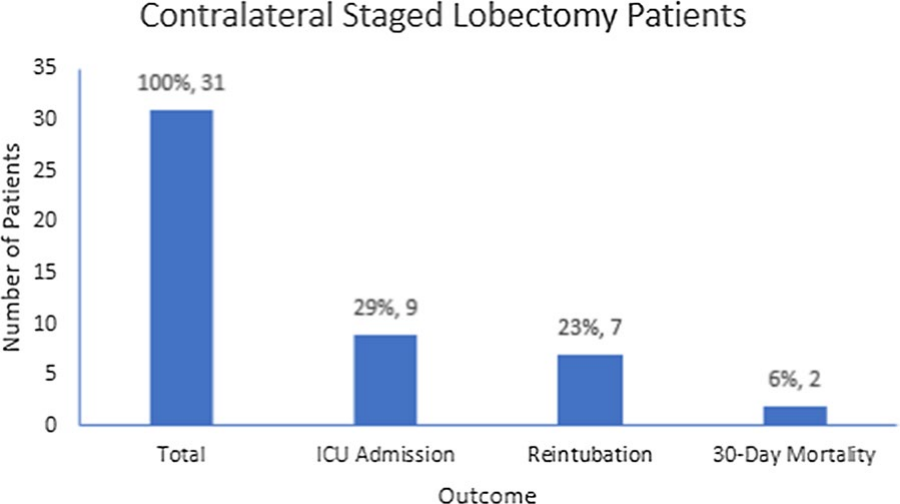


### A276 Factors linked to Outcome in Resected Pulmonary Typical Carcinoid Tumours

#### Patel, Akshay^1^, Mr; Perris, Rebecca^1^, Dr; Mangel, Tobin^2^, Dr; Humphries, Sian^1^, Ms; Smith, Stacey^1^, Mrs; Shah, Tahir^1^, Dr; Hughes, Simon^1^, Dr; Rogers, Vanessa^1^, Miss; Naidu, Babu^1^, Mr; Kalkat, Maninder^1^, Mr

##### ^1^Queen Elizabeth Hospital, Birmingham, UK; ^2^St. George's Hospital, London, UK

*Journal of Cardiothoracic Surgery* 2023, **18(Supp 1)**:A276


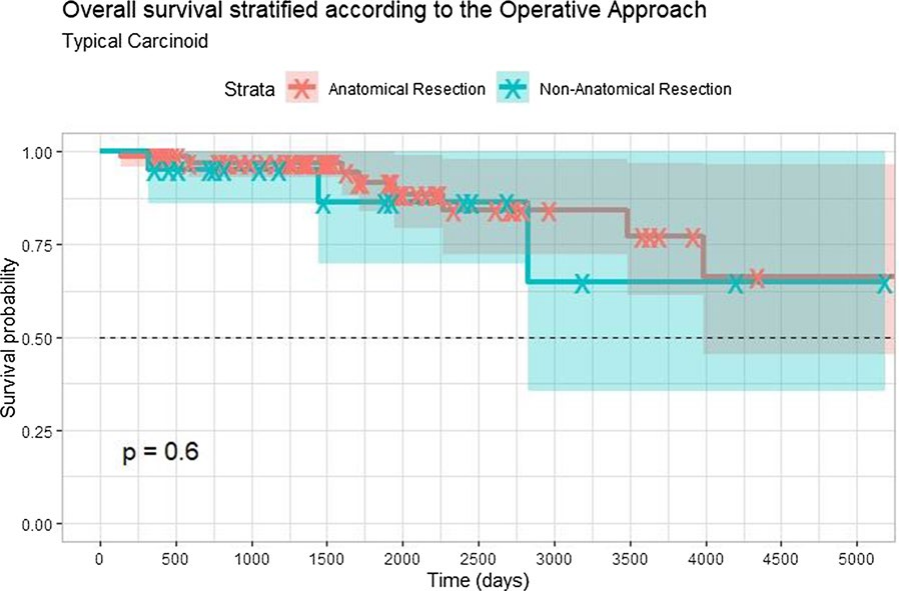


**Objectives**

Typical carcinoids account for the majority of lung carcinoids and have a better prognosis and lower propensity for metastatic spread than atypical carcinoids. Surgical resection with lymphadenectomy is the gold standard for patients with bronchial carcinoids. Typical carcinoids are amenable to treatment with non-anatomical sublobar resection with numerous groups having reported no significant difference in outcomes in patients with wedge resection compared to those patients that underwent anatomical resection. We explored the impact of pre-operative, operative and pathological factors on overall and disease-free survival.

**Methods**

We performed a retrospective interrogation of data collected from 92 patients with typical pulmonary carcinoid tumours who underwent surgical resection over a 25-year period at our institute. All demographic, operative, post-operative pathological and survival data was collected. Median follow-up was 1359 days (726–2265 days). Kaplan–Meier and Multivariate Cox Proportional Hazards modelling were performed in R studio, Rv4.0.3.

**Results**

Forty-one of these tumours were endobronchial and 51 were in the lung parenchyma. The majority were resected anatomically (n = 71), and through a VATS approach (n = 61). Mortality and recurrence rates at 10 years were 16% (n = 15) and 14% (n = 13) respectively. There was no significant difference between anatomically and non-anatomically resected typical carcinoids (log-rank, p = 0.6; Fig. 1). Significant independent predictors of overall survival were advanced age (HR 1.09, p = 0.004) and VATS approach (HR 0.31, p = 0.048). Type 2 Diabetes was a negative prognostic factor for disease recurrence in this cohort (HR18.9, p = 0.013).

**Conclusions**

Sublobar resection may be an appropriate surgical strategy in typical carcinoid tumours, however, further work is needed to investigate the role of segmentectomy versus lobectomy and indeed segmentectomy versus wedge resection.

### A277 Surgery vs. Radiotherapy for The Treatment of Early-Stage Non-Small Cell Lung Cancer: A Systematic Review/Meta-Analysis of Propensity Matched Studies

#### Barrett, Sean, Mr; Rice, Darragh, Dr; Higgins, Patrick, Mr; McLoughlin, Joseph, Mr; Fleming, Christina, Ms; Eaton, Donna, Ms

##### Mater Misericordiae University Hospital, Dublin, Ireland

*Journal of Cardiothoracic Surgery* 2023, **18(Supp 1)**:A277

**Objectives**

Lung cancer is the leading cause of cancer death worldwide. With the increasing popularity of lung cancer screening, the incidence of early-stage lung cancers is on the rise. The current gold standard for management of early disease is surgery. Advances in radiotherapy techniques and recent literature demonstrating similar outcomes in certain patient groups has led to an increase in the popularity of this treatment modality. We performed a meta-analysis of propensity score matched studies to compare these treatments.

**Methods**

The overall aim was to synthesis the best available evidence comparing surgery versus radiotherapy for the treatment of early-stage non-small cell lung cancer with regard to 5- and 3- year survival and recurrence rates. A comprehensive search of Pubmed, Embase, Scopus, and Web of Science was performed up to April 2021. We included retrospective propensity score matched studies for quantitative analysis. The study was performed in line with PRISMA guidelines. Statistical analysis was performed using Revman software.

**Results**

22 studies were included in the meta-analysis. There were statistically significant superior outcomes in the surgical group for 5-year overall survival [RR 1.47 (95% CI 1.28–1.68) p < 0.001] and 3-year overall survival[RR 1.24 (95% CI 1.10–1.40) p < 0.001]. These findings persisted on subgroup analysis of lobectomy and sublobar resection versus radiotherapy.

**Conclusions**

According to the best current evidence in the form of retrospective propensity matched studies, surgical approaches remain the gold standard of treatment of early-stage non-small cell lung cancer. Further evidence in the form of prospective randomised controlled trials is needed to provide optimum evidence in the subject.

Figure 1: Table of Outcomes.
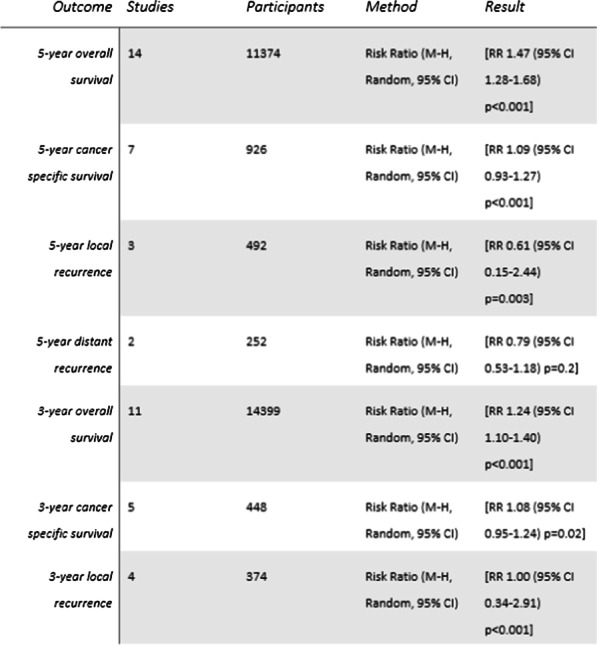


### A278 The Current Under the Wave: Increase in Upstaging in Early-stage NSCLC in the COVID Era

#### Lodhia, Joshil, Mr; Hussein, Nabil, Mr; Brunelli, Alex, Mr; Chaudhuri, Nilanjan, Mr; Milton, Richard, Mr; Papagiannopoulos, Kostas, Mr; Teh, Elaine, Miss; Tcherveniakov, Peter, Mr

##### St James University Hospital, Leeds, UK

*Journal of Cardiothoracic Surgery* 2023, **18(Supp 1)**:A278

**Objectives**

The COVID pandemic has led to reduced access to outpatient surgical appointments and operative capacity. The purpose of this study was to evaluate the delay from diagnosis to treatment of early-stage non-small cell lung cancers (NSCLC) [Stage IA–IB] and if this led to an increase in upstaging and in turn a need for adjuvant therapy.

**Methods**

Data of consecutive NSCLC lung resections between January 2019 to August 2021 were retrospectively collected. The COVID period was identified as post January 2020. Patients who required further surgical staging or those with secondary lung cancers were excluded. Chi-square test was used for statistical analysis.

**Results**

The number of resections, following exclusion, were 195 [2019], 194 [2020] 144 [2021]. There was an increase in the referrals of early-stage lung cancers 67% [2019], 72% [2020] and 81% [2021, p = 0.015]. The proportion of lobectomies performed were 75% [2019], 66% [2020], 62% [2021, p = 0.068]. The proportion of sub-lobar resections increased from 19% [2019] to 32% [2020] and 36% [2021, p = 0.004]. The proportion of VATS procedures increased from 80% [2019] to 88% [2020] and 92% [2021, p = 0.023]. There was no delay in PET to surgical review [31 ± 17 vs 33 ± 19, p = 0.94], however, a delay was observed between surgical review and surgery [21 ± 19 vs 29 ± 22, p < 0.0001]. There was an increase in upstaging between 2019 and the COVID period of both tumour [35% vs 48%, respectively] and nodal [19% vs 28%, respectively] staging, p = 0.02 but no difference in R1 margins [5%, p = 0.99]. Overall, the percentage of patients who were upstaged to require adjuvant chemotherapy increased from 18% [2019] to 25% [2021, p = 0.01].

**Conclusions**

Despite the increased proportion of patients being referred with early-stage lung cancers, this cohort has seen an increase in upstaging above the levels from the pre-COVID era. This is, likely, a consequence of the delay from surgical outpatient assessment to surgery, brought on by the COVID pandemic.

### A279 Electromagnetic Navigational Bronchoscopy Marking Image Guided Robotic (iRATS) S9 + 10 Segmentectomy—Movie

#### Lau, Kelvin, Mr; Perikleous, Periklis, Mr; Nardini, Marco, Mr; Stamenkovic, Steven, Mr

##### St Bartholomew's Hospital, London, UK

*Journal of Cardiothoracic Surgery* 2023, **18(Supp 1)**:A279

https://www.youtube.com/watch?v=ueh-bMqNUGA

### A280 Pulmonary Metastasectomy Outcomes for Sarcoma: Should we Always Operate?

#### Hoppe, Solveig^1^, Dr; Taberham, Rhona^2^, Miss; Stavroulias, Dionisios^2^, Mr

##### ^1^St Mary's Hospital, Isle of Wight NHS Trust, Newport, UK; ^2^John Radcliffe Hospital, Oxford University Hospitals NHS Foundation Trust, Oxford, UK

*Journal of Cardiothoracic Surgery* 2023, **18(Supp 1)**:A280

**Objectives**

Pulmonary metastases are a frequent site of disease recurrence in patients with sarcoma. Metastasectomy is a mainstay of treatment for these patients who otherwise have controlled disease. We aimed to review the survival outcomes from sarcoma patients undergoing pulmonary metastasectomy in our institution.

**Methods**

We retrospectively reviewed the outcomes of a single surgeon’s consecutive patients who underwent their initial pulmonary metastasectomy for sarcoma between October 2013 and March 2020. Electronic patient records were interrogated for primary tumour characteristics, number and location of metastases, methods of treatment and survival.

**Results**

A total of 46 patients were included, with a median age at diagnosis of 57.5 years (range 13–80). All the primary tumours were treated surgically, of which 8 (17.4%) patients had positive margins. The mean disease-free interval was 27.9 months*. *The median number of thoracic metastases removed in a single operation was one (range 1 to 11), 28 patients underwent more than one operation for pulmonary metastases (median 2, range 1–6, IQR 1–3). Size of metastases resected ranged from 2 to 110 mm, with the median size metastasis excised being 10 mm (IQR 6–19). 30-day mortality was 0%. Median follow-up time from initial pulmonary metastasectomy was 33.5 months (range 4 to 89 months). 29 patients (63.0%) are still alive. Of the 17 patients (36.9%) who died, the mean survival from pulmonary metastasectomy was 21.9 months. Actual 5-year survival was 8 out of 14 patients (57.1%). 28 patients received chemotherapy, with 15 following metastasectomy.

**Conclusion**

Pulmonary metastasectomy, in our cohort of patients, was associated with no post-operative mortality. As a low-risk procedure, it is an acceptable treatment option with survival in selected metastatic cases reaching 5 years and beyond.

### A281 Characteristics and Postoperative Trajectory of Patients Requiring ICU Admission After Extended Pleurectomy and Decortication for Mesothelioma

#### Chandarana, Karishma, Dr; Koulouroudias, Marinos, Mr; Caruana, Edward, Mr; Dawson, Alan, Mr; Nakas, Apostolos, Mr

##### Glenfield Hospital, Leicester, UK

*Journal of Cardiothoracic Surgery* 2023, **18(Supp 1)**:A281

**Objectives**

Radical surgery in the form of Extended Pleurectomy Decortication (EPD) presents a major physiological stressor to patients undergoing treatment for malignant pleural mesothelioma (MPM).

The interplay of a patient’s susceptibility to organ injury and the stress response to surgery likely determine the occurrence of postoperative organ dysfunction and need for intensive care.

Identification of factors associated with admission to ICU after EPD can help improve perioperative decision-making and patient counselling.

We aim to identify and compare the differences in clinical, oncological and operative characteristics of patients undergoing EPD that require ICU and those who are discharged from recovery to level 2 care.

**Methods**

All patients who underwent EPD for MPM between January 2019 and September 2021 were included in final analysis. Data was collected from operative databases, electronic patient records and the national ICNARC dataset. Statistical analysis was performed using RStudio.

**Results**

74 patients underwent EPD for MPM, with a total of 34 ICU admissions directly from theatre or recovery (46%).

Patients admitted to ICU were more likely to be male (88.5% v. 72.5%, p = 0.041), have advanced stage pT3/4 mesothelioma, (88.2%, v.70.0%, p = 0.057) and receive more units of red cells intraoperatively (2.8 v. 1.2, p = 0.0001).

50% of patients admitted to ICU required advanced cardiovascular support, 18% advanced respiratory support, and 3% continuous veno-venous hemofiltration (CVVH). Median length of ITU stay was 3 days (range 1–30), with all patients successfully discharged to a high dependency unit (HDU).

**Conclusions**

Overall, admission to ICU following EPD for MPM is common and associated with more advanced disease stages and a higher requirement for intraoperative red cell transfusions. Further understanding of factors predisposing to post- operative organ injury can open new avenues for prehabilitation, risk stratification and improved resource allocation.

### A282 Streamlining Pre-operative Investigations and Pathway for Patients Referred to a Thoracic Surgery Service

#### Brahambhatt, Krupali^1^, Mr; Mohammed, Rayhaan^2^, Mr; Rathinam, Sridhar^3^, Mr

##### ^1^Northampton General Hospital NHS Trust, Northampton, UK; ^2^Glenfield Hospital, Leicester, UK; ^3^University Hospitals of Leicester NHS Trust, Leicester, UK

*Journal of Cardiothoracic Surgery* 2023, **18(Supp 1)**:A282

**Objectives**

To design a streamlined process map for pre-operative investigations in referrals to our peripheral clinics. Aimed at enabling better patient experience, efficient use of junior doctor time and cost saving. Our service covers 4 different peripheral clinics. Access to investigations is often limited resulting in repeated and unnecessary blood tests on admission. This has led to delayed operating lists, patient dissatisfaction, negative impact on trust finances and increased junior doctor workload. Lung cancer referrals are discussed at MDT meetings and planned for theatre and as such they should have investigations in keeping with NICE guideline NG45 and Cancer 62-day pathway. With the introduction of ERAS for lung surgery, pre-operative anemia and hypoalbuminemia can be corrected 2 weeks prior to surgery.

**Methods**

We audited preoperative blood investigations taken in keeping with these guidelines and the 62-day cancer pathway for lung cancer referrals. We looked at how many of these were available on prior to admission and which blood tests are carried out on admission. We designed a process map to be carried out at the peripheral clinics. (Fig. 1.) This was implemented in 2 of our peripheral clinics. We then focussed on colleague education on current guidelines especially junior staff.

**Results**

With this process map in place, we found an increase in number of investigations requested at our peripheral clinics in keeping with guidelines. Less blood investigations were requested on admission at our institution with an average cost reduction of £10 per patient. With approximately 500 patients referred to our service this would ultimately result in £5000 savings per annum.

**Conclusion**

Our process map is cost-effective, simple to implement and reduces unnecessary tests. The results can be greatly improved by involvement of all our clinics.
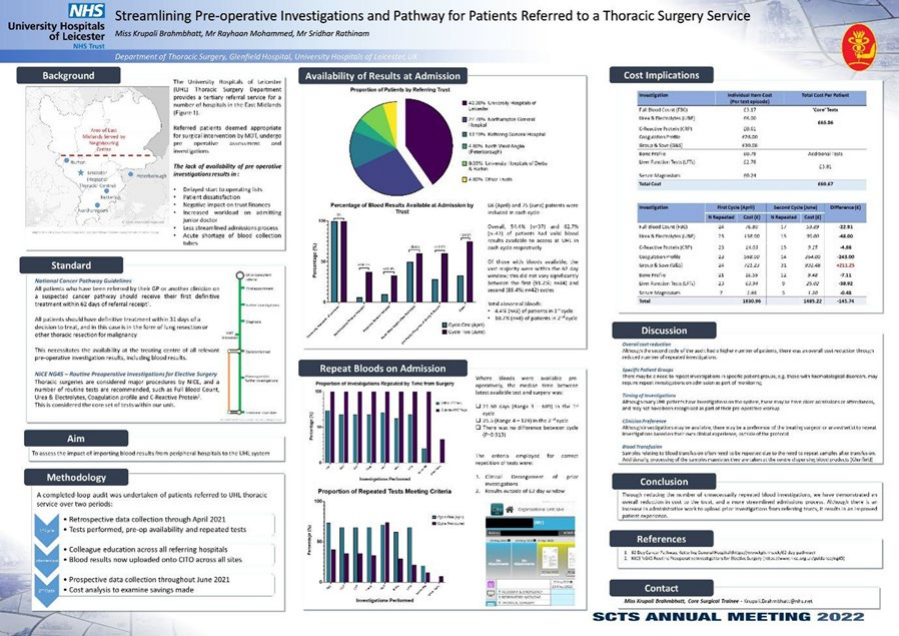


### A283 Smoker Thoracic Physiotherapy Requirements in Anatomical Lung Resections

#### Badran, Abdul^1^, Dr; Lee, Alexander^1^, Dr; Elena, Maria^2^, Miss; Cooper, T^1^, Miss; Neilsen, Louisa^1^, Miss; Alzetani, Aiman^1^, Mr

##### ^1^University Hospital Southampton, Southampton, UK; ^2^University of Southampton, Southampton, UK

*Journal of Cardiothoracic Surgery* 2023, **18(Supp 1)**:A283

**Objective**

Consideration of the effectiveness of thoracic physiotherapy interventions in anatomical lung resections.

**Method**

Prospective cohort of 59 anatomical lung resections and assessment of the physiotherapy interventions in a busy teaching hospital over a 3-month period.

**Results**

60 patients were included. Median age of 71 (36–83). The majority were female (64% vs 36%) with 63% performance status 0. Most were resections for adenocarcinoma (63%) and squamous cell carcinoma (19%). 88% were resected by VATS and 11% open. A minority had no comorbidities (17%), with hypertension (32%) and COPD (23%) being most common. The vast majority of patients 93% were seen on day 1 by physiotherapy. Barriers to this were weekend working (75%). Average length of stay was 4 days for the cohort (3.8 for VATS and 5.3 for Open). Advanced physiotherapy techniques were needed in 18% (n = 11), these were 30% (n = 3) current smokers and the rest ex vs 13% current smokers (n = 6) and ex-smokers in 75% (n = 36) for those that didn’t need advance techniques.

**Conclusion**

We found that elective thoracic patients are more likely to have a reduced LOS and are less likely to require advanced respiratory techniques when reviewed D1PO. This highlights a need for potential service developments within our weekend and Level 2/3 elective thoracic caseload to optimise all patients within service.

### A284 Open Versus Video-Assisted Thoracoscopic Surgery (VATs) Versus Robotic Approaches to Thymectomy – a Systematic Review and Bayesian Analysis

#### Smith, Harry; Chan, Jeremy, Dr

##### Morriston Hospital, Swansea, UK

*Journal of Cardiothoracic Surgery* 2023, **18(Supp 1)**:A284

**Introduction**

Thymectomy has traditionally been performed via a median sternotomy approach. More recently evidence has emerged showing that alternative techniques are adequate if not superior to the median sternotomy; these include video-assisted thoracoscopic surgery (VATS) and robotic surgery – collectively these techniques allow a less invasive, or minimally invasive approach. Our study group aimed to examine the relationship between the approach used for thymectomy and its effect on some defined post-operative outcomes.

**Methods**

A systematic review was conducted adhering to PRISMA guidelines using PubMed, Embase, Cochrane library and Web of Science databases. We included original research articles comparing robotics to open thymectomy to VATs for thymoma, anterior mediastinal masses or myasthenia gravis associated thymomas. Bayesian meta-analysis were performed for mortality, length of stay, recurrence rates, 5-year disease-free survival and complication rates.

**Results**

Our analysis showed no statistically significant differences between the 3 groups on mortality (p = 0.61). Length of hospital stay favours VATs and robotic groups when compare to open (p < 0.05) but no difference was noted between VATs and robotic arm. No statistically significant differences were noted in 5-year disease free survival between 3 groups.

**Conclusion**

We have shown a definite hierarchy in surgical approach to thymectomy from the available original articles in the selected databases when it comes to length of hospital stay, which favours VATs. However, in order to form concrete conclusions regarding, randomised controlled studies of a large magnitude are needed.

### A285 Decision-making, Education, and Staff Confidence Regarding Chest Drain Assessment in the Thoracic Surgical Patient: A Service Evaluation

#### Gopalaswamy, Madhura, Dr; Calvert, Rachel, Mrs; Connelly, Leanne, Mrs; Dunning, Joel, Mr; Waterhouse, Benjamin, Mr

##### South Tees NHS Foundation Trust, Middlesbrough, UK

*Journal of Cardiothoracic Surgery* 2023, **18(Supp 1)**:A285

**Objective**

Chest drains are an important part of the post-operative management of thoracic surgical patients. Timing the removal of a chest drain is important for several reasons.

Removal too soon can result in pneumothorax or pleural effusion necessitating drain re-insertion.

Leaving a drain in longer than required can be a source of infection, cause discomfort and pain, limit mobility, and delay discharge from hospital.

The aims:

1. To assess concordance between ward round and drain chart assessment of air leak
2. To look for any weekend effect: day of the week drains removed (to evaluate if non-thoracic registrars are less comfortable making decisions to remove drains)
3. Assess whether staff feel they are confident assessing chest drains

**Methods**

The study looked at lung resections and was conducted retrospectively over a 6-month period (October 2020- March 2021).

Metrics included variation in length of time with drain in situ by day of operation, chest drain chart entries and compared it to the ward round documentation of the drain.

To support this work and as a baseline for further assessment after intervention, a staff questionnaire was given to measure confidence assessing different chest drain devices.

**Results**

86 lung resections were performed over the 6 months.

Chest drain charts reviewed for 73 patient-days.

- ***17/73 (23.3%) were discordant with the ward round documentation of air leak.***

Chest drains removed by day of the week: *No significant variation.*

- Patients operated on Mon
  - Mean 3.65 d
  - Median 1 d
- Patients operated on Fri
  - Mean 3.25 d
  - Median 1 d

Results from the staff questionnaires showed a mean confidence of 82% for underwater seal and 48% for the flutter bag.

**Conclusions**

There was significant difference between chest drain chart entries and ward round notes of air leak and poor staff confidence especially in assessing flutter bags.

We were pleased that despite preconceptions within the team we found no evidence of a "weekend effect" when it comes to removal of drains.

### A286 The Role of Blood Patch Pleurodesis in the Covid-19 Era: Lessons Learned

#### Mustaev, Muslim, Dr; Hurley, Patrick, Dr; Harrison-Phipps, Karen, Mrs

##### Guy's & St Thomas' NHS Foundation Trust, London, UK

*Journal of Cardiothoracic Surgery* 2023, **18(Supp 1)**:A286

**Objectives**

During the Covid-19 pandemic, the efforts of the cardithoracic surgical units were concentrated on reducing postoperative complications, including persistent air leak (PAL). As management of PAL by Heimlich valve adds on to potential risk of spreading the Covid-19 in the community, the objective of this study was to analyse the efficacy of blood patch pleurodesis (BPP) in the postoperative patients.

**Methods**

From January through October 2021, ten patients, six males (mean age 64.7 ± 11.4 yrs) and four females (mean age 66.3 ± 12.3 yrs) operated on for various indications (pneumothorax (1 pt), empyema (1 pt), mediastinal lymph node dissection (1 pt) and lung cancer (7 pts)) were analysed. All patients developed PAL and underwent BPP using 50–60 mL of autologous blood as sterile once-only procedure.

**Results**

The mean duration of PAL from the index operation to BPP procedure was 13.6 ± 12.3 days, median was 11.5 days (range 2–45 days), the mean period post BPP to drain removal was 5.8 ± 5.1 days, median was 4.5 days (range 1–19 days). Nine patients were discharged home uneventfully, and one patient (10%) was discharged home with Heimlich valve which was removed in 19 days. There were no immediate complications after BPP. None of the patients developed fevers or empyema post procedure.

**Conclusions**

Our study confirms overall efficacy and safety of BPP in the postoperative patients with PAL. BPP provides a satisfactory seal of PAL in majority of patients and may serve as a valid alternative to Heimlich valve and chemical pleurodesis.

### A287 Significantly Reduced Blood Loss From the First Case: Early Outcomes of a New Robot-assisted Lobectomy Programme

#### Harrison, Oliver, Mr; Veres, Lukacs, Mr

##### University Hospital Southampton, Southampton, UK

*Journal of Cardiothoracic Surgery* 2023, **18(Supp 1)**:A287

**Objectives**

To review the early outcomes of a newly established robot-assisted (RATS) lobectomy programme and compare to an established video-assisted (VATS) lobectomy practice.

**Methods**

The case notes from all patients undergoing RATS lobectomy at our institution from the first case performed in May 2021 to October 2021 were reviewed. Cases involving intra-operative frozen section were excluded. A matched series of patients undergoing VATS lobectomy between January 2020–December 2020 were identified and used as a comparison group. All procedures were performed by the same surgeon. Outcomes included operative time, blood loss, length of stay and complications defined as any adverse event delaying discharge by ^3^1 day. Statistical analysis was performed with SPSS (v26). Independent samples t-tests and Mann–Whitney U tests were used to compare parametric and non-parametric outcomes respectively. Data are presented as mean ± standard deviation (SD) or median ± interquartile range (IQR) based on normality testing.

**Results**

Table 1 conveys the key study data. Twenty RATS lobectomy cases were performed during the study period and were matched with 16 VATS lobectomy cases. There were no significant differences in major surgical risk factors between the groups. Operative time was significantly longer in the RATS group (95% CI 8 – 47 min; p = 0.008). There was a weak trend towards decreasing operation time over the study period (r = -0.289; p = 0.216). Intraoperative blood loss was significantly lower in the RATS versus VATS lobectomy group (30 ml vs. 50 ml; p = 0.016). There were no conversions in the RATS lobectomy group compared to 7/35 for the unselected lobectomy cases started VATS in 2020.

**Conclusions**

Evidence supporting the benefits of RATS over VATS is limited. We demonstrate a significant reduction in intraoperative blood loss with a RATS approach, including cases performed on the initial learning curve, when compared to an established VATS lobectomy.
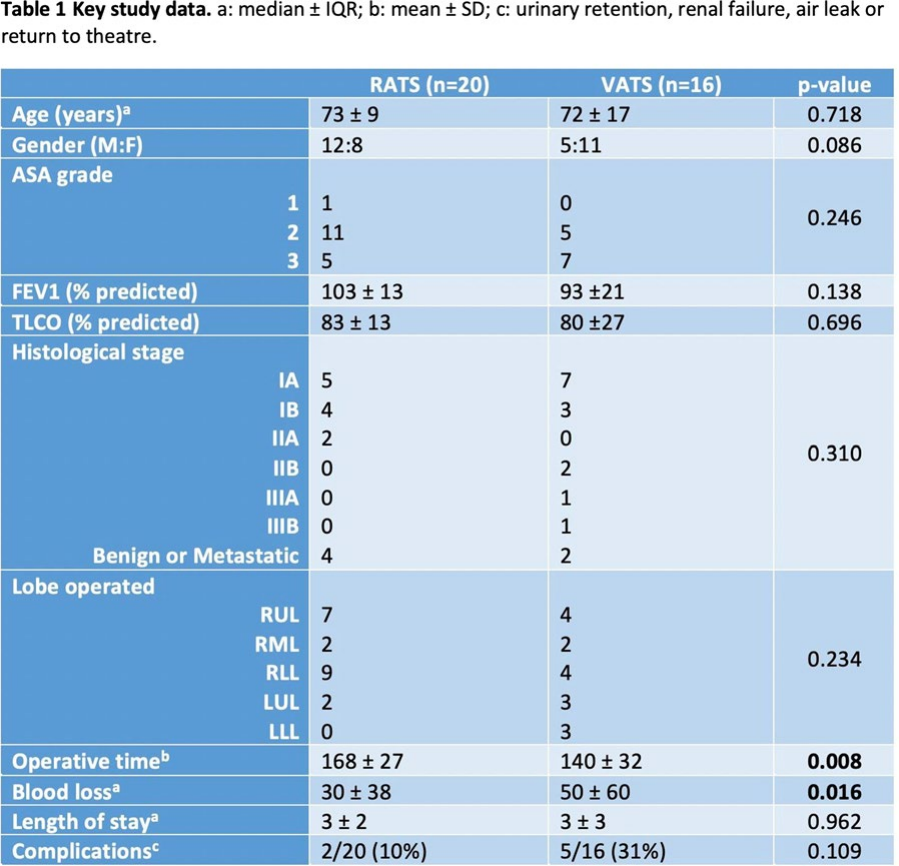


## Transplant and Failure

### A288 AManagement of Bronchial Stenosis in Post Lung Transplantation—Initial Evaluation of Biodegradable Stents

#### Cyclewala, Shabnam, Dr; Padukone, Ashok, Dr; Asadi, Nizar, Mr

##### Royal Brompton and Harefield Hospital, London, UK

*Journal of Cardiothoracic Surgery* 2023, **18(Supp 1)**:A288

**Introduction**

Bronchial stenosis one of the most common airway complications post lung transplantation.

**Methods**

Retrospective analysis of prospectively collected data of patients who have undergone lung transplantation. Data was gathered with regards to the type of interventions used to treat the bronchial stenosis and the outcomes were compared, along with looking at the efficacy of absorbable bio-degradable stents.

**Results**

A total of 524 lung transplantations were performed. 44 Patients developed bronchial stenosis out of which 32 patients required interventions for the stenosis. The most common site for stenosis was found to be bronchus intermedius (28 – 63%) followed by left main bronchus (9 – 20%), right main bronchus (5 – 11%) and left upper lobe bronchus (2%). The patients were treated with initially balloon dilatation (32—72%), cryotherapy (15 -34%) and later endobronchial stents (9 – 18%). With metallic stents (4), 3-have had bronchomalacia, 1 -re-stenting, and 2 had persistent stentosis post removal. The biodegradable stents(5), which have been followed up from 4 to 10 months, have had no reports of bronchomalacia and also decreased the need for intervention to removal the stent.

**Conclusions**

Conventional stents carry a risk of complications. Biodegradable stents have been newly introduced which hold strength initially and degrade over months. It also bypasses the issue of stent removal and show improved FEV1. Follow-up and prospective studies need to be undertaken to compare benefits and subsequent complications.

### A289 VATS Right Lower Bilobectomy Post Bilateral Single Sequential Lung Transplantation (BSSLTX) for Airway Stenosis—Movie

#### Mayer, Nora^1^, Dr; Perikleous, Periklis^1^, Mr; Khoshbin, Espeed^2^, Mr; Asadi, Nizar^1^, Mr

##### ^1^Royal Brompton and Harefield Hospitals, Part of Guy`s and St. Thomas NHS Foundation Trust, Department of Thoracic Surgery, London, UK; ^2^Royal Brompton & Harefield Hospitals, Part of Guy`s and St. Thomas NHS Foundation Trust, Heart&Lung Transplantation, London, UK

*Journal of Cardiothoracic Surgery* 2023, **18(Supp 1)**:A289

https://www.youtube.com/watch?v=XJKXloQxPvA

### A290 Cardiac Power Output Index as a Predictor of Severe Primary Graft Dysfunction and Early Mortality in Cardiac Transplantation

#### Williams, Luke^1^, Dr; Duval, Jean-Luc^2^, Dr; Lim, Sern^3^, Dr; Catarino, Pedro^4^, Mr; Berman, Marius^2^, Mr

##### ^1^NHS Blood and Transplant; ^2^Royal Papworth Hospita, Cambridge, UKl; ^3^University Hospitals Birmingham, Birmingham, UK; ^4^Cedars Sinai, Los Angeles, USA

*Journal of Cardiothoracic Surgery* 2023, **18(Supp 1)**:A290

**Objectives**

Cardiac Power Output Index (CPOi) has been suggested as an easy to measure and useful haemodynamic predictor of severe primary graft dysfunction (PGD) and early mortality after cardiac transplantation. We sought to analyse the utility of the recently published cut-offs for CPOi as a predictor of severe PGD and early mortality.

**Methods**

We retrospectively analysed 250 consecutive adult patients who underwent cardiac transplant between January 2016 to August 2021 at our institution. We used electronic records to calculate CPOi (CIx(MAP-CVP)/451) at admission to ICU (0 h) and 6 h post-operation. We applied the previously determined cut-off of CPOi < 0.34W/m2 at 0 h and < 0.33W/m2 at 6 h to determine the sensitivity, specificity, negative and positive predictive values as a determinant of the combined outcome of severe PGD or 30 day mortality.

**Results**

27 patients were excluded due to incomplete recording of data required to calculate CPOi at one or both timepoints. 16 patients met the primary outcome (6 died, 10 developed severe PGD) within 30 days of their transplant. 14 of these fell below the cut-offs at both timepoints, two fell below the cut-off at one timepoint. A total of 139 patients fell below the cutoffs at both time-points, 30 patients were above the cut-offs at both time points and 54 patients were below the cut-off at one time-point only. The sensitivity of CPOi below < 0.34W/m2 at 0 h and  < 0.33W/m2 at 6 h was 100%, with a specificity of 19.35%, a positive predictive value of 0.1 and a negative predictive value of 1.

**Conclusions**

CPOi can accurately rule out severe PGD and 30-day mortality within 6 h of operation and can help to predict those who will require early mechanical circulatory support, potentially facilitating earlier intervention and improving outcomes in cardiac transplantation.

### A291 'A Window of Opportunity to be Considered': Challenges in Lung Transplantation in Patients with COVID-19 Lung Disease Bridged with ECMO Support

#### Graziano, Giovanni, Dr; Gallagher, Grainne, Dr; Hutchison, Susan, Ms; Pereira, Charlotte, Ms; Soliman-Aboumarie, Hatem, Dr; Kaul, Sundeep, Dr; Khoshbin, Espeed, Mr

##### Royal Brompton and Harefield NHS Foundation Trust, London, UK

*Journal of Cardiothoracic Surgery* 2023, **18(Supp 1)**:A291

**Background**

Lung transplant is a last resort for COVID-19 patients with end-stage lung disease. Despite lack of data for long-term outcomes, multi-institutional case series show that lung transplantation can be carried out successfully with encouraging early outcomes (92% survival at 80 days, Bharat 2021). Extracorporeal membrane oxygenation (ECMO) is a supportive measure for patients with irreversible lung disease following COVID-19 infection as a bridge to transplant.

**Case**

We report a long run of veno-venous (VV) ECMO for COVID-19 of 252 days. This is the longest ever ECMO run documented in a COVID-19 patient. A 41 year-old man with a background of hypercholesterolemia and type-2 diabetes mellitus presented with COVID-19 pneumonitis. Despite optimal medical therapy and rest ventilation, his support escalated to peripheral VV-ECMO, for which he was sedated for the first 60 days. He developed irreversible lung injury with CT evidence of end-stage pulmonary fibrosis. He was referred for consideration of lung transplantation, but was deemed unsuitable due to profound physical deconditioning, manifested as severe central and peripheral muscle wasting, increasing ventilator driving pressure, low tidal volumes (< 2 ml/Kg) and right heart failure.

**Discussion**

This case gives us the opportunity to learn about the challenges associated with the use of ECMO as a bridge to transplant in the context of COVID-19. As illustrated, the patient underwent prolonged sedation, which negatively impacted prognosis as it delayed reconditioning with physiotherapy.

**Conclusion**

In addition to the International Society for Heart & Lung Transplantation (ISHLT) criteria (table) we suggest a multifactorial strategy to improve muscle strength and endurance. This includes optimising nutritional status, and implementing an early, personalised, physiotherapy-led muscle strengthening program, using muscle function tests (effort and non-effort related) to guide progress and inform decision-making.
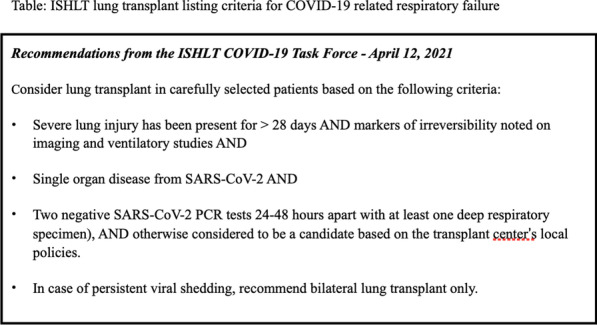


### A292 'Long-Term' Use of Impella—Safe to Do?

#### Ahmed, Hesham, Mr; Alayyar, Mohammed, Mr; Jothidasan, Anand, Mr; Husain, Mubassher, Mr; Stock, Ulrich, Prof; Smail, Hassiba, Ms

##### Royal Brompton and Harefield Hospital as part of Guys and St Thomas Foundation Trust, London, UK

*Journal of Cardiothoracic Surgery* 2023, **18(Supp 1)**:A292

**Objectives**

Impella CP, 5.0 and RP belong to the group of short-term mechanical circulatory support (MCS) devices used for patients with severe heart failure and cardiogenic shock. The Impella is designed for short- term support up to 10 days, but our patients frequently require a longer duration of support. As our patients are often bridged to transplant and the long waiting lists often result in long waiting times, we are frequently forced to prolong the use of the Impella. Therefore, we aimed to assess the safety of prolonged device duration past 10 days.

**Methods**

We present our single center data of 69 patients who underwent Impella placement between May 2017 and May 2021. We divided the patients into two groups, Group A (39 patients) with device duration < 10 days and Group B (30 patients) with device duration > 10 days and compared the occurrence of complications and mortality in the two groups.

**Results**

Median device duration for Group A was 6.08 (± 2.2) and 21.6 (± 11.8) days in Group B (p 0.001). Group A contained 6 Impella 5.0, 27 Impella CP and 6 Impella RP vs. Group B with 18 Impella 5.0, 10 Impella CP and 2 Impella RP (p 0.001).

There were no significant differences between Group A and Group B regarding: haemolysis 52.4% vs. 42.6% (p 0.36), access site bleeding 24.3% vs. 30% (p 0.4), thrombocytopenia 27% vs. 23.3% (p 0.47), vascular complications 8.1% vs. 6.7% (p 0.6), device migration 5.6% vs. 6.7% (p 0.6), pump thrombosis 10.8% vs. 17.9% (p 0.3), device malfunction 0% vs. 3.3% (p 0.4), ventricular arrhythmias 29.7% vs. 23.3% (p 0.38), access site infection 0% vs. 3.3% (p 0.44), sepsis 32.4% vs. 46.7% (p 0.17), ischemic cerebrovascular accident (CVA) 0% vs. 3.3% and haemorrhagic CVA 0% vs 6.8%. (p 0.13).

There was no significant difference regarding pre-implant and pre-explant bilirubin levels in both groups (p 0.35 and p 0.19) and creatinine levels (p 0.2 and 0.06). The platelet count pre-implant showed no significant difference (p 0.07). However, the pre-explant level was significantly lower in Group A (p 0.005).

The mortality rate on Impella in Group A and Group B was 28.2% and 13.3% (p 0.1), respectively.

**Conclusion**

Prolonged device duration has no significant impact on the short-term outcome except on platelet levels. In cases where longer MCS is mandatory careful monitoring might allow longer Impella support.

### A293 Primary Graft Dysfunction (PGD) After Lung Transplantation, Incidence and Outcomes; a Single-centre Experience

#### McGinley, Jack^1^, Mr; Hardman, Gillian^2^, Miss; Parry, Gareth^2^, Dr; Clark, Stephen^2^, Prof; Dark, John^2^, Prof; Fisher, Andrew^1^, Prof; Booth, Karen^2^, Mrs

##### ^1^Faculty of Medical Sciences, Newcastle University, Newcastle-upon-Tyne, UK; ^2^Department of Cardiothoracic Transplantation, Freeman Hospital, Newcastle-upon-Tyne, UK

*Journal of Cardiothoracic Surgery* 2023, **18(Supp 1)**:A293

**Objectives**

Data for the diagnosis and grading of Primary Graft Dysfunction (PGD) following lung transplantation are not routinely recorded in the UK Transplant Registry (UKTR).

This study aimed to examine the incidence and impact of PGD after lung transplantation from a single UK transplant centre.

**Methods**

Data for adult first-time lung transplant recipients in our centre between 1 January 2010 and 31 December 2019 were reviewed. Diagnosis and grading of PGD was established retrospectively by ISLHT criteria.

Unadjusted 90-day, 1-year and 5-year survival by PGD grade was assessed using Kaplan–Meier survival analysis and log-rank tests. Univariable analysis of PGD grade 3 at 72 h post-transplantation, was performed.

**Results**

A total of 424 recipients were identified, with PGD grading available for 401 (95%). The incidence of PGD3 at 72 h was 16% (64 recipients). There was a significant difference in survival at 90-days, 1-year (p =  < 0.0001) and 5-years (p = 0.0002).

Recipients with PDG3 at 72 h had a statistically significant higher mean BMI (26 SD ± 4 versus 24 (± 4) p = 0.0009), more donors with a history of smoking (41 (64%) versus 141 (42%) p = 0.001) and a lower proportion of ‘off-pump’ procedures (23% versus 41% p = 0.04).
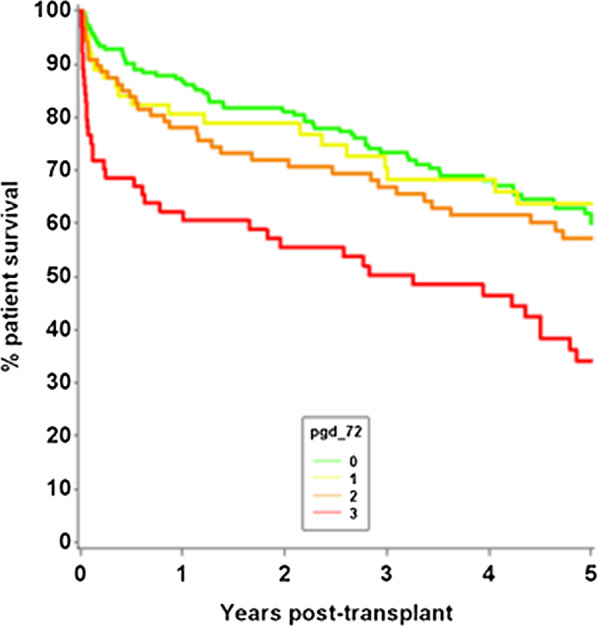


**Conclusions**

These results indicate a comparable incidence of PGD3 to studies outside the UK, and a significant impact on both short- and long-term survival for recipients with PGD3 at 72 h post-transplant.

### A294 Awake Non-intubated Veno-arterial ECMO in Acute Decompensation of Chronic Heart Failure

#### Mohite, Prashant^1^, Mr; Umakumar, Kabeer^2^, Mr; Verzelloni Sef, Alessandra^2^, Dr; Sef, Davorin^2^, Mr; Husain, Mubassher^2^, Mr; Farmidi, Abu^2^, Mr; Marczin, Nandor^2^, Dr; Stock, Ulrich^2^, Prof

##### ^1^Golden Jubilee National Hospital, Glasgow, UK; ^2^Royal Brompton & Harefield NHS Foundation Trust, London, UK

*Journal of Cardiothoracic Surgery* 2023, **18(Supp 1)**:A294

**Objectives**

Acute decompensation in patients of chronic heart failure that cannot be managed with inotropes and diuretics may require short-term mechanical circulatory assist. These patients usually have a good lung function and may tolerate awake peripheral implantation of the veno-arterial extracorporeal membrane oxygenation (VA ECMO) or sedation hold and extubation if ventilated at the time of ECMO implantation. We share our experience of keeping these patients awake during the ECMO support.

**Methods**

53 patients (21 females) with a mean age of 39 (16–63) years received VA ECMO for acute decompensation in chronic advanced heart failure due to cardiomyopathy. We utilized Cardiohelp (Getinge, Sweden) or CentriMag pump (Abbott, USA) and Medos HiLite oxygenator (Inspiration, Germany) for ECMO support. Peripheral ECMO was implanted percutaneously under local anaesthesia without sedation in 51 patients whereas 2 patients received central ECMO via sternotomy. Patients on ventilator support at the time of ECMO implantation were given an early trial of sedation wean and extubation. Awake patients underwent active mobilisation and physiotherapy.

**Results**

The average duration of the ECMO support was 12.1 (2–32)days and awake duration on the ECMO support was 7.5 (1–29)days. Eleven (20.7%) patients died on the ECMO support, 16 (30.1%) patients had myocardial recovery with successful ECMO explant, 20 (37.7%) patients were bridged to the long-term ventricular assist device, 1 patient to short-term VAD, and 5(9.4%) patients were bridged to the heart transplant. The average ITU stay was 22.6 (3–114)days and the average hospital stay was 50.1 (3–250)days. 21 patients died in the hospital with a discharge to home survival of 60.3%.

**Conclusions**

Chronic heart failure patients supported on the ECMO can be maintained awake and can mobilise, eat and drink and undergo physiotherapy. Complications related to mechanical ventilation, sedation, and immobilisation can be avoided.

### A295 Redo-sternotomy in Heart transplantation – Outcomes from the Scottish National Advanced Heart Failure Service

#### Avtaar Singh, Sanjeet Singh^1^, Mr; Das De, Sudeep^2^, Mr; Curry, Philip^2^, Mr

##### ^1^Aberdeen Royal Infirmary, Aberdeen, UK; ^2^Golden Jubilee National Hospital, Glasgow, UK

*Journal of Cardiothoracic Surgery* 2023, **18(Supp 1)**:A295

**Objectives**

Heart transplant recipients with previous cardiac interventions often have poorer outcomes postoperatively. This could be due to increased technical difficulty, subclinical deconditioning, bleeding and infections. We analysed the results from the Scottish National Advanced Heart Failure Service to ascertain if redo sternotomies have poorer outcomes.

**Methods**

103 adults underwent heart transplantation in our institution between January 2011–January 2020. Recipients were divided into 2 groups: Virgin chest (first sternotomy group; n = 72(69.9%) and those with at least 1 prior sternotomy (redo sternotomy group; n = 31(30.1%). Univariable analysis was performed using student’s T test and Chi-squared test. A time to event analysis was used to depict long-term outcomes between the groups. Outcomes of interest were post-operative ECMO, Length of Hospital stay, 30-day survival and 1-year survival.

**Results**

**Table:** 

Description	Virgin Chest (n = 72)	Redo-Sternotomy (n = 31)	p-value
Recipient Age(years)	46.3 ± 12.2	44.8 ± 11.8	0.570
Preoperative Inotropes(%)	38(52.8)	18(58)	0.621
Preoperative IABP(%)	27(37.5)	7(22.6)	0.216
Height(cm)	172 ± 10.1	174 ± 8.2	0.376
Weight(kg)	76.9 ± 12.2	77.5 ± 11.7	0.828
Female(%)	23(31.9)	5(19.2)	0.076
Ischaemic Aetiology(%)	14(19.4)	11(35.5)	0.070
Donor Age(years)	43.3 ± 11.6	37.4 ± 11.5	0.067
Total Ischaemic Time(mins)	188.9 ± 64	194.1 ± 60.5	0.764
Cold Ischaemic Time(mins)	101.4 ± 45.8	117.0 ± 45.9	0.244
Post-operative ECMO	20(27.7)	10(32.2)	0.644
Bypass Time (mins)	226.8 ± 69.8	253.0 ± 105.1	0.299
30-day Mortality	6(8.3)	5(16.1)	0.101
1-Year Survival	61(84.7)	23(74.2)	0.162
Post-operative length of stay(days)	37.6 ± 22.7	33.4 ± 17.8	0.383


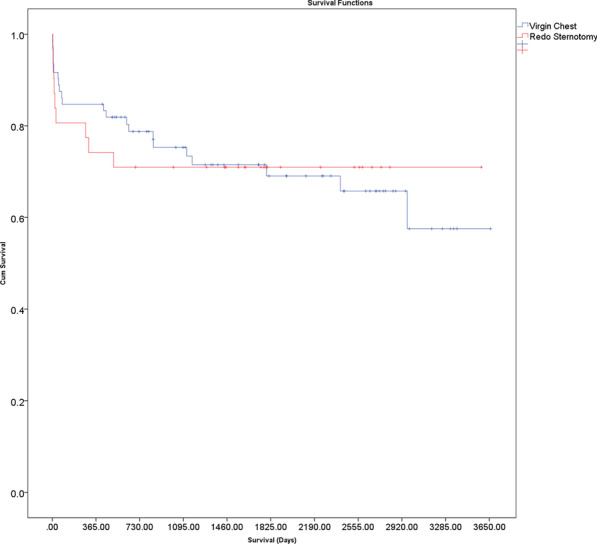


There was a trend towards higher number of patients with ischaemic aetiology and mortality within the first 30 days in the redo sternotomy cohort. There was also a trend towards a younger donor age for these recipients, which may partly explain the equivocal findings.

The Kaplan–Meier curve shows a steep drop within the first year in the redo sternotomy group but no differences were noted at up to 10 years follow-up (Log Rank p = 0.974).

**Conclusion**

There was no statistically significant increase in post-operative length of stay, mortality or post-operative ECMO rates in the redo sternotomy cohort in our study. This could be due to the preference towards younger donors in this cohort.

### A296 Direct Procurement of Thoracic Organs Along With Abdominal Normothermic Regional Perfusion in Donation After Circulatory Death

#### Husain, Mubassher, Mr; Jothidasan, Anand, Mr; Zeschky, Charlotte, Miss; Padukone, Ashok, Mr; Ahmed, Hesham, Mr; Khoshbin, Espeed, Mr; Stock, Ulrich, Prof

##### Royal Brompton and Harefield Hospital as part of Guys and St Thomas Foundation Trust, London, UK

*Journal of Cardiothoracic Surgery* 2023, **18(Supp 1)**:A296

**Objective**

Direct procurement of thoracic organs as compared to using thoraco-abdominal normothermic regional perfusion (NRP), a modified form of extracorporeal membrane oxygenation (ECMO) is the preferred method for thoracic organ procurement in donors after circulatory death (DCD). The use of abdominal NRP alone has proven to significantly improve outcomes from liver transplantation. We have developed a technique for thoracic organ isolation during abdominal NRP that allows successful co-procurement of thoracic and abdominal organs.

**Method**

In order to achieve successful thoracic isolation both the brain perfusion and abdominal volume loss must be prevented. After certification of circulatory death and a period of standoff the thoraco-abdominal incision is performed. Blood is collected either from the donor right atrium or a side-arm of the abdominal NRP circuit to prime the organ care system (OCS) before correct identification and isolation of vascular structures in a systematic way. 1. The left pleura is opened, and the left lung retracted to allow identification and clamping of the descending thoracic aorta above the diaphragm. 2. The ascending aorta is clamped, and the aortic arch vented cranial to the clamp. Abdominal NRP can then commence. 3. The inferior vena-cava is clamped within the pericardium. 4. The SVC and the azygous vein are tied off. 5. The heart is vented on the left and right side before induction of cardioplegia (Diagram 1). Diagram 2 illustrates complete vascular isolation of the thoracic cavity enabling explanation of the heart and lung after delivery of selective antegrade pneumoplegia while abdominal organs continue to be perfused for 2-h before the start of abdominal procurement.

**Results**

Between 2019–21 we successfully performed seven such procurements. Three heart and lungs, three lungs and one heart procurement alone. All organs were successfully implanted with successful immediate outcome in all thoracic and abdominal recipients.

**Conclusion**

Direct procurement of thoracic organs is a feasible option during abdominal NRP. This will potentially expand the thoracic organ donor pool and improve outcome of liver transplantation.

### A297 Prehabilitation in Lung Transplant Candidates- In Person or Virtual, Group or Individual?

#### Holden, Nina, Miss; Winters, Julie, Miss

##### Mater Misericordiae University Hospital, Dublin, Ireland

*Journal of Cardiothoracic Surgery* 2023, **18(Supp 1)**:A297

The objective was to develop a prehabilitation programme to optimise exercise capacity and overall muscle strength in lung transplant candidates.

A virtual pulmonary rehabilitation programme (VPR) was designed. The programme consisted of an eight week virtual exercise class and pre and post programme face to face assessment of the following:

- Six-minute walk test (6MWT)
- Grip strength

Subsequently, a physiotherapist attended pre transplant clinic once per week. Participants were referred to provide the following-

- Exercise testing and advice
- Review of oxygen
- Referral to pulmonary rehabilitation locally

Participants were followed up six monthly or via virtual clinic if required sooner.

During the 10 weeks of VPR, 50% participants were admitted to hospital. Half of participants required an increase in oxygen. One participant passed away. Of eighty potential attendances, 19% of these were missed. Four of the remaining five participants improved 6MWT distance and grip strength.

Analysis of outpatient clinic activity and data is underway.

Lung transplant candidates have complex needs. Virtual intervention provides opportunity to access specialist input without travelling. Challenges include remote monitoring and access to technology. In person contact facilitates real time assessment and tailoring of interventions. It is resource intensive from a staffing and space perspective.

### A298 Post-transplant Outcomes after Bridge to Candidacy and Heart Transplantation with the CentriMag Short-term Ventricular Assist Device

#### Sef, Davorin^1^, Mr; Verzelloni Sef, Alessandra^1^, Dr; Jothidasan, Anand^1^, Dr; Mohite, Prashant^2^, Mr; Raj, Binu^1^, Mr; De Robertis, Fabio^1^, Mr; Stock, Ulrich^1^, Prof

##### ^1^Harefield Hospital, Uxbridge, UK, Royal Brompton and Harefield Hospitals, London, UK; ^2^Golden Jubilee National Hospital, Glasgow, UK

*Journal of Cardiothoracic Surgery* 2023, **18(Supp 1)**:A298

**Objectives**

The role of short-term mechanical circulatory support (MCS) as a bridge to transplant (BTT) or candidacy (BTC) remains unestablished. The CentriMag™ short-term ventricular assist device (VAD) can be used as either BTT or BTC in patients with decompensated end-stage heart failure when there is a contraindication for the use of a long-term device or urgent heart transplantation (HTx). We have analyzed outcomes of patients that were bridged to either transplant or candidacy with the CentriMag™ device.

**Methods**

In this retrospective study, we describe our 15-year single-centre experience of all patients successfully bridged to candidacy or HTx with the CentriMag™ device due to decompensated end-stage heart failure.

**Results**

A total of 29 patients (37.2 ± 13.8 years) underwent implantation of the CentriMag™ device as a BTT (18 patients, 62%) or BTC (11 patients, 38%). The device was used for the left ventricular in 9 (31%), right ventricular in 6 (21%) and biventricular support in 14 patients (48%). Preoperatively, 4 patients (17%) were mechanically ventilated, 4 (14%) had uncertain neurological status, 9 (31%) had intra-aortic balloon pump, 26 (90%) had moderate/severe right ventricular failure, 14 (48%) had renal failure, 5 (17%) had multi-organ failure, and 6 (21%) had previous sternotomy at the time of the device implantation. 30-day mortality after implantation of the CentriMag was 7%. Mean duration of support was 38 ± 44 days. We had no device failure. Post-transplant 30-day and 1-year survival were 90% and 83%, respectively.

**Conclusions**

The CentriMag™ device can be effective in rescuing critically ill patients that are considered unsuitable for long-term VAD or HTx. It can be used as either BTT or BTC with satisfactory posttransplant outcomes.

Table 1. Post-transplant outcomes and complications.

**Table:** 

30-day survival	26 (90)
1-year survival	24 (83)
Stroke	0 (0)
Renal failure	8 (28)
Acute rejection	4 (14)
Mechanical circulatory support	7 (24)
Sepsis	5 (17)
Bleeding	6 (21)
Arrhythmia requiring pacemaker	0 (0)

Data are presented as n(%).

### A299 An Assessment of Risk Scores on the Survival of Post-cardiotomy Extra-Corporeal Life Support (ECLS) Patients

#### Volpi, Sara, Miss; Oyebanji, Oluwatobiloba, Miss; Makariou, Nicole, Miss; Hamid, Umar, Mr; Awad, Wael, Mr

##### St Bartholomew's Hospital, London, UK

*Journal of Cardiothoracic Surgery* 2023, **18(Supp 1)**:A299

**Objectives**

Post-cardiotomy cardiogenic shock (PCCS) is associated with poor outcomes. A number of multivariable risk models have been developed to predict mortality following ECLS, but the relative utility of these models in PCCS is unknown. We assess the predictive value of 4 risk scores on mortality following PCCS.

**Methods**

Patients receiving post-cardiotomy ECLS at our centre between January 2015 and April 2021 were retrospectively risk-stratified using four commonly used scoring systems: SAVE-score, ACEF-II, EuroSCORE II, and PC-ECMO. Area under the receiver-operating curve (AUROC) was calculated for each risk model.

**Results**

112 patients underwent ECLS during this period, 46 (41.1%) patients for PCCS. The median age of the PCCS cohort was 55 (19–79) years, 28/46 (60.9%) were male; 26/46 (56%) had pre-operative LV impairment; 52% were elective procedures. In-hospital mortality was 34/46 (74%). AUROC for SAVE-score was 0.7 (95% CI 0.511–0.889), predicted mortality was 63%, ACEF-II was 0.62 (95% CI 0.41–0.82), predicted mortality was 7.5%, the EuroSCORE II was 0.53 (95% CI 0.313–0.745), predicted mortality was 16.7%, and PC-ECMO was 0.51 (95% CI 0.315–0.710), predicted mortality was 71%.

**Conclusions**

SAVE-score appears to be the better risk model in predicting mortality in PCCS patients receiving ECLS in our patients. Larger studies to validate these models may guide patient selection for PC-ECLS.
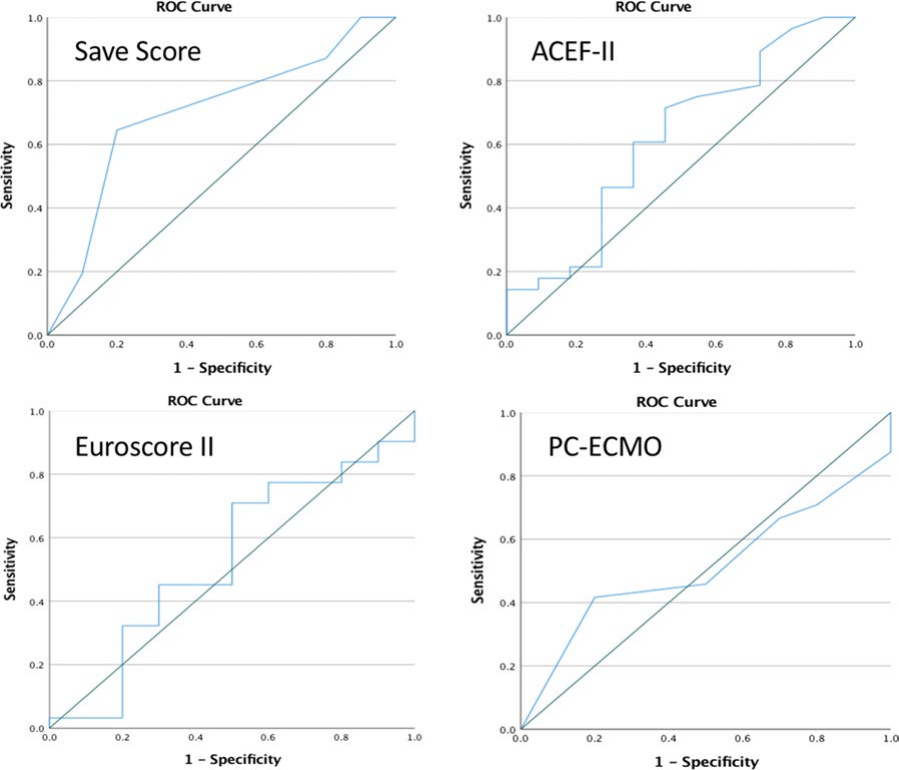


Figure 1: ROC for risk models used to calculate predicted mortality post ECLS.

### A300 Selective Pneumoplegia Delivery System for Procurement in Donors After Circulatory Death

#### Husain, Mubassher, Mr; Jothidasan, Anand, Mr; Zeschky, Charlotte, Miss; Padukone, Ashok, Mr; Khoshbin, Espeed, Mr; Smail, Hassiba, Ms; Stock, Ulrich, Prof

##### Royal Brompton and Harefield Hospital as part of Guys and St Thomas Foundation Trust, London, UK

*Journal of Cardiothoracic Surgery* 2023, **18(Supp 1)**:A300

**Objectives**

Donation after circulatory death (DCD Maastricht III) increases the donor pool, but the induction of organ preservation solutions necessitates a staged approach. As the heart has a low ischemic tolerance it is vital in a DCD retrieval to reanimate the heart quickly without waiting for pneumoplegia to finish. We have devised a selective pneumoplegia delivery system to aid independent lung procurement from the heart to reduce ischemic times.

**Methods**

The figure shows the parts of the customized pneumoplegia delivery system and its use. It consists of two self-inflated soft balloon retrograde cardioplegia cannulae connected with a ¼ inch y-connector and ¼ inch silicone tubes. During cardioplegia the pulmonary artery is transacted, the cannulae inserted into the right and left pulmonary arteries to deliver simultaneous selective antegrade pneumoplegia. After cardioplegia, the heart procurement and its reanimation on the organ care system can commence as the pneumoplegia is running. The self-inflated balloons of the cannulae do not require manual fixation, saving time as there are several litres of pneumoplegia. The same cannulae may be used to deliver the retrograde pneumoplegia.

**Results**

Between 2019–2021 we performed four such procurements, with good immediate outcomes in two of the lung transplant recipients. One lung was declined on basis of poor results of ex-vivo lung perfusion. The fourth was successfully procured but later declined on history of donor drowning.

**Conclusion**

Our customized system allows simultaneous delivery of selective antegrade and retrograde pneumoplegia during and after cardiectomy in a DCD heart and lung retrieval. This may reduce ischaemia of the lungs and improve organ utilisation. More studies are required to assess the potential benefit of our pneumoplegia delivery system.
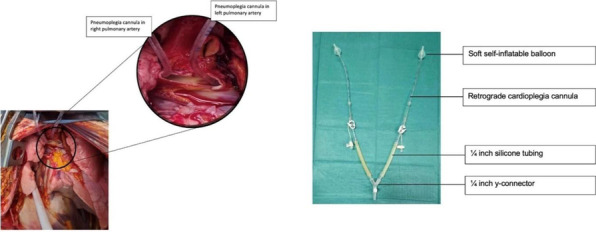


### A301 Impact of Prolonged Total Ischemic Time on the Outcomes of Lung Transplantation

#### Ahmed, Hesham, Mr; Umakumar, Kabeer, Mr; Smail, Hassiba, Ms; De Robertis, Fabio, Mr; Khoshbin, Espeed, Mr; Stock, Ulrich, Prof

##### Harefield Hospital, Uxbridge, UK

*Journal of Cardiothoracic Surgery* 2023, **18(Supp 1)**:A301

**Objective**

According to the international society of heart and lung transplantation guidelines recommended total lung ischemic time for transplantation is less than 6 h. Utilising lungs with longer period of ischaemia however may allow expansion of donor pool and potentially increase transplant numbers. We studied the effect of moderately prolonged ischemic time (> 8 h) on outcome of our lung transplant recipients.

**Methods**

158 patients who underwent bilateral sequential lung transplantation between 2013 and 2017 were studied. Patients with pre-operative extracorporeal membrane oxygenation (ECMO), ex-vivo perfusion, mechanical ventilation and single lung transplantations were excluded. Patients were divided into group A (n = 81) with ischemic time < 8 h for both lungs, group B (n = 36) > 8 h and group C (n = 41) with one lung < 8 and the second lung > 8 h.

**Results**

There was no significant difference in age, gender, preoperative echo findings and diagnosis between groups. Postoperative need for ECMO was 8.6%, 11.1%, 4.9% respectively, p = 0.59. Duration of ventilation (62, 64, 39 h, p = 0.6) and acute rejections (1.2%, 2.8%, 12.2%, p = 0.018). Reintubation rate (18.5%, 30.6%, 22%, p = 0.35) and length of ITU stay (12.36, 19.36, 16.07 days, p = 0.10). 1, 3- and 5-year survival was 73.3%, 73.7%, 69%, p = 0.97.

**Conclusions**

Prolonged lung allograft total ischemic time above 8 h does not impair short- and medium-term outcomes of transplantation however may improve organ utilisation.

### A302 Median Sternotomy vs Clamshell for Sequential Bilateral Lung Transplantation: The Impact of Surgical Approach on Post-operative Lung Function

#### Chilvers, Nicholas^1^, Mr; McPherson, Iain^1^, Mr; Grayling, Michael^2^, Dr; Freystaetter, Kathrin^1^, Ms; Ozalp, Faruk^1^, Mr; Fisher, Andrew^1^, Prof; Parry, Gareth^1^, Dr; Clark, Stephen^1^, Prof; Dark, John^2^, Prof

##### ^1^The Freeman Hospital, Newcastle, UK; ^2^Newcastle University, Newcastle, UK

*Journal of Cardiothoracic Surgery* 2023, **18(Supp 1)**:A302

**Objectives**

The clamshell incision remains the standard approach for bilateral lung transplantation, however, this comes at the cost of high rates of chronic pain and wound complications. Sternotomy offers fewer complications, superior wound healing, good short-term outcomes and preserved chest wall mechanics. We sought to examine the impact of operative approach on post-operative lung function.

**Methods**

Adult patients undergoing bilateral lung transplantation from 1995 to 2019 were identified retrospectively using local transplant databases. Data collected included baseline demographics, ischaemic times, ICU stay, length of stay, survival and post-operative lung function at 1 month, 3 months, 6 months and 12 months.

**Results**

656 patients (177 sternotomy, 448 clamshell, 31 anterolateral thoracotomies) underwent bilateral lung transplant. Sternotomy patients tended to be older (51.1 vs 38.8 years) and less likely to have infective pathology. Post-operative FEV1 was significantly better at 1 and 6 months (p = 0.0105, p = 0.0028) and FVC at 1, 6 and 12 months (p = 0.0345, p = 0.0200, p = 0.0156). There was no difference in ICU stay or 30-day survival.

**Conclusions**

We have described the largest cohort of bilateral lung transplants via median sternotomy in the literature and shown superior lung function compared to the clamshell incision. The importance of this needs to be investigated further, however previous research has shown a correlation between post-operative lung function and 3-year survival.

**Table:** 

Lung function test	Time post op	Clamshell (n = 448)	Sternotomy (n = 177)
FEV1	1 month	64.5 (18.7)	70.3 (18.8)
	3 months	74.2 (21.1)	74.6 (23.3)
	6 months	80.9 (21.9)	78.3 (25.6)
	12 months	82.7 (23.5)	81.3 (23.9)
FVC	1 month	60.6 (16.6)	66.3 (14.8)
	3 months	71.4 (18.2)	74.0 (19.4)
	6 months	79.6 (19.1)	79.9 (20.9)
	12 months	83.4 (20.3)	85.9 (20.0)

### A303 The Sternotomy Approach to Bilateral Lung Transplantation does not Mandate the use of Cardiopulmonary Bypass

#### Chilvers, Nicholas^1^, Mr; McPherson, Iain^1^, Mr; Freystaetter, Kathrin^1^, Ms; Senbaklavaci, Omer^1^, Mr; Fisher, Andrew^1^, Prof; Parry, Gareth^1^, Dr; Clark, Stephen^1^, Prof; Dark, John^2^, Prof

##### ^1^The Freeman Hospital, Newcastle, UK; ^2^Newcastle University, Newcastle, UK

*Journal of Cardiothoracic Surgery* 2023, **18(Supp 1)**:A303

**Objectives**

Bilateral lung transplantation via sternotomy offers many benefits over the standard clamshell incision. However, many consider cardiopulmonary bypass to be mandatory. We have one of the largest experiences of this approach and have more recently employed ECMO or off-pump techniques. We sought to assess our outcomes in these patients.

**Methods**

Adult patients undergoing bilateral lung transplantation from 1995 to 2019 were identified retrospectively using local transplant databases. Data collected included baseline demographics, mode of circulatory support, ischaemic times, ICU stay, length of stay, post-operative lung function and survival.

**Results**

177 patients underwent double lung transplant via sternotomy (147 cardiopulmonary bypass, 18 ECMO, 12 off-pump). In the ECMO group, median ICU stay and length of stay were 4.5 days and 31 days respectively and in the off-pump group, 8 days and 34 days respectively. 30-day survival was 100% in both groups.

**Conclusions**

We have the largest cohort of bilateral lung transplants via median sternotomy in the literature and the only group to describe the off-pump sternotomy approach. ECMO or off-pump techniques in this situation are both safe and feasible, with some minor modifications including the use of a cardiac stabiliser device, and have excellent outcomes. Furthermore, it has advantages over the anterolateral thoracotomy approach as it offers easy access to the aorta and right atrium should unplanned mechanical circulatory support be required. In summary, the sternotomy approach to bilateral lung transplantation does not mandate cardiopulmonary bypass.

### A304 Comparison of Early Postoperative Outcomes in DBD and DCD Lung Transplants: A Single-centre Experience

#### Sef, Davorin^1^, Mr; Verzelloni Sef, Alessandra^2^, Dr; Jothidasan, Anand^3^, Dr; Raj, Binu^2^, Mr; Trkulja, Vladimir^4^, Prof; De Robertis, Fabio^2^, Mr; Stock, Ulrich^2^, Prof

##### ^1^St. Bartholomew's Hospital, London, UK, Barts Health NHS Trust, London, UK; ^2^Harefield Hospital, Royal Brompton and Harefield Hospitals, London, UK; ^3^Harefield Hospital, Uxbridge, UK; ^4^Medical School, University of Zagreb, Croatia, EU

*Journal of Cardiothoracic Surgery* 2023, **18(Supp 1)**:A304

**Objectives**

Utilization of donation after circulatory death (DCD) donors can decrease donor shortage in lung transplantation (LTx). There is increasing evidence that early clinical outcomes following DCD LTx are satisfactory and could be comparable with the results from brain dead (DBD) donors. We aimed to compare the early postoperative outcomes of LTx from DBD and DCD donors.

**Methods**

All consecutive LTxs performed between April 2017 and March 2019 at our centre were included. Donor characteristics and recipient preoperative, intraoperative and early postoperative characteristics were analyzed and compared between DBD and DCD LTx. Redo LTx and single LTx were excluded.

**Results**

Out of 105 patients, 25 (24%) were DCD LTx. Donors’ and preoperative recipients’ characteristics were comparable between both subgroups. There were no statistically significant differences between the two subgroups in terms of donor demographics and preoperative recipient characteristics, except for higher incidence of female gender, aspiration and inotropic support requirement in the DBD donors. Intraoperatively, mechanical circulatory support (MCS) was more common in DCD LTx (56% vs. 36%). MCS duration (332 vs. 166 min, *p* = 0.046), and first (*p* = 0.003) and second lung (*p* = 0.010) ischemia time were longer in the DCD group. Postoperatively, DCD recipients more commonly required ECMO (32% vs. 8%, *p* = 0.004). Postoperatively, patients from DCD group had significantly higher incidence of stented chest (*p* = 0.008), drainage (*p* = 0.001) and peak lactate level (*p* = 0.023).

**Conclusions**

DCD donation has increased our lung transplant activity by almost 25% and early postoperative outcomes are in general comparable with those achieved after DBD LTx. However, we have observed a higher need for both intraoperative and postoperative MCS in DCD LTx which could influence clinical outcomes, although further studies are required.

**Tbl 1.** Early postoperative outcomes.

**Table:** 

Early postoperative outcomes	DBD	DCD	P
Mechanical ventilation (hours)	32.8 (19.3–56.8; 2–992)	42.5 (26.8–69.0; 6.5–640)	0.122
Tracheostomy	28 (35.0)	11 (44.0)	0.420
ECMO [VA/VV]	6 (7.5) [4/2]	8 (32.0) [7/1]	0.004
Drainage 24 h (mL)	1062 (806–1319; 400–5800)	1625 (1037–3275; 400–5900)	0.001
Delayed chest closure	8 (10.0)	8 (32.0)	0.012
Renal replacement therapy	21 (26.2)	13 (52.0)	0.019
Sepsis	11 (13.8)	6 (24.0)	0.241
Peak lactates 24 h (mmol/L)	4.9 (3.3–6.3; 1.5–14.4)	6.6 (3.9–10.6; 2.3–17.0)	0.023
Hospital length of stay (days)	28 (21–52; 2–163)	34 (18–52; 6–105)	0.772

Data are count (%) or median (quartiles; range).

### A305 Eradication of Aspergillus Fumigatus Following Cardiothoracic Transplantation: A Complex Case – The MDT Solution

#### Asemota, Nicole, Dr; Sitaranjan, Daniel, Mr; Osman, Mohamed, Mr; Kaul, Pradeep, Mr

##### Royal Papworth Hospital NHS Foundation Trust, Cambridge, UK

*Journal of Cardiothoracic Surgery* 2023, **18(Supp 1)**:A305

**Objectives**

We present a case of a 48-year-old female who, in fewer than 10 years, underwent aortic valve surgery and then subsequent cardiac & renal transplantation. Her progress was complicated with a mycotic aortic aneurysm and sternal osteomyelitis. Despite posing such a complex surgical challenge, fungal eradication was achieved.

**Methods**

She initially presented with severe aortic and tricuspid regurgitation secondary to infective endocarditis with renal infarction. She had a known muscular VSD. She underwent a Konno procedure with aortic valve replacement, tricuspid annuloplasty and VSD closure. She suffered with ongoing heart and renal failure eventually requiring listing for transplant.

She underwent redo-sternotomy and heart & kidney transplant making an excellent recovery. She developed a wound infection, growing aspergillus fumigatus which remained resistant to antifungal therapy and debridement. An interval CT scan also revealed an ascending aortic mycotic aneurysm.

She underwent ascending aortic mycotic aneurysm excision and replacement with a homograft. Our plastic surgical colleagues then performed a musculocutaneous free flap with vastus lateralis to close the ste**r**nal defect.

**Results**

She has no further clinical or microbiological evidence of A.fumigatus. She has a great quality of life with excellent allograft function. She continues to be followed up by the cardiac and renal transplant and plastic surgery teams, with close microbiology and radiology input.

**Conclusion**

This case provides evidence that invasive A.fumigatus infection can be eliminated with combined surgical, topical and systemic drug treatment even in an immunocompromised patient. Furthermore, it highlights the importance of a multi-disciplinary team approach in decision making and treatment.

Informed consent to publish had been obtained.

### A306 Donor Cause of Death in Heart Transplantation and Its Effect on Post-Transplant Survival

#### Surendran, Arthika^1^, Dr; Mascaro, Jorge^2^, Mr

##### ^1^Queen Elizabeth Hospital, Birmingham, UK

*Journal of Cardiothoracic Surgery* 2023, **18(Supp 1)**:A306

**Objectives**

There are no concrete analyses in the UK that assess the effect of donor cause of death (CoD) on survival in heart transplantation (HTx). We sought to evaluate whether the various CoDs cause significant short- and long-term survival differences in HTX recipients.

**Methods**

We evaluated the registry for all adult HTX recipients at ***** Hospital from 2011 to 2021 and their adult donors. Recipients were stratified based on CoD into road traffic accidents (RTA), intracranial haemorrhage (ICH), Hypoxic brain damage (HBD) or other. Kaplan–Meier (KM) curves depicted the 24-h, 30-day, 1-year, and 5-year survival of the CoD groups with the remaining study population (RSP). Further, Cox proportional hazards survival models were used to estimate the effect of CoD adjusting for recipient and donor age and gender.

**Results**

267 HTX donors were identified, of which, 26.2% died from RTA and 42.6% died from ICH. For RTA, KM analyses showed a significant (P < 0.05) inverse association in mortality at all time intervals as compared to RSP for RTA. In contrast, ICH showed a significant positive association at all time intervals as compared to RSP for ICH. In adjusted models omitting donor age and gender, RTA and ICH were significant, but when those variables were included, donor death by RTA or ICH were not significant.

**Conclusion**

Donor cause of death, specifically RTA increasing survival and ICH decreasing survival, showed to be a significant predictor of short- and long-term mortality in heart transplant recipients. However, when donor age and gender were accounted for, there was no significance: for RTA, it was most likely the robust health status of the donor that accounted for the protective effect while for ICH, it was the frailty.

## Author Index

### A

**Table:** 

Abah, U	312, 333, 396, 398, 401
Abba, P	217
Abbas, M	360
Abbas, S	86, 168
Abbasciano, R	127, 151, 161
Abdelbar, A	107
Abdelhadi, A	330
Abdelrahman, A	177
Abdul Hakeem, M	27, 28, 56
Abdul Khader, A	120, 274, 370
Abdullahi, Y	63
Abousteit, A	213
Acharya, A	298
Acharya, M	64
Ackah, J	375
Adams, B	29
Adebayo, A	191, 203
Adikoesoema, Mohamad S	69
Afoke, J	108, 179
Agrawal, S	355
Ahearn, U	261
Ahern, S	181
Ahmadi, F	291
Ahmadi, N	280, 291
Ahmed, A	124, 276, 311
Ahmed, E	25
Ahmed, H	400, 428, 433, 439
Ahmed, I	96, 236, 288, 289, 290
Ahmed, U	126
Ahmed-Issap, A	312, 333, 401
Akbarzad-Yousefi, A	275
Akberali, U	66
Akhtar, R	14
Akowuah, E	127, 167, 175, 187
Aktuerk, D	142
Al Attar, N	161
Aladaileh, M	327, 328, 329, 442
Alam, R	147, 161
Alayyar, M	428
Ali, J	131
Aljanadi, F	43, 314
Alkalbani, R	161
Allam, M	175
Allen, C	344
Allen, J	427
Allen, R	379
Almeida, Ana B	303, 305
Alphonso, N	211, 212
Alqudah, O	219, 382
Alshammari, A	326, 338, 340, 342, 370, 388, 413
AlShiekh, M	84
Alvarado, P	392
Alvarez Gallesio, J	338, 340, 342
Alwis, S	11, 306
Alzetani, A	243, 267, 315, 400, 415
Amin, F	180
Amoros Rivera, C	442
Amos-Hirst, R	209
Andrews, D	212
Angelini, G	25, 67, 76, 77, 79, 85, 102, 104, 127, 135, 138, 143, 144
Annamaneni, R	348
Ansaripour, A	161
Antolin, R	256
Anzaar, Ahamed A	161
Apicella, G	86, 168
Arcegono, T	256, 258
Aresu, G	280, 291, 346, 363
Argyle, R	177
Argyriou, A	161
Ariyaratnam, P	355
Arjomandi Rad, A	179
Arnold, P	225
Aroori, S	153
Asadi, N	306, 309, 422, 423
Asemota, N	444
Asemota, O	291
Ashraf, Muhammad A	30, 388
Ashraf, S	87
Ashrafian, L	380
Ashry, A	225
Asimakopoulos, G	6, 7, 40, 46
Asonitis, A	46
Asopa, S	281
Athanasiou, T	7, 8, 9, 63, 110, 120, 149, 194
Athansiou, T	11
Attia, R	130
Aujla, H	191
Austin, C	208, 223, 224, 226
Avci Demir, F	169
Avlonitis, V	100, 161
Avtaar S	432
Avtaar Singh, S	294
Aw, TC	332
Awad, W	31, 137, 201, 436
Ayrton, L	378

### B

**Table:** 

Bader, V	214, 215
Badran, A	106, 109, 243, 300, 344, 400, 415
Badran, D	106, 109, 300, 344
Baghai, M	125, 169, 171
Bagona, L	245
Bahrami, T	72, 84
Baig, K	30
Bakir, A	267
Bakr, L	123
Ball, P	174
Balmforth, D	45, 83, 142
Bannister, C	250
Baquedano, M	223
Baranowski, R	336, 359, 372, 386, 387
Bardolia, C	183
Barnard, J	105
Barnard, S	354
Barrett, S	221, 403, 407
Bartley, T	238, 239
Bartosik, W	219, 382, 385
Bartram, J	64
Barve, R	276
Basak, B	373
Basharat, K	391
Basilio, K	256
Batchelor, T	375
Bate, C	238
Baxter, J	235, 241
Beattie, G	140
Beaumont, E	119
Begum, S	326, 332
Bekker, H	371
Benedetto, U	25, 67, 79, 102, 104, 135, 138, 143, 144
Bennett, J	23
Bennett, S	183
Bentham, J	217
Berman, M	235, 241, 424, 427
Betts, K	211, 212
Bhag, G	213
Bhagra, S	427
Bharucha, T	267
Bhaskaran, Anusuya P	277
Bhaskaran, Arya P	277
Bhaskaran, India P	277
Bhatti, F	87
Bhudia, S	69, 72, 84, 161
Bibleraaj, B	239
Bingley, P	151
Birdi, I	86, 168
Birdsall, D	306
Bishay, E	365
Biswas, S	393
Blair, Joyce B	256
Bleetman, D	125, 169, 171
Bleibleh, S	126
Blythe, A	318
Boateng, M	118
Boele, F	134, 371
Bola, H	108
Bone, G	315
Booth, K	161, 273, 429
Booth, S	274, 370, 388, 413
Booton, R	394
Borger, M	50
Bose, A	48
Botha, P	209, 268
Boulemden, A	86, 168
Boyle, M	207, 225
Brahambhatt, K	414
Braidley, P	176
Braithwaite, S	19
Brazier, A	312, 333, 401
Breen, D	374
Brennan, M	265
Briant, Z	401
Briffa, N	167
Brizard, C	212
Brown, C	306
Brown, D	229
Brown, J	161
Brown, L	216
Brown, R	244, 247, 248, 252, 257, 265, 317, 327, 328, 329, 442
Brumpton, M	183
Brunelli, A	409
Bruno, Vito D	10, 66, 85, 173
Brunswicker, A	378
Buchan, K	294
Buderi, S	326, 332, 338, 340, 342
Bueser, T	119
Butler, C	164
Butt, S	92

### C

**Table:** 

Cabolis, K	145
Cacciottolo, P	427
Cahill, J	234
Calvert, R	349, 364, 417
Cannoletta, M	46
Capoccia, M	51
Caputo, M	25, 222, 223
Carroll, B	351
Cartwright, N	167
Caruana, E	302, 353, 389, 412
Caruso, V	149
Casey, A	96
Cassidy, R	318
Casula, R	194
Catarino, P	424
Chacko, J	108, 125, 179
Chadwick, A	251
Chambault, Aimee-L	216
Chambers, J	167
Chan, C	298
Chan, J	5, 60, 67, 76, 77, 87, 89, 102, 116, 117, 121, 143, 161, 397, 416
Chan,Shie W	196
Chandarana, K	262, 302, 353, 412
Chaney, U	229
Chaubey, S	64, 108
Chaudhry, M	55
Chaudhuri, N	409
Chauhan, I	46
Chavan, H	326
Chawla, A	34
Cheng, T	276
Cheng, Yeu Wah M	276
Chidambaram, S	298
Chilvers, N	395, 440, 441
Chivasso, P	10
Chiwera, L	236
Christodoulidou, M	12
Chrysikopoulou, M	55
Chubsey, R	339, 389
Cianci, V	87, 89
Clapon, I	250
Clare, C	68
Clark, S	114, 115, 275, 429, 440, 441
Clayton, T	119
Coats, L	205
Cocomello, L	144, 223
Codispoti, M	112
Cole, A	368
Colombino, Anna M	258, 336
Comanici, M	120, 130
Combellack, T	313, 319, 325, 334, 379
Conneely, J	327
Connelly, L	349, 364, 417
Connolly, K	245
Coonar, A	280, 291, 346, 363
Cooper, T	243, 415
Coppola, G	72
Cormack, S	73
Cormican, L	257
Crosbie, P	394, 396
Crucean, A	268
Crush, J	157
Curry, P	137, 432
Cyclewala, S	422
Cypel, M	366

### D

**Table:** 

D'Alessio, A	19, 263, 264
Daley, M	212
Dalrymple-Hay, M	98, 128
Daly-Devereux, M	345
Danaher, D	240
Dandekar, U	161
Danton, M	215
Dark, J	275, 440, 441
Darling, G	366
Darzi, A	298
Das De, S	432
Dawson, A	99, 412
Day, J	24
De Costa, J	238
De Franco, V	154
De Garate, E	85
De Paulis, R	50
De Perrot, M	366
De Rita, F	205, 218
De Robertis, F	72, 84, 435, 443
De Silva, R	82, 280
De Sousa, P	274, 370, 388, 413
Dean, A	187
Dearling, J	161
Debnath, P	284
Deehan, B	161
Deglurkar, I	52, 103
Derobertis, F	69
Derobrtis, F	439
Deshpande, R	92, 125, 169, 171
Desouza, Abigail-S	315
Devbhandari, M	313, 325, 413
Dhannapuneni, R	213, 225
Dhuga, Y	288, 290
Di Tommaso, E	10, 66, 85, 135, 138, 161
Diamond, O	318
Dilworth, Joseph M	296
Dimagli, A	67, 76, 77, 79, 102, 104, 135, 138, 143, 144
Divya, A	82
Dixit, P	108
Dixon, L	66, 79, 85, 135, 138, 144, 161
Djordjevic, J	263, 264
Docherty, C	388
Dodd, M	119
Doddakula, K	296
Doherty, P	187
Dominic, C	284
Donahoe, L	366
Dong, T	67, 76, 77, 104, 143
Doonan, R	23
Doshi, H	137
Downes, A	351
Drury, N	209
Dubecz, A	303, 305
Duckett, S	183
Duncan, A	57
Dunn, N	238
Dunning, J	239, 270, 349, 395, 417
Dutta, S	111
Duval, J	157, 424

### E

**Table:** 

Eagle-Hemming, B	191, 203
Eaglestone, E	263, 264
Earnshaw, C	330, 390
Eaton, D	247, 252, 257, 317, 327, 328, 329, 407, 442
Eckersley, M	142, 372
Edwards, J	355
Efthymiou, C	97
Elango, M	75
El-Dean, Z	151
Elena, M	415
Elfadil, A	46
El-Gamal, I	361
Elhassan, H	107
El-Hilly, A	30
Elliot, J	327
Elmahdy, W	51
El-Shafei, H	70
Elshafie, G	80
ElSherbini, A	208
Elsiddig, M	118
Elston, V	330
Endean, A	222
Enemosah, I	153
Eranki, A	21, 22, 158
Eskandari, M	169
Evans, N	131, 336
Evans, P	149
Evison, M	378

### F

**Table:** 

Fabroa, S	245
Fairhurst, C	187
Fallouh, H	365
Fang, Chen C	112, 304, 352
Fanning, N	403
Farinelli, E	380
Farmidi, A	72, 431
Ferguson, J	395
Ferrett, J	245
Field, M	5, 12, 14, 23, 24, 27, 28, 56, 261
Fisher, A	429, 440, 441
Fisher, R	14
Fisichella, S	8
Fitzmaurice, G	384, 403
Fitzpatrick, T	257
Fleck, R	94, 221
Fleet, B	270, 286, 293
Fleming, C	407
Folaranmi, O	175
Fontaine, E	393, 394, 399
Francis, J	219
Frattolin, J	9
Fredericks, S	228
Freystaetter, K	129, 395, 440, 441
Friedrich, O	217
Fudulu, D	67, 76, 77, 79, 102, 135, 143, 144
Fuentes-Warr, J	219

### G

**Table:** 

G Malvindi, P	37
Gadallah, B	442
Gaer, J	84
Gallagher, D	257
Gallagher, G	425
Gallesi, Jose A	326
Gama de Abreu, M	303
Gamal, M	212
Ganesananthan, S	184
Gannon, R	236
Garg, S	69, 72, 249
Garner, M	398
Gatta, F	35
Gemelli, M	10, 67, 79
Generali, T	205
George, J	59, 60
George, S	66, 173
Ghazarians, N	282
Ghosh, S	126, 312, 333, 401
Gibb, M	262
Giblin, S	253
Gkikas, A	376
Gnanalingham, S	271
Godolphin, P	376
Goodwin, A	129, 175
Gopalaswamy, M	349, 417
Goulden, C	276
Gradinariu, G	70, 137, 161
Graham, T	126
Granato, F	311, 393, 394, 396, 399
Grandjean, J	68, 132
Grant, S	197, 393, 394, 396, 398, 399
Grayling, M	440
Graziano, G	425
Green, J	161
Gregg, A	174
Guerrero, R	207, 213, 225
Guida, G	79, 85
Guida, M	85
Guo, A	17
Gurney, S	330

### H

**Table:** 

Habib, A	312, 333, 401
Hadjinikolaou, L	39
Hafiz, I	100
Hagmeijer, R	68
Halfwerk, F	68, 132, 192
Hambly, J	142
Hamid, U	31, 436
Hamilton, R	202, 297
Hammad, W	324
Hanna, G	347
Hanna, L	298
Haq, I	40, 53
Haq, M	284
Haqzad, Y	55
Hardman, G	429
Harfield, J	16, 128
Hargrave, J	358, 359
Harky, A	161, 213, 216, 276, 284
Harraz, A	194
Harrington, B	161, 273
Harrington, D	27, 28, 56, 261
Harris, W	25
Harrison, O	420
Harrison-Phipps, K	419
Hartley, J	280
Hartley, P	6
Hasan, A	205
Hasan, R	161
Hashmi, F	311, 360
Hashmi, Syed F	292
Haworth, K	164
Hawwash, N	292
Haycox, A	24
Hayes, T	197
Hayre, S	118
Healy, D	345, 351
Heatlie, G	183
Hernandez, L	304, 352
Hewitt, K	231, 238, 378
Higgins, P	407
Hildick-Smith, D	182
Hill, J	318
Hinde, S	187
Hing Chi, Kristie Hing C	169
Hoffman, R	326, 332, 338, 340, 342
Hogan, J	219, 363, 382
Holden, N	434
Holland, L	147, 182
Holmes, C	129, 354, 395
Hoppe, S	411
Horsfall, G	161
Hossack, M	14
Huddart, H	378
Hughes, S	307, 405
Humphries, S	405
Hunt, I	320, 322, 331
Hunter, S	159, 160
Hurley, P	419
Husain, M	428, 431, 433, 438
Husemann, Z	233
Hussain, A	64, 92
Hussein, N	409
Hutchison, S	425
Hutton, S	236
Hyde, J	147

### I

**Table:** 

Ike, D	83, 387
Imran Hamid, U	174
Inman, C	231
Internullo, E	375, 390
Iqbal, A	126
Iqbal, Y	92, 126
Irvine, M	385
Iyer, A	111

### J

**Table:** 

Jaber, O	209, 217
Jackson-Wade, R	387
Jacob, A	201
Jahangiri, M	99
Jain, S	312, 333, 401
Jakub, M	86
Jansen, K	205
Jansen, M	132
Jarral, O	8, 30, 83
Jarvis, M	55
Jawarchan, A	161, 249
Jeganathan, R	174
Jenkins, D	427
Jin, Xu Y	180
Jingco, F	258
Joel-David, L	191, 203
John, A	52
Johns, J	134
Johnson, T	66
Jones, C	207
Jones, M	43, 161, 208, 314
Jones, Mary E	244
Jones, N	280, 291
Jones, T	209, 268
Jordan, S	326, 332, 338, 340
Jos, H	31
Joseph, B	84
Joseph, D	292
Joshi, V	393, 399
Jothidasan, A	428, 433, 435, 438, 443
Justo, R	211

### K

**Table:** 

Kabir, S	208
Kadlec, J	219, 382, 385
Kakos, C	376
Kalkat, M	234, 307, 361, 365, 405
Kamalanathan, K	330, 390
Kandasamy, K	222
Kaniu, D	326
Kapur, A	371
Kar, A	368
Karia, C	348
Karsan, R	140
Karthikeyan, S	52, 103
Karunanantham, J	131
Karuppannan, M	48, 177
Kassai, I	209, 217
Kaul, P	241, 427, 444
Kaul, S	425
Keane, C	403
Kearns, D	19
Keiralla, A	19
Kellner, P	303
Kelly, M	247, 317
Kemp, B	19
Kenawy, A	27, 28, 56
Kendall, S	167, 175
Kennedy, F	403
Kenyon, L	234
Keshavjee, S	366
Kesieme, E	215
Kew, Ee P	320, 321, 391
Khalil, H	361
Khan, H	64, 92, 125, 169, 171
Khan, J	112
Khan, M	400
Khan, N	94, 209, 221, 268
Khan, T	51, 53
Kho, J	161, 180
Khodaghalian, B	207
Khor, B	307, 361
Khoshbin, E	84, 309, 423, 425, 433, 438, 439
King, E	319
King, J	394
Kinsella, A	242, 255
Klaassen, R	192
Knight, B	215
Knowles, A	177
Kogkas, A	347
Komber, M	355
Kornaszewska, M	313, 319, 325, 334, 379
Korre, S	348
Koskolou, S	325
Kothari, N	216
Kotta, Prasanti A	75
Koulouroudias, M	412
Kouritas, V	219, 382, 385
Krasopoulos, G	19, 190, 263, 264
Kreaden, U	357
Krishnadas, R	375
Krishnamoorthy, B	292
Krysiak, P	393
Kubiak, K	348
Kuduvalli, M	12, 23, 27, 28, 56, 261
Kumar, N	201
Kumar, P	59, 60, 87, 89, 161
Kumar, T	191
Kumar, U	202, 297
Kuo, J	16
Kutty, R	213, 225
Kutywayo, K	348, 389
Kwok, Chun S	183
Kydd, A	427

### L

**Table:** 

Lai, F	65, 78, 127, 191, 203
Lallmahomed, N	397
Lampridis, S	376
Langanay, T	50
Large, S	131, 427
Laskar, N	167
Laskawski, G	177
Lau, K	386, 410
Laufer, G	50
Law, Jacie J	346
Lawler, Z	442
Lawlor, D	223
Layson, R	69
Layton, G	39, 151, 161
Lee, A	415
Lee, G	211
Lee, M	336, 358, 359, 386, 392
Leone, F	80
Leung, K	125, 169, 171
Leung, Kristie Hing C	171
Leung, M	370, 413
Levine, A	239
Lewis, C	427
Lhote, F	397
Lim, E	274, 332, 370, 388, 413
Lim, K	41
Lim, Ru j	69
Lim, S	424
Limbachia, D	161
Linehan, K	95
Liu, G	108, 179
Lodhia, J	409
Longbone, T	134
Loo, Peh S	354
Lopez-Marco, A	29, 45
Lotto, A	213, 225
Loubani, M	35, 55, 80
Low, Mei K	151
Lukban-Bunalade, R	245
Luthra, S	37, 42
Lynch, W	192

### M

**Table:** 

Madine, J	14
Magboo, R	236, 256
Magpantay, A	251
Mahendran, K	312, 333, 401
Mahmood, Z	70, 163
Mahoud, L	161
Makam, R	161
Makariou, N	436
Malvindi, P	42
Mangel, T	29, 45, 321, 405
Manoharan, G	174
Manoj, S	351
Mansour, S	124
Mantio, K	312, 333
Marathe, S	211, 212
Marczin, N	69, 120, 431
Mariani, S	68
Mariscalco, G	39
Markides, C	194
Marsico, R	39, 151
Martinez, L	427
Martin-Ucar, A	352
Martorella, G	228
Mascaro, J	445
Masood, S	245
Masraf, H	37
Massey, J	159, 160, 176
Mastracci, T	29
Mayer, N	306, 309, 423
Mayooran, N	86, 389
McGinley, J	429
McGuinness, J	221
Mcgurk, C	30
McInerney, N	327
McInerney, P	153
McKeon, E	242, 248
McLean, A	215
McLoughlin, J	403, 407
McManus, B	111
McManus, K	318
McNaught, H	364
McNeilly, G	229
McPherson, I	205, 440, 441
Meghani, N	378
Mehat, N	190
Mehdi, R	365
Mehta, D	5
Mellor, S	216
Menon, S	401
Mensah, K	46
Messer, S	427
Metwalli, A	11, 57, 180, 245, 249
Meuris, B	50
Miksza, J	65, 78
Miller, D	191
Milton, R	409
Missouris, C	180
Mitchell, A	187
Mitchell, N	187
Mittal, A	64, 92
Moawad, K	161
Moawad, N	16, 98, 128
Modi, A	147, 161, 281
Modi, S	288, 290
Mohamadzade, N	173
Mohamed Ahmed, E	10
Mohamed, W	124
Mohammed, A	73
Mohammed, R	414
Mohite, P	137, 431, 435
Monaghan, M	169
Montaque, M	173
Montgomery, L	314
Moore Jr, J	8, 9
Morais, C	161, 236, 245, 249
Morcos, K	137
Moreira, L	190
Morosin, M	6
Mouyer, Z	122
Mozalbat, D	11, 321
Mughal, A	268
Mujtaba, Syed S	114, 115
Mulryan, K	310
Muneer, A	12
Murphy, G	39, 65, 78, 127, 145, 161, 189, 191, 203
Murray, S	119
Musab, M	313, 319
Mussa, S	222
Mustaev, M	419
Mustafa, A	118
Muston, B	17
Mylonas, G	347

### N

**Table:** 

Naase, H	149
Naidu, B	234, 365, 405
Naik, S	86, 168
Nair, J	263, 264
Nakas, A	262, 302, 353, 412
Nanjaiah, P	183
Narang, K	280, 291
Narayan, P	143
Narayana, A	182
Nardini, M	358, 410
Naruka, V	29, 108, 179, 388
Nassar, M	205
Nawaytou, O	27, 28, 56, 261
Nawaz, H	284
Neilsen, L	415
Ng Yin Ling, C	125, 169, 171
Ngaage, D	187
Nguyen, B	161
Nichols, S	187
Nicou, N	168
NiDhonnchu, T	181
Nielsen, L	243
Nienabar, C	11
Nimako, K	368
Nithiananthan, M	339
Nizami, M	363, 380
Nkolimbo, C	245
Nolke, L	94
Noonan, P	214, 215
Norkunas, M	332, 338, 340
Normahani, P	298
Ntouskou, M	12
Nunes, Joao P	235
Nwaejike, N	105, 161
Nwakwu, C	109

### O

**Table:** 

O’Mahony, S	327
O'Brien, L	327
O'Dwyer, M	384
Oezalp, F	40
Ohri, S	37, 42, 109, 250
Okorocha, C	42
Olivar, M	70
Olsen, K	216
Oo, A	24, 29, 45, 119
Oo, S	116, 121
Ooues, G	222
O'Regan, D	8
O'Rourke, S	41
Osman, M	241, 427, 444
Othman, A	27, 28, 56, 261
Owen, R	281
Owens, C	174
Owens, G	119
Oyebanji, O	436
Oyebanji, T	43, 137
Ozalp, F	440

### P

**Table:** 

P V S, P	232
Padukone, A	422, 433, 438
Pagliarulo, V	218
Pai, V	427
Pal, S	171
Palima, J	118
Palmares, A	274, 370, 413
Panahi, P	73, 152, 153, 285
Papagiannopoulos, K	409
Papalois, V	75
Parameshwar, J	427
Parry, G	275, 429, 440, 441
Patel, A	307, 361, 388, 405, 413
Patel, N	302
Patel, R	238
Paterson, S	287
Pathak, S	65, 127
Patrini, D	376
Patsalides, Michalis A	287
Paul, I	395
Pelella, G	209, 217
Peng, E	214, 215
Pengelly, S	283
Pepper, J	7, 46
Pereira, C	425
Perikleous, P	309, 336, 387, 410, 423
Perris, R	405
Peryt, A	280, 291, 363
Peters, M	236
Petrie, M	65
Petrou, M	46, 84, 161, 180, 249
Petrov, G	384
Pettit, S	235, 427
Philip, B	161, 373
Phillips, D	137
Pierre, A	366
Pilling, J	380
Pinto, A	344
Pirola, Selene	8
Pirtnieks, A	313, 319, 325, 334, 379
Platt, M	222
Plonek, T	132
Plunkett, D	240
Podd, S	98, 128
Pompili, C	134, 371
Pons, A	274, 326, 370, 413
Poon, S	59, 60, 87, 89
Popescu, F	56
Power, H	19
Price, N	34
Proli, C	274, 370, 388, 413
Punjabi, P	63, 108, 179
Purmessur, R	291
PVS, Prakash	3

### Q

**Table:** 

Qsous, G	351
Quarto, C	57, 199, 249
Quigley, R	235, 241
Qureshi, S	86, 168

### R

**Table:** 

Rafiq, M	427
Rai, K	45
Raj Krishna, G	105, 399
Raj, B	435, 443
Raja, M	288, 289, 290
Raja, S	7, 40, 53, 69, 72, 84, 120, 123, 130, 165, 236, 249
Rajakaruna, C	10, 25, 161
Rajamani, S	3, 232
Rajamiyer, V	197
Rajan, L	255
Ramalingam, A	385
Ramaraju, S	40, 53
Rammohan, K	378, 393, 394, 398, 399
Ramzi, J	127
Rao, A	28
Rao, J	355
Rathinam, S	99, 302, 339, 348, 353, 389, 414
Rathod, V	108
Raubenheimer, H	413
Ravendren, A	276
Ravishankar, R	63
Redmond, K	247, 252, 257, 310, 316, 317, 357
Redondo, A	224, 226
Reilly, S	190
Rescigno, G	118
Reynolds, A	273, 281
Rice, D	94, 221, 345, 407
Richards, T	12
Ridley, P	183
Ripoll, B	55
Rizzello, A	127
Rizzo, V	34, 100, 110, 120, 161
Roberts, N	83
Robinson, P	190
Robson, J	222
Rochon, M	161, 236, 245, 249, 306
Rodrigues, G	442
Rogers, L	98, 128, 161
Rogers, V	365, 405
Roman, M	78, 127, 191
Rooney, S	238
Rose, D	48
Rosendahl, U	7, 46
Rowe, H	109
Rowe, M	24
Rowe, S	351
Rubino, A	235
Ryan, R	384, 403

### S

**Table:** 

Saad, H	219, 382
Sabeshan, P	108
Sabetai, M	30, 34, 110
Sadia Aftab, S	137
Sadler, C	238
Saftic, I	375
Sahai, P	17
Sahdev, N	165, 386
Sajic, M	145
Salem, A	24, 155
Salih, C	208
Sallam, M	34
Salmasi, M Y	7, 8, 9, 11, 40, 57, 63, 120, 130
Salmasi, Mohammad Y	30, 194, 199
Salmasi, Y	6, 53
Samaddar, A	213
Samaraweera, D	157
Sanders, J	119, 228
Sandhu, M	135, 138, 144
Santhirakumaran, G	322, 331
Santhosh, G	3
Saravanan, P	177
Sarvananthan, S	42
Sasidharan, S	8, 9
Sayeed, R	161, 190
Scarrott, H	164
Schweigert, M	303, 305
Seale, A	268
Sebastian, L	256
Sef, D	431, 435, 443
Selvaraj, S	3, 232
Senage, T	50
Senbaklavaci, O	441
Seraj, Shaikh S	152, 285
Sereda, V	70
Shaarawy, E	332
Shackcloth, M	373, 394, 396, 398
Shah, B	167
Shah, M	322
Shah, O	106, 300
Shah, P	164
Shah, S	371
Shah, T	307, 365, 405
Shahansha, S	3
Shanahan, B	247, 357
Shanmugananthan, S	155
Shannon, J	57
Sharkey, A	159, 160, 176
Sharma, S	58, 59, 60, 89
Shatila, M	361
Shaw, M	12, 23, 24, 27
Shehata, M	34, 100, 110
Sheikh, A	137
Sheikh, S	189
Shenoy, R	391
Sheridan, N	244, 257
Sherif, M	51, 95
Shetty, G	211
Shetty, V	3, 232
Shinn, O	250
Shipolini, A	45
Shirke, M	284
Shoeib, M	163
Siepe, M	50
Simmonds, S	402
Simoniuk, U	12, 29
Singh, H	238
Singh, S	332
Singhania, A	161, 378, 393
Sinha, S	10, 16, 25, 67, 76, 77, 79, 102, 104, 135, 138, 143, 222, 223
Sinnott, N	394
Sinobas, A	272
Sitaranjan, D	444
Skulbedova, N	263, 264
Slim, N	194
Smail, H	84, 428, 438, 439
Smelt, J	321, 331, 368
Smith, B	215
Smith, E	328
Smith, H	5, 416
Smith, M	396, 398
Smith, S	405
Sobhun, G	70
Socci, L	355
Soh, Karen Chien L	346
Soliman, N	197
Soliman-Aboumarie, H	425
Somasundram, K	332
Soo, A	74, 374
Sorathia, N	216
Sorensen, J	357
Sounderajah, V	298
Spear, M	287
Speggiorin, S	208
Spence, A	312, 333
Spence, M	174
Spieth, P	303
Spinthakis, N	98
Spyridopoulos, I	73
Srinivasan, L	312, 333, 401
Sriskandarajah, S	56
Stamenkovic, S	357, 386, 410
Stavroulias, D	411
Stefano, P	50
Stein, H	305
Sterne, J	104
Steyn, R	365
Stickley, J	268
Stock, U	9, 84, 199, 428, 431, 433, 435, 438, 439, 443
Stockdale, S	364
Streets, E	233
Strickland, J	314
Struzik, E	223
Suhail, S	60
Sunny, J	225, 284
Surendran, A	445
Suseeladevi, A	223
Sutherland, F	70
Syed Aidil H	52, 103
Syed Nong C	52, 103
Szafranek, A	86, 168
Szafron, B	219, 382, 385

### T

**Table:** 

Taberham, R	411
Tafuro, J	183
Taghavi, J	157
Tahhan, G	334
Takyi, C	175
Talukder, S	385, 427
Tan, C	99, 321, 331, 391
Tandon, E	286, 293
Tariq, H	373
Taylor, M	105, 360, 393, 394, 396, 398, 399
Tcherveniakov, P	409
Teh, E	409
Tenconi, S	355
Thammandra, V	186
The RAV Group	35
Thekumkattil T, T	232
Theodore, S	164
Theologu, T	12
Thomas, A	216
Thomson, G C	3
Tincknell, L	388
Toale, C	316
Toerien, L	327, 329, 345
Toh, S	276
Tolan, M	351
Tomkova, K	145
Torella, F	14
Trevarthen, T	201
Trivedi, U	182
Trkulja, V	443
Tseng, Yuan-T	199
Tsin Yan, Grace T	275
Tsitsias, T	366
Tsui, S	427
Ttofi, I	19
Tuff, C	229
Turner, M	222
Turton, M	263, 264
Tyson, N	86, 99

### U

**Table:** 

Uberoi, R	19
Ugur, T	380
Umakumar, K	431, 439
Unsworth-White, J	16, 152
Uppal, R	45
Uy, C	256
Uzzaman, M	92

### V

**Table:** 

Vaja, R	161
Valesco-Sanchez, D	217
Valtzoglou, V	313, 319, 325, 334, 379
van Delden, R	192
van Doorn, C	209, 217
Van Tornout, F	385
Varghese, D	137
Veeralakshmanan, P	152, 285
Velissaris, T	106
Veltink, P	192
Venkateswaran, R	105
Venu, G	232
Venugopal, P	211, 212
Verdichizzo, D	19
Veres, L	420
Verzelloni Sef, A	431, 435, 443
Villaquiran, C	16
Villaquiran, J	16
Viola, C	390
Viola, N	267
Virdi, A	427
Visan, A	205
Vlastos, D	46, 413
Vohra, H	25, 102, 104, 116, 272
Volpi, S	436

### W

**Table:** 

Waddell, T	366
Wadey, K	117
Wald, D	387
Wali, A	16, 161
Walker, A	48, 282, 286, 293
Wallace, W	298
Waller, D	336, 358, 359, 386, 392
Wang, L	218
Wang, Y	254
Waterhouse, B	349, 395, 417
Watson, J	187
Weaver, H	353
Webb, S	131
Webb, V	259
Weedle, R	74, 345, 374
Wells, F	280
Wendler, O	64, 125, 169, 171
West, D	375
Whitaker, D	125, 171
White, A	74, 374
White, R	175
Whittaker, G	347
Whooley, J	74, 374
Wicks, W	31
Wilkinson, G	238
Williams, J	313, 319, 325, 379
Williams, L	363, 424
Wilson, H	386, 387
Wilson, I	161
Wilson, K	161
Wilson-Smith, A	17
Winters, J	434
Witzigmann, H	305
Wong, Qing N	294
Woo, E	315, 344
Woolley, R	209
Wozniak, M	127, 145, 189, 191, 203
Wright, L	302
Wynne, R	228

### X

**Table:** 

Xu, Xiao Yun	8

### Y

**Table:** 

Y Oo, A	12
Yao, L	153
Yap, T	100
Yasufuku, K	366
Yates, M	45
Yeung, J	366
Youhana, A	87
Young, A	371
Young, V	403
Yousef, O	272

### Z

**Table:** 

Zacharias, J	107, 177
Zaidi, A	87
Zakkar, M	39, 151, 161
Zargaran, D	30
Zeschky, C	433, 438
Zibdeh, O	165
Zientara, A	57, 199
Zlocha, V	124
Zouki, J	211

